# Exploiting Continuous Processing for Challenging Diazo Transfer and Telescoped Copper-Catalyzed Asymmetric Transformations

**DOI:** 10.1021/acs.joc.1c01310

**Published:** 2021-08-11

**Authors:** Daniel C. Crowley, Thomas A. Brouder, Aoife M. Kearney, Denis Lynch, Alan Ford, Stuart G. Collins, Anita R. Maguire

**Affiliations:** † School of Chemistry, Analytical and Biological Chemistry Research Facility, University College Cork, Cork, Ireland; ‡ School of Chemistry, Analytical and Biological Chemistry Research Facility, Synthesis and Solid State Pharmaceutical Centre, University College Cork, Cork, Ireland; § School of Chemistry and School of Pharmacy, Analytical and Biological Chemistry Research Facility, Synthesis and Solid State Pharmaceutical Centre, University College Cork, Cork, Ireland

## Abstract

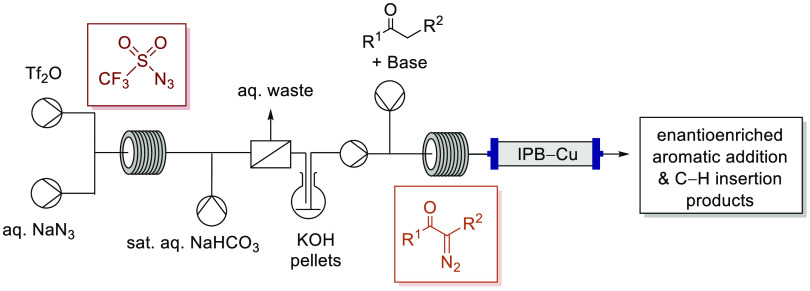

Generation and use of triflyl azide in flow enables efficient synthesis of a range of α-diazocarbonyl compounds, including α-diazoketones, α-diazoamides, and an α-diazosulfonyl ester, via both Regitz-type diazo transfer and deacylative/debenzoylative diazo-transfer processes with excellent yields and offers versatility in the solvent employed, in addition to addressing the hazards associated with handling of this highly reactive sulfonyl azide. Telescoping the generation of triflyl azide and diazo-transfer process with highly enantioselective copper-mediated intramolecular aromatic addition and C–H insertion processes demonstrates that the reaction stream containing the α-diazocarbonyl compound can be obtained in sufficient purity to pass directly over the immobilized copper bis(oxazoline) catalyst without detrimentally impacting the catalyst enantioselectivity.

## Introduction

The transition-metal-catalyzed reactions of α-diazocarbonyl compounds are among the most versatile transformations in organic synthesis.^1−5^ In particular, enantioselective rhodium- and copper-catalyzed processes have attracted significant attention, facilitating the efficient formation of new C–C bonds, among other transformations, in a highly diastereoselective and enantioselective fashion, including C–H insertion,^4,5^ aromatic addition reaction,^1^ and cyclopropanation.^6−8^

Within our research team, highly enantioselective copper-catalyzed C–H insertions, employing bis(oxazoline) ligands, have been reported across a range of substrates leading to thiopyran *S*,*S*-dioxides (up to 98% ee),^9^ sulfolanes (up to 80% ee),^10^ cyclopentanones (up to 82% ee),^11^ and β- and γ-lactams (up to 82% ee),^12^ with particular success using sulfonyl-substituted α-diazoketones and α-diazoesters. Extension of this work to copper-mediated desymmetrization proved highly effective, resulting in formation of the desymmetrized thiopyran *S*,*S*-dioxide in up to 98% ee.^13^

**Scheme 1:**
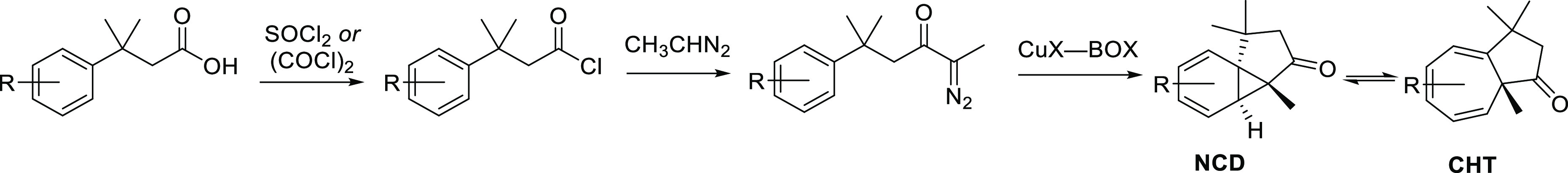
Synthetic Route to Azulenones Previously Reported by Maguire^14,17,18^

Furthermore, the same copper–bis(oxazoline) catalysts have been employed in intramolecular Buchner additions (Scheme 1),^14−19^ leading to azulenones with excellent enantiocontrol (up to 95% ee);^16^ the azulenones exist in a dynamic equilibrium of the norcaradiene (NCD) and cycloheptatriene (CHT) forms through reversible electrocyclic ring opening/closing, as evidenced by time-averaged ^1^H NMR signals.^20,21^ Formation of the quaternary bridgehead stereocenter with excellent enantiofacial control through use of the copper bis-oxazoline catalysts is very attractive from a synthetic perspective.

Despite the synthetic versatility of α-diazocarbonyl compounds, their use at scale has been limited by safety concerns around the potentially hazardous nature of these compounds and, more particularly, their sulfonyl azide or diazoalkane precursors.^22,23^ For example, the practical synthetic utility of the copper-mediated intramolecular Buchner research in Scheme 1 is impacted by the requirement to utilize a diazoalkane, in this instance diazoethane, to generate the α-diazoketone. To address this challenge, efforts have been made to establish continuous processing methodologies for in situ generation and use of diazo compounds and, by extension, for in situ preparation of the requisite diazo-transfer reagents.^24−27a^ Of the reports of the diazo-transfer process in flow, however, most have employed sulfonyl azides directly as reagents.^28−31^ The improved safety profile associated with the handling and use of hazardous compounds in flow chemistry platforms has been a critical aspect of the increasing emergence of these technologies. Enhanced process control,^24,31−36^ due to automation, in-line reaction monitoring, and the superior efficiency of mass and heat transfer in continuous processes—principally, a consequence of the high surface-area-to-volume ratios inherent in pipe or tube reactors—together with the specific facility for immediate use of hazardous material, generated in situ in minimal quantities,^37^ has enabled synthetic chemistry approaches that would previously have been dismissed due to the associated risks at large scale.

**Scheme 2:**
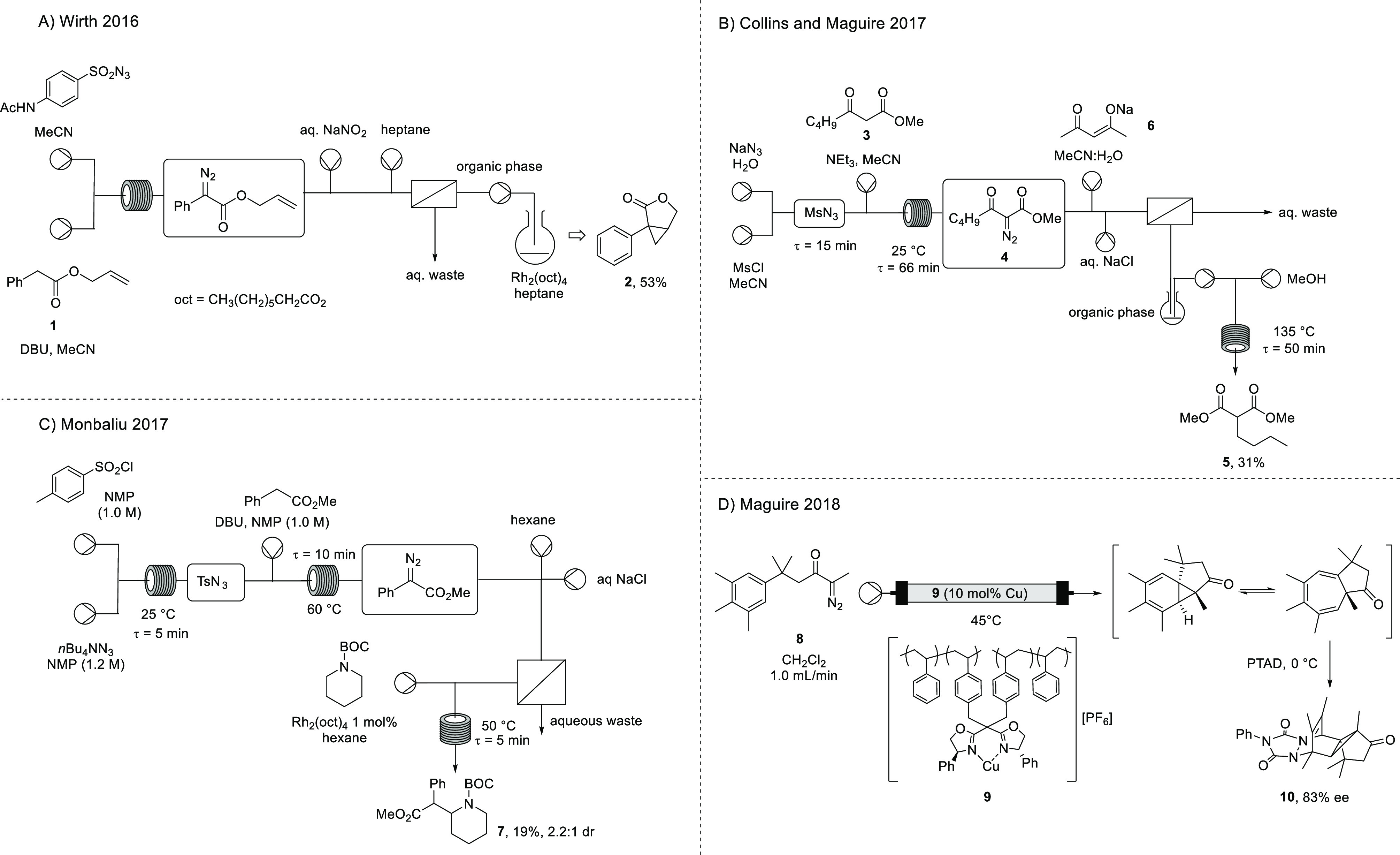
Previous work by Wirth,^28^ Collins and Maguire,^39^ Monbaliu,^40^ and Maguire^14^

Wirth has reported a two-step telescoped process where batch-generated sulfonyl azide was used for diazo transfer to an aryl ester **1** which, following liquid–liquid separation, was flowed into a round-bottom flask containing a rhodium(II) catalyst, affording a racemic cyclopropanation product **2** (Scheme 2A).^28^ Wirth’s method included a drying trap with MgSO_4_, which notably improved yield, although side product formation was still observed.

To overcome the requirement to synthesize (in advance) and handle sulfonyl azides, we have demonstrated successful in situ generation of tosyl or mesyl azide in flow telescoped with diazo transfer to a range of substrates in good to excellent yields and including development of a sodium acetylacetonate **6** quench system to remove any unreacted sulfonyl azide from the reaction outflow following the diazo transfer.^38,39^ As illustrated in Scheme 2B, the generation of mesyl azide, diazo transfer to the β-ketoester **3**, and subsequent transformation of the α-diazo-β-ketoester **4** in a thermal Wolff rearrangement with trapping of the ketene by methanol has been successfully telescoped in a continuous flow process establishing that α-diazocarbonyl compounds can be generated and used in flow without isolation and handling of either the α-diazocarbonyl compounds or, critically, the sulfonyl azide precursors.

Subsequently, Monbaliu has described a telescoped process for the synthesis of BOC-protected methylphenidate, starting with in situ TsN_3_ preparation followed by Regitz diazo transfer and rhodium(II) octanoate catalyzed intermolecular C–H insertion leading to the product **7** in 19% yield (Scheme 2C).^40^ An alternative telescoped synthesis was also reported whereby the thermolysis of a tosyl hydrazone followed by intramolecular C–H insertion and β-lactam methanolysis afforded the deprotected derivative of **7** in a much improved yield of 70%.^40^

Over the past two decades, there has been tremendous progress in immobilization of rhodium carboxylate and carboxamidate catalysts,^41−46^ with significant contributions from Davies,^47−50^ Doyle,^51,52^ and Hashimoto,^53^ among others. Davies has reported in situ formation of aryl α-diazoesters in flow, via oxidation of the corresponding hydrazones, followed by telescoped intermolecular cyclopropanation or C–H insertion with an immobilized rhodium(II) carboxylate catalyst.^50^ Excellent yields and enantioselectivities were reported which matched the results from the corresponding batch reactions.

Copper-catalyzed transformations utilizing heterogeneous immobilized bis(oxazoline) ligands have also been investigated,^54−59^ including the immobilization of a (4*S*)-Ph-bis(oxazoline) ligand onto laponite clay by electrostatic interactions, which was used in the intermolecular C–H insertion into THF. Only a handful of reports, however, have described immobilized copper-catalyzed transformations of α-diazocarbonyl compounds performed in flow.^60,61^ Following the precedent of Burguete^62^ for generation of an insoluble polymer bound (IPB) copper–bis(oxazoline) catalyst **9**, our group has demonstrated its use for enantioselective intramolecular Buchner reactions in batch and using continuous flow processing, in up to 83% ee (Scheme 2D);^14^ critically, the immobilized copper catalyst could be washed and reused a number of times.

To the best of our knowledge, however, a fully telescoped continuous flow process involving formation of the sulfonyl azide followed sequentially by diazo transfer and a copper-catalyzed process has not been reported to date. A key challenge is to ensure that the reaction outflow from the diazo transfer is amenable to direct exposure to the enantioselective transition-metal catalyst. In particular, removal of any reaction components from the diazo-transfer process—sulfonamide, base, and any unreacted sulfonyl azide—that could negatively impact on the transition-metal-catalyzed transformation through metal leaching, catalyst poisoning, or complexation leading to reduced activity and/or enantioselectivity must be addressed; in addition, the removal of water is critical to avoiding competing O–H insertion pathways. Furthermore, for efficient telescoping, conducting the sulfonyl azide generation, prior to the diazo transfer, in a solvent which is compatible with the enantioselective transition metal-catalyzed step is critically important.

In general, it is evident that any telescoped process using diazo compounds, generated in flow, in a downstream transition-metal catalysis requires a diazo feed stream that is (1) in a suitable solvent for the desired transition metal-catalyzed process, (2) free of any byproducts and reagents from upstream processes, and (3) sufficiently dry to undergo water-sensitive transformations.

Our objective was, therefore, to further develop our earlier telescoped continuous sulfonyl azide synthesis and diazo-transfer methodologies,^38,39^ which did not completely fulfill these criteria (vide infra), to deliver a process that would enable the in situ generation of α-diazoketones through diazo transfer to be directly linked with copper-catalyzed downstream transformations utilizing IPB copper–BOX catalyst **9**. Specifically, aromatic addition and C–H insertion were the transformations of interest. As part of this work, we also wished to develop a versatile route which could be employed for the synthesis of α-diazoketones such as those illustrated in Scheme 1, possessing a simple methyl substituent, but also for more diverse α-diazocarbonyl compounds bearing additional substituents, including α-diazo-β-ketonitriles.

Herein, we report the successful telescoping of triflyl azide generation and use in the synthesis of a range of different α-diazocarbonyl compounds including α-diazoketones **8**, **11**–**13**, an α-diazolactam **29**, α-cyano-α-diazoacetamides **55**–**62**, an α-diazo-β-ketonitrile **38**, and an α-diazosulfonyl ester **63** and demonstration of their direct use in three enantioselective copper-mediated transformations, thereby establishing the feasibility of this approach.

## Results and Discussion

**Scheme 3:**
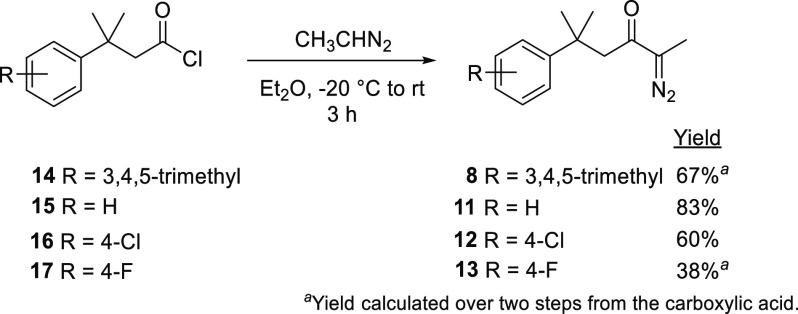
Synthesis of α-Diazoketones **8** and **11**–**13** in Batch^14^

α-Diazoketones **8** and **11**–**13** were selected for the initial investigations in this work, with a view to telescoping the in situ generation of these compounds with their direct use in copper-catalyzed aromatic addition in continuous flow. Prior to this work, the diazoethane acylation route to α-diazoketones **8** and **11**–**13**, summarized in Scheme 3,^14^ has been utilized rather than diazo transfer (in order to overcome the insufficient activation afforded by a single ketone group), but while effective, this route suffers from a number of challenges; these include the safety hazards associated with diazoethane and its precursor, *N*-ethyl-*N*-nitrosourea, which limit its scalability.^63^ Furthermore, this process affords the α-diazoketones in diethyl ether, while dichloromethane is the usual choice of solvent for copper-catalyzed aromatic additions.^17^ An alternative method that avoids the use of diazoalkanes and which is more versatile in the choice of solvent was, therefore, highly desirable, enhancing compatibility with downstream reactions.

**Figure 1:**
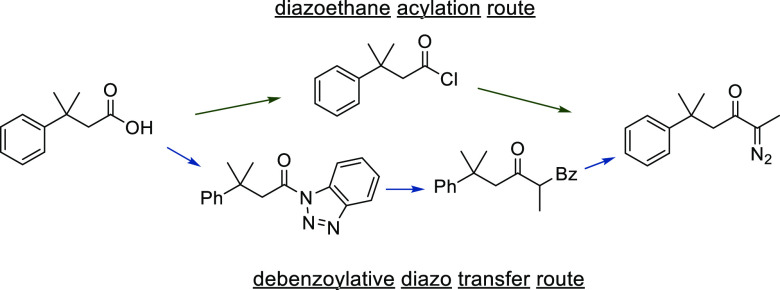
Diazoethane acylation route vs debenzoylative diazo-transfer route.

Debenzoylative diazo transfer for preparation of α-diazoketones **8** and **11**–**13**, as pioneered by Taber,^64,65^ was explored (Figure 1); an adaptation of this route for a continuous flow platform would offer significant advantages over the diazoethane acylation route, obviating use of hazardous diazoalkanes and dramatically improving the safety profile of the process. Dichloromethane is typically employed as the reaction solvent for these transformations, which would be compatible with subsequent telescoped aromatic addition or C–H insertion, while allowing further scope to use alternative solvents instead. This proposed route would exploit sulfonyl azide diazo-transfer reagents, which the group has experience in generating and using in flow.^38,39^

### Synthesis of α-Diazoketones by Debenzoylative Diazo Transfer

**Scheme 4:**
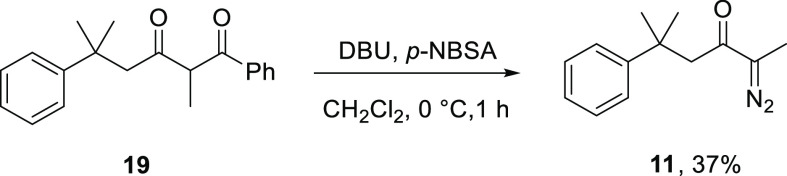
Synthesis of α-Diazoketone **11** by Debenzoylative Diazo Transfer in Batch

Initial studies focused on debenzoylative diazo transfer to β-diketone **19** (prepared via the corresponding acyl benzotriazole^66^) conducted in batch, prior to investigation in flow. Thus, 2,5-dimethyl-1,5-diphenylhexan-1,3-dione (**19**) was transformed to α-diazoketone **11**, employing DBU as base and *p*-nitrobenzenesulfonyl azide (*p*-NBSA) as diazo-transfer reagent in dichloromethane at 0 °C (Scheme 4), in an adaption of Taber’s original route.^64^ Purification of α-diazoketone **11** proved challenging due to coelution with the sulfonyl benzamide byproduct resulting in 37% yield, which was later improved through alteration of the sulfonyl azide (vide infra).

Extending this process to flow (Table 1, entry 1) was first undertaken using peristaltic pumps matching the “in batch” reaction conditions with the exception of temperature; the reaction was performed at room temperature for convenience due to the favorable dissipation of heat in continuous flow. A residence time of 1 h proved sufficient for complete consumption of the starting material. As the outflow of the diazo transfer was ultimately envisaged to be directly used in a transition-metal-catalyzed reaction, it was important to remove DBU from the reaction stream to prevent complexation with metal catalysts, compromising the subsequent transformation. Accordingly, the reactor effluent was flowed through a plug of silica gel prior to collection. As with the batch process, it proved challenging to completely remove the sulfonyl benzamide byproduct and **11** was afforded in a comparable 36% yield; this result confirmed, however, that conducting this diazo transfer in continuous flow and passing the effluent through silica gel had no detrimental impact on the reaction outcome.

**Table 1:**
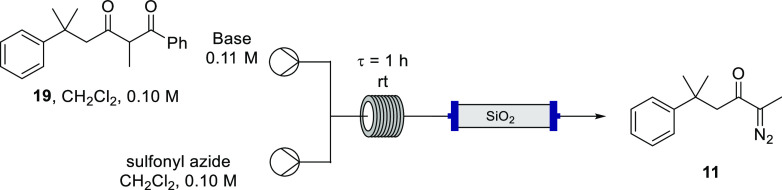
Optimization of Debenzoylative Diazo Transfer to Diketone **19** in Flow

entry	base	sulfonyl azide	temp (°C)	yield (%)
1	DBU	*p*-NBSA	rt	36
2^a^	Amberlyst A21	*p*-NBSA	rt	b
3^a^	polymer-bound DBU	*p*-NBSA	rt	b
4	triethylamine	*p*-NBSA	rt	b
5	tetramethylguanidine	*p*-NBSA	rt	18
6	1,4-diazabicyclo[2.2.2]octane	*p*-NBSA	rt	b
7	*N*,*N*-diisopropylethylamine	*p*-NBSA	rt	b
8	DBU	*p*-NBSA	45	54
9	DBU	MsN_3_	45	25
10	DBU	TsN_3_	45	53

a Solid base was packed in a 6.6 mm diameter glass column, and the combined 1,3-diketone **19**/ *p*-NBSA stream was passed through it.

b No signals for α-diazoketone product **11** were observed in the ^1^H NMR spectrum of the crude product mixture; only unreacted starting material **19** was recovered.

As summarized in Table 1, investigation of a range of bases was undertaken, none of which led to a better outcome than DBU. Interestingly, diazo transfer was not observed when immobilized DBU was used. Use of sodium hydride, which had proved effective under batch conditions,^67^ could not be readily employed in flow.

**Scheme 5:**
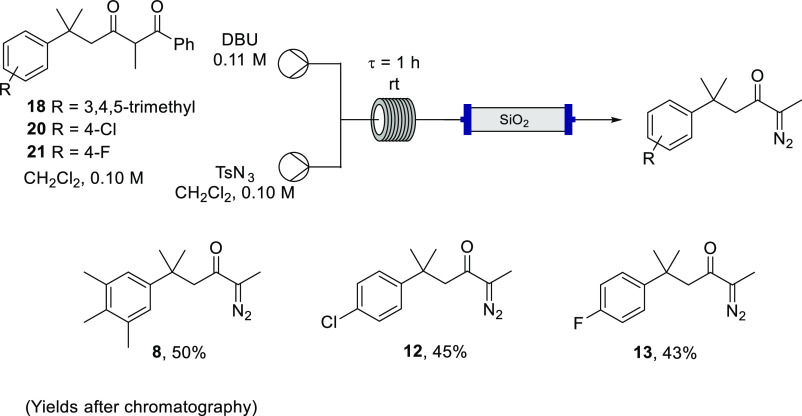
Debenzoylative Diazo Transfer to Diketones **18**, **20**, and **21** in Flow

An improved yield (54%, Table 1, entry 8) was observed when the temperature in the reactor coils was elevated to 45 °C. Methanesulfonyl azide (MsN_3_) proved to be a poor choice of diazo-transfer reagent for this transformation; however, use of *p*-toluenesulfonyl azide (TsN_3_) (53%, Table 1, entry 10) gave a result comparable to that of *p*-NBSA (Table 1, entry 8). TsN_3_ was also a more judicious choice of diazo-transfer reagent than *p*-NBSA as its sulfonyl benzamide byproduct was more easily removed during flash chromatography, allowing clean fractions of α-diazoketone **11** to be more readily obtained. Using this methodology, employing TsN_3_ on continuous flow, the aryl-substituted α-diazoketones **8**, **12**, and **13** were afforded in similar yields from their corresponding 1,3-diketones and DBU (Scheme 5).

Despite the encouraging results with these improved conditions, the yields obtained remained moderate at best (**11**, 53%, Table 1, entry 10 cf. 37% in batch, Scheme 4 vs 83% for diazoethane acylation from the acid chloride, Scheme 3).

As the reaction was envisaged to be part of a telescoped process, the diazo-transfer reagent would ideally be generated in the solvent to be used in the downstream diazo transfer and copper-catalyzed transformations; aromatic addition reactions are typically conducted in dichloromethane with a significant decrease in chemo- and enantioselectivity observed with other solvents.^14^ In particular, acetonitrile has been found to inhibit homogeneous copper–bis(oxazoline) catalysis of aromatic addition and to be incompatible with the IPB copper catalyst **9**, causing leaching of copper.^14^ Tosyl azide is prepared in flow in aqueous acetonitrile,^38^ or NMP,^40^ and in batch in aqueous acetone,^68^ solvents which are not readily compatible with transition-metal catalysis downstream.

Trifluoromethanesulfonyl azide (**22**) (triflyl azide, TfN_3_) is a powerful diazo-transfer reagent that reacts even with highly stabilized acceptor molecules.^69^ In addition to its potent reactivity, it is versatile due to the variety of organic solvents in which it can be generated, including dichloromethane, toluene, hexane, acetonitrile, pyridine, MeOH, and EtOH;^69−75^ this is a desirable attribute for diazo transfers that are part of telescoped processes and which necessitate the α-diazocarbonyl compound to be in a requisite solvent for the “end-of-line” transformation. However, there are inherent risks associated with triflyl azide (**22**) and its byproducts and *extreme caution should be exercised during its preparation and use*; it is a toxic compound and is known to detonate if not handled correctly.^23,73^ Where appropriate procedures are followed, however, it can be used in a safe manner (see the Supporting Information (SI) for further discussion on safety considerations on using triflyl azide in flow), facilitating the preparation of many otherwise difficult-to-access diazo substrates.^70,74,75^

**Scheme 6:**
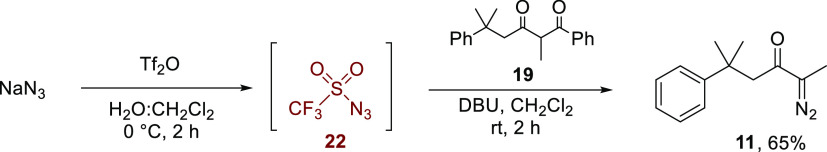
Debenzoylative Diazo Transfer to Diketone **19** Using Triflyl Azide (**22**) in Batch

Triflyl azide (**22**) is prepared by the reaction of triflic anhydride with sodium azide. For the purpose of this work, the “in batch” preparation of triflyl azide (**22**) was carried out using a slightly modified version of the procedure reported by Xu,^70^ whereby the sulfonyl azide was generated with sodium azide (5.0 equiv) and triflic anhydride (1.1 equiv relative to **19**) in a biphasic (2:1) water/dichloromethane mixture at 0 °C for 2 h. The layers were separated, and then the dichloromethane solution of triflyl azide (**22**) was used directly in the diazo-transfer step (Scheme 6).

There are important safety and process considerations when carrying out the process shown in Scheme 6. Triflyl azide (**22**) should never be isolated in pure form, and the organic solvent should not be removed from its solutions, as this is known to lead to detonation.^73^ The separated organic phase was washed with aqueous sodium bicarbonate to neutralize any triflic acid that may be present and which could degrade the subsequent α-diazoketone products. The resulting triflyl azide solution was added slowly (20–30 min) to a dichloromethane solution of 1,3-diketone **19** and DBU at room temperature, and reaction progression was monitored by IR spectroscopy. After 2 h, complete disappearance of the azide stretch (∼2150 cm^–1^)^73^ was observed, and the pure α-diazoketone **11** (ν(C=N_2_): 2061 cm^–1^) was isolated in 65% yield. The excess of sodium azide used in the reaction and remaining in the aqueous layer was quenched by the literature method.^76^

**Scheme 7:**
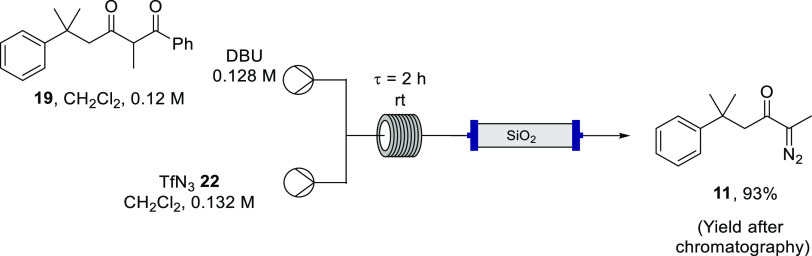
Debenzoylative Diazo Transfer to Diketone **19** in Flow Using Triflyl Azide (**22**)

Given this much improved yield for the debenzoylative diazo transfer through use of triflyl azide, the diazo-transfer element of the process was initially trialled in flow (Scheme 7). Triflyl azide (**22**) was generated in batch, as described above (Scheme 6); the resulting dichloromethane solution of triflyl azide (**22**) was pumped to a T-piece to meet a solution stream of **19** and DBU, and then the combined stream was pumped through a coiled reactor for a residence time of 2 h at room temperature. Use of a peristaltic pump proved effective, while HPLC (piston) pumps were found to be less effective. The orange effluent stream was passed through a silica gel plug, collected, and checked by IR spectroscopy to verify complete consumption of triflyl azide (**22**) prior to concentration.

The desired α-diazoketone **11** was afforded in 93% yield after chromatography. ^19^F NMR spectra were recorded for the isolated product to ensure that no residual triflyl azide or other fluorinated byproducts, without observable signals in the ^1^H NMR spectrum, were present. There is one report in the literature of the byproduct trifluoromethylsulfonyl benzamide (δ_F_ −76.97);^77^ this compound was not observed or isolated during this work and was presumably retained on the silica gel.

This increase in yield (up to 93%, from 65% for the batch process (Scheme 6) and 53% for the tosyl azide mediated transformation (Table 1, entry 10)) was a significant breakthrough and effectively represented optimized conditions for diazo transfer to form **11** in continuous flow. The improved outcome in flow vs batch can be rationalized due to the superior process control achieved in flow, in this instance, the control over reactant ratios and temperature, specifically the consistent manner in which reagents were combined during the continuous process.

### Telescoped Debenzoylative Diazo Transfer in Flow

**Scheme 8:**
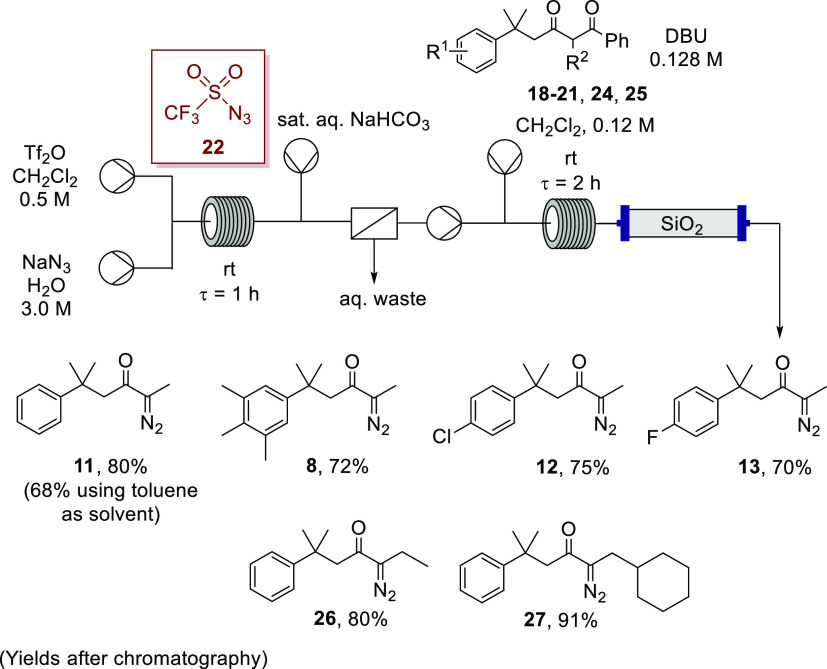
Telescoped Generation of Triflyl Azide (**22**) and Direct Use for Debenzoylative Diazo Transfers in Flow

Having established that use of triflyl azide in flow for diazo transfer was effective, the next challenge was to telescope the preparation of this hazardous reagent in flow with the diazo-transfer step. As illustrated in Scheme 8, the formation of triflyl azide (**22**) and subsequent use in debenzoylative diazo transfer to 1,3-diketones **18**–**21** proved successful, enabling the use of triflyl azide as a powerful reagent for diazo transfer while obviating the need for isolation or handling of this reagent. Focusing on the generation of triflyl azide in flow, based on the Xu procedure for batch preparation of triflyl azide (**22**)^70^ and the successful use of a dichloromethane solution of **22** for diazo transfer in flow (Scheme 7), generation of triflyl azide (**22**) in flow was initially implemented in dichloromethane. Thus, an aqueous solution of sodium azide was pumped to a T-piece to meet a dichloromethane solution of triflic anhydride with the combined biphasic stream passed through a reactor coil for a residence time of 1 h. To avoid the reaction of triflic anhydride with adventitious water, the dichloromethane solution was freshly prepared before use, and any hydrolysis was effectively reduced by charging the reagent solutions at 3 mL min^–1^ to minimize exposure to air during the transfer. The reaction stream then joined a stream of saturated aqueous sodium bicarbonate to remove any triflic acid, and the combined biphasic effluent was then separated by an in-line liquid–liquid separator.

**Scheme 9:**
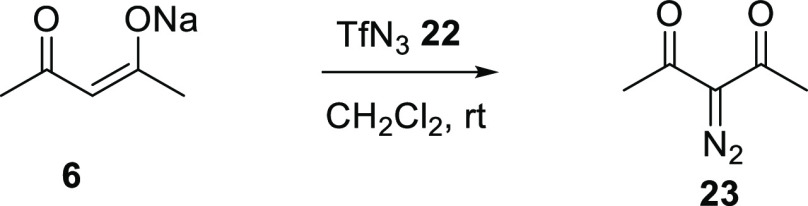
Diazo Transfer to Sodium Acetylacetonate **6**

The reactant ratios for this process were determined by prior experiments involving diazo transfer to a known excess of sodium acetylacetonate hydrate (see the SI for further details), which indirectly established the efficiency of formation of triflyl azide (**22**) as 58% by measuring the extent of diazo transfer to the β-diketonate **6** to form **23** (as shown, more generally, in Scheme 9), without having to isolate and quantify the hazardous sulfonyl azide. The ratio of triflic anhydride to 1,3-diketone **19** required to execute a telescoped synthesis of α-diazoketone **11** in flow, therefore, was estimated to be 1.7:1, respectively. Scheme 8 outlines the continuous flow setup used for the telescoped synthesis of triflyl azide (**22**) and subsequent debenzoylative diazo transfer to 1,3-diketones **18**–**21**, **24**, and **25**. As was noted for the triflyl azide solution (vide supra), use of a peristaltic pump was required.

The conditions for the preparation of triflyl azide (**22**) for this diazo transfer in flow were chosen to deliver similar reactant ratios and concentrations to those successfully employed for the process with pregenerated triflyl azide (**22**) in Scheme 7. The stream containing the triflyl azide after the phase separation was mixed with the solution containing the substrate and DBU and passed through a reactor coil for 2 h. The effluent from the diazo transfer was again pumped through a column reactor of silica gel at room temperature (for removal of polar reaction components), and the collected reaction solution was checked by IR spectroscopy to ensure complete consumption of triflyl azide (**22**). The process was first implemented for debenzoylative diazo transfer to diketone **19**, and gratifyingly, α-diazoketone **11** was isolated reproducibly in 80% yield, albeit slightly lower than the yield recorded for the flow process using a “preformed” triflyl azide solution (93%, Scheme 7). This result, however, was still a considerable improvement on other batch diazo-transfer processes investigated with alternative diazo-transfer reagents during this work. The reduced yield for the telescoped method is most likely due to the presence of residual water in the organic stream, compared to the pregenerated solution of triflyl azide in Scheme 7, which was dried when prepared in batch prior to use in the diazo transfer. For later telescoped processes, involving downstream transition-metal catalysis, this observation was addressed through incorporation of a drying agent into the process (vide infra).

On the basis of the successful synthesis of α-diazoketone **11** using this telescoped preparation of triflyl azide **(22**) and subsequent diazo transfer in flow, this process using identical conditions was applied to each of the other precursors identified for this study at the outset, namely 1,3-diketones **18**, **20**, and **21** (Scheme 8), with good yields achieved for α-diazoketones **8**, **12**, and **13** (70–80%, after chromatography). The flow process was also used to synthesize novel α-diazoketones **26** and **27** in excellent yields (80% and 91%, respectively) from their 1,3-diketone precursors **24** and **25** (Scheme 8), indicating that the process is efficient even when the steric demand of the alkyl substituent at the site of reaction is increased. The flexibility of this route to α-diazoketones bearing different substituents on the diazo carbon is a clear advantage, avoiding generation and use of a series of diazoalkanes to access differently substituted α-diazoketones. The precursor diketones **24** and **25** were readily accessed using the route employed for **19**.

The yields obtained for the telescoped flow process with triflyl azide (**22**) were generally comparable with those of the diazoethane acylation route (from the preformed acid chlorides), although in the case of the 4-fluoro α-diazoketone **13**, the yield from the telescoped TfN_3_ flow process offered a far superior yield (Table 2, entry 4, 70% vs 38%). Previously, we reported the synthesis of α-diazoketone **13** in just 38% yield.^14^ The yields for the telescoped triflyl azide flow process were also higher than those recorded when using batch prepared tosyl azide as diazo-transfer reagent in flow. Notably, the yields of **11** and **13** obtained using the telescoped TfN_3_ flow process were higher than those obtained using TfN_3_**22** in the corresponding batch reaction, highlighting a synthetic advantage in addition to the enhanced safety through generation and use in situ (Table 2, entries 2 and 4).

**Table 2:** Comparison of Methods for Synthesis of α-Diazoketones **8** and **11**–**13**

entry	diazo	flow yield (in situ TfN_3_) (%)^a^	flow yield (TsN_3_)(%)^a^,^b^	batch yield (TfN_3_)^a^ (%)	batch yield (diazoethane)^c^,^d^ (%)
1	**8**	72	50		69
2	**11**	80	53	65	76
3	**12**	75	45		62
4	**13**	70	43	44	38

a Yield after flash chromatography, based on the 1,3-diketone.

b TsN_3_ was preprepared in batch.

c Diazoethane acylation using an acid chloride.

d Yield after flash chromatography, based on the acyl chloride.

**Figure 2:**
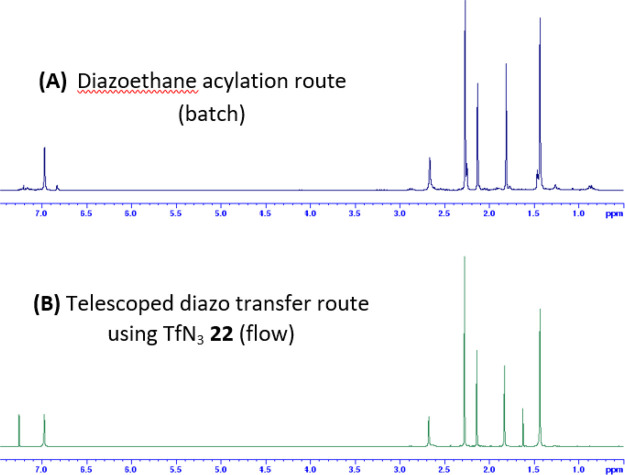
Comparison of the ^1^H NMR spectra of α-diazoketone **8** recorded after chromatography following synthesis in batch with diazoethane (A) and following synthesis with the telescoped TfN_3_ methodology in flow (B). Spectra recorded in CDCl_3_ at 400 MHz (showing residual CHCl_3_ signal at δ_H_ 7.26).

It was noted that the chromatographic purification of α-diazoketones **8** and **11**–**13**, synthesized by the telescoped TfN_3_ flow methodology, afforded cleaner isolated products than those obtained from chromatographic purification of the crude products from the diazoethane acylation route; in particular, isolated α-diazoketone **8** was afforded in significantly improved purity than from the diazoethane acylation route (Figure 2).

With downstream processes in mind, ease of purification is an important benefit. Byproducts may prove to be detrimental to a transition-metal-catalyzed transformation in a telescoped process; the telescoped diazo-transfer process with triflyl azide (**22**) furnished the crude α-diazoketones with minimal byproducts. The ^1^H NMR spectra of the crude effluent, before being passed through the in-line silica gel plug, indicated the presence of just the α-diazoketone and DBU salts (which are removed by passage through a silica gel plug) with no other substantive byproducts.

Notably the telescoped generation of triflyl azide and debenzoylative diazo transfer can be effected in toluene in place of dichloromethane, affording α-diazoketone **11** in 68% yield after chromatography, with just a minor decrease in yield relative to that in the chlorinated solvent (80%) (Scheme 8). Both toluene and dichloromethane are compatible with the downstream copper-mediated transformations. In addition to the advantage of using a more environmentally friendly solvent,^78,79^ there is also a very significant safety benefit, as the risk of formation of diazidomethane during the formation of the triflyl azide is completely eliminated. For all of the processes described herein, the aqueous sodium azide solution and dichloromethane are typically in contact for less than 2 h; there are no reported instances of diazidomethane forming at room temperature in this time frame and there are multiple examples in the literature of sodium azide and dichloromethane being safely used together under similar conditions.^70,80,81^ An in situ IR study was also undertaken, which supported this assertion (see the SI).

An important difference between the telescoped generation of triflyl azide (**22**) and our previously reported method for mesyl azide generation in flow^39^ is that the liquid–liquid separation, to remove the aqueous stream, is carried out prior to the diazo-transfer step. This change was made as, in contrast to the homogeneous aqueous acetonitrile medium used for mesyl azide generation, a biphasic system (CH_2_Cl_2_/toluene/water) might lead to inefficient mixing between the reagents during the diazo-transfer step. The aqueous waste from the liquid–liquid separator can also be safely destroyed in a continuous manner (see the SI for experimental details), based on the literature method.^76^

The telescoped triflyl azide generation and debenzoylative diazo-transfer methodology affords a vastly improved safety profile relative to use of diazoalkane acylation, particularly when toluene is employed as the reaction solvent. Isolation and handling of hazardous diazoalkanes and nitrosoureas were avoided, while the organic azide was synthesized in a safe continuous manner without isolation or handling. Furthermore, the diazoethane acylation approach, in terms of overall yield, offers only marginally better results than the telescoped TfN_3_ flow process; for example, synthesis of α-diazoketone **11** (Figure 1): 58% yield for diazoethane acylation (over two steps: carboxylic acid → acyl halide → α-diazoketone) versus 50% for debenzoylative diazo transfer (over three steps: carboxylic acid → acyl benzotriazole → 1,3-diketone → α-diazoketone).

**Scheme 10:**
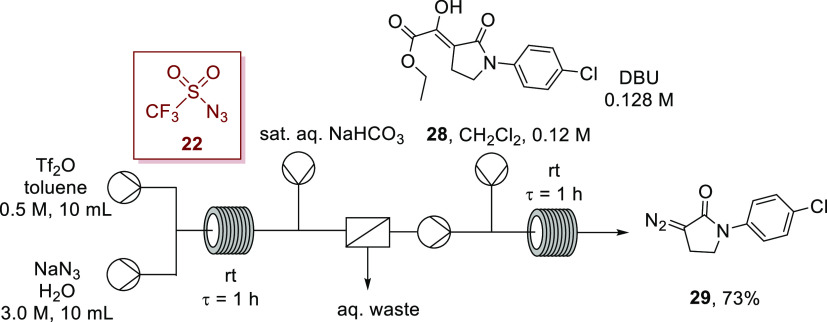
Synthesis of α-Diazolactam **29** via Telescoped in Situ Generation of Triflyl Azide (**22**) and Deacylative Diazo Transfer

The versatility of this telescoped process was further demonstrated by preparation of α-diazolactam **29** from the activated lactam **28**, utilizing a deacylative diazo-transfer strategy,^82,83^ demonstrating that this approach can be generalized beyond debenzoylation (Scheme 10). In this instance, the triflyl azide was generated in toluene while the reagent solution was in dichloromethane indicating the process remains efficient in a mixed solvent system.

### Telescoped Aromatic Additions of α-Diazoketones

With the viability of our continuous flow strategy for generation and in situ use of triflyl azide (**22**) for debenzoylative diazo transfer leading to a range of α-diazocarbonyl compounds established, our attention turned to telescoping this process with copper-mediated asymmetric transformations, with an initial focus on intramolecular aromatic addition.

**Figure 3:**
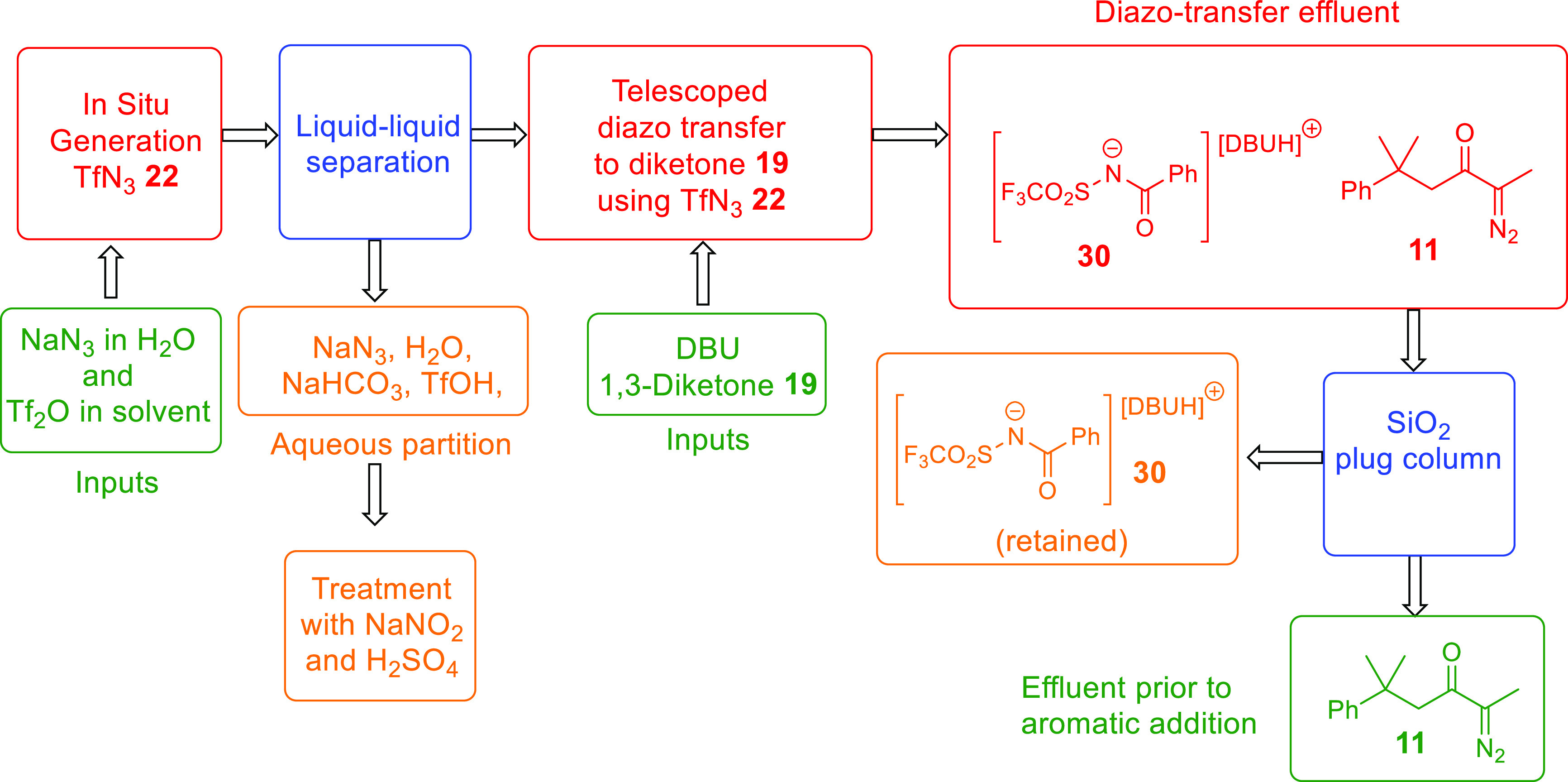
Flowchart for the telescoped generation of TfN_3_**22** and diazo transfer to diketone **19** in flow including the in-line removal of byproducts; required process inputs and desired output are shown in green; process byproducts are shown in orange; steps involving separation of byproducts are shown in blue; components of the process without user handling are shown in red.

Generation of triflyl azide (**22**) and subsequent debenzoylative diazo-transfer reactions in flow provided a crude reaction effluent in a solvent suitable to use in a downstream copper-catalyzed aromatic addition (see Figure 3 for an overview of the overall process). Furthermore, excess sodium azide and any triflic acid were readily removed in the aqueous layer prior to diazo transfer, and subsequently, the organic effluent from the diazo transfer was readily purged of both DBU (or, most likely, its salt **30**, Figure 3) and the sulfonyl benzamide byproduct by passing through a silica gel plug. The sulfonyl benzamide byproduct and DBU could, each, potentially coordinate to the copper catalyst downstream, hindering the transition-metal-catalyzed aromatic additions, and therefore, their removal prior to flowing into the immobilized catalyst bed was critical for successful transition-metal-catalyzed transformation. Indeed, we have established experimentally that residual DBU is not compatible with the immobilized copper catalyst **9**, as passing a diazo-transfer reaction solution containing DBU and its salts through the IPB catalyst **9** (by omitting prior passage through silica gel) was found to lead to high levels of copper leaching—as indicated by a dramatic change of color (green to white) occurring with concomitant loss of catalytic activity.

Evidently, the silica gel column has a finite capacity to retain the polar byproducts; accordingly, the volume of the solution of α-diazoketone passed over the immobilized copper catalyst was adjusted to avoid elution of any of the unwanted polar components.

**Scheme 11:**
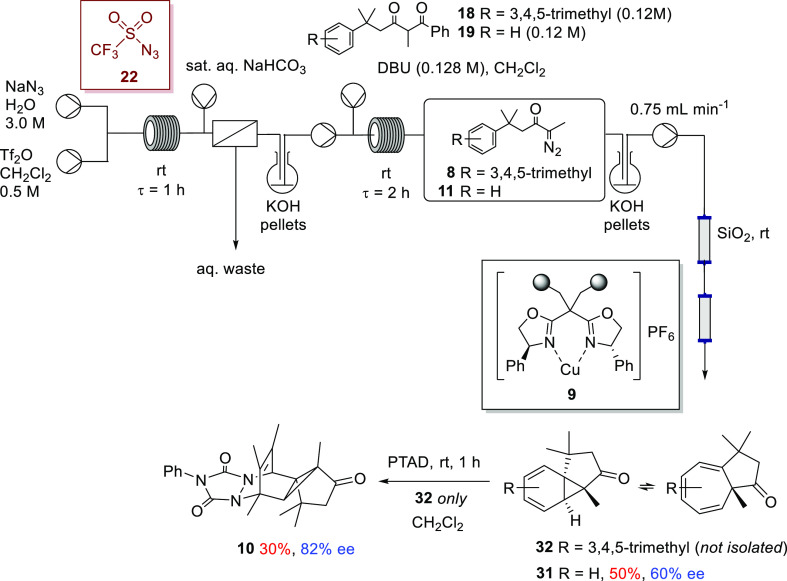
Telescoped Syntheses of α-Diazoketones **8** and **11** with Downstream Copper-Catalyzed Asymmetric Aromatic Addition^85,86^

Two telescoped processes comprising triflyl azide generation, debenzoylative diazo transfer, and aromatic addition were investigated, furnishing enantioenriched azulenone **31** and the PTAD adduct **10** derived from azulenone **32** (Scheme 11). In both tranformations, the triflyl azide (**22**) prepared in flow was passed over KOH pellets in a round-bottomed flask as a reservoir and then pumped forward to effect debenzoylative diazo transfer with either 1,3-diketone **18** or **19**.^84^

The diazo-transfer effluent was also collected over KOH pellets, and an appropriate portion of this reaction solution, containing the α-diazoketone **8** or **11**, was pumped forward to undergo aromatic addition, ensuring the capacity of the silica gel column to retain the polar byproducts, especially the sulfonyl benzamide salts **30**, is not exceeded and thereby avoiding leaching of the copper catalyst (vide supra). Following passage through silica gel, the stream containing α-diazoketone **8** or **11** was directly passed through a packed bed reactor containing IPB catalyst **9** (10 mol %). The aromatic addition of α-diazoketone **11** was performed at 45 °C, while the aromatic addition of α-diazoketone **8** was performed at room temperature.

The process afforded azulenone **31** in 50% yield after chromatography (over the two steps from the 1,3-diketone precursor **19**). Rather than attempt to isolate and purify the labile azulenone **32**, the reactor effluent containing **32** was collected into a flask containing PTAD in dichloromethane to transform it to the stable cycloadduct **10** prior to isolation; once all the effluent was collected, the contents of the flask were stirred for 1 h at room temperature and the product cycloadduct **10** was isolated in 30% yield (over three steps from 1,3-diketone precursor **18**).

Critically, the isolated products **31** and **10** were afforded with comparable enantiopurities (60 and 82% ee, cf. 61% ee and 83% ee) to those reported for the transformations carried out with IPB **9** in batch and flow but which were not telescoped, instead using preprepared α-diazoketone.^14^ These results were encouraging and demonstrated that the telescoped triflyl azide generation and subsequent diazo-transfer reaction produced an effluent, that after treatment with silica gel and drying agent, was sufficiently clean and anhydrous to undergo downstream transition metal-catalyzed transformations with no noticeable impact on enantioselection. While the yields were slightly lower than those achieved with pure α-diazoketones, the benefit of achieving these enantioselective transformations without isolating or handling either the triflyl azide (**22**) or the α-diazoketone offsets this. *These results demonstrate that the synthetic power of enantioselective transition-metal-catalyzed transformations of α-diazoketones can be achieved without handling either the hazardous precursor sulfonyl azide or the α-diazoketone and that the solution of the α-diazoketone generated in flow is sufficiently clean to flow directly into an immobilized copper catalyst without undermining the enantioselectivity*.

**Scheme 12:**
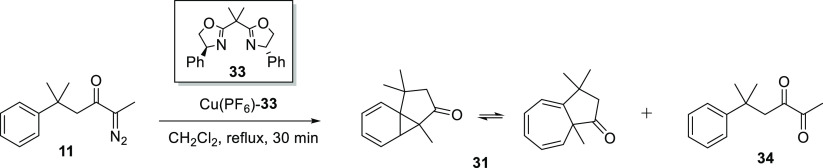


It was notable that no signals associated with DBU or DBU salt **30** were observed in the ^1^H NMR spectra of the concentrated crude reaction effluents, indicating that, at least on the scale investigated, the silica gel column had successfully removed these byproducts. Despite employing KOH pellets as drying agent, 2,3-diketone **34** (Scheme 12) was also afforded as a side product during the aromatic addition of α-diazoketone **11**, and was clearly observed in the ^1^H NMR spectrum of the crude product mixture and was isolated after chromatography in 19% yield as a yellow oil.

Use of KOH pellets as a drying agent was undertaken to combat the impact of residual water in the reaction stream as it passes on to the immobilized copper catalyst. Although the liquid–liquid separator used following the upstream generation of triflyl azide successfully partitions the organic and aqueous phases, the organic layer is not anhydrous at this point. In order to test the effect that the water content of dichloromethane has on aromatic addition, a homogeneous Cu(PF_6_)–**33** [(4*S*)-Ph-BOX] catalyzed transformation of α-diazoketone **11** was carried out (Scheme 12) under identical conditions, but with three different sources of dichloromethane as solvent: (A) dichloromethane which had been freshly distilled over calcium hydride, (B) HPLC grade dichloromethane (with amylene as a stabilizing agent), and (C) HPLC grade dichloromethane (with amylene as a stabilizing agent), spiked with a few drops of water per 100 mL of dichloromethane.

**Figure 4:**
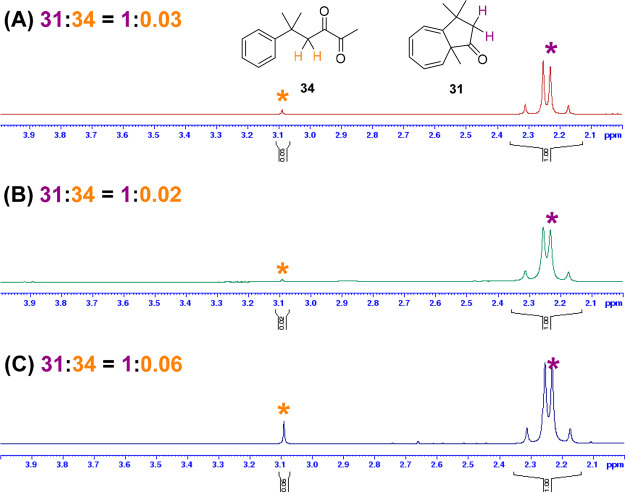
^1^H NMR spectra of the crude reaction mixtures from the aromatic addition of **11** performed in freshly distilled dichloromethane (A), HPLC-grade dichloromethane (B), and HPLC-grade dichloromethane spiked with water (C). Spectra recorded in CDCl_3_ at 300 MHz. The ratio of azulenone **31** to 2,3-diketone **34** was readily determined from the relative integration of the 2H AB quartet of **31** and the 2H singlet of **34**.

The ^1^H NMR spectra of the crude product mixtures of the three transformations indicated increased formation of the 2,3-diketone byproduct **34** in the reaction spiked with water (Figure 4C). The formation of the 2,3-diketone **34** can be envisaged either by reaction of the carbene with oxygen or by O–H insertion into water followed by oxidation of the α-hydroxyketone,^87^ possibly facilitated by the copper catalyst^88^—the outcome of the spiking experiment indicating that water is a more likely source in this instance.

In contrast, the corresponding 2,3-diketone product was not isolated for the telescoped aromatic addition of α-diazoketone **8**, although its formation could not be excluded based on the presence of minor impurity signals in the ^1^H NMR spectrum of the cycloadduct **10** prior to chromatography.

As use of a drying agent, following in-line partitioning of the biphasic stream, to reduce the water content is clearly advantageous, accordingly, in all the telescoped transformations described herein, KOH pellets were placed in the reservoir containing the organic effluent (the solution of triflyl azide (**22**)) from the in-line liquid–liquid separation as it is pumped forward to the diazo-transfer step (Scheme 11). Furthermore, the effluent from the diazo-transfer stream was collected over KOH pellets before being pumped forward to undergo downstream aromatic additions.

During the phase separation in the liquid–liquid separator, a small volume of dichloromethane remains after the triflyl azide separation has been undertaken and the solution progressed downstream to the diazo transfer. To check the efficiency with which the hazardous triflyl azide (**22**) was progressed through (Scheme 11), quantification of the amount of triflyl azide that remains in the residual dichloromethane phase in the separator was undertaken and estimated as approximately 2% of the total generated; this was readily quenched by addition of sodium acetylacetonate (**6**).

### Telescoped Regitz-Type Diazo Transfer in Flow

**Scheme 13:**
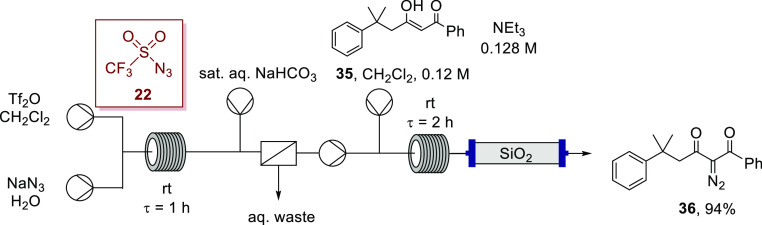
Synthesis of α-Diazoketone **36** with the Telescoped Flow Synthesis Using in Situ Generated Triflyl Azide

While the debenzoylative diazo transfer using triflyl azide generated in flow proved very successful, generalizing this approach to standard Regitz diazo transfer was investigated to broaden the synthetic scope and specifically to enable telescoping with a C–H insertion process (vide infra). Accordingly, Regitz-type diazo transfer to form the novel α-diazo-β-diketone **36** was achieved in an excellent yield of 94% (Scheme 13); in this instance, triethylamine was sufficiently basic to deprotonate β-hydroxyenone **35**. The residence times, reagent ratios, and temperatures were consistent with the previously performed telescoped transformations. In the absence of an alkyl substituent at the α-carbon of the β-hydroxyenone, the reaction mechanism was seen to proceed entirely through Regitz diazo transfer, with no evidence for competing debenzoylation. This result supports the extensive evidence reported by Regitz, Taber and others that the debenzoylation pathway is only observed when the α-carbon possesses an alkyl substituent.^64,65,89−92^

#### Diazo Transfer to a β-Ketonitrile and α-Cyanoacetamides

**Scheme 14:**
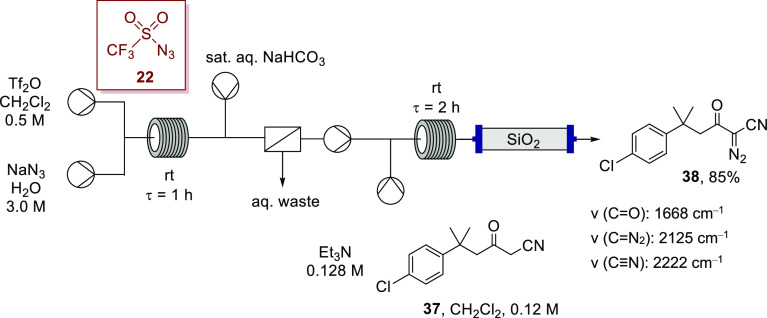
Synthesis of α-Diazo-β-ketonitrile **38** via a Telescoped Flow Synthesis Using in Situ Generated Triflyl Azide (**22**)

Having demonstrated efficient synthesis of α-diazoketones via the in situ generation and use of triflyl azide in flow, extension to a broader range of α-diazocarbonyl compounds was undertaken to expand the scope of this approach with a particular focus on nitrile derivatives which can be challenging to access in certain instances. Accordingly, Regitz-type diazo transfer employing our telescoped triflyl azide generation and diazo-transfer methodology proved successful when employing β-ketonitrile **37** as the substrate, affording the desired α-diazo-β-ketonitrile **38** in 85% yield (Scheme 14); this outcome is comparable with the yields for α-diazo-β-ketonitriles under batch conditions reported by Charette, who had also utilized TfN_3_**22**,^75^ and with the yields obtained when imidazole-1-sulfonyl azide hydrochloride was used.^93,94^ It has also been demonstrated that acylation of diazoacetonitrile can afford α-diazo-β-ketonitriles;^95^ however, this reagent poses its own safety challenges.^96^ Interestingly, a debenzoylative diazo-transfer approach to **38** had proved unsuccessful, with the benzoylated starting material recovered essentially quantitatively.

**Scheme 15:**
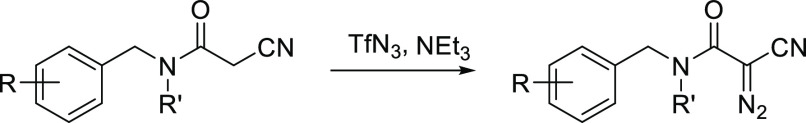
Synthesis Route to α-Cyano-α-diazoacetamides Reported by Xu^70^

Following the successful application of our telescoped triflyl azide generation and diazo-transfer methodology to a Regitz-type diazo transfer, for synthesis of α-diazo-β-ketonitrile **38**, our attention was attracted to its potential for preparation of α-cyano-α-diazoacetamides. The reported synthesis of these α-diazocarbonyl compounds, by Xu,^70^ required the use of triflyl azide (**22**) (Scheme 15), and therefore, these substrates appeared to be clear candidates for preparation via our telescoped flow procedures, without storage or handling of triflyl azide (**22**).

**Scheme 16:**
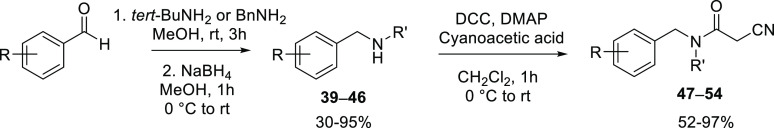
Synthetic Route to α-Cyanoacetamides **47**–**54**

The α-cyanoacetamides **47**–**54** were synthesized by the route described by Xu (Scheme 16).^70^ A range of substituted benzaldehydes were transformed to secondary benzyl amines **39**–**46** by reductive amination with *tert*-butylamine or benzylamine. These amines were then converted to amides in a DMAP-mediated coupling reaction with cyanoacetic acid. Compounds **47**–**54** were prepared on a synthetically useful scale (2–10 g) and were afforded as pure solids, usually upon recrystallization from ethanol, and were stable on storage at room temperature.

A modified version of the method reported by Xu,^70^ with triflyl azide (**22**) and using triethylamine as base, was utilized for synthesis of α-cyano-α-diazoacetamides **55**–**62** in batch (Table 3), as a means of comparing the outcome of their preparation via our telescoped continuous process. As part of this work, the potential viability of tosyl azide (TsN_3_) and DBU, and also *p*-acetamidobenzenesulfonyl azide (*p*-ABSA) and triethylamine, were assessed for the diazo transfer to α-cyanoacetamide **47**, but without success. This outcome highlights the importance of developing a safe method for the generation and use of triflyl azide for diazo transfers which cannot be readily effected using less reactive sulfonyl azides, although Xu had reported a single example of diazo transfer to an α-cyanoacetamide using *p*-ABSA and DBU.^97^

**Table 3:**
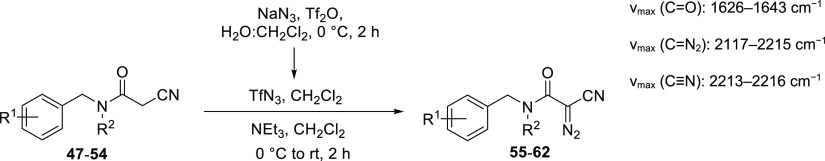
Synthesis of α-Diazo-α-cyanoacetamides **55**–**62** in Batch with Triflyl Azide (**22**)

entry	substrate	diazo	R^1^	R^2^	yield^a^ (%)
1	**47**	**55**	H	benzyl	66
2	**48**	**56**	4-Cl	*t*-Bu	64
3	**49**	**57**	H	*t*-Bu	72
4	**50**	**58**	4-F	*t*-Bu	65
5	**51**	**59**	4-Me	*t*-Bu	83
6	**52**	**60**	4-Br	*t*-Bu	90
7	**53**	**61**	3,5-dimethyl	*t*-Bu	58
8	**54**	**62**	4-pyridyl	*t*-Bu	46

a Isolated after flash chromatography.

The ^1^H NMR spectra of the crude reaction mixtures following concentration were relatively clean in all cases, and the α-cyano-α-diazoacetamides **55**–**62** were isolated after chromatography in yields ranging from moderate to excellent as yellow solids in most cases. These compounds were relatively stable and could be stored for extended periods in a freezer without any evidence of deterioration. Using this protocol, α-cyano-α-diazoacetamides can be readily accessed in multigram quantities, with the primary limitation being the scale of triflyl azide (**22**) handled.

Our telescoped preparation of triflyl azide (**22**) followed by Regitz diazo transfer was then applied to generate the α-cyano-α-diazoacetamides in flow (Table 4). Once again, the organic effluent from the in-line liquid–liquid separator was collected in a flask containing potassium hydroxide pellets before being immediately pumped forward to the diazo-transfer step.

**Table 4:**
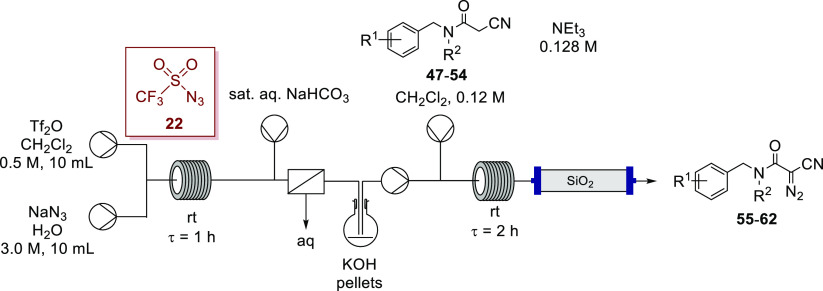
Synthesis of α-Cyano-α-diazoacetamides with the Telescoped Flow Synthesis Using in Situ Generated Triflyl Azide (**22**)

entry	substrate	diazo	R^1^	R^2^	yield^a^ (%)
1	**47**	**55**	H	benzyl	80
2	**48**	**56**	4-Cl	*t*-Bu	95
3	**49**	**57**	H	*t*-Bu	78
4	**50**	**58**	4-F	*t*-Bu	88
5	**51**	**59**	4-Me	*t*-Bu	83
6	**52**	**60**	4-Br	*t*-Bu	84
7	**53**	**61**	3,5-dimethyl	*t*-Bu	96
8	**54**	**62**	4-pyridyl	*t*-Bu	76

a Isolated after flash chromatography.

In practice, synthetically useful amounts (3.0 mmol) of the α-cyano-α-diazoacetamides were prepared and purification by column chromatography was employed to ensure complete removal of the byproducts. Excellent yields were achieved across the series. Furthermore, the quality of the products isolated was comparable in flow or batch. It was notable that the yields for the α-cyano-α-diazoacetamides were more uniform in flow, across the series investigated, than the yields obtained in batch (Table 4 cf. Table 3), presumably due to enhanced control of reagent mixing and temperature.

Introduction of an in-line silica gel plug was undertaken with a view to telescoping the diazo transfer with subsequent transition-metal-catalyzed transformations by removal of the triethylamine salts in-line at smaller scales (approximately 1 mmol). Evidently, the volume of silica gel used in the plug must be appropriate to the scale of the reaction to avoid leaching of the polar byproducts—typically, in this work the reactions were conducted at a 1 mmol scale and using a 100 mm × 10 mm internal diameter glass column of silica gel. Notably, synthesis of gram quantities of α-cyano-α-diazoacetamides using this methodology in flow does not require inclusion of the silica gel column as chromatographic purification affords the products in analytically pure form, with or without the silica gel plug in a flow system.

While triflyl azide (**22**) is the most common choice of diazo-transfer reagent for synthesizing α-cyano-α-diazoacetamides, Reisman has reported using imidazole-1-sulfonyl azide hydrochloride as an alternative in batch,^94^ although the reaction times reported are much longer (24 h vs approximately 4 h) and marginally lower yields were reported in comparison to yields obtained for the telescoped flow process in this work. Most importantly, however, as mentioned earlier, use of triflyl azide (**22**) in flow offers clear safety advantages relative to its generation and use in batch.

### Telescoped C–H Insertion

Earlier work by our team has demonstrated excellent enantiocontrol in intramolecular C–H insertions with α-diazo-β-oxosulfones;^9,13^ the generation and use of triflyl azide in flow for diazo transfer offers for the first time the possibility of telescoping the synthesis of α-diazo-β-oxosulfones with the copper-catalyzed C–H insertion leading to thiopyran *S*,*S*-dioxides. Key to this is the potential to prepare the α-diazo-β-oxosulfones in a solvent medium which is compatible with the catalyst and sufficiently clean to avoid catalyst poisoning or deterioration. Given its successful use for enantioselective intramolecular Buchner reactions in batch and using continuous flow processing,^14^ investigation of the use of IPB copper–bis(oxazoline) catalyst **9** in the copper-catalyzed desymmetrization of α-diazo-β-oxosulfone **63** was investigated.^13^ Use of the immobilized catalyst **9**, in this instance, was key to a fully telescoped process involving triflyl azide generation, diazo transfer and direct C–H insertion reaction of α-diazo-β-oxosulfone **63** in continuous flow.

A number of heterogeneous batch reactions were initially conducted to support development of the final continuous process, as shown in Table 5. These reactions investigated whether the IPB copper–bis(oxazoline) catalyst **9** would afford efficient C–H insertion of α-diazo-β-oxosulfone **63**, as observed upon homogeneous copper-catalyzed batch reactions,^13^ prior to attempting a telescoped diazo-transfer reaction and C–H insertion step in flow. To examine results of a homogeneous versus heterogeneous copper-catalyzed C–H insertion during this investigation, a homogeneous reaction was also undertaken for comparison using Cu(CH_3_CN)_4_PF_6_ (5 mol %) and the analogous (4*R*)-Ph bis(oxazoline) ligand **64** (6 mol %), in dichloromethane at reflux (Table 5, entry 5).

**Table 5:**
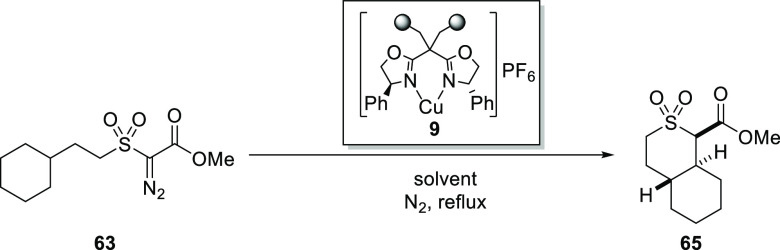

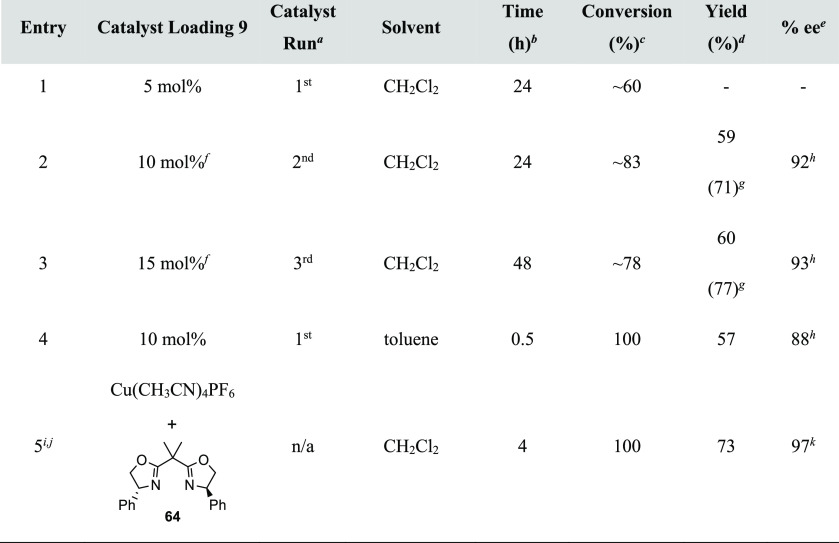
Heterogeneous Copper-Catalyzed C–H Insertion of α-Diazo-β-oxosulfone **63** in Batch

a Compound **9** was reused multiple times with addition of fresh catalyst (entries 1–3), collected by filtration once the crude reaction mixture was cooled to room temperature, agitated in fresh dichloromethane for 2 h, collected by filtration, and air-dried overnight.

b Reaction completion was determined by IR spectroscopic analysis upon disappearance of the diazo stretch for **63**.

c Conversion was calculated on the basis of comparison of the integrations for the C(1)*H* doublet of doublets at δ_H_ 3.75 (1H, *J* 4.7, 3.1 Hz) for **65** and the methyl singlet at δ_H_ 3.88 (3H) for **63**.

d Yields after column chromatography.

e The enantiopurity was measured by chiral-phase HPLC analysis (for full details, see the SI).

f Fresh **9** was used to make up the difference in polymer catalyst mol % from the previous run.

g Yield in parentheses was calculated on the basis of the percent conversion from the ^1^H NMR spectrum of the crude reaction material.

h The major enantiomer has a 1*R*,4a*R*,8a*S* configuration.

i Homogeneous batch reaction.

j Cu(CH_3_CN)_4_PF_6_ (5 mol %) and (4*R*)-Ph BOX ligand **64** (6 mol %) used.

k The major enantiomer has a 1*S*,4a*S*,8a*R* configuration.

Reactions using IPB copper catalyst **9** at 5 and 10 mol % (5 mol % fresh catalyst and 5 mol % of reused catalyst) loadings in dichloromethane at reflux did not achieve complete conversion of **63**; the heterogeneous reaction was considerably slower than the homogeneous reaction, which was complete after 4 h (Table 5, entry 1 cf. entry 5).

After reflux for 24 h at a 10 mol % loading of **9** (Table 5, entry 2), 83% conversion was observed in the ^1^H NMR spectrum of the crude reaction mixture which resulted in a yield of 59% for **65** with 92% ee after column chromatography. The decrease in enantioselectivity from 97% ee to 92% ee observed, upon immobilization of the ligand, when using the IPB copper catalyst **9** is relatively modest and is in line with that observed for aromatic addition.^14^ As expected, the major enantiomer of **65** isolated from the use of the IPB (4*S*)-Ph bis(oxazoline) copper catalyst **9** was the 1*R*,4a*R*,8a*S* enantiomer, the opposite enantiomer observed to that isolated from the homogeneous reaction when using the (4*R*)-Ph bis(oxazoline) ligand **64**. This result highlights that very similar ligand–substrate interactions occur with the heterogeneous IPB copper catalyst, compared to the homogeneous copper-catalyzed reaction.

In an attempt to achieve 100% conversion of the α-diazo-β-oxosulfone **63**, 15 mol % of **9** was used (5 mol % fresh catalyst and 10 mol % reused catalyst) with the reaction mixture heated under reflux for 48 h (Table 5, entry 3); however, only 78% conversion was achieved, with a 60% yield of thiopyran **65**, while retaining 93% ee in the insertion product. An interesting point to note is that the regio- and diastereoselectivity of the reaction appear unaffected by use of the IPB copper catalyst **9**; in common with similar homogeneously catalyzed reactions, **65** was found to be the major product.^13^

Although maintaining a high level of stereoselectivity, achieving reaction completion in dichloromethane under reflux was challenging with the IPB copper catalyst **9**. In order to facilitate the transition of the copper-catalyzed desymmetrization process of α-diazo-β-oxosulfone **63** to a continuous flow platform, the reaction would ideally reach completion in less than an hour to avoid requiring impractically low flow rates that would correspond to excessively long residence times. Accordingly, conducting the IPB copper-catalyzed C–H insertion of **63** in toluene, a higher boiling (bp 111 °C) and “greener” solvent compared to dichloromethane (bp 40 °C) was investigated with a view to reducing the reaction time.

A heterogeneous reaction of α-diazo-β-oxosulfone **63** was conducted using toluene under reflux with a fresh sample (10 mol %) of **9** in a manner similar to that employed for the other entries in Table 5, other than the change of solvent. The reaction was observed to occur rapidly (30 min) at the elevated temperature of 111 °C; however, some additional impurity signals were noticed in the ^1^H NMR spectrum of the crude product mixture, albeit with **65** still observed as the major product. Gratifyingly, **65** was isolated with a 57% yield and 88% ee after column chromatography. Both the yield and the enantioselectivity of **65** were reduced somewhat in comparison to those seen for the dichloromethane reactions using **9** and for the homogeneous reaction (Table 5, entries 1–5), however, with clear advantages in terms of reaction time and efficiency.

**Scheme 17:**
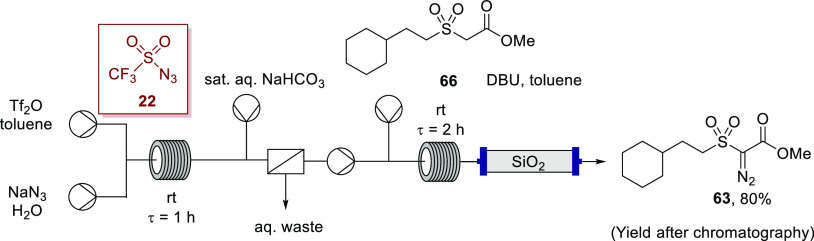
Synthesis of α-Diazoketone **63** with the Telescoped Flow Synthesis Using in Situ Generated Triflyl Azide (**22**)

On the basis of these encouraging preliminary results using the IPB copper catalyst **9** in batch, the desymmetrization reaction was transferred to the three-step telescoped flow process. Initially, focusing on the diazo-transfer step, the synthesis of **63** was undertaken in flow in toluene via Regitz-type diazo transfer following triflyl azide formation (Scheme 17), with 80% yield of α-diazoester **63** obtained after column chromatography, which was comparable to the batch reaction using the diazo-transfer agent *p*-ABSA. Thus, the triflyl azide flow methodology is also very effective for diazo transfer to α-sulfonyl esters.

**Scheme 18:**
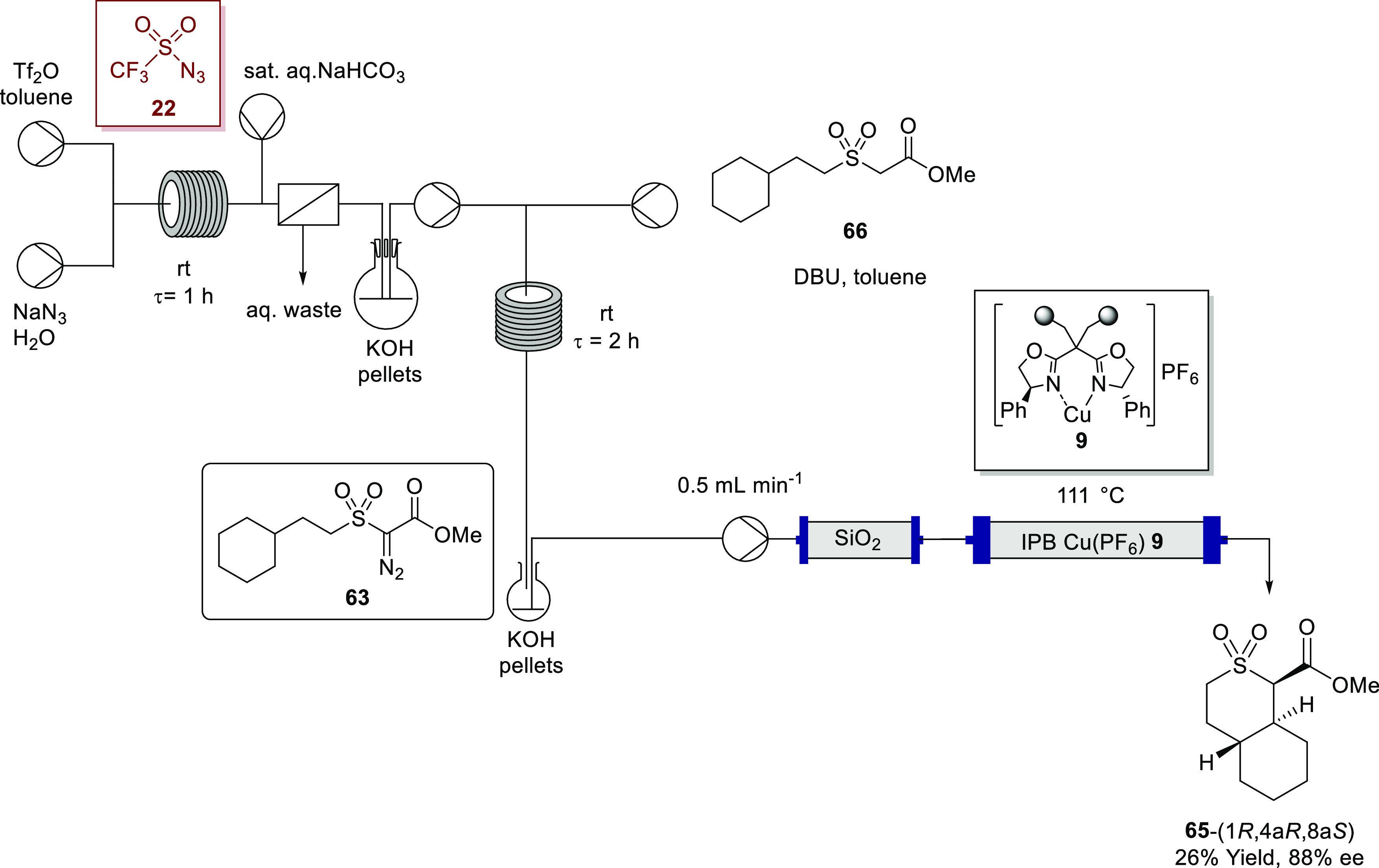
Telescoped Process for the Synthesis and C–H Insertion of α-Diazo-β-oxosulfone **63**

For the telescoped three-step continuous process, including C–H insertion, in situ prepared α-diazo-β-oxosulfone **63** solution was pumped, first, through a glass column reactor packed with silica gel to remove polar reagents and byproducts (principally, DBU and DBU salts), followed subsequently by a glass column reactor packed with the IPB copper catalyst **9** (10 mol %) that was heated to 111 °C, where the final C–H insertion step occurred (Scheme 18).

The telescoped process resulted in the desymmetrized product **65** being isolated in 26% yield and 88% ee after column chromatography. Critically, this outcome showed that it is possible to utilize the reaction stream containing the α-diazosulfone **63** directly in the copper-mediated C–H insertion without any deterioration in the enantiopurity of the desymmetrized product **65**, albeit with a reduced yield. Thus, the telescoping is feasible not only with copper-mediated aromatic addition but also with the much more demanding C–H insertion into an unactivated bond. Although a lower yield was observed, the enantioselectivity observed is comparable to that obtained when the heterogeneous batch reaction was conducted in toluene (Table 5, entry 4).

Thus, it appears the generation of triflyl azide (**22**) and telescoping this with the diazo transfer to form **63** proceeds efficiently; however, the outflow of the diazo transfer is a relatively complex solution containing a stoichiometric amount of DBU, triflyl sulfonamide and potentially other byproducts which could detrimentally impact on the IPB copper catalyst. Observation of enantioselective C–H insertion, albeit at a reduced efficiency compared to a typical homogeneous reaction,^13^ solely by passing the reaction mixture through silica gel, offers promise that this process can be further optimized in terms of yield, especially as the extent of the enantioselectivity was not detrimentally affected (88% ee). Notably, the synthesis of triflyl azide (**22**), the diazo transfer to **66**, and the copper-mediated C–H insertion can be conducted in a single telescoped process without detrimental impact on the stereochemical outcome and, in particular, the enantiopurity of the thiopyran dioxide **65**.

**Figure 5:**
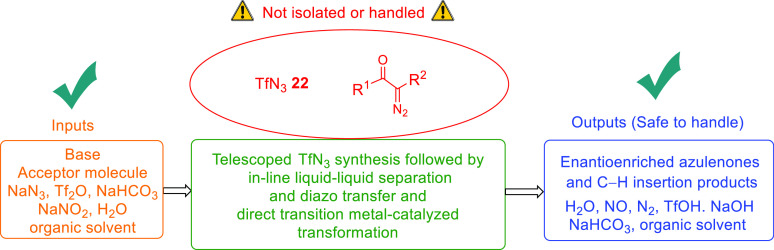
Summary of process inputs and outputs.

Previous literature reports generated or utilized TsN_3_ in solvents which had detrimental effects on the yields of downstream transition-metal-catalyzed processes, such as NMP^40^ or MeCN.^38^ In addition to the high level of reactivity of TfN_3_, one of the primary reasons for selecting TfN_3_ as diazo-transfer reagent was due to the ability to generate the compound in a solvent (toluene or dichloromethane in this work) which was amenable for use in downstream transition-metal-catalyzed processes without requiring a solvent swap. Ultimately, the potential hazard of residual triflyl azide (**22**) in the reaction outflow was mitigated by two features: (1) in the development of the diazo transfer telescoped with triflyl azide formation, it was established using IR spectroscopy that no residual triflyl azide (**22**) was present in the reaction outflow, for the reagent ratio levels employed; (2) reaction monitoring of each the reaction effluents by IR spectroscopy prior to concentration showed no evidence of the characteristic azide stretch for triflyl azide (**22**), or indeed sodium azide. As summarized in Figure 5 the synthetic power of triflyl azide and the α-diazocarbonyl compounds has been effectively exploited, in tandem with immobilized copper catalysts, in transforming achiral starting materials to highly enantioenriched products while obviating the need to isolate, handle, or store these compounds.

## Conclusion

While triflyl azide (**22**) is a very powerful reagent for diazo transfer, its use is limited by the hazards associated with its handling. Generation and use of triflyl azide in flow leads to efficient synthesis of a range of α-diazocarbonyl compounds, including α-diazoketones **8**, **11**–**13**, an α-diazolactam **29**, α-cyano-α-diazoacetamides **55**–**62**, an α-diazo-β-ketonitrile **38**, and an α-diazosulfonyl ester **63**, via both Regitz-type diazo transfer and deacylative/debenzoylative diazo-transfer processes in excellent yields (70–96%) and offers versatility in the solvent employed, in addition to addressing the hazards associated with handling of this highly reactive sulfonyl azide. Notably, triflyl azide (**22**) led to successful diazo transfer to form α-diazoketones **8** and **11**–**13** with substantially higher yields than achieved with tosyl azide in flow. Furthermore, using this continuous flow protocol the diazo substrates are generated in solvents that are compatible with downstream transition-metal-catalyzed processes, such as toluene and dichloromethane. Telescoping the generation of the triflyl azide (**22**) and diazo-transfer process with enantioselective copper-mediated intramolecular aromatic addition and C–H insertion processes demonstrates that the reaction stream of the α-diazocarbonyl compound can be obtained in sufficient purity to pass directly into the immobilized copper bis(oxazoline) catalyst **9** without detrimentally impacting on the enantioselectivity observed. Thus, azulenones **31** and **32** (as its PTAD adduct **10**) were obtained in 60% ee and 82% ee, in 50% and 30% yield respectively, with little or no decrease in enantioselectivity observed when the Buchner addition was telescoped with the diazo-transfer protocol. Furthermore, the desymmetrisation via copper-mediated C–H insertion to form the thiopyran *S*,*S*-dioxide proved successful in toluene, leading to the desymmetrized product in 88% ee, comparable with the enantioselectivity observed using the immobilized copper catalyst in a batch reaction.

## Experimental Section

### General Procedures

Solvents were distilled prior to use as follows: dichloromethane was distilled from calcium hydride when used for transformations of α-diazoketones, ethyl acetate was distilled from potassium carbonate, tetrahydrofuran was distilled from sodium benzophenone ketyl in a nitrogen atmosphere and hexane was distilled prior to use. All commercial reagents were used without further purification unless otherwise stated. Amines **39**–**46**,^70^ sulfone **63**,^13^ and pyrrolidinone **28**,^82^ were prepared according to literature methods. Insoluble polymer bound (IBP) catalyst **9** was prepared from copper(I) hexafluorophosphate and 2,2′-(1,3-bis(4-vinylphenyl)propylidene)bis((4*S*)-4-phenyl-4,5-dihydro-2-oxazole) polymer as previously reported.^14^

^1^H (300 MHz) and ^13^C (75.5 MHz) NMR spectra were recorded on a Bruker Avance 300 NMR spectrometer. ^1^H (400 MHz) and ^13^C (100.6 MHz) NMR spectra were recorded on a Bruker 400 MHz NMR spectrometer. All spectra were recorded at 300 K in deuterated chloroform (CDCl_3_) unless otherwise stated, using tetramethylsilane (TMS). Chemical shifts (δ_H_ and δ_C_) are reported in parts per million (ppm) relative to TMS and coupling constants are expressed in hertz (Hz). ^19^F NMR spectra showed chemical shifts (δ_F_) measured relative to hexafluorobenzene, which shows a single resonance at −163.0 ppm. Splitting patterns in ^1^H spectra are designated as s (singlet), bs (broad singlet), d (doublet), bd (broad doublet), t (triplet), bt (broad triplet), q (quartet), qu (quintet), dd (doublet of doublets), dt (doublet of triplets), dq (doublet of quartets), ddd (doublet of doublets of doublets), td (triplet of doublets), ddt (doublet of doublets of triplets), AB (AB system), or (m) multiplet. ^13^C NMR spectra were calibrated using the solvent signal, i.e. CDCl_3_: δ_C_ 77.0 ppm, and multiplicities were assigned with the aid of DEPT experiments. Assignment of ^1^H signals was aided using 2D NMR including ^1^H–^1^H COSY, HSQC and HMBC.

Infrared spectra were measured using a Perkin–Elmer FTIR UATR2 spectrometer.

Organic phases were dried using magnesium sulfate or potassium hydroxide pellets. Flash column chromatography was carried out using Kieselgel silica gel 60, 0.040–0.063 mm (Merck). Thin-layer chromatography (TLC) was carried out on precoated silica gel plates (Merck 60 PF254). Visualization was achieved by UV (254 nm) light absorption.

Elemental analysis was carried out by Microanalysis Laboratory, National University of Ireland, Cork, using Exeter Analytical CE440 elemental analysers. Low-resolution mass spectra (LRMS) were recorded on a Waters Quattro Micro triple quadrupole instrument in electrospray ionization (ESI) mode using 50% acetonitrile–water containing 0.1% formic acid as eluent. High-resolution mass spectra (HRMS) were recorded on a Waters LCT Premier TofLC–MS instrument in electrospray ionization (ESI) mode using 50% acetonitrile–water containing 0.1% formic acid as eluent or on an Agilent 6530B Accurate Mass Q-TOF LC/MS instrument in electrospray ionization (ESI) mode using 50% acetonitrile–water containing 0.1% formic acid as eluent. High resolution (precise) mass spectra (HRMS) were also recorded on a Waters Vion IMS instrument (SAA055 K) with Waters Acquity I-class UPLC in electrospray ionization (ESI) mode using 50% acetonitrile–water containing 0.1% formic acid as eluent and Leucine Enkephalin as reference solution. Samples were prepared for either LRMS or HRMS by employing acetonitrile as solvent and are accurate to within 5 ppm.

Melting points were obtained using a Unimelt Thomas-Hoover Capillary melting point apparatus and are uncorrected. Enantiopurity of chiral compounds was determined by chiral stationary phase high-performance liquid chromatography (HPLC) performed on a Chiralcel OD-H or Lux Amylose-1 column. HPLC analysis was performed on a Waters alliance 2690 separations module. All chiral columns were purchased from Daicel Chemical Industries Limited or Phenomenex. Optical rotations were measured at 589 nm in a 10 cm cell on a PerkinElmer 141 polarimeter or on a Rudolph Autopol V Plus polarimeter; concentrations (*c*) are expressed in g/100 mL, and the specific rotation of a compound and is expressed in units of 10^–1^ deg cm^2^ g^–1^.

All continuous processes were performed using a flow chemistry system consisting of four HPLC pumps and up to four temperature-controlled tubular reactors (a glass reactor manifold containing a temperature controlled glass column) and a flow chemistry system consisting of three peristaltic pumps. To prepare the reactor for operation pumps were purged with the solvent to be used in the reaction prior to use. All reaction tubing, coils, inlets and connections were also purged thoroughly in a similar manner. All pumps were primed using appropriate solvents and pump backwash reservoirs were filled. The solvent that was to be used was flushed through all injectors and reactors. Pumps were run at reaction flow rates to check for stability, in both reagent and solvent lines, before committing reagents. Reactors that were to be used were then heated to the desired temperatures, using the flow chemistry platform, to check system pressurization. General specifications of flow systems used: *Material of tubing*: PFA; *Diameter of tubing*: 1 mm; *Working flow rates*: 0.05 mL/min −9.99 mL/min; *Tubular reactor working volume*: 10 mL; *Temperature range*: −70 to +250 °C.

### Preparation of 1,3-Diketones

#### General Procedure for Preparation of Acyl Benzotriazoles^98^

Thionyl chloride (1.15 equiv) was added in one portion to a stirring solution of 1(*H*)-benzotriazole (4.25 equiv) in dichloromethane. The resulting yellow solution was stirred at room temperature for 30 min, after which carboxylic acid (1 equiv) was added in one portion. A white suspension formed following the addition, which was stirred at room temperature for 2 h. The suspension was filtered by gravity filtration, and the white solid collected was washed with dichloromethane (2 × 30 mL). The combined organic washings and filtrate were collected, dried, and concentrated under reduced pressure to afford the crude acylbenzotriazole.

##### 1′-(1*H*-Benzo[*d*][1,2,3]triazol-1-yl)-3′-methyl-3′-phenylbutan-1′-one^98^

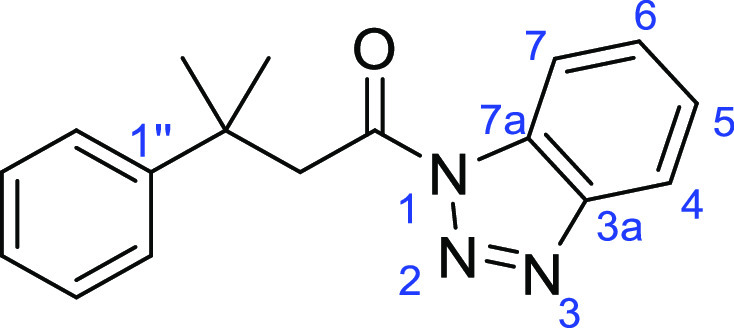



This compound was prepared according to the general procedure using thionyl chloride (1.0 mL, 13.0 mmol), 1(*H*)-benzotriazole (5.80 g, 48.7 mmol), 3-methyl-3-phenylbutanoic acid (2.03 g, 11.4 mmol) and dichloromethane (75 mL). The crude product was purified by flash chromatography on silica gel with hexane/ethyl acetate (80:20) as eluent which afforded *acylbenzotriazole* as a colorless oil (2.39 g, 75%). ν_max_/cm^–1^ (ATR): 2966, 1739, 1596, 1484, 1450, 1365. ^1^H NMR (CDCl_3_, 400 MHz): δ 8.21 (d, 1H, *J* = 8.3 Hz, Ar*H*), 8.08 (d, 1H, *J* = 8.2 Hz, Ar*H*), 7.63–7.55 (m, 1H, Ar*H*), 7.51–7.41 (m, 3H, Ar*H*), 7.33–7.26 (m, 2H, Ar*H*), 7.21–7.13 (m, 1H, Ar*H*), 3.82 [s, 2H, C(2′)C*H*_*2*_], 1.60 [s, 6H, C(3′)(C*H*_*3*_)_2_]. ^13^C{^1^H} NMR (CDCl_3_, 100.6 MHz): δ 170.4 (C), 147.6 (C), 146.2 (C), 131.0 (C), 130.2 (CH), 128.3 (CH), 126.2 (CH), 126.1 (CH), 125.5 (CH), 120.1 (CH), 114.6 (CH), 47.7 (CH_2_), 38.0 (C), 29.2 (CH_3_). HRMS (ESI) *m*/*z*: [M + H]^+^ Calcd for C_17_H_18_N_3_O, 280.1450; Found 280.1450.

##### 1′-(1*H*-Benzo[*d*][1,2,3]triazol-1-yl)-3′-methyl-3′-(3′′,4′′,5′′-trimethylphenyl)-butan-1′-one

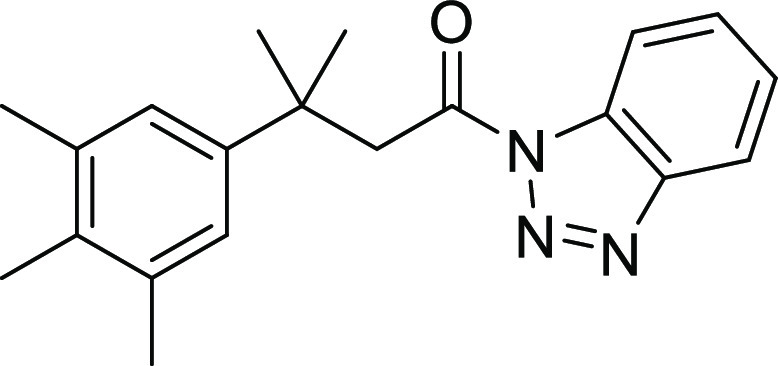



This compound was prepared according to the general procedure^98^ using thionyl chloride (2.30 mL, 30.0 mmol), 1(*H*)-benzotriazole (14.30 g, 120.0 mmol), 3-methyl-3-(3′,4′,5′-trimethylphenyl)butanoic acid (6.50 g, 30.0 mmol) and dichloromethane (100 mL). The crude *acyl benzotriazole* was isolated as a brown oil [8.31 g, 86%—due to the high viscosity of the sample (and consequent difficulty with solvent removal in vacuo), it contained residual dichloromethane (δ_H_5.30 ppm)—estimated corrected yield 64%] and carried forward without chromatographic purification. ^1^H NMR (CDCl_3_, 400 MHz): δ 8.23 (d, 1H, *J* 8.3, Ar*H*), 8.08 (d, 1H, *J* = 8.3 Hz, Ar*H*), 7.63–7.54 (m, 2H, Ar*H*), 7.51–7.41 (m, 2H, Ar*H*), 7.07 [s, 2H, C(2′′)*H* and C(6′′)*H*], 3.78 [s, 2H, C(2′)C*H*_*2*_], 2.23 [s, 6H, C(3′′)C*H*_3_ and C(5′′)C*H*_3_], 2.08 [s, 3H, C(4′′)C*H*_3_], 1.56 [s, 6H, C(3′)(C*H*_*3*_)_2_].

##### 1′-(1*H*-Benzo[*d*][1,2,3]triazol-1-yl)-3′-methyl-3′-(4′′-chlorophenyl)butan-1′-one

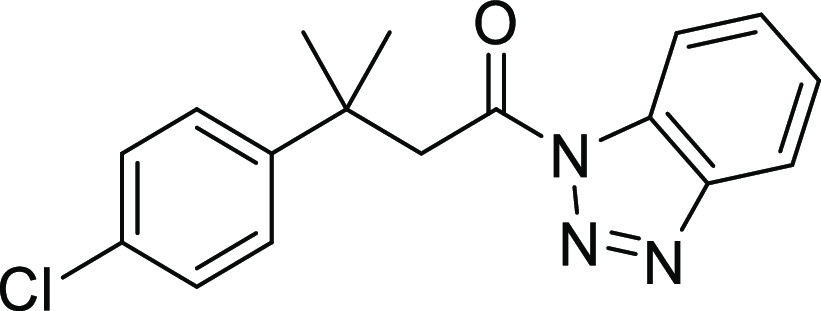



This compound was prepared according to the general procedure^98^ using thionyl chloride (1.03 mL, 13.8 mmol), 1(*H*)-benzotriazole (6.59 g, 55.36 mmol), 3-methyl-3-(4′-chlorophenyl)butanoic acid (2.94 g, 13.84 mmol), and dichloromethane (75 mL). The crude *acyl benzotriazole* was isolated as a colorless oil (3.09 g, 71%) and carried forward without chromatographic purification. ^1^H NMR (CDCl_3_, 400 MHz): δ 8.20 (d, 1H, *J* = 8.3 Hz, Ar*H*), 8.10 (d, 1H, *J* = 8.3 Hz, Ar*H*), 7.65–7.56 (m, 1H, Ar*H*), 7.53–7.45 (m, 1H, Ar*H*), 7.44–7.36 (m, 2H, Ar*H*), 7.30–7.22 (m, 2H, Ar*H*), 3.81 [s, 2H, C(2′)C*H*_*2*_], 1.58 [s, 6H, C(3′)(C*H*_*3*_)_2_]. ^13^C{^1^H} NMR (CDCl_3_, 100.6 MHz): δ 170.1 (C), 146.2 (C), 146.1 (C), 132.0 (C), 131.0 (C), 130.4 (CH), 128.4 (CH), 127.0 (CH), 126.2 (CH), 120.1 (CH), 114.5 (CH), 47.6 (CH_2_), 37.6 (C), 29.3 (CH_3_).

##### 1′-(1*H*-Benzo[*d*][1,2,3]triazol-1-yl)-3′-methyl-3′-(4′′-fluorophenyl)butan-1′-one

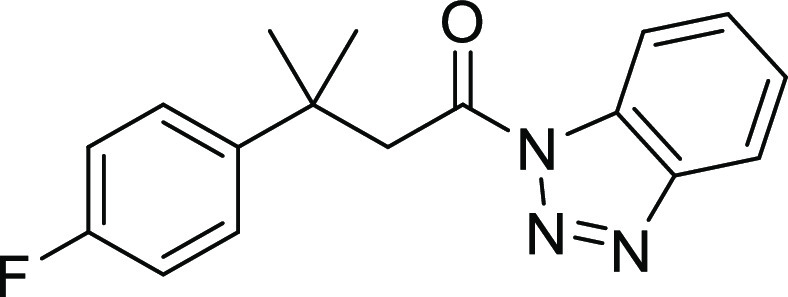



This compound was prepared according to the general procedure^98^ using thionyl chloride (0.66 mL, 5.50 mmol), 1(*H*)-benzotriazole (4.00 g, 34.0 mmol), 3-methyl-3-(4′-fluorophenyl)butanoic acid (1.66 g, 8.50 mmol), and dichloromethane (50 mL). The crude *acyl benzotriazole* was isolated as a yellow oil [2.30 g, 91%—due to the high viscosity of the sample (and consequent difficulty with solvent removal in vacuo), it contained residual dichloromethane (δ_H_5.30 ppm)—estimated corrected yield 72%] and carried forward without chromatographic purification. ^1^H NMR (CDCl_3_, 400 MHz): δ 8.21 (d, 1H, *J* = 8.3 Hz, Ar*H*), 8.09 (d, 1H, *J* = 8.2 Hz, Ar*H*), 7.66–7.56 (m, 1H, Ar*H*), 7.53–7.34 (m, 3H, Ar*H*), 6.98 (t, 2H, *J* = 8.7 Hz, Ar*H*), 3.80 [s, 2H, C(2′)C*H*_*2*_], 1.59 [s, 6H, C(3′)(C*H*_*3*_)_2_].

##### 2,5-Dimethyl-1,5-diphenylhexane-1,3-dione (**19**)^66^

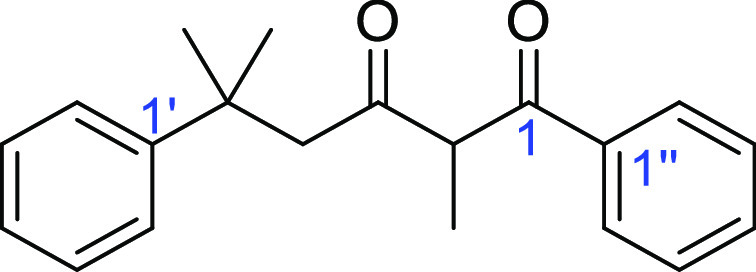



Lithium bis(trimethylsilyl)amide (LiHMDS) (1.0 M in THF, 17.7 mL, 17.7 mmol) was diluted in freshly distilled tetrahydrofuran (60 mL) and cooled to −80 °C (Bath temp). A tetrahydrofuran solution (10 mL) of propiophenone (2.2 mL, 16.8 mmol) was added slowly over 15 min. The reaction mixture was stirred at −80 °C for 1 h after which a tetrahydrofuran solution (15 mL) of 1′-(1*H*-benzo[*d*][1,2,3]triazol-1-yl)-3′-methyl-3′-phenylbutan-1′-one (4.68 g, 16.8 mmol) was added in one portion. The reaction mixture was allowed to warm slowly to room temperature overnight. The reaction mixture was diluted with aqueous hydrochloric acid (3.2 M, 30 mL) and stirred for 10 min. The layers were separated and the aqueous layer was extracted with diethyl ether (2 × 25 mL). The organic layers were combined, dried, and concentrated under reduced pressure. The crude product was purified by flash chromatography on silica gel with hexane/ethyl acetate (95:5) as eluent which afforded *diketone***19** as a colorless oil (2.75 g, 56%). ν_max_/cm^–1^ (ATR): 2929, 1709, 1677. ^1^H NMR (CDCl_3_, 400 MHz): δ 7.77–7.70 (m, 2H, Ar*H*), 7.60–7.52 (m, 1H, Ar*H*), 7.46–7.37 (m, 2H, Ar*H*), 7.32–7.22 (m, 4H, Ar*H*), 7.20–7.12 (m, 1H, Ar*H*), 4.03 [q, 1H, *J* = 7.0 Hz, C(2)*H*], 2.79 [s, 2H, C(4)*H*_2_], 1.41, 1.34 [2 × s, 2 × 3H, C(5)(C*H*_3_)_2_], 1.24 [d, 3H, *J* = 7.0 Hz, C(2)HC*H*_3_]. ^13^C{^1^H} NMR (100.6 MHz): δ 205.3 (C), 197.8 (C), 148.1 (C), 135.9 (C), 133.5 (CH), 128.7 (CH), 128.6 (CH), 128.3 (CH), 126.0 (CH), 125.4 (CH), 56.8 (CH), 54.2 (CH_2_), 37.3 (C), 29.8 (CH_3_), 28.1 (CH_3_), 13.4 (CH_3_). HRMS (ESI) *m*/*z*: [M + H]^+^ Calcd for C_20_H_23_O_2_ 295.1698; Found 295.1710.

##### 2,5-Dimethyl-1-phenyl-5-(3′,4′,5′-trimethylphenyl)hexane-1,3-dione (**18**)

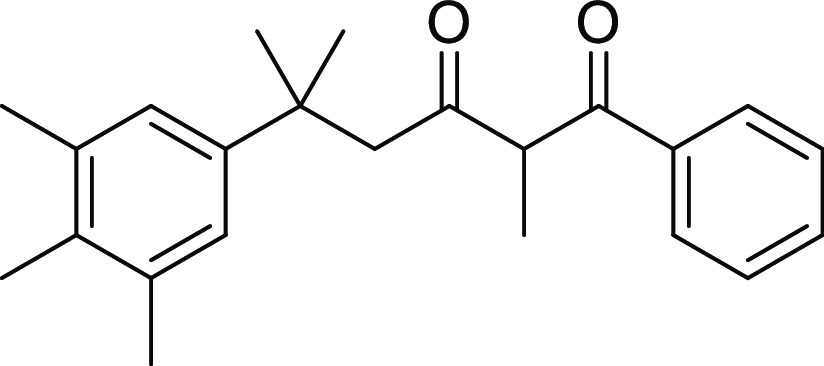



This compound was prepared according to the procedure described for **19** from lithium bis(trimethylsilyl)amide (LiHMDS) (1.0 M in THF, 25.0 mL, 25.0 mmol), propiophenone (3.10 mL, 22.8 mmol) and 1′-(1*H*-benzo[*d*][1,2,3]triazol-1-yl)-3′-methyl-3′-(3′′,4′′,5′′-trimethylphenyl)butan-1′-one (7.33 g, 22.8 mmol) in THF (100 mL). The crude product was purified by flash chromatography on silica gel with hexane/ethyl acetate (97:3) as eluent which afforded *diketone***18** as a colorless oil (4.91 g, 64%); ν_max_/cm^–1^ (ATR): 2965, 1715, 1675. ^1^H NMR (CDCl_3_, 400 MHz): δ 7.70–7.65 [m, 2H, C(2′′)*H* and C(6′′)*H*], 7.58–7.53 [m, 1H, C(4′′)*H*], 7.42–7.37 [m, 2H, C(3′′)*H* and C(5′′)*H*], 6.90 [s, 2H, C(2′)*H* and C(6′)*H*], 3.98 [q, 1H, *J* = 7.1 Hz, C(2)*H*], 2.77 [AB, 2H, *J*_AB_ = 22.5 Hz, H_A_ δ = 2.84, H_B_ δ = 2.71, C(4)*H*_2_], 2.18 [s, 6H, C(3′)C*H*_3_ and C(5′)C*H*_3_], 2.08 [s, 3H, C(4′)C*H*_3_], 1.37, 1.33 [2 × s, 2 × 3H, C(5)(C*H*_3_)_2_], 1.23 [d, 3H, *J* = 7.0 Hz, C(2)HC*H*_3_]. ^13^C{^1^H} NMR (CDCl_3_, 100.6 MHz): δ 205.8 (C), 198.0 (C), 144.8 (C), 136.3 (C), 135.7 (C), 133.4 (CH), 132.7 (C), 128.6 (CH), 124.7 (CH), 56.3 (CH), 54.6 (CH_2_), 36.8 (C), 30.5 (CH_3_), 27.8 (CH_3_), 20.8 (CH_3_), 15.0 (CH_3_), 13.4 (CH_3_). HRMS (ESI) *m*/*z*: [M + H]^+^ Calcd for C_23_H_29_O_2_ 337.2162; Found 337.2160.

##### 2,5-Dimethyl-1-phenyl-5-(4′-chlorophenyl)hexane-1,3-dione (**20**)

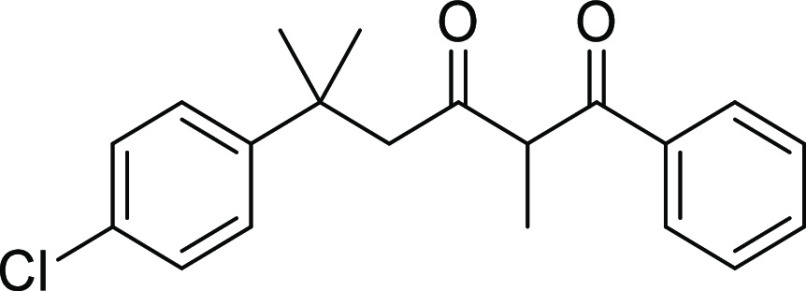



This compound was prepared according to the procedure described for **19** from lithium bis(trimethylsilyl)amide (LiHMDS) (1.0 M in THF, 25.0 mL, 25.0 mmol), propiophenone (3.05 mL, 23.0 mmol), and 1′-(1*H*-benzo[*d*][1,2,3]triazol-1-yl)-3′-methyl-3′-(4′′-chlorophenyl)butan-1′-one (7.22 g, 23.0 mmol) in THF (100 mL). The crude product was purified by flash chromatography on silica gel with hexane/ethyl acetate (97:3) as eluent which afforded *diketone***20** as a colorless oil (6.43 g, 85%). ν_max_/cm^–1^ (ATR): 2966, 1715, 1674. ^1^H NMR (CDCl_3_, 400 MHz): δ 7.82–7.73 [m, 2H, C(2′′)*H* and C(6′′)*H*], 7.64–7.55 [m, 1H, C(4′′)*H*], 7.49–7.39 [m, 2H, C(3′′)*H* and C(5′′)*H*], 7.21–7.09 [m, 4H, C(2′)*H*, C(3′)*H*, C(5′)*H* and C(6′)*H*], 4.13 [q, 1H, *J* = 7.0 Hz, C(2)*H*], 2.79 [s, 2H, C(4)*H*_2_], 1.38, 1.33 [2 × s, 2 × 3H, C(5)(C*H*_3_)_2_], 1.30 [d, 3H, *J* = 7.0 Hz, C(2)HC*H*_3_]. ^13^C{^1^H} NMR (CDCl_3_, 100.6 MHz): δ 205.0 (C) 197.5 (C), 146.6 (C), 135.8 (C), 133.7 (CH), 131.6 (C), 128.8 (CH), 128.6 (CH), 128.3 (CH), 126.9 (CH), 57.1 (CH), 53.4 (CH_2_), 36.8 (C), 29.8 (CH_3_), 28.4 (CH_3_), 13.4 (CH_3_). HRMS (ESI) *m*/*z*: [M + H]^+^ Calcd for C_20_H_22_O_2_^35^Cl 329.1303; Found 329.1294.

##### 2,5-Dimethyl-1-phenyl-5-(4′-fluorophenyl)hexane-1,3-dione (**21**)

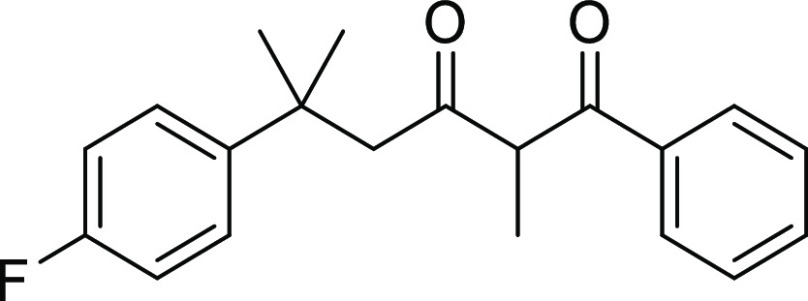



This compound was prepared according to the procedure described for **19** from lithium bis(trimethylsilyl)amide (LiHMDS) (1.0 M in THF, 8.2 mL, 8.2 mmol), propiophenone (1.02 mL, 7.70 mmol) and 1′-(1*H*-benzo[*d*][1,2,3]triazol-1-yl)-3′-methyl-3′-(4′′-fluorophenyl)butan-1′-one (2.30 g, 7.70 mmol) in THF (75 mL). The crude product was purified by flash chromatography on silica gel with hexane/ethyl acetate (97:3) as eluent which afforded *diketone***21** as a *c*olourless oil (1.47 g, 62%). ν_max_/cm^–1^ (ATR): 2967, 1715, 1676. ^1^H NMR (CDCl_3_, 400 MHz): δ 7.80–7.74 [m, 2H, C(2′′)*H* and C(6′′)*H*], 7.61–7.54 [m, 1H, C(4′′)*H*], 7.47–7.39 [m, 2H, C(3′′)*H* and C(5′′)*H*], 7.24–7.16 [m, 2H, C(2′)*H* and C(6′)*H*], 6.93–6.85 [m, 2H, C(3′)*H* and C(5′)*H*], 4.11 [q, 1H, *J* = 7.0 Hz, C(2)*H*], 2.78 [s, 2H, C(4)*H*_2_], 1.39, 1.34 [2 × s, 2 × 3H, C(5)(C*H*_3_)_2_], 1.28 [d, 3H, *J* = 7.0 Hz, C(2)HC*H*_3_]. ^13^C{^1^H} NMR (CDCl_3_, 100.6 MHz): δ 205.1 (C), 197.6 (C), 161.0 (C, d, ^1^*J*_CF_ = 244.4 Hz), 146.7 (C, d, ^4^*J*_CF_ = 3.2 Hz), 135.8 (C), 133.6 (CH), 128.8 (CH), 128.6 (CH), 127.0 (CH, d, ^3^*J*_CF_ = 7.7 Hz), 114.9 (CH, d, ^2^*J*_CF_ = 20.9 Hz), 57.1 (CH), 53.7 (CH_2_), 36.8 (C), 30.0 (CH_3_), 28.5 (CH_3_), 13.4 (CH_3_). ^19^F{^1^H} NMR (CDCl_3_, 376.5 MHz) δ −117.7; HRMS (ESI) *m*/*z*: [M + H]^+^ Calcd for C_20_H_22_O_2_F 313.1598; Found 313.1592.

##### 2-Ethyl-5-methyl-1,5-diphenylhexane-1,3-dione (**24**)

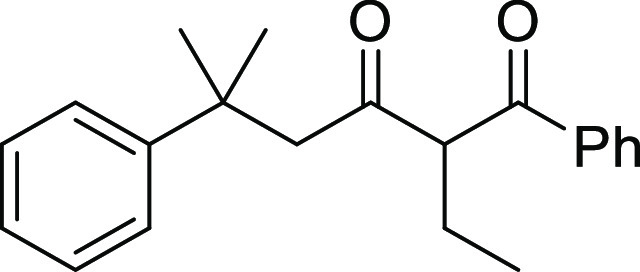



This compound was prepared according to the procedure described for **19** from lithium bis(trimethylsilyl)amide (LiHMDS) (1.0 M in THF, 15.0 mL, 15.0 mmol), butyrophenone (1.93 g, 13.0 mmol), and 1′-(1*H*-benzo[*d*][1,2,3]triazol-1-yl)-3′-methyl-3′-phenylbutan-1′-one (3.63 g, 13.0 mmol) in THF (100 mL). The crude product was purified by flash chromatography on silica gel with hexane/ethyl acetate (97:3) as eluent which afforded *diketone***24** as a colorless oil (3.41 g, 85%). ν_max_/cm^–1^ (ATR): 2965, 1716, 1673. ^1^H NMR (CDCl_3_, 400 MHz): δ 7.81–7.74 (m, 2H, Ar*H*), 7.60–7.53 (m, 1H, Ar*H*), 7.48–7.39 (m, 2H, Ar*H*), 7.30–7.20 (m, 4H, Ar*H*), 7.19–7.11 (m, 1H, Ar*H*), 3.94 [t, 1H, *J* = 6.8 Hz, C(2)*H*], 2.76 [AB, 2H, *J*_AB_ = 14.0 Hz, H_A_ δ = 2.79, H_B_ δ = 2.74, C(4)*H*_2_], 1.83 (qd, 2H, *J* = 7.3, 7.2 Hz, C*H*_2_CH_3_) 1.40, 1.35 [2 × s, 2 × 3H, C(5)(C*H*_3_)_2_], 0.77 (t, 3H, *J* = 7.4 Hz, CH_2_C*H*_3_). ^13^C{^1^H} NMR (CDCl_3_, 100.6 MHz): δ 204.5 (C), 197.0 (C), 148.0 (C), 136.5 (C), 133.5 (CH), 128.74 (CH), 128.67 (CH), 128.3 (CH), 125.9 (CH), 125.5 (CH), 65.0 (CH), 54.0 (CH_2_), 37.2 (C), 29.9 (CH_3_), 28.2 (CH_3_), 22.1 (CH_2_), 12.3 (CH_3_). HRMS (ESI) *m*/*z*: [M + H]^+^ Calcd for C_21_H_25_O_2_ 309.1849; Found 309.1850.

##### 2-(Cyclohexylmethyl)-5-methyl-1,5-diphenylhexane-1,3-dione (**25**)

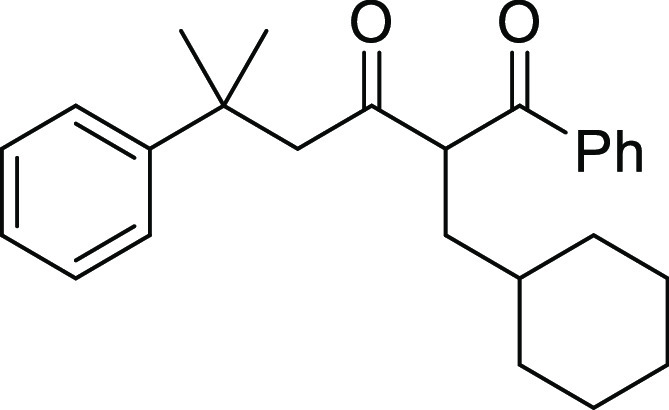



This compound was prepared according to the procedure described for 19 from lithium bis(trimethylsilyl)amide (LiHMDS) (1.0 M in THF, 10.0 mL, 10.0 mmol), 3-cyclohexyl-1-phenylpropan-1-one (1.73 g, 8.0 mmol), and 1′-(1*H*-benzo[*d*][1,2,3]triazol-1-yl)-3′-methyl-3′-phenylbutan-1′-one (2.23 g, 8.0 mmol) in THF (75 mL). The crude product was purified by flash chromatography on silica gel with hexane/ethyl acetate (95:5) as eluent which afforded *diketone***25** as a colorless oil (1.77 g, 59%). ν_max_/cm^–1^ (ATR): 2921, 2850, 1716, 1673. ^1^H NMR (CDCl_3_, 400 MHz): δ 7.82–7.74 [m, 2H, Ar*H*], 7.61–7.54 [m, 1H, Ar*H*], 7.48–7.39 (m, 2H, Ar*H*), 7.30–7.21 (m, 4H, Ar*H*), 7.19–7.12 (m, 1H, Ar*H*), 4.18 [t, 1H, *J* = 6.8 Hz, C(2)*H*], 2.77 [AB, 2H, *J*_AB_ = 14.2 Hz, H_A_ δ = 2.80, H_B_ δ = 2.75, C(4)*H*_2_], 1.76–1.44 (m, 7H), 1.40, 1.34 [2 × s, 2 × 3H, C(5)(C*H*_3_)_2_], 1.20–0.93 [m, 4H], 0.86–0.67 [m, 2H]. ^13^C{^1^H} NMR (CDCl_3_, 100.6 MHz): δ 204.5 (C), 197.0 (C), 148.0 (C), 136.3 (C), 133.5 (CH), 128.8 (CH), 128.7 (CH), 128.3 (CH), 125.9 (CH), 125.5 (CH), 61.1 (CH), 53.9 (CH_2_), 37.2 (C), 35.92 (CH_2_), 35.89 (CH), 33.4 (CH_2_), 33.0 (CH_2_), 30.0 (CH_3_), 28.3 (CH_3_), 26.3 (CH_2_), 26.09 (CH_2_), 26.07 (CH_2_). HRMS (ESI) *m*/*z*: [M + H]^+^ Calcd for C_26_H_33_O_2_ 377.2475; Found 377.2468.

##### 3-Hydroxy-5-methyl-1,5-diphenylhex-2-en-1-one (**35**)

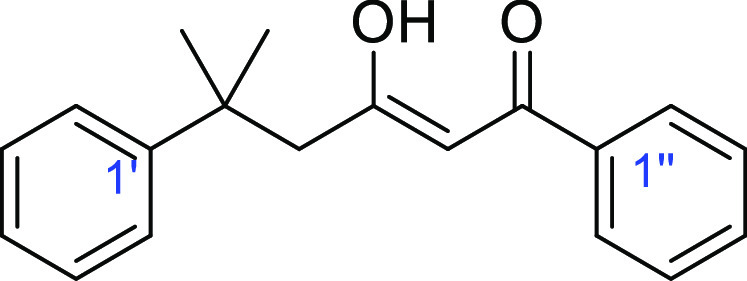



This compound was prepared according to the procedure described for **19** from lithium bis(trimethylsilyl)amide (LiHMDS) (1.0 M in THF, 10.0 mL, 10.0 mmol), acetophenone (1.08 g, 9.0 mmol), and 1′-(1*H*-benzo[*d*][1,2,3]triazol-1-yl)-3′-methyl-3′-phenylbutan-1′-one (2.51 g, 9.0 mmol) in THF (75 mL). The crude product was purified by flash chromatography on silica gel with hexane/ethyl acetate (99:1) as eluent which afforded *hydroxyenone***35** as a colorless oil (1.08 g, 43%). ν_max_/cm^–1^ (ATR): 2967, 1601. ^1^H NMR (CDCl_3_, 400 MHz): 16.12 (br s, 1H, O*H*), 7.72–7.60 (m, 2H, Ar*H*), 7.57–7.17 (m, 8H, Ar*H*), 5.66 [s, 1H, C(2)*H*], 2.69 [s, 2H, C(4)*H*_2_], 1.49 [s, 6H, C(5)(C*H*_3_)_2_]. HRMS (ESI) *m*/*z*: [M + H]^+^ Calcd for C_19_H_21_O_2_ 281.1536; Found 281.1530.

##### 5-Methyl-5-(4′-chlorophenyl)-3-oxohexanenitrile (**37**)

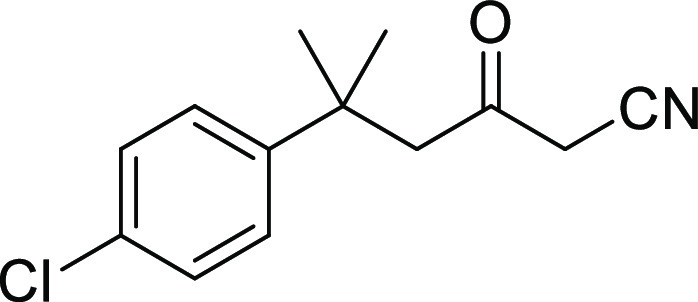



This compound was prepared according to a procedure for synthesis of β-ketonitriles.^99^ A 250 mL round-bottom flask was charged with acetonitrile (1.2 mL, 22.0 mmol) and freshly distilled THF (30 mL). The flask was cooled to 0 °C and potassium *tert*-pentoxide (25% w/v in toluene, 1.7 M, 13.0 mL, 22.0 mmol) was added slowly to the solution. While still at 0 °C, the flask was then charged with methyl 3-methyl-3-(4′-chlorophenyl)butanoate (4.73 g, 20.8 mmol) in THF (30 mL). The cooling bath was removed, and the flask was warmed to room temperature and after 2 h the solution was diluted with aqueous sat. ammonium chloride (40 mL). The organic layer was separated and the aqueous layer was extracted with diethyl ether. The combined organic layers were dried and concentrated under reduced pressure. The crude product was purified by flash chromatography on silica gel with hexane/ethyl acetate (90:10) as eluent which afforded *ketonitrile***37** as a colorless oil (1.86 g, 38%). ν_max_/cm^–1^ (ATR): 2967, 2259, 1731. ^1^H NMR (CDCl_3_, 400 MHz): δ 7.33–7.24 (m, 4H, Ar*H*), 3.06 [s, 2H, C(2)*H*_2_CN], 2.88 [s, 2H, C(4)*H*_2_], 1.43 [s, 6H, C(5)(C*H*_3_)_2_]. ^13^C{^1^H} NMR (CDCl_3_, 100.6 MHz): δ 195.9 (C), 145.5 (C), 132.3 (C), 128.7 (CH), 126.9 (CH), 113.6 (C), 55.0 (CH_2_), 37.3 (C), 33.4 (CH_2_), 28.8 (CH_3_). HRMS (ESI) *m*/*z*: [M + H]^+^ Calcd for C_13_H_15_NO^35^Cl 236.0931; Found 236.0948.

### Preparation of α-Cyanoacetamides

#### General Procedure for Preparation of α-Cyanoacetamides^70^

A round-bottom flask was charged with amine (**39**–**46**) (1.0 equiv), 2-cyanoacetic acid (1.02 equiv), and dichloromethane (60 mL) at 0 °C. The flask was then charged with a solution of *N,N′*-dicyclohexylcarbodiimide (1.02 equiv) and 4-dimethylaminopyridine (0.01 equiv). The reaction mixture was stirred for 1 h at room temperature. During this period, a white precipitate formed which was subsequently removed by suction filtration. The solvent was removed from the filtrate under reduced pressure. The product was recrystallized from ethanol.

##### *N*,*N*-Dibenzyl-2-cyanoacetamide (**47**)^70^

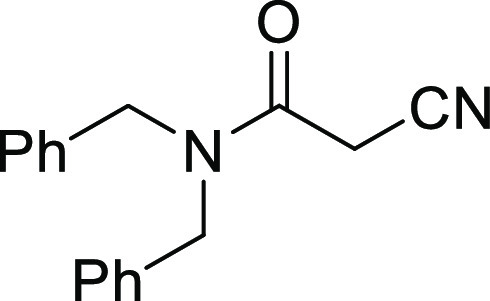



This compound was prepared according to the general procedure^70^ from *N*,*N*-dibenzylamine (**39**) (9.47 g, 48.0 mmol), 2-cyanoacetic acid (4.09 g, 50.0 mmol), *N,N′*-dicyclohexylcarbodiimide (9.90 g, 50.0 mmol), and 4-dimethylaminopyridine (0.31 g, 2.80 mmol) in dichloromethane (60 mL). Colorless crystals (4.43 g, 75%). Mp: 112–115 °C (lit.^70^ mp 117–119 °C). ν_max_/cm^–1^ (ATR): 2260 (CN), 1657 (CO). ^1^H NMR (CDCl_3_, 400 MHz): δ 7.45–7.10 (m, 10H, 10 × Ar*H*), 4.66 (s, 2H, NC*H*_2_), 4.44 (s, 2H, NC*H*_2_), 3.53 (s, 2H, C*H*_2_CN). ^13^C{^1^H} NMR (CDCl_3_, 100.6 MHz): 162.5 (C), 136.0 (C), 134.9 (C), 129.4 (CH), 128.9 (CH), 128.5 (CH), 128.3 (CH), 128.0 (CH), 126.2 (CH), 113.9 (C), 50.7 (CH_2_), 49.6 (CH_2_), 25.3 (CH_2_).

##### *N*-*tert*-Butyl-2-cyano-*N*-(4′-chlorobenzyl)acetamide (**48**)^70^

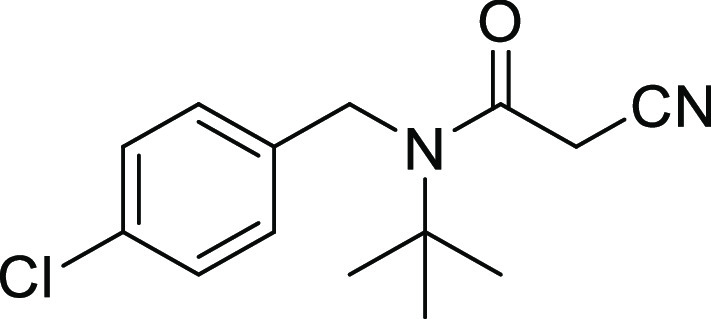



This compound was prepared according to the general procedure^70^ from *N*-(4′-chlorobenzyl)-2-methylpropan-2-amine (**40**) (3.44 g, 17.4 mmol), 2-cyanoacetic acid (1.61 g, 19.3 mmol), *N,N′*-dicyclohexylcarbodiimide (3.90 g, 19.3 mmol), and 4-dimethylaminopyridine (0.11 g, 0.90 mmol) in dichloromethane (40 mL). Colorless crystals (2.62 g, 57%). Mp: 127–130 °C (lit.^70^ mp 125–127 °C). ν_max_/cm^–1^ (ATR): 2259 (CN), 1658 (CO). ^1^H NMR (CDCl_3_, 400 MHz) 7.41–7.36 [m, 2H, C(3′)*H* and C(5′)*H*], 7.14 [d, 2H, *J* = 8.5 Hz, C(2′)*H* and C(6′)*H*], 4.54 (s, 2H, C*H*_*2*_N), 3.41 (s, 2H, C*H*_*2*_CN), 1.46 [s, 9H, C(C*H*_*3*_)_3_]. ^13^C{^1^H} NMR (CDCl_3_, 100.6 MHz) 162.8 (C), 136.0 (C), 133.7 (C), 129.5 (CH), 126.6 (CH), 114.3 (C), 59.4 (C), 48.6 (CH_2_), 28.4 (CH_3_), 27.8 (CH_2_).

##### *N*-Benzyl-*N*-*tert*-butyl-2-cyanoacetamide (**49**)^70^

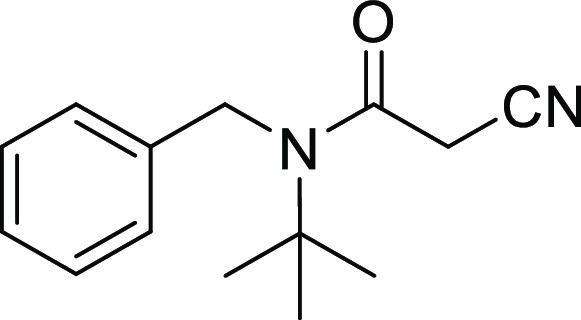



This compound was prepared according to the general procedure^70^ from *N*-(benzyl)-2-methylpropan-2-amine (**41**) (6.81 g, 42.0 mmol), 2-cyanoacetic acid (3.66 g, 43.0 mmol), *N,N′*-dicyclohexylcarbodiimide (8.87 g, 43.0 mmol) and 4-dimethylaminopyridine (0.25 g, 2.10 mmol) in dichloromethane (60 mL). Colorless crystals (8.50 g, 88%). Mp: 97–100 °C (lit.^70^ mp 97–99 °C). ν_max_/cm^–1^ (ATR): 2260 (CN), 1657 (CO). ^1^H NMR (CDCl_3_, 400 MHz): δ 7.44–7.16 (m, 5H, 5 × Ar*H*), 4.57 (s, 2H, C*H*_*2*_N), 3.43 (s, 2H, C*H*_*2*_CN), 1.48 [s, 9H, C(C*H*_*3*_)_3_]. ^13^C{^1^H} NMR (CDCl_3_, 100.6 MHz): δ 162.9 (C), 137.4 (C), 129.3 (CH), 127.8 (CH), 125.2 (CH), 114.5 (C), 59.3 (C), 49.2 (CH_2_), 28.4 (CH_3_), 27.9 (CH_2_).

##### *N*-*tert*-Butyl-2-cyano-*N*-(4′-fluorobenzyl)acetamide (**50**)^70^

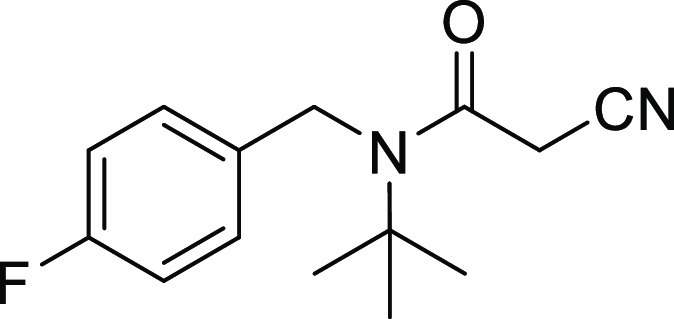



This compound was prepared according to the general procedure^70^ from *N*-(4′-fluorobenzyl)-2-methylpropan-2-amine (**42**) (6.24 g, 34.0 mmol), 2-cyanoacetic acid (3.03 g, 36.0 mmol), *N,N′*-dicyclohexylcarbodiimide (7.35 g, 36.0 mmol), and 4-dimethylaminopyridine (0.210 g, 1.73 mmol) in dichloromethane (60 mL). Colorless crystals (4.44 g, 52%). Mp: 101–105 °C (lit.^70^ mp 98–101 °C). ν_max_/cm^–1^ (ATR): 2260 (CN), 1651 (CO). ^1^H NMR (CDCl_3_, 400 MHz): δ 7.21–7.06 (m, 4H, 4 × Ar*H*), 4.55 (s, 2H, NC*H*_*2*_), 3.44 (s, 2H, C*H*_*2*_CN), 1.46 [s, 9H, C(C*H*_*3*_)_3_]. ^13^C{^1^H} NMR (CDCl_3_, 75.5 MHz): δ 162.9 (C), 162.2 (C, d, ^1^*J*_CF_ = 247.0 Hz), 133.1 (C, d, ^4^*J*_CF_ = 3.2 Hz), 126.9 (CH, d, ^3^*J*_CF_ = 8.2 Hz), 116.3 (CH, d, ^2^*J*_CF_ = 21.7 Hz), 114.4 (C), 59.4 (C), 48.6 (CH_2_), 28.4 (CH_3_), 27.8 (CH_2_). ^19^F{^1^H} NMR (CDCl_3_, 376.5 MHz): δ −114.4.

##### *N*-*tert*-Butyl-2-cyano-*N*-(4′-methylbenzyl)acetamide (**51**)^70^

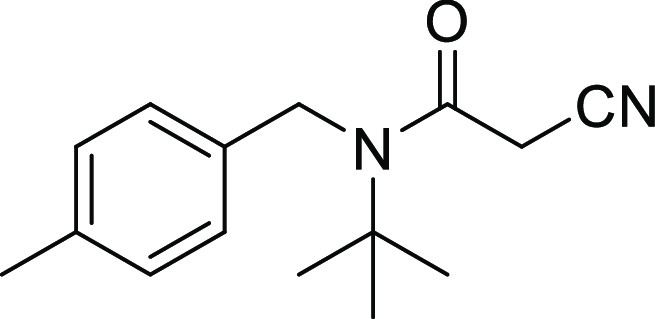



This compound was prepared according to the general procedure^70^ from *N*-(4′-methylbenzyl)-2-methylpropan-2-amine (**43**) (3.00 g, 17.0 mmol), 2-cyanoacetic acid (1.53 g, 18.0 mmol), *N,N′*-dicyclohexylcarbodiimide (3.71 g, 18.0 mmol), and 4-dimethylaminopyridine (0.10 g, 0.81 mmol) in dichloromethane (45 mL). Colorless crystals (1.57 g, 57%). Mp: 162–165 °C (lit.^70^ mp 159–161 °C). ν_max_/cm^–1^ (ATR): 2259 (CN), 1661 (CO). ^1^H NMR (CDCl_3_, 400 MHz): δ 7.20 [d, 2H, *J* = 8.0 Hz, 2 × Ar*H*], 7.07 [d, 2H, *J* = 8.0 Hz, 2 × Ar*H*], 4.53 (s, 2H, C*H*_*2*_N), 3.44 (s, 2H, C*H*_*2*_CN), 2.36 [s, 3H, C(4′)(C*H*_*3*_)], 1.47 [s, 9H, C(C*H*_*3*_)_3_]. ^13^C{^1^H} NMR (CDCl_3_, 100.6 MHz): δ 163.0 (C), 137.5 (C), 134.3 (C), 129.9 (CH), 125.2 (CH), 114.6 (C), 59.3 (C), 48.9 (CH_2_), 28.4 (CH_3_), 27.9 (CH_2_), 21.0 (CH_3_).

##### *N*-*tert*-Butyl-2-cyano-*N*-(4′-bromobenzyl)acetamide (**52**)^70^

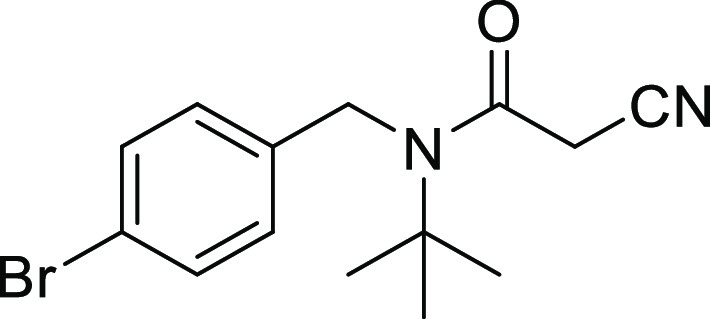



This compound was prepared according to the general procedure^70^ from *N*-(4′-bromobenzyl)-2-methylpropan-2-amine (**44**) (5.78 g, 23.8 mmol), 2-cyanoacetic acid (2.04 g, 25.0 mmol), *N,N′*-dicyclohexylcarbodiimide (4.95 g, 25.0 mmol), and 4-dimethylaminopyridine (0.12 g, 1.20 mmol) in dichloromethane (60 mL). Colorless crystals (5.10 g, 69%). Mp: 112–115 °C (lit.^70^ mp 115–117 °C). ν_max_/cm^–1^ (ATR): 2260 (CN), 1661 (CO). ^1^H NMR (CDCl_3_, 400 MHz): δ 7.54 (d, 2H, *J* = 8.4 Hz, 2 × Ar*H*), 7.08 (d, 2H, *J* = 8.3 Hz, 2 × Ar*H*), 4.52 (s, 2H, C*H*_*2*_N), 3.40 (s, 2H, C*H*_*2*_CN), 1.46 [s, 9H, C(C*H*_*3*_)_3_]. ^13^C{^1^H} NMR (CDCl_3_, 100.6 MHz): δ 162.8 (C), 136.5 (C), 132.5 (CH), 127.0 (CH), 121.7 (C), 114.2 (C), 59.5 (C), 48.7 (CH_2_), 28.4 (CH_3_), 27.8 (CH_2_).

##### N-*tert*-Butyl-2-cyano-*N*-(3′,5′-dimethylbenzyl)acetamide (**53**)

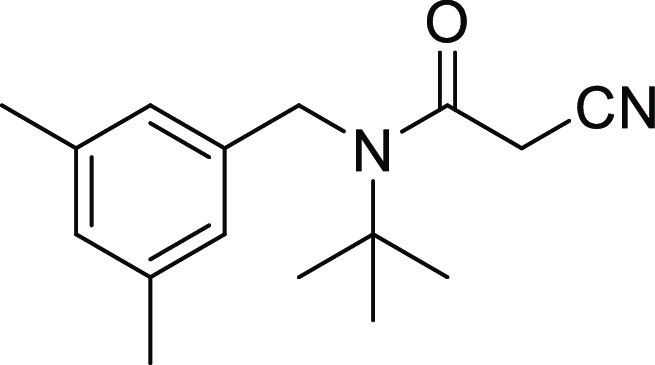



This compound was prepared according to the general procedure^70^ from *N*-(3′,5′-dimethylbenzyl)-2-methylpropan-2-amine (**45**) (3.18 g, 16.6 mmol), 2-cyanoacetic acid (1.55 g, 17.7 mmol), *N,N′*-dicyclohexylcarbodiimide (3.97 g, 17.7 mmol), and 4-dimethylaminopyridine (0.11 g, 0.90 mmol) in dichloromethane (60 mL). Colorless crystals (2.95 g, 69%). Mp: 160–164 °C. ν_max_/cm^–1^ (ATR): 2260 (CN), 1659 (CO). ^1^H NMR (CDCl_3_, 400 MHz): δ 6.93 [s, 1H, C(4′)*H*], 6.77 [s, 2H, C(2′)*H* and C(6′)*H*], 4.49 (s, 2H, NC*H*_*2*_), 3.43 (s, 2H, C*H*_2_CN), 2.32 [s, 6H, C(3′)C*H*_*3*_ and C(5′)C*H*_*3*_], 1.48 [s, 9H, C(C*H*_*3*_)_3_]. ^13^C{^1^H} NMR (CDCl_3_, 100.6 MHz): δ 163.0 (C), 139.0 (C), 137.3 (C), 129.4 (CH), 122.9 (CH), 114.6 (C), 59.3 (C), 49.1 (CH_2_), 28.4 (CH_3_), 27.9 (CH_2_), 21.4 (CH_3_). HRMS (ESI) *m*/*z*: [M + H]^+^ Calcd for C_16_H_23_N_2_O 259.1805; Found 259.1802.

##### *N*-*tert*-Butyl-2-cyano-*N*-(pyridin-4′-ylmethyl)acetamide (**54**)

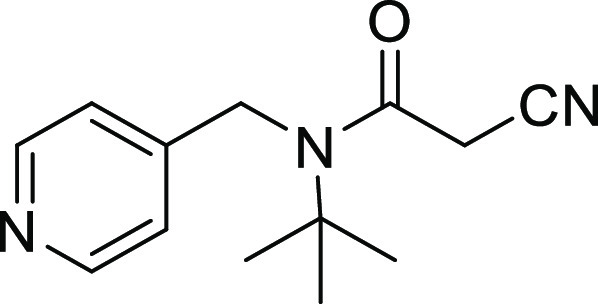



This compound was prepared according to the general procedure^70^ from *N*-(pyridin-4′-ylmethyl)-2-methylpropan-2-amine (**46**) (6.99 g, 42.6 mmol), *N*′,*N*′-dicyclohexylcarbodiimide (8.87 g, 43.0 mmol), cyanoacetic acid (3.66, 43.0 mmol), and 4-dimethylaminopyridine (0.24 g, 1.9 mmol) in dichloromethane (60 mL). The crude residue was not recrystallized but was washed with warm diethyl ether to afford pure *acetamide***54** as a pale brown solid (8.09 g, 82%). Mp: 108–111 °C. ν_max_/cm^–1^ (ATR): 2977, 2256, 1660, 1602, 1412, 1362, 1191. HRMS (ESI) *m*/*z*: [M + H]^+^ Calcd for C_13_H_18_N_3_O 232.1444; Found 232.1440. *Two sets of signals exist due to the presence of rotamers*.

Major rotamer (83%): ^1^H NMR (CDCl_3_, 400 MHz): δ 8.65 [d, 2H, *J* = 3.0 Hz, C(2)*H* and C(6)*H*], 7.17 [d, 2H, *J* = 5.7 Hz, C(3)*H* and C(5)*H*], 4.58 (s, 2H, C*H*_*2*_N), 3.40 (s, 2H, C*H*_*2*_CN), 1.47 [s, 9H, C(C*H*_*3*_)_3_]. ^13^C{^1^H} NMR (CDCl_3_, 100.6 MHz): δ 162.8 (C), 150.7 (CH), 147.0 (C), 120.5 (CH), 114.1 (C), 59.6 (C), 48.4 (CH_2_), 28.5 (CH_3_), 27.9 (CH_2_).

Minor rotamer (17%): ^1^H NMR (CDCl_3_, 400 MHz): δ 8.55 [d, 2H, *J* = 5.9 Hz, C(2)*H* and C(6)*H*], 7.47 [d, 2H, *J* = 5.8 Hz, C(3)*H* and C(5)*H*], 3.94 (s, 2H, C*H*_*2*_N), 3.23 (s, 2H, C*H*_*2*_CN),1.23 [s, 9H, C(C*H*_*3*_)_3_]. ^13^C{^1^H} NMR (CDCl_3_, 75.5 MHz) 166.7 (C), 150.0 (CH), 124.9 (CH), 117.4 (C), 56.5 (C), 44.4 (CH_2_), 27.3 (CH_2_), 26.0 (CH_3_); signal for C(1) not observed.

### Synthesis of α-Diazoketones via Debenzoylative Diazo Transfer

#### 3-Diazopentan-2,4-dione (**23**)^101^

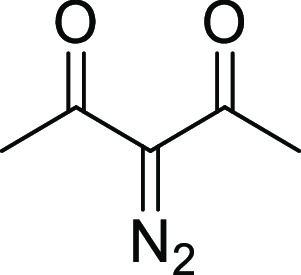



An aqueous solution of sodium azide (10 mL, 1.95 g, 30.0 mmol, 3.0 mL min^–1^) was pumped through a micromixer T-piece where it met a dichloromethane solution of trifluoromethanesulfonic anhydride (10 mL, 0.85 mL, 5.0 mmol, 3.0 mL min^–1^); the combined stream passed through a reactor coil (4 × 10 mL, rt). After all reagent solutions had been charged, the combined flow rate was changed to 0.2 mL min^–1^ to give a residence time of 1 h. The reactor effluent passed through a T-piece where it met a stream of saturated aqueous sodium bicarbonate (0.1 mL min^–1^). The reaction stream was passed through a back-pressure regulator (8 bar). The biphasic effluent was then separated by an in-line liquid–liquid separator. The organic effluent (25 mL) was added to flask containing a suspension of sodium 2,4-pentanedionate (0.631 g, 5.0 mmol) **6** in dichloromethane (10 mL). The resulting suspension was stirred at room temperature for 24 h. The suspension was then washed with water (20 mL), dried and concentrated under reduced pressure. The crude residue was purified by flash chromatography on silica gel with hexane/ethyl acetate (90:10) as eluent and isolated as a yellow oil (0.400 g, 65%). ν_max_/cm^–1^ (ATR): 2127, 1663. ^1^H NMR (CDCl_3_, 300 MHz): δ 2.44 (s, 6H, C(O)C*H*_*3*_).

#### General Flow Procedure for Telescoped Generation of TfN_3_**22** and Direct Use for Diazo Transfer

An aqueous solution of sodium azide (10 mL, 3.0 M, 10 equiv, 3.0 mL min^–1^) was pumped through a micromixer T-piece where it met a dichloromethane solution of trifluoromethanesulfonic anhydride (10 mL, 0.5 M, 1.67 equiv, 3.0 mL min^–1^); the combined stream passed through a reactor coil (4 × 10 mL, rt). After all reagent solutions had been charged, the combined flow rate was changed to 0.2 mL min^–1^ to give a residence time of 1 h. The reactor effluent passed through a T-piece where it met a stream of saturated aqueous sodium bicarbonate (0.1 mL min^–1^). The reaction stream was passed through a back-pressure regulator (8 bar). The biphasic effluent was then separated by an in-line liquid–liquid separator. The organic effluent (25 mL) was directly fed to another pump. The pump delivered the separated triflyl azide solution (25 mL, 0.17 mL min^–1^) to a T-piece where it met a solution of the relevant acceptor substrate (25 mL, 0.12 M, 1 equiv, [base] 0.128 M, 1.1 equiv, 0.17 mL min^–1^) which was then passed through a reactor coil (4 × 10 mL, rt, τ = 2 h) and subsequently through a glass column packed with silica gel (100 mm × 10 mm internal diameter) and then finally passed through a back pressure regulator (8 bar). The reactor effluent was analyzed by IR spectroscopy and concentrated under reduced pressure affording the crude α-diazoketone which was subsequently purified by flash chromatography on silica gel with hexane/ethyl acetate as eluent.

For the synthesis of compounds **55**–**62**, the triflyl azide solution was collected over KOH prior to use in diazo transfer.

##### 2-Diazo-5-methyl-5-(3′,4′,5′-trimethylphenyl)hexan-3-one (**8**)^14^

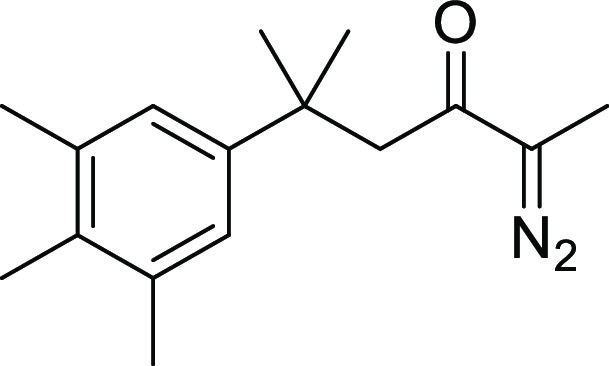



This compound was prepared according to the general flow procedure from 2,5-dimethyl-1-phenyl-5-(3′,4′,5′-trimethylphenyl)hexane-1,3-dione (**18**) and DBU (25 mL, 0.12 M, [DBU] 0.128 M), aqueous sodium azide (10 mL, 3.0 M), and a dichloromethane solution of trifluoromethanesulfonic anhydride (10 mL, 0.5 M). The crude product was purified by flash chromatography on silica gel with hexane/ethyl acetate (95:5) as eluent affording *α-diazoketone***8** as a yellow oil (0.558 g, 72%). ν_max_/cm^–1^ (ATR): 2963, 2061, 1712, 1624, 1445, 1346. ^1^H NMR (CDCl_3_, 300 MHz): δ 6.98 [s, 2H, C(2′)*H* and C(6′)*H*], 2.68 [s, 2H, C(4)C*H*_*2*_], 2.28 [s, 6H, C(3′)C*H*_*3*_ and C(5′)C*H*_*3*_], 2.14 [s, 3H, C(4′)C*H*_*3*_], 1.83 [s, 3H, C(1)*H*_*3*_], 1.43 [s, 6H, C(5)(C*H*_*3*_)_2_]. ^13^C{^1^H} NMR (CDCl_3_, 75.5 MHz): δ 193.4 (C), 145.0 (C), 136.1 (C), 132.7 (C), 124.7 (CH), 63.8 (C), 50.7 (CH_2_), 37.9 (C), 28.5 (CH_3_), 20.9 (CH_3_), 15.1 (CH_3_), 8.3 (CH_3_).

##### 2-Diazo-5-methyl-5-phenylhexan-3-one (**11**)^14^

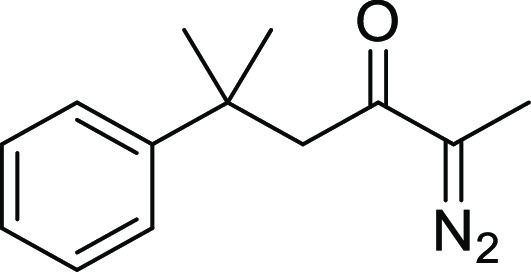



This compound was prepared according to the general flow procedure from 2,5-dimethyl-1,5-diphenylhexane-1,3-dione (**19**) and DBU (25 mL, 0.12 M, [DBU] 0.128 M), aqueous sodium azide (10 mL, 3.0 M) and a dichloromethane solution of trifluoromethanesulfonic anhydride (10 mL, 0.5 M). The crude product which was purified by flash chromatography on silica gel with hexane/ethyl acetate (85:15) as eluent affording *α-diazoketone***11** as a yellow oil (0.519 g, 80%). ν_max_/cm^–1^ (ATR): 2964, 2061, 1621, 1348, 1266, 1054. ^1^H NMR (CDCl_3_, 300 MHz): δ 7.37–7.18 [m, 5H, Ar*H*], 2.69 [s, 2H, C(4)*H*_*2*_], 1.80 [s, 3H, C(1)*H*_*3*_], 1.47 [s, 6H, C(5)(C*H*_*3*_)_2_]. ^13^C{^1^H} NMR (CDCl_3_, 75.5 MHz): δ 193.0 (C), 148.0 (C), 128.2 (CH), 126.1 (CH), 125.5 (CH), 63.8 (C), 50.7 (CH_2_), 38.4 (C), 28.5 (CH_3_), 8.2 (CH_3_).

##### 2-Diazo-5-methyl-5-(4′-chlorophenyl)hexan-3-one (**12**)^14^

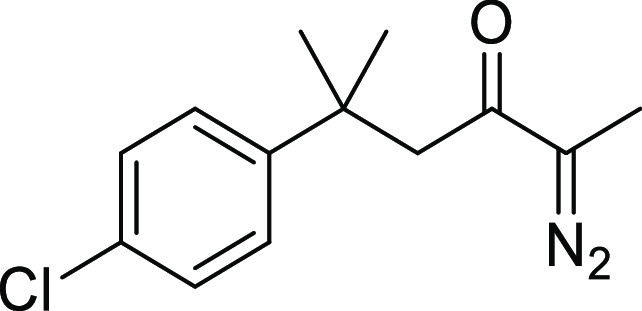



This compound was prepared according to the general flow procedure from a dichloromethane solution of 2,5-dimethyl-1-phenyl-5-(4′-chlorophenyl)hexane-1,3-dione(**20**) and DBU (25 mL, 0.12 M, [DBU] 0.128 M), aqueous sodium azide (10 mL, 3.0 M), and a dichloromethane solution of trifluoromethanesulfonic anhydride (10 mL, 0.5 M). The crude product was purified by flash chromatography on silica gel with hexane/ethyl acetate (95:5) as eluent affording affording *α-diazoketone***12** as a yellow oil (0.564 g, 75%). ν_max_/cm^–1^ (ATR): 2965, 2061, 1621, 1353, 1279, 1011. ^1^H NMR (CDCl_3_, 300 MHz): δ 7.30–7.26 [m, 4H, Ar*H*], 2.68 [s, 2H, C(4)*H*_*2*_], 1.82 [s, 3H, C(1)*H*_*3*_], 1.45 [s, 6H, C(5)(C*H*_*3*_)_2_]. ^13^C{^1^H} NMR (CDCl_3_, 75.5 MHz): δ 192.5 (C), 147.6 (C), 131.8 (C), 128.2 (CH), 127.0 (CH), 63.6 (C), 50.3 (CH_2_), 38.0 (C), 29.1 (CH_3_), 8.1 (CH_3_).

##### 2-Diazo-5-methyl-5-(4′-fluorophenyl)hexan-3-one (**13**)^14^

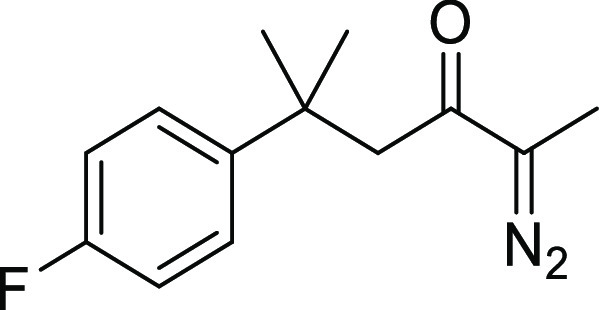



This compound was prepared according to the general flow procedure from a dichloromethane solution of 2,5-dimethyl-1-phenyl-5-(4′-fluorophenyl)hexane-1,3-dione (**21**) and DBU (25 mL, 0.12 M, [DBU] 0.128 M), aqueous sodium azide (10 mL, 3.0 M), and a dichloromethane solution of trifluoromethanesulfonic anhydride (10 mL, 0.5 M). The crude product was purified by flash chromatography on silica gel with hexane/ethyl acetate (95:5) as eluent affording *α-diazoketone***13** as a yellow oil (0.491 g, 70%). ν_max_/cm^–1^ (ATR): 2967, 2066, 1625. ^1^H NMR (CDCl_3_, 300 MHz): δ 7.33–7.28 [m, 2H, 2 × Ar*H*], 7.02–6.96 [m, 2H, 2 × Ar*H*], 2.68 [s, 2H, C(4)*H*_*2*_], 1.81 [s, 3H, C(1)*H*_*3*_], 1.46 [s, 6H, C(5)(C*H*_*3*_)_2_]. ^13^C{^1^H} NMR (CDCl_3_, 75.5 MHz): δ 192.8 (C), 161.2 (C, d, ^1^*J*_CF_ 244.2), 143.6 (C), 127.1 (CH, d, ^3^*J*_CF_ 7.8), 114.8 (CH, d, ^2^*J*_CF_ 20.9), 63.7 (C), 50.7 (CH_2_), 38.0 (C), 28.8 (CH_3_), 8.1 (CH_3_).

##### 5-Diazo-2-methyl-2-phenylheptan-4-one (**26**)

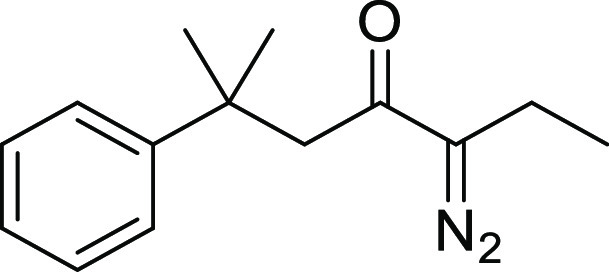



This compound was prepared according to the general flow procedure from a dichloromethane solution of 2-ethyl-5-methyl-1,5-diphenylhexan-1,3-dione (**24**) and DBU (25 mL, 0.12 M, [DBU] 0.128 M), aqueous sodium azide (10 mL, 3.0 M), and a dichloromethane solution of trifluoromethanesulfonic anhydride (10 mL, 0.5 M). The crude product was purified by flash chromatography on silica gel with hexane/ethyl acetate (90:10) as eluent affording *α-diazoketone***26** as a yellow oil (0.552 g, 80%). ν_max_/cm^–1^ (ATR): 2928, 2067, 1609. ^1^H NMR (CDCl_3_, 300 MHz): δ 7.39–7.27 [m, 4H, Ar*H*], 7.24–7.15 [m, 1H, Ar*H*], 2.67 [s, 2H, C(3)*H*_*2*_], 2.22 [q, 2H, *J* = 7.3 Hz, C(6)*H*_2_], 1.48 [s, 6H, C(2)(C*H*_*3*_)_2_], 0.94 [t, 3H, *J* = 7.4 Hz, C(7)*H*_3_]. ^13^C{^1^H} NMR (CDCl_3_, 100.6 MHz): δ 192.4 (C), 147.9 (C), 128.2 (CH), 126.1 (CH), 125.6 (CH), 69.5 (C), 51.0 (CH_2_), 38.5 (C), 28.6 (CH_3_), 16.0 (CH_3_), 11.2 (CH_2_). HRMS (ESI) *m*/*z*: [M + Na]^+^ Calcd for C_14_H_18_N_2_ONa 253.1311; Found 253.1312.

##### 1-Cyclohexyl-2-diazo-5-methyl-5-phenylhexan-3-one (**27**)

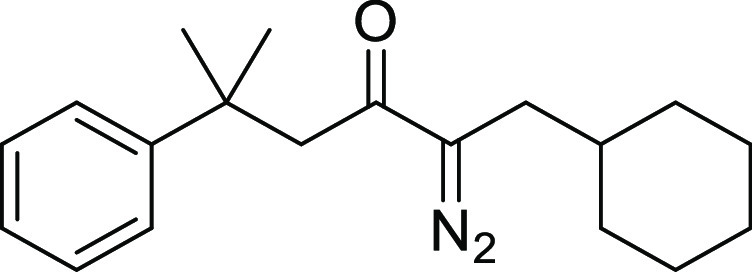



This compound was prepared according to the general flow procedure from 2-(cyclohexylmethyl)-5-methyl-1,5-diphenylhexane-1,3-dione (**25**) and DBU (25 mL, 0.12 M, [DBU] 0.128 M), aqueous sodium azide (10 mL, 3.0 M) and a dichloromethane solution of trifluoromethanesulfonic anhydride (10 mL, 0.5 M). The crude product was purified by flash chromatography on silica gel with hexane/ethyl acetate (95:5) as eluent affording *α-diazoketone***27** as a yellow oil (0.812 g, 91%). ν_max_/cm^–1^ (ATR): 2922, 2060, 1627. ^1^H NMR (CDCl_3_, 400 MHz): 7.39–7.27 [m, 4H, Ar*H*], 7.23–7.16 [m, 1H, C(4′)*H*], 2.69 [s, 2H, C(4)*H*_2_], 2.03 [2H, d, *J* = 7.1 Hz, C(2)*H*_2_], 1.78–1.38 [m, 11H, contains singlet at 1.48 ppm for C(5)(C*H*_3_)_2_], [C(5)(C*H*_3_)_2_], 1.32–0.99 [m, 4H], 0.90–0.69 [2H, m]. ^13^C{^1^H} NMR (CDCl_3_, 100.6 MHz) 192.6 (C), 147.8 (C), 128.2 (CH), 126.1 (CH), 125.6 (CH), 67.0 (C), 50.8 (CH_2_), 38.5 (C), 36.7 (CH), 32.6 (CH_2_), 30.4 (CH_2_), 28.8 (CH_3_), 26.2 (CH_2_), 26.0 (CH_2_). HRMS (ESI) *m*/*z*: [M + Na]^+^ Calcd for C_19_H_26_N_2_ONa 321.1937; Found 321.1940.

##### 1-(4-Chlorophenyl)-3-diazopyrrolidin-2-one (**29**)^82^

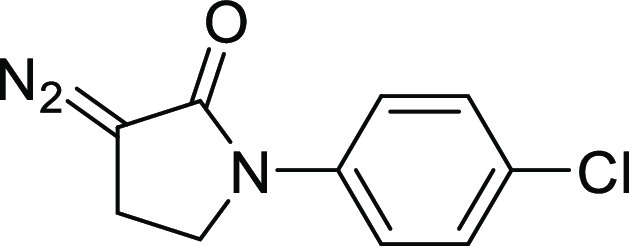



An aqueous solution of sodium azide (10 mL, 3.0 M, 10 equiv, 3.0 mL min^–1^) was pumped through a micromixer T-piece where it met a toluene solution of trifluoromethanesulfonic anhydride (10 mL, 0.5 M, 1.67 equiv, 3.0 mL min^–1^); the combined stream passed through a reactor coil (4 × 10 mL, rt). After all reagent solutions had been charged, the combined flow rate was changed to 0.2 mL min^–1^ to give a residence time of 1 h. The reactor effluent passed through a T-piece where it met a stream of aqueous sodium bicarbonate (5% w/v, 0.9 mL min^–1^). The reaction stream was passed through a back-pressure regulator (8 bar). The biphasic effluent was then separated by an in-line liquid–liquid separator. The organic effluent (25 mL) was directly fed to another pump. The pump delivered the separated triflyl azide solution (25 mL, 2.0 mL min^–1^) to a T-piece where it met a solution of ethyl-2-hydroxy-2-(2-oxo-1-(4-chlorophenyl)pyrrolidin-3-ylidene)acetate (**28**)^82^ and DBU in dichloromethane (25 mL, 0.12 M, [DBU] 0.128 M, 1.1 equiv relative to **28**, 2.0 mL min^–1^) and was then passed through a reactor coil (4 × 10 mL, rt). When both reagent solutions had been charged, the combined flow rate was reduced to 0.5 mL min^–1^ to give a residence time of 1 h, after which the reactor effluent subsequently passed through a back pressure regulator (8 bar). The reactor effluent was analyzed by IR spectroscopy and concentrated under reduced pressure to give the crude product which was subsequently purified by chromatography on silica gel (treated by adding 1% weight of triethylamine to the dry silica prior to packing) using hexane:chloroform:ethanol (50:49:1) as eluent affording *α-diazoamide***29** as a bright orange crystalline solid (0.48 g, 73%). Mp: 131–135 °C. ν_max_/cm^–1^ (ATR): 2089 (N_2_), 1664 (CO). ^1^H NMR (CDCl_3_, 400 MHz): δ 7.53 (d, 2H, *J* = 9.0 Hz, Ar*H*), 7.29 (d, 2H, *J* = 9.0 Hz, Ar*H*), 3.80 [dd, 2H, *J* = 7.6 Hz, 7.0 Hz, C(4)*H*_2_ or C(5)*H*_2_], 3.22 [dd, 2H, *J* = 8.2 Hz, 7.2 Hz, C(4)*H*_2_ or C(5)*H*_2_]. ^13^C {^1^H} NMR (CDCl_3_, 100.6 MHz,): δ 166.9 (C), 138.6 (C), 128.8 (CH), 128.7 (C), 119.9 (CH), 54.0 (C), 44.8 (CH_2_), 17.9 (CH_2_). HRMS (ESI) *m*/*z*: [M + Na^+^] Calcd for C_10_H_8_N_3_O^35^ClNa 244.0248; Found 244.0218.

### Telescoped Aromatic Additions of α-Diazoketones

#### 3,8a-Dihydro-3,3,8a-trimethylazulen-1(2*H*)-one (**31**)^14^

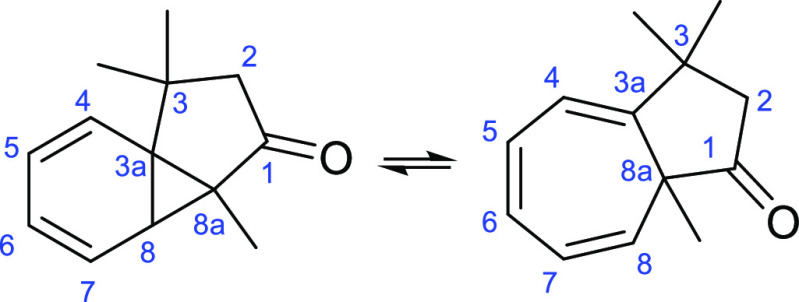



An aqueous solution of sodium azide (10 mL, 30 mmol, 3.0 M, 3.0 mL min^–1^) was pumped through a micromixer T-piece where it met a dichloromethane solution of triflic anhydride (10 mL, 5 mmol, 0.5 M, 3.0 mL min^–1^); the combined biphasic stream was passed through a reactor coil (4 × 10 mL, rt). After all reagent solutions had been charged, the combined flow rate was changed to 0.2 mL min^–1^ to give a residence time of 1 h for triflyl azide generation. The reactor effluent for the first step passed through a T-piece where it met a stream of saturated aqueous sodium bicarbonate (0.1 mL min^–1^). The combined stream was passed through a back pressure regulator (8 bar). The biphasic effluent was then separated by an in-line liquid–liquid separator and the dichloromethane effluent (25 mL), containing triflyl azide (**22**), was collected over KOH pellets. The separated dichloromethane triflyl azide solution (25 mL, 0.17 mL min^–1^) was pumped to a T-piece where it met a dichloromethane solution of 2,5-dimethyl-1,5-diphenylhexane-1,3-dione (**19**) and DBU (25 mL, 3 mmol, 0.12 M, [DBU] 0.128 M, 0.17 mL min^–1^) and the combined stream was then passed through a reactor coil (4 × 10 mL, rt, 120 min residence time). The diazo-transfer effluent (75 mL) was also collected over KOH pellets, and a 25 mL portion, containing the α-diazoketone **11**, was pumped forward to undergo aromatic addition; this 25 mL solution was passed through a silica gel plug (glass column, 100 mm × 10 mm internal diameter, rt, 0.75 mL min^–1^) to remove polar components. Immediately after eluting from the silica gel plug, the effluent was passed through a packed bed reactor containing IPB catalyst **9** (0.417 g, 10 mol % based on **19**, glass column, 150 × 66 mm internal diameter, 0.75 mL min^–1^, 45 °C). The reactor effluent was analyzed by IR spectroscopy and concentrated under reduced pressure to give the crude product, which was purified by flash chromatography on silica gel with hexane/ethyl acetate (95:5) as eluent, affording *azulenone***31** as a pale yellow oil (0.095 g, 50%, 60% ee).^85^ [α]_D_^20^ +13.86 (*c* 1.05, CHCl_3_). ν_max_/cm^–1^ (ATR): 3042, 2926, 1747 (CO), 1715 (CO). ^1^H NMR (CDCl_3_, 300 MHz): δ 6.43–6.23 [m, 3H, C(4)*H*, C(5)*H* and C(6)*H*], 6.14–6.07 [m, 1H, C(7)*H*], 4.18 [d, 1H, *J* = 8.0 Hz, C(8)*H*], 2.28 [B of AB, 1H, *J*_AB_ = 17.3 Hz, one of C(2)*H*_2_], 2.20 [A of AB, 1H, *J*_AB_ = 17.3 Hz, one of C(2)*H*_2_], 1.31, 1.14 [2 × s, 2 × 3H, C(3)(C*H*_*3*_)_2_], 0.76 [s, 3H, C(8a)C*H*_*3*_]. ^13^C{^1^H} NMR (CDCl_3_, 75.5 MHz): δ 218.5 (C), 127.1 (CH), 126.8 (CH), 125.3 (CH), 119.7 (CH), 109.6 (C), 84.9 (CH), 50.1 (CH_2_), 40.7 (C), 38.6 (C), 28.8 (CH_3_), 28.6 (CH_3_), 11.4 (CH_3_). Also isolated as a side product during the aromatic addition reaction of α-diazoketone **11** was the side product:

#### 5-Methyl-5-phenylhexane-2,3-dione (**34**)^87^

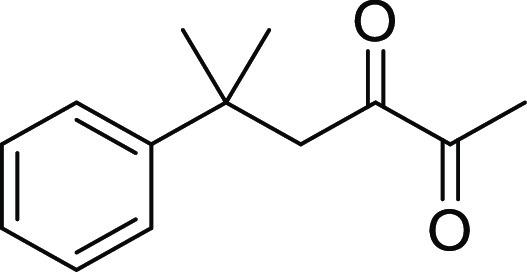



The compound was observed in the ^1^H NMR spectrum of the crude product mixture in a ratio of 1:0.3 (**30**:**34**). The *2,3-diketone***34** was isolated after chromatography on silica gel with hexane/ethyl acetate as a yellow oil (0.039 g, 19%). ν_max_/cm^–1^ (film) 2967, 1713. ^1^H NMR (CDCl_3_, 300 MHz): δ 7.37–7.25 (m, 4H, Ar*H*), 7.22–7.15 (m, 1H, Ar*H*), 3.09 [s, 2H, C(4)*H*_2_], 1.98 [s, 3H, C(1)*H*_3_], 1.45 [s, 6H, C(5)(C*H*_3_)_2_]. ^13^C{^1^H} (CDCl_3_, 100.6 MHz): δ 199.1 (C), 198.1 (C), 147.2 (C), 128.3 (CH), 126.3 (CH), 125.7 (CH), 47.9 (CH_2_), 37.5 (C), 29.0 (CH_3_), 23.0 (CH_3_). HRMS (ESI) *m*/*z*: [M + H]^+^ Calcd for C_13_H_17_O_2_ 205.1223; Found 205.1223.

#### 1,2,3b,4-Tetrahydro-1,1,3a,4,11,12-hexamethyl-7-phenyl-4,10-etheno-6*H*,10*H*-cyclopenta[1,3]cyclopropa[1,2-*d*][1,2,4]triazolo[1,2-*a*]pyridazine-3,6,8(3a*H*,7*H*)-trione (**10**)^14^

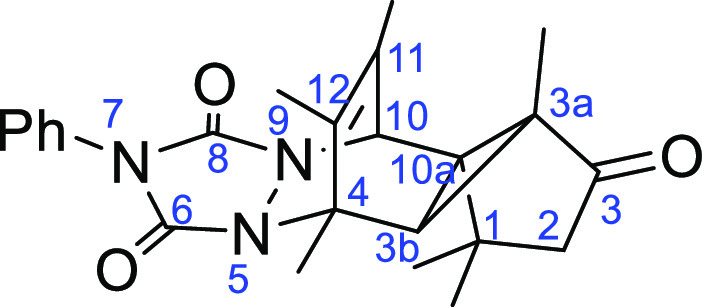



An aqueous solution of sodium azide (10 mL, 30 mmol, 3.0 M, 3.0 mL min^–1^) was pumped through a micromixer T-piece where it met a dichloromethane solution of triflic anhydride (10 mL, 5 mmol, 0.5 M, 3.0 mL min^–1^); the combined biphasic stream was passed through a reactor coil (4 × 10 mL, rt). After all reagent solutions had been charged, the combined flow rate was changed to 0.2 mL min^–1^ to give a residence time of 1 h for triflyl azide generation. The reactor effluent for the first step passed through a T-piece where it met a stream of saturated aqueous sodium bicarbonate (0.1 mL min^–1^). The combined stream was passed through a back pressure regulator (8 bar). The biphasic effluent was then separated by an in-line liquid–liquid separator and the dichloromethane effluent (25 mL), containing triflyl azide (**22**), was collected over KOH pellets. The separated dichloromethane triflyl azide solution (25 mL, 0.17 mL min^–1^) was pumped to a T-piece where it met a dichloromethane solution of 2,5-dimethyl-1-phenyl-5-(3′,4′,5′-trimethylphenyl)hexane-1,3-dione (**18**) and DBU (25 mL, 3 mmol, 0.12 M, [DBU] 0.128 M, 0.17 mL min^–1^), and the combined stream was then passed through a reactor coil (4 × 10 mL, rt, 120 min residence time). The diazo-transfer effluent (75 mL) was also collected over KOH pellets, and a 25 mL portion, containing the α-diazoketone **8**, was pumped forward to undergo aromatic addition; this 25 mL solution was passed through a silica gel plug (glass column, 100 mm × 10 mm internal diameter, rt, 0.75 mL min^–1^) to remove polar components. Immediately after eluting from the silica gel plug, the effluent was passed through a packed bed reactor containing IPB catalyst **9** (0.417 g, 10 mol % based on **18**, glass column, 150 × 66 mm internal diameter, 0.75 mL min^–1^, rt). The reactor effluent containing azulenone **32** was collected in a flask containing PTAD (0.175 g, 1.0 mmol) in dichloromethane (10 mL). Once all the effluent was collected, the contents of the flask were stirred for 1 h at room temperature. The reaction solution was analyzed by IR spectroscopy and concentrated under reduced pressure to give the crude product, which was purified by flash chromatography on silica gel with hexane/ethyl acetate (97:3) as eluent afforded the *adduct***10** as a white solid (0.121 g, 30%, 82% ee).^86^ Mp: 176–178 °C. [α]_D_^20^ −64.38 (*c* 0.08, CHCl_3_). ν_max_/cm^–1^ (ATR): 2966, 2927, 1757, 1727, 1699, 1504, 1396. ^1^H NMR (CDCl_3_, 300 MHz): δ 7.47–7.31 (m, 5H, Ar*H*), 5.13 [s, 1H, C(10)*H*], 2.13 [B of AB, 1H, *J* = 17.9 Hz, one of C(2)*H*_*2*_], 2.02 [s, 3H, C(12)C*H*_*3*_], 1.95 [A of AB, 1H, *J* = 17.8 Hz, one of C(2)*H*_*2*_], 1.84 [d, 3H, *J* = 1.1 Hz, C(11)C*H*_*3*_], 1.75 [d, 3H, *J* = 1.1 Hz, C(4)C*H*_*3*_], 1.69 [s, 1H, C(3b)*H*], 1.30, 1.24 [2 × s, 2 × 3H, C(1)(C*H*_*3*_)_2_], 1.12 [s, 3H, C(3a)C*H*_*3*_]. ^13^C NMR (CDCl_3_, 100.6 MHz): δ 211.7 (C), 156.1 (C), 155.4 (C), 131.5 (C), 130.7 (C), 129.6 (C), 129.0 (CH), 128.1 (CH), 125.4 (CH), 66.2 (C), 57.3 (CH), 48.5 (CH_2_), 43.5 (C), 41.0 (C), 36.4 (C), 34.5 (CH), 27.1 (CH_3_), 23.9 (CH_3_), 21.1 (CH_3_), 16.8 (CH_3_), 13.3 (CH_3_), 7.6 (CH_3_). HRMS (ESI) *m*/*z*: [M + H]^+^ Calcd for C_24_H_27_N_3_O_3_ 406.2125; Found 406.2121.

### Telescoped Regitz-Type Diazo Transfer in Flow

#### 2-Diazo-5-methyl-1,5-diphenylhexane-1,3-dione (**36**)

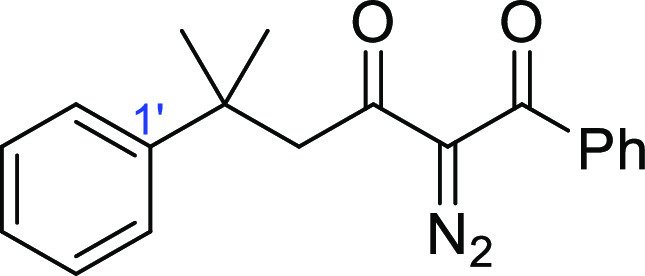



This compound was prepared according to the general flow procedure from a dichloromethane solution of 3-hydroxy-5-methyl-1,5-diphenylhex-2-ene-1-one (**35**), triethylamine (25 mL, 0.12 M, [NEt_3_] 0.128 M), aqueous sodium azide (10 mL, 3.0 M) and a dichloromethane solution of trifluoromethanesulfonic anhydride (10 mL, 0.5 M). The crude product was purified by flash chromatography on silica gel with hexane/ethyl acetate (95:5) as eluent affording *α-diazoketone***36** as a yellow oil (0.864 g, 94%). ν_max_/cm^–1^ (ATR): 2967, 2118, 1640. ^1^H NMR (CDCl_3_, 400 MHz): δ 7.58–7.51 [m, 1H, C(4′′)*H*], 7.50–7.38 (m, 6H, Ar*H*), 7.32–7.25 (m, 2H, Ar*H*), 7.20–7.14 [m, 1H, C(4′)*H*], 3.38 [s, 2H, C(4)*H*_2_], 1.51 [s, 6H, C(5)(C*H*_3_)_2_]. ^13^C{^1^H} NMR (CDCl_3_, 100.6 MHz): δ 191.8 (C), 185.2 (C), 148.0 (C), 137.4 (C), 132.6 (CH), 128.8 (CH), 128.2 (CH), 127.4 (CH), 125.9 (CH), 125.7 (CH), 84.0 (C)_,_ 52.5 (CH_2_), 38.3 (C), 29.2 (CH_3_). HRMS (ESI) *m*/*z*: [M + Na]^+^ Calcd for C_19_H_18_N_2_O_2_Na 329.1260; Found 329.1262.

#### 5-(4′-Chlorophenyl)-2-diazo-5-methyl-3-oxohexanenitrile (**38**)

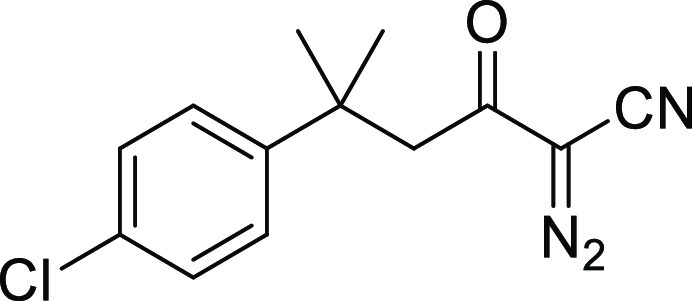



This compound was prepared according to the *general flow procedure* from a dichloromethane solution of 5-methyl-5-(4-chlorophenyl)-3-oxohexanenitrile (**37**), triethylamine (25 mL, 0.12 M, [NEt_3_] 0.128 M), aqueous sodium azide (10 mL, 3.0 M), and a dichloromethane solution of trifluoromethanesulfonic anhydride (10 mL, 0.5 M). The crude product was purified by flash chromatography on silica gel with hexane/ethyl acetate (90:10) as eluent affording *α-diazoketone***38** as a yellow oil (0.667 g, 85%). ν_max_/cm^–1^ (ATR) 2969, 2222 (CN), 2125, 1668. ^1^H NMR (CDCl_3_, 400 MHz): δ 7.32–7.27 (m, 4H, Ar*H*), 2.90 [s, 2H, C(4)*H*_2_], 1.48 [s, 6H, C(5)(C*H*_3_)_2_]. ^13^C{^1^H} NMR (CDCl_3_, 100.6 MHz): δ 188.2 (C), 145.4 (C), 132.2 (C), 128.5 (CH), 127.0 (CH), 108.6 (C), 58.6 (C), 51.5 (CH), 38.1 (C), 28.9 (CH_3_). HRMS (ESI) *m*/*z*: [M + Na]^+^ Calcd for C_13_H_12_N_3_O^35^ClNa 284.0561; Found 284.0559.

#### *N*,*N*-Dibenzyl-2-cyano-2-diazoacetamide (**55**)^70^

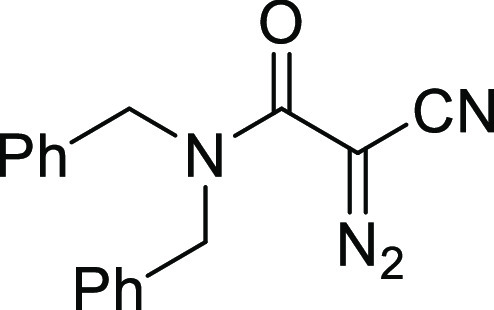



This compound was prepared according to the general flow procedure from *N,N*-(dibenzyl)-2-cyanoacetamide (**47**), triethylamine (25 mL, 0.12 M, [NEt_3_] 0.128 M), aqueous sodium azide (10 mL, 3.0 M), and a dichloromethane solution of trifluoromethanesulfonic anhydride (10 mL, 0.5 M). The crude product was purified by flash chromatography on silica gel with hexane/ethyl acetate (80:20) as eluent affording *α-diazoacetamide***55** as a yellow oil (0.697 g, 80%). ν_max_/cm^–1^ (ATR) 2214 (CN), 2117 (CN_2_), 1626 (CO). ^1^H NMR (CDCl_3_, 400 MHz): δ 7.38–7.19 (m, 10H, Ar*H*), 4.59 (s, 4H, 2 × NC*H*_*2*_). ^13^C{^1^H} NMR (CDCl_3_, 100.6 MHz): δ 159.9 (C), 135.5 (C), 128.9 (CH), 128.1 (CH), 127.7 (CH), 109.7 (C), 50.3 (CH_2_).

#### *N*-*tert*-Butyl-2-cyano-2-diazo-*N*-(4′-chlorobenzyl)acetamide (**56**)^70^

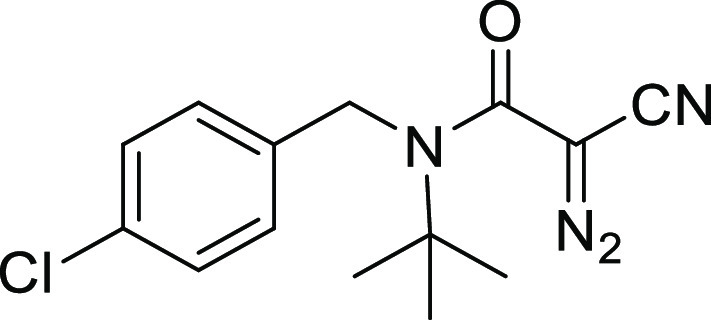



This compound was prepared according to the general flow procedure from *N*-*tert*-butyl-2-cyano-*N*-(4-chlorobenzyl)acetamide (**48**), triethylamine (25 mL, 0.12 M, [NEt_3_] 0.128 M), aqueous sodium azide (10 mL, 3.0 M), and a dichloromethane solution of trifluoromethanesulfonic anhydride (10 mL, 0.5 M). The crude product was purified by flash chromatography on silica gel with hexane/ethyl acetate (80:20) as eluent affording *α-diazoacetamide***56** as a yellow crystalline solid (0.829 g, 95%). Mp: 108–110 °C (lit.^70^ mp 109–113 °C). ν_max_/cm^–1^ (ATR): 2213 (CN), 2118 (CN_2_) 1634 (CO). ^1^H NMR (CDCl_3_, 300 MHz): δ 7.34 (d, 2H, *J* = 8.4 Hz, Ar*H*), 7.16 (d, 2H, *J* = 8.4 Hz, Ar*H*), 4.67 (s, 2H, NC*H*_*2*_), 1.41 [s, 9H, C(C*H*_*3*_)_3_]. ^13^C{^1^H} NMR (CDCl_3_, 75.5 MHz): 160.9 (C), 137.0 (C), 133.3 (C), 128.9 (CH), 127.4 (CH), 109.7 (C), 60.4 (C), 54.8 (C), 49.2 (CH_2_), 28.5 (CH_3_).

#### *N*-Benzyl-*N*-*tert*-butyl-2-cyano-2-diazoacetamide (**57**)^70^

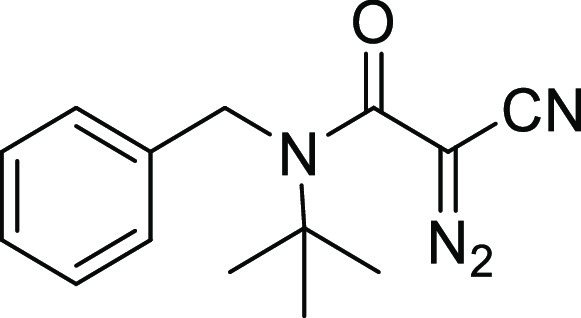



This compound was prepared according to the general flow procedure from *N*-*tert*-butyl-2-cyano-*N*-(benzyl)acetamide (**49**), triethylamine (25 mL, 0.12 M, [NEt_3_] 0.128 M), aqueous sodium azide (10 mL, 3.0 M), and a dichloromethane solution of trifluoromethanesulfonic anhydride (10 mL, 0.5 M). The crude product was purified by flash chromatography on silica gel with hexane/ethyl acetate (80:20) as eluent affording *α-diazoacetamide***57** as a yellow crystalline solid (0.599 g, 78%). Mp: 88–92 °C (lit.^70^ mp 89–91 °C). ν_max_/cm^–1^ (ATR): 2214 (CN), 2121 (CN_2_) 1634 (CO). ^1^H NMR (CDCl_3_, 300 MHz): δ 7.42–7.18 (m, 5H, 5 × Ar*H*), 4.71 (s, 2H, C*H*_*2*_N), 1.43 [s, 9H, C(C*H*_*3*_)_3_]. ^13^C{^1^H} NMR (CDCl_3_, 75.5 MHz): δ 160.9 (C), 138.4 (C), 128.7 (CH), 127.5 (C), 126.0 (CH), 109.8 (C), 60.3 (C), 49.7 (CH_2_), 28.5 (CH_3_).

#### *N*-*tert*-Butyl-2-cyano-2-diazo-*N*-(4′-fluorobenzyl)acetamide (**58**)^70^

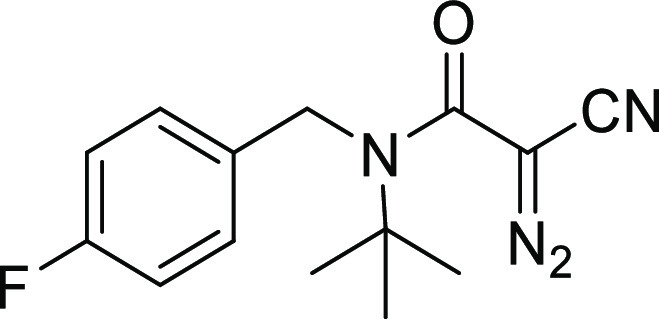



This compound was prepared according to the general flow procedure from *N*-*tert*-butyl-2-cyano-*N*-(4-fluorobenzyl)acetamide (**50**), triethylamine (25 mL, 0.12 M, [NEt_3_] 0.128 M), aqueous sodium azide (10 mL, 3.0 M), and a dichloromethane solution of trifluoromethanesulfonic anhydride (10 mL, 0.5 M). The crude product was purified by flash chromatography on silica gel with hexane/ethyl acetate (80:20) as eluent affording *α-diazoacetamide***58** as a yellow crystalline solid (0.724 g, 88%). Mp: 105–107 °C. ν_max_/cm^–1^ (ATR): 2213 (CN), 2121 (CN_2_) 1635 (CO). ^1^H NMR (CDCl_3_, 400 MHz): δ 7.22–7.13 (m, 2H, 2 × Ar*H*), 7.11–7.01 (m, 2H, 2 × Ar*H*), 4.67 (s, 2H, NC*H*_*2*_), 1.42 [s, 9H, C(C*H*_*3*_)_3_]. ^13^C{^1^H} NMR (CDCl_3_, 100.6 MHz): δ 162.0 (C, ^1^*J*_CF_ = 246.0 Hz), 160.9 (C), 134.1 (C, ^4^*J*_CF_ = 3.2 Hz), 127.6 (CH, ^3^*J*_CF_ = 8.1 Hz), 115.7 (CH, ^2^*J*_CF_ = 21.7 Hz), 109.8 (C), 60.4 (C), 49.1 (CH_2_), 28.5 (CH_3_). ^19^F{^1^H} NMR (CDCl_3_, 376.5 MHz): δ −114.9.

#### *N*-*tert*-Butyl-2-cyano-2-diazo-*N*-(4′-methylbenzyl)acetamide (**59**)^70^

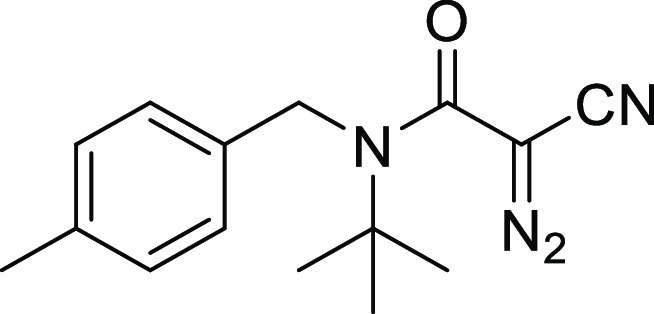



This compound was prepared according to the general flow procedure from *N*-*tert*-butyl-2-cyano-*N*-(4-methylbenzyl)acetamide (**51**) (and triethylamine (25 mL, 0.12 M, [NEt_3_] 0.128 M), aqueous sodium azide (10 mL, 3.0 M), and a dichloromethane solution of trifluoromethanesulfonic anhydride (10 mL, 0.5 M). The crude product was purified by flash chromatography on silica gel with hexane/ethyl acetate (80:20) as eluent affording *α-diazoacetamide***59** as a yellow crystalline solid (0.673 g, 83%). Mp: 86–88 °C (lit.^70^ mp 85–86 °C). ν_max_/cm^–1^ (ATR) 2213 (CN), 2117 (CN_2_), 1634 (CO). ^1^H NMR (CDCl_3_, 300 MHz): δ 7.17 [d, 2H, *J* = 8.0 Hz, C(3′)*H* and C(5′)*H*], 7.09 [d, 2H, *J* = 8.1 Hz, C(2′)*H* and C(6′)*H*], 4.67 (s, 2H, C*H*_*2*_N), 2.34 [s, 3H, C(4′)(C*H*_*3*_)], 1.42 [s, 9H, C(C*H*_*3*_)_3_]. ^13^C{^1^H} NMR (CDCl_3_, 75.5 MHz): δ 160.8 (C), 137.1 (C), 135.3 (C), 129.4 (CH), 125.9 (CH), 109.8 (C), 60.2 (C), 49.5 (CH_2_), 28.5 (CH_3_), 21.1 (CH_3_).

#### *N*-*tert*-Butyl-2-cyano-2-diazo-*N*-(4′-bromobenzyl)acetamide (**60**)^70^

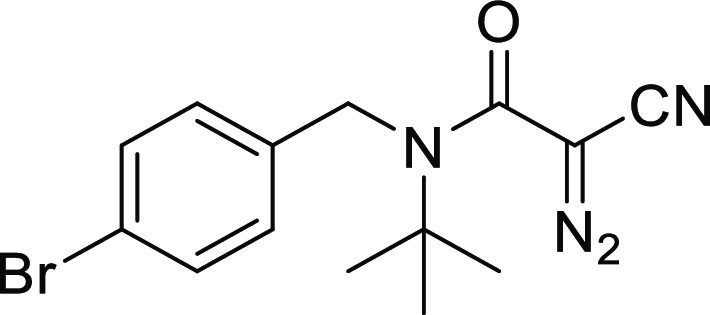



This was prepared according to the general flow procedure from *N*-*tert*-butyl-2-cyano-*N*-(4-bromobenzyl)acetamide (**52**), triethylamine (25 mL, 0.12 M, [NEt_3_] 0.128 M), aqueous sodium azide (10 mL, 3.0 M), and a dichloromethane solution of trifluoromethanesulfonic anhydride (10 mL, 0.5 M). The crude product was purified by flash chromatography on silica gel with hexane/ethyl acetate (80:20) as eluent affording *α-diazoacetamide***60** as a yellow crystalline solid (0.844 g, 84%). Mp: 109–111 °C (lit.^70^ mp 109–110 °C). ν_max_/cm^–1^ (ATR): 2213 (CN), 2119 (CN_2_), 1634 (CO). ^1^H NMR (CDCl_3_, 400 MHz): δ 7.50 [d, 2H, *J* = 8.4 Hz, C(3′)*H* and C(5′)*H*], 7.10 [d, 2H, *J* = 8.4 Hz, C(2′)*H* and C(6′)*H*], 4.65 (s, 2H, C*H*_*2*_N), 1.41 [s, 9H, C(C*H*_*3*_)_3_]. ^13^C{^1^H} NMR (CDCl_3_, 100.6 MHz): δ 160.9 (C), 137.6 (C), 131.9 (CH), 127.7 (CH), 121.3 (C), 109.7 (C), 60.4 (C), 54.8 (C), 49.3 (CH_2_), 28.5 (CH_3_).

#### *N*-*tert*-Butyl-2-cyano-2-diazo-*N*-(3′,5′-dimethylbenzyl)acetamide (**61**)

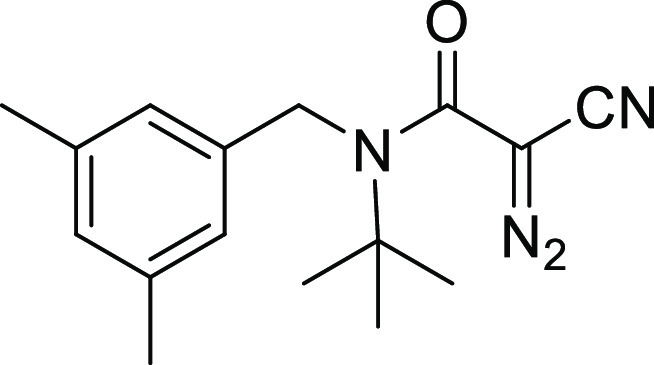



This compound was prepared according to the general flow procedure from *N*-*tert*-butyl-2-cyano-*N*-(3,5-dimethylbenzyl) acetamide (**53**), triethylamine (25 mL, 0.12 M, [NEt_3_] 0.128 M), aqueous sodium azide (10 mL, 3.0 M), and a dichloromethane solution of trifluoromethanesulfonic anhydride (10 mL, 0.5 M). The crude product was purified by flash chromatography on silica gel with hexane/ethyl acetate (80:20) as eluent affording *α-diazoacetamide***61** as a yellow crystalline solid (0.818 g, 96%). Mp: 105–107 °C. ν_max_/cm^–1^ (ATR): 2213 (CN), 2119 (CN_2_), 1634 (CO). ^1^H NMR (CDCl_3_, 300 MHz): δ 6.90 [s, 1H, C(4′)*H*], 6.79 [s, 2H, C(2′)*H* and C(6′)*H*], 4.63 (s, 2H, NC*H*_*2*_), 2.31 [s, 6H, C(3′)C*H*_*3*_ and C(5′)C*H*_*3*_], 1.42 [s, 9H, C(C*H*_*3*_)_3_]. ^13^C{^1^H} NMR (CDCl_3_, 75.5 MHz): δ 160.8 (C), 138.3 (C), 138.2 (C), 129.1 (CH), 123.8 (CH), 109.8 (C), 60.3 (C), 49.7 (CH_2_), 28.6 (CH_3_), 21.4 (CH_3_). HRMS (ESI) *m*/*z*: [M + Na]^+^ Calcd for C_16_H_20_N_4_ONa 307.1529; Found 307.1533.

#### *N*-*tert*-Butyl-2-cyano-2-diazo-*N*-(pyridin-4′-ylmethyl)acetamide (**62**)

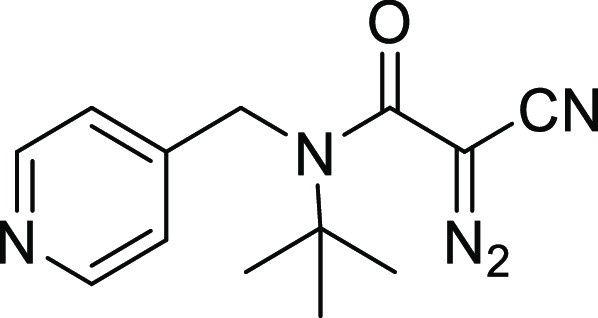



This compound was prepared according the general flow procedure from *N*-*tert*-butyl-2-cyano-*N*-(pyridin-4′-ylmethyl)acetamide (**54**), triethylamine (25 mL, 0.12 M, [NEt_3_] 0.128 M), aqueous sodium azide (10 mL, 3.0 M), and a dichloromethane solution of trifluoromethanesulfonic anhydride (10 mL, 0.5 M). The crude product was purified by flash chromatography on silica gel with hexane/ethyl acetate (80:20 to 40:60) as eluent affording *α-diazoacetamide***62** as a yellow crystalline solid (0.587 g, 76%). Mp: 88–91 °C. ν_max_/cm^–1^ (ATR): 2982, 2214, 2125, 1635. ^1^H NMR (CDCl_3_, 400 MHz): δ 8.61 [d, 2H, *J* = 6.0 Hz, C(2)*H* and C(6)*H*], 7.19 [d, 2H, *J* = 5.6 Hz, C(3)*H* and C(5)*H*], 4.70 (s, 2H, C*H*_*2*_N), 1.43 [s, 9H, C(C*H*_*3*_)_3_]. ^13^C{^1^H} NMR (CDCl_3_, 100.6 MHz): δ 161.1 (C), 150.0 (CH), 148.2 (C), 121.1 (CH), 109.5 (C), 60.6 (C), 48.9 (CH_2_), 28.5 (CH_3_). HRMS (ESI) *m*/*z*: [M + Na]^+^ Calcd for C_13_H_15_N_5_ONa 280.1169; Found 280.1169.

#### Methyl 2-((2′-Cyclohexylethyl)sulfonyl)-2-diazoacetate (**63**)^13^

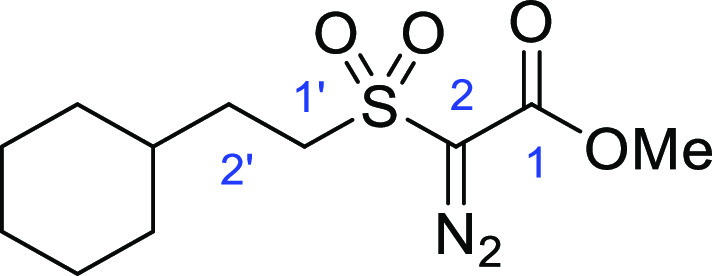



This compound was prepared according to the general flow procedure from methyl 2-((2′-cyclohexylethyl)sulfonyl)acetate **66** and DBU (25 mL, 0.12 M, [DBU] 0.128 M), aqueous sodium azide (10 mL, 3.0 M), and a dichloromethane solution of trifluoromethanesulfonic anhydride (10 mL, 0.5 M). The crude product was purified by flash chromatography on silica gel with hexane/ethyl acetate (80:20) as eluent affording *α-diazosulfonyl ester***63**. Yellow oil (0.658 g, 80%). ν_max_/cm^–1^ (ATR): 2124 (CN_2_), 1713 (CO), 1331, 1294, 1144 (SO_2_). ^1^H NMR (CDCl_3_, 300 MHz): δ 3.88 (s, 3H, C*H*_3_), 3.46–3.34 [symmetrical m, 2H, C(1′)*H*_*2*_], 1.81–1.58 [m, 7H, C(2′)*H*_*2*_ and cyclohexane ring C*H*_*2*_], 1.46–1.06 (m, 4H, cyclohexane ring C*H* and cyclohexane ring C*H*_*2*_), 1.05–0.84 (m, 2H, cyclohexane ring C*H*_*2*_). ^13^C{^1^H} NMR (CDCl_3_, 75.5 MHz): δ 160.5 (C), 72.8 (C), 54.7 (CH_2_), 53.0 (CH_3_), 36.4 (CH), 32.8 (CH_2_), 29.6 (CH_2_), 26.2 (CH_2_), 25.9 (CH_2_).

### Telescoped C–H Insertion

#### Methyl (1*R*,4a*R*,8a*S*)-Octahydro-1*H*-isothiochromene-1-carboxylate 2,2-Dioxide (**65**)^13^

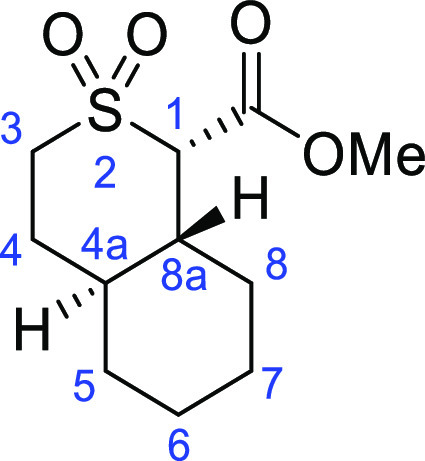



An aqueous solution of sodium azide (10 mL, 30 mmol, 3.0 M, 3.0 mL min^–1^) was pumped through a micromixer T-piece where it met a toluene solution of triflic anhydride (10 mL, 5 mmol, 0.5 M, 3.0 mL min^–1^); the combined stream passed through a reactor coil (4 × 10 mL, rt). After all reagent solutions had been charged, the combined flow rate was changed to 0.2 mL min^–1^ to give a residence time of 1 h. The reactor effluent passed through a T-piece where it met a stream of saturated aqueous sodium bicarbonate (0.1 mL min^–1^). The reaction stream was passed through a back pressure regulator (8 bar). The biphasic reactor effluent was then separated by an in-line liquid–liquid separator. The toluene layer (25 mL) was collected over KOH pellets and was directly fed to another pump. The pump delivered the separated triflic azide solution (25 mL, 0.165 mL min^–1^) to a T-piece where it met a DBU and toluene solution of **66** (25 mL, 3 mmol, 0.12 M, [DBU] 0.128 M, 0.165 mL min^–1^) which then passed through a reactor coil (4 × 10 mL, rt, 120 min residence time), and then passed through a back pressure regulator (8 bar). The reactor effluent (100 mL) was collected over KOH pellets, from which a a 25 mL portion, containing α-diazo-β-sulfonyl ester solution **63** was pumped forward to undergo C–H insertion; this 25 mL solution was passed through a silica gel plug (glass column, 100 mm × 10 mm internal diameter, rt, 0.5 mL min^–1^) to remove polar components. Immediately after eluting from the silica gel plug, the effluent was passed through a packed bed reactor containing IPB catalyst **9** (0.313 g, 10 mol % based on **66**, glass column, 150 × 66 mm internal diameter, 0.5 mL min^–1^, 111 °C). The reactor effluent was analyzed by IR spectroscopy and concentrated under reduced pressure. The crude product mixture was purified by flash chromatography on silica gel, employing ethyl acetate/hexane (10:90 to 40:60) as eluent and afforded methyl (1*R*,4a*R*,8a*S*)-octahydro-1*H*-isothiochromene-1-carboxylate 2,2-dioxide (**65**) as a white solid (47 mg, 26%, 88% ee (determined by chiral phase HPLC)). Mp: 123–125 °C. ν_max_/cm^–1^(ATR): 1731 (CO), 1315, 1288, 1229, 1167, 1109 (SO_2_). ^1^H NMR (CDCl_3_, 300 MHz): δ 3.80 (s, 3H, C*H*_*3*_), 3.77–3.58 [m containing dd at 3.75, 2H, *J* = 4.7 Hz, 3.1 Hz, C(1)*H* and *H*_*B*_ of C(3)*H*_2_], 2.96 (overlapping dddd, 1H, *J* = 14.0 Hz, 3.2 Hz, *H*_*A*_ of C(3)*H*_2_), 2.14–1.62 (m, 8H, C(4)*H*_*2*_, C(4a)*H*, C(8a)*H* and cyclohexane ring C*H*_*2*_), 1.36–1.14 (m, 2H, cyclohexane ring C*H*_*2*_), 1.11–0.92 (m, 2H, cyclohexane ring C*H*_*2*_). ^13^C{^1^H} NMR (CDCl_3_, 75.5 MHz): δ 166.9 (C), 68.9 (CH), 52.8 (CH_3_), 48.5 (CH_2_), 43.0 (CH), 33.6 (CH), 32.9 (CH_2_), 31.1 (CH_2_), 30.9 (CH_2_), 25.5 (CH_2_), 25.4 (CH_2_).

## Supporting Information Available

Details of continuous flow platforms; determination of reactant ratios for use of in situ generated triflyl azide; supplementary general flow procedure for telescoped generation of TfN_3_**22** and direct use for diazo transfer; safety considerations; chiral stationary phase HPLC of PTAD adduct **10** and thiopyran **65**; ^1^H NMR determination of enantiopurity of azulenone **31** using a chiral shift reagent; copies of ^1^H, ^13^C{^1^H} and ^19^F{^1^H} NMR spectra (PDF)
