# Supplementary material for: Exploiting Continuous Processing for Challenging Diazo Transfer and Telescoped Copper-Catalyzed Asymmetric Transformations
Source: J Org Chem. 2021 Aug 11;86(20):13955–82. doi: 10.1021/acs.joc.1c01310 (PMC8524431; doi:10.1021/acs.joc.1c01310)
Supplement: Supplementary file 1 — jo1c01310_si_001.pdf [file jo1c01310_si_001.pdf]

Supporting Information

Exploiting Continuous Processing for Challenging Diazo Transfer and Telescoped Copper-Catalyzed Asymmetric Transformations

Daniel C. Crowley[†], Thomas A. Brouder[†], Aoife M. Kearney[†], Denis Lynch[‡], Alan Ford[†], Stuart G. Collins[‡] and Anita R. Maguire[§]*

[†] *School of Chemistry, Analytical and Biological Chemistry Research Facility, University College Cork, Ireland.*
[‡] *School of Chemistry, Analytical and Biological Chemistry Research Facility, Synthesis and Solid State Pharmaceutical Centre, University College Cork, Ireland.*
[§] *School of Chemistry and School of Pharmacy, Analytical and Biological Chemistry Research Facility, Synthesis and Solid State Pharmaceutical Centre, University College Cork, Ireland.*

**E-mail:* a.maguire@ucc.ie

Table of Contents

Details of Continuous Flow Platforms S2

Determination of Reactant Ratios for Use of In Situ Generated Triflyl Azide S4

Supplementary General Flow Procedure for Telescoped Generation of TfN_3 **22** and Direct Use for Diazo Transfer S6

Safety Considerations S7

Chiral Stationary Phase HPLC of PTAD Adduct **10** and Thiopyran **65** S11

^{1}H NMR Determination of Enantiopurity of Azulenone **31** using a Chiral Shift Reagent S13

Copies of ^{1}H, ^{13}C{^{1}H} and ^{19}F{^{1}H} NMR Spectra S15

References S57

Details of Continuous Flow Platforms

Continuous processes were performed using a Vapourtec R-Series flow system consisting of four piston (HPLC) pumps and a Vapourtec E-Series flow system consisting of three peristaltic pumps. Where a solid phase reagent/reaction component was required at a specific temperature, a glass reactor manifold containing a temperature controlled glass Omnifit® column was employed. Where peristaltic pumps were used, the Vapourtec 'red' peristaltic tubing was used for aqueous solutions or toluene solutions and the Vapourtec 'blue' peristaltic tubing was used for dichloromethane or toluene solutions. In our experience, pumping of the triflic anhydride solutions used during this work was associated with reduced lifetime of the 'red' peristaltic tubing (toluene solutions) and 'blue' peristaltic tubing (dichloromethane or toluene solutions). Where a micromixer reactor was incorporated in a continuous process, a 200 µl LTF-MX micromixer reactor was used.

Table S1. General specifications for Vapourtec R-Series system

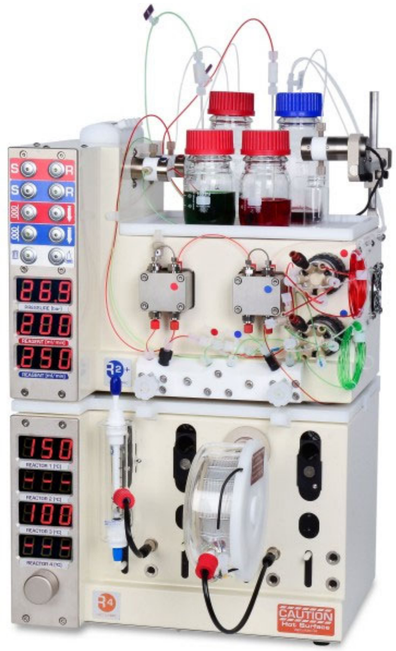

General specifications for continuous-flow system	
Material of tubing	PFA
Diameter of tubing	1 mm
Working flow rates	0.05 mL/min – 9.99 mL/min
Tubular reactor working volume	10 mL
Temperature range	–70 ºC to 250 ºC

Table S2. General specifications for Vapourtec E-Series system

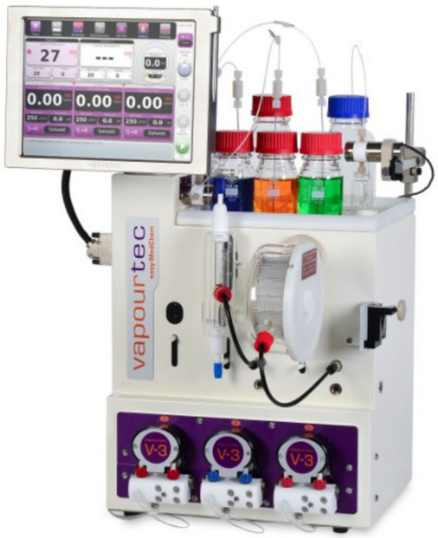

General specifications for continuous-flow system	
Material of tubing	PFA
Diameter of tubing	1 mm
Working flow rates	0.02 mL/min – 10.0 mL/min
Tubular reactor working volume	10 mL
Temperature range	–70 ºC to 250 ºC

Determination of Reactant Ratios for Use of In Situ Generated Triflyl Azide

The ratio of in situ generated triflyl azide (**22**) to diazo acceptor required for complete diazo transfer, as part of a telescoped process, was determined by diazo transfer to a strong diazo acceptor, sodium acetylacetonate **6**, on flow. An aqueous solution of sodium azide was pumped to a T-piece where it met a dichloromethane solution of triflic anhydride and the combined biphasic stream passed through a reactor coil (4 ×10 mL, at room temperature). After all reagent solutions had been charged, the combined flow rate was lowered from 3 mL min^{-1} to 0.2 mL min^{-1} to give a residence time of 1 h. The reactor effluent then met a stream of saturated aqueous sodium bicarbonate (0.1 mL min^{-1}) and the combined stream was passed through a back-pressure regulator (8 bar). The biphasic effluent was then separated by an in-line liquid–liquid separator (Scheme S1).

Scheme S1. Generation of triflyl azide (22) on flow and diazo transfer to sodium acetylacetonate (6)

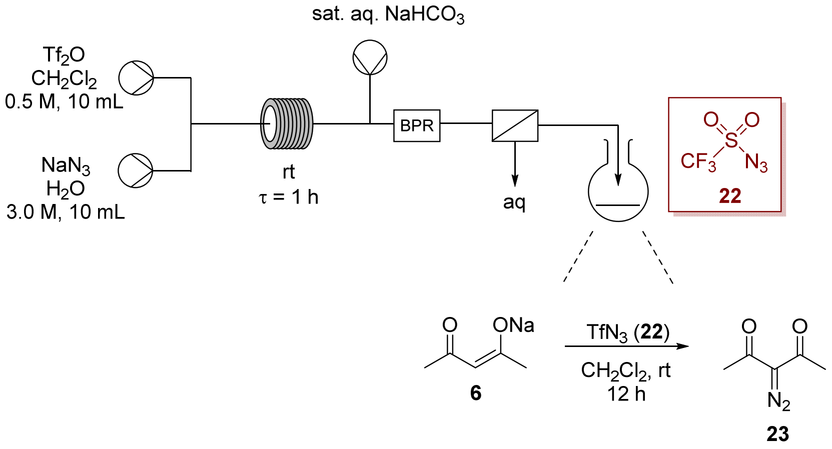

The reagent solutions were charged at 3 mL min^{-1}due to the labile nature of triflic anhydride; it is readily hydrolyzed to triflic acid upon contact with adventitious moisture. In order to further minimize its exposure to ambient atmospheric conditions, the preparation of the dichloromethane solution of triflic anhydride was undertaken immediately prior to commencing the reaction. As was noted for the triflyl azide solution it is also critical that an appropriate model of pump is used to transfer the triflic anhydride solution, with a peristaltic pump used for this work; in our experience, HPLC pumps are not effective for this operation.

The organic stream was collected in a round bottom flask which contained a known excess of sodium acetylacetonate hydrate **6** to determine the efficiency of formation of triflyl azide indirectly, by measuring the extent of diazo transfer to the β-diketone, without having to isolate this hazardous material. As the in-line liquid–liquid separator effluent flowed into the round bottom flask, the white suspension slowly turned yellow; a strong indication of α-diazo-β-diketone formation. Once all the effluent had been collected, the resulting mixture in the round bottom flask was stirred at room temperature overnight. The reaction mixture was concentrated under reduced pressure (**N.B.** IR analysis was used to check for residual sulfonyl azide prior to concentration) and purified by chromatography. The α-diazo-β-diketone **23** was isolated in 60% yield. The procedure was repeated under identical conditions to ensure the process could be replicated and α-diazo-β-diketone **23** was isolated, a second time, in 65% yield. From these experiments it was indicated that triflyl azide was efficiently formed by our continuous flow methodology to at least 58.5% conversion (based on reaction with a known excess of sodium acetylacetonate **6**).

Supplementary General Flow Procedure for Telescoped Generation of TfN_3 22 and Direct Use for Diazo Transfer

An aqueous solution of sodium azide (Pump B: 10 mL, 3.0 M, 10 eq., 3.0 mL min^{-1}) was pumped through a micromixer T-piece where it met a dichloromethane solution of trifluoromethanesulfonic anhydride (Pump A: 10 mL, 0.5 M, 1.67 eq., 3.0 mL min^{-1}); the combined stream passed through a reactor coil (4 × 10 mL, rt). After all reagent solutions had been charged, the combined flow rate was changed to 0.2 mL min^{-1} to give a residence time of 1 h. The reactor effluent passed through a T-piece where it met a stream of saturated aqueous sodium bicarbonate (Pump C: 0.1 mL min^{-1}). The reaction stream was passed through a back-pressure regulator (8 bar). The biphasic effluent was then separated by an in-line liquid–liquid separator. The organic effluent (25 mL) was directly fed to Pump D; once Pump D began pumping (25 mL, 0.17 mL min^{-1}), Pump E began pumping a solution of the relevant acceptor substrate (25 mL, 0.12 M, 1 eq., [Base] 0.128 M, 1.1 eq., 0.17 mL min^{-1}). Pumps D and E delivered their respective solutions to a T-piece after which the combined reaction stream was then passed through a reactor coil (4 × 10 mL, rt, τ = 2 h) and subsequently through a glass column packed with silica gel (100 mm × 10 mm internal diameter) and then finally passed through a back pressure regulator (8 bar). The reactor effluent was analysed by IR spectroscopy and concentrated under reduced pressure affording the crude α-diazoketone which was subsequently purified by flash chromatography on silica gel with hexane:ethyl acetate as eluent.

Scheme S2. Telescoped generation and use of triflyl azide (22) for diazo transfer, with pumps indicated as A–E.

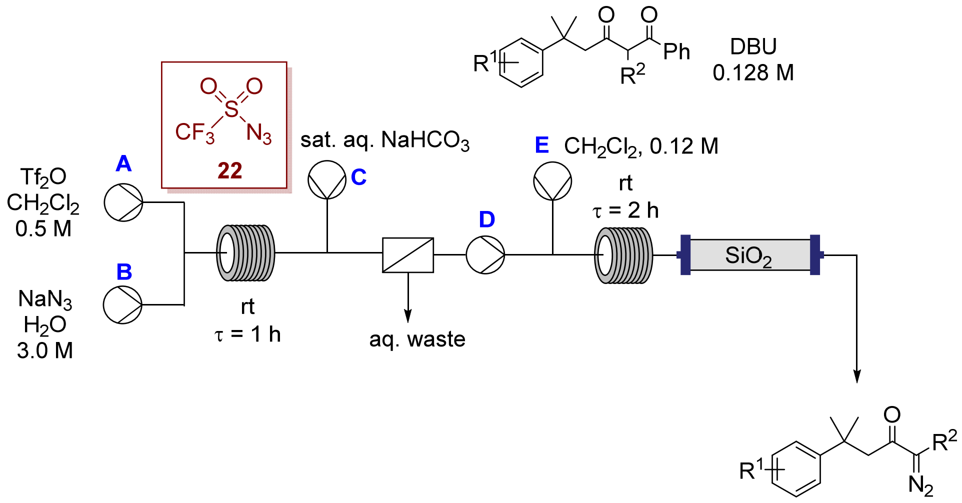

Safety Considerations

In Situ IR Study

When generating triflyl azide (**22**) from triflic anhydride in dichloromethane and aqueous sodium azide it is noteworthy that, over prolonged periods of time, the azide anion can displace chloride. There are reported instances of diazidomethane being formed when sodium azide and dichloromethane are heated in solution or upon prolonged storage together for weeks at room temperature.[1,2] In the batch process described herein, the aqueous sodium azide solution and dichloromethane are in contact for just 2 hours. There are no reported instances of diazidomethane forming at room temperature in this timeframe and there are multiple examples in the literature of sodium azide and dichloromethane being safely used together under similar conditions.[3-5] The batch approach to utilizing TfN_3 **22** involves a phase split,[3] after generation of the sulfonyl azide and prior to diazo transfer; by removing the aqueous stream and, with it, excess sodium azide, at the earliest possible stage, the possibility of the sodium azide reacting with dichloromethane to form diazidomethane was greatly reduced. In the methodology used in this work, with the in-line liquid–liquid separation occurring after 1 hour, the risk of diazidomethane forming was considered negligible.

In order to confirm that formation of diazidomethane was not a risk in the reaction timeframe, a mixture of dichloromethane (15 mL) and aqueous sodium azide (2.1 M, 15 mL) was stirred at room temperature in a Schlenk tube for 4 hours. The dichloromethane layer was monitored continuously with an in situ IR probe. A Mettler–Toledo ReactIR 15 system using a DST (6.35 × 2.0 m × 305) SiComp probe fitted into the reaction vessel was used to monitor experiments for this purpose, with the data collected and processed using iC IR 7.0 software.

Specific attention was paid to any absorptions that might be attributed to dissolved azide (inorganic azide N_3^- ν_{max} 2050 cm^{-1}) or diazidomethane (CN_2 ν_{max} 2100 cm^{-1}).[5] The first image is the overall IR spectrum observed for the 4 hours; as can be seen, only minor absorptions are noted in the distinctive azide region (Figure 2, Image 1). The second image depicts an expansion of the range 1900–2500 cm^{-1} (Figure 2, Image 2), the absorption observed at ~2300 cm^{-1} is associated with dichloromethane and its intensity remained unchanged throughout the experiment.[6] Another absorption (2050 cm^{-1}) was first observed distinctly at around 2 hours, and very slowly increased over the course of the experiment; this indicated that trace amounts

of azide were slowly appearing in the organic layer. It was noted, however, that it was one of the weakest absorptions in the spectrum.

To attempt to quantify the dissolved azide being observed, the reaction mixture was spiked with 4-dodecylbenzenesulfonyl azide (*p*-DBSA) (30 mg, 0.085 mmol) whose azide stretch (~2120 cm^{-1}) is observed in the third image (Figure 2, Image 3). *p*-DBSA was selected as the reference azide source due to the low probability of partitioning into the aqueous layer, when only the dichloromethane phase was monitored during the study. The absorption peak observed for this spike of *p*-DBSA was closely similar in intensity to the peak observed at 2050 cm^{-1}, enabling approximation of the quantity of the dissolved azide to be of the same order as *p*-DBSA (0.0056 M) at this point. Two further portions of were added (45 mg and 35 mg, respectively) and the increase in intensity of the corresponding azide stretch at ~2120 cm^{-1} was observed. Over a four-hour period, the above IR study showed that no evidence of appreciable formation of diazidomethane was detected at room temperature, consistent with literature reports that detailed the conditions required for formation and detonation of this compound.

Throughout the extent of this work, the length of contact time between dichloromethane and sodium azide was limited to 1 hour at room temperature on flow and 2 hours at 0 °C in batch in accordance with literature procedure.[3] It can also be noted that while use of dichloromethane is employed for most of the subsequent enantioselective transition metal catalysis undertaken as part of this work, the flow procedure to generate TfN_3 for diazo transfer has also been safely performed in toluene, which avoids any risk associated with potential formation of diazidomethane.

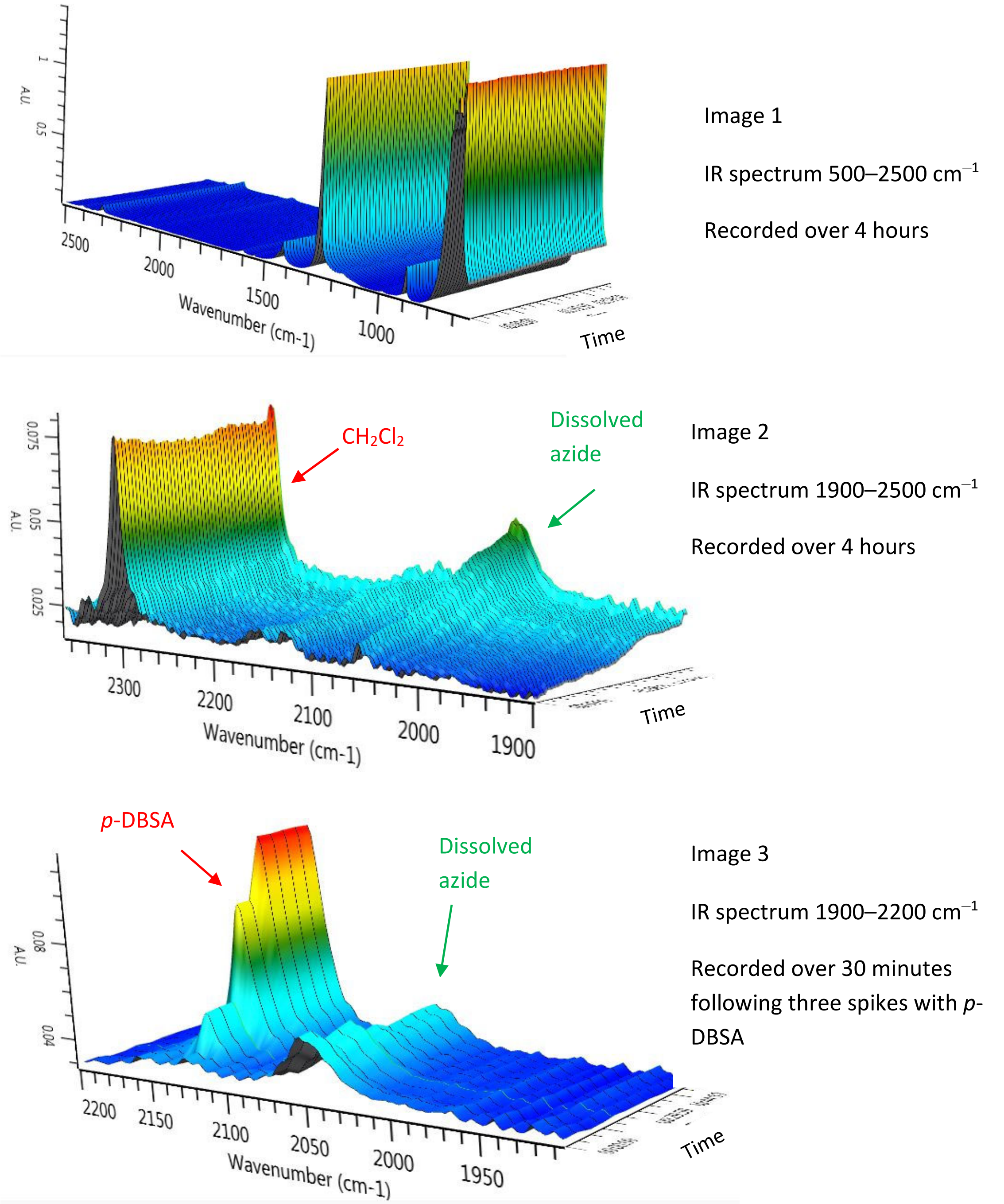

Figure S1. IR spectra recorded continuously for four hours at room temperature (Image 1 and 2) and for a further 30 minutes showing three separate spikes with samples of *p*-DBSA (Image 3).

Disposal of Aqueous Effluents from Telescoped Generation of Triflyl Azide

In order to demonstrate the safe disposal of the aqueous effluents from the continuous generation of triflyl azide (**22**), the process was undertaken on a 3.0 mmol scale and the aqueous layer, upon separation using the in-line liquid–liquid separator was collected and was pumped to T-piece where it met a stream of aqueous sodium nitrite and the combined stream was passed through a reactor coil (2 × 10 mL, rt, 5 min total residence time) after which the effluent passed through a back pressure regulator and was collected in flask containing excess aqueous sulfuric acid. This process destroyed the unreacted sodium azide affording gaseous N_2 and NO. The evolution of brown NO gas was observed as the reactor effluent was collected in the flask. After the effluent was collected, the aqueous solution was checked with pH paper, which indicated that it was acidic and that all unreacted azide had been destroyed.[7]

Scheme S3. Generation of triflyl azide (22) on flow including disposal of separated aqueous effluents using continuous processing

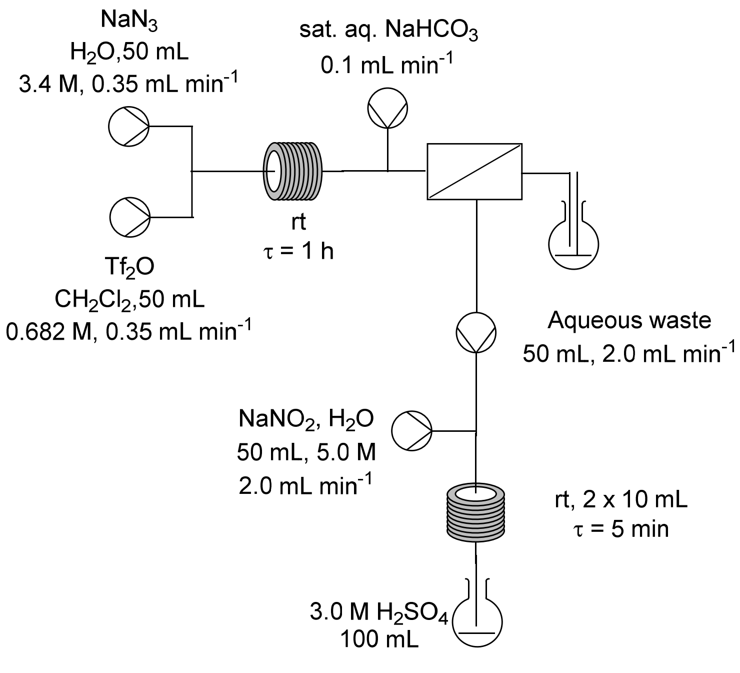

Chiral Stationary Phase HPLC of PTAD Adduct 10 and Thiopyran 65

Resolution of the *PTAD adduct* **10** (injection volume:25 μL) was achieved using a Chiracel® OD-H column at room temperature, with isopropanol:hexane (10:90) as eluent, a flow rate of 0.5 mL/min, and the detector set at λ 219 nm. Under these conditions, the dextrorotatory (+) enantiomer elutes at 19.0 min and the levorotatory (–) enantiomer elutes at 21.6 min. All samples were made in IPA at a concentration of 1 mg mL^{-1}.

1,2,3b,4-Tetrahydro-1,1,3a,4,11,12-hexamethyl-7-phenyl-4,10-etheno-6H,10H-cyclopenta[1,3]cyclopropa[1,2-d][1,2,4]triazolo[1,2-a]pyridazine-3,6,8(3a*H*,7*H*)-trione (10)

.

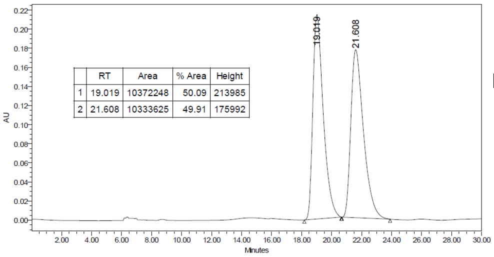

	RT	Area	% Area	Height
1	19.019	10372248	50.09	213985
2	21.608	10333625	49.91	175992

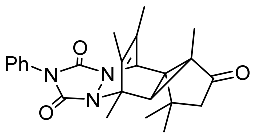

Figure S2. Reaction catalysed with $Rh_2(OAc)_4$ in batch.

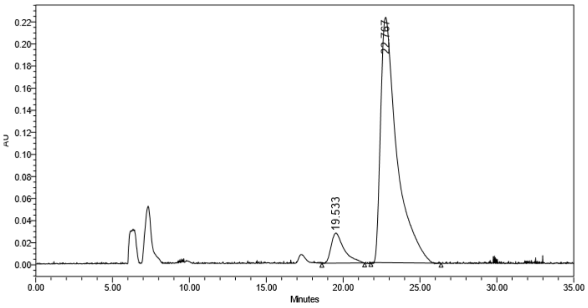

	RT	Area	% Area	Height
1	19.533	1582554	8.82	27213
2	22.767	16367046	91.18	222082

Figure S3. Telescoped generation of triflyl azide (**22**), debenzoylative diazo transfer and aromatic addition of α-diazoketone **8** with IPB **9** in flow. $[\alpha]_D^{20}$ –64.38 [c 0.080, $CHCl_3$, 82% ee].

Resolution of the *thiopyran* **65** (injection volume:25 µL) was achieved using a Phenomenex Lux® 3µm Amylose-1 column at 25 °C, with isopropanol:hexane (10:90) as eluent, a flow rate of 0.5 mL/min, and the detector set at λ 231 nm. Under these conditions, the levorotatory (–) enantiomer elutes at 12.3 min and the dextrorotatory (+) enantiomer elutes at 15.4 min. All samples were made in IPA at a concentration of 1 mg mL^{-1}.

Methyl (1*R*,4a*R*,8a*S*)-octahydro-1H-isothiochromene-1-carboxylate 2,2-dioxide (65)

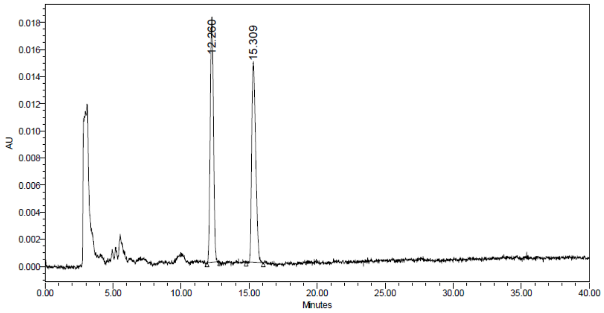

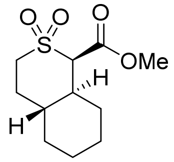

	RT	Area	% Area	Height
1	12.260	301687	49.98	18081
2	15.309	301952	50.02	14696

Figure S4. Reaction catalysed with $Rh_2(OAc)_4$ in batch.

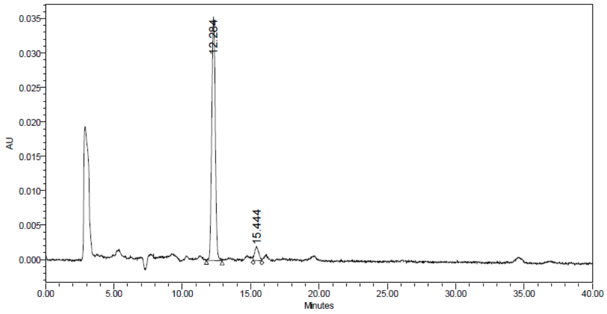

	RT	Area	% Area	Height
1	12.284	635898	93.89	35249
2	15.444	41402	6.11	1964

Figure S5. Telescoped generation of triflyl azide (**22**), Regitz-type diazo transfer and C–H insertion of α-diazo-β-ketosulfone **63** with IPB **9** in flow.

^{1}H NMR Determination of Enantiopurity of Azulenone 31 using a Chiral Shift Reagent

3,8a-Dihydro-3,3,8a-trimethylazulen-1(2*H*)-one 31

Reaction catalysed with IPB ***9*** *on flow.*

^{1}H NMR spectra run at 300 K; 20 mg of azulenone in 0.5 mL $CDCl_3$.

Figure S6. Expansion of signals with varying amounts of (+)-Eu(hfc)$_3$ added to 20 mg of azulenone **31** in 0.5 mL of $CDCl_3$.

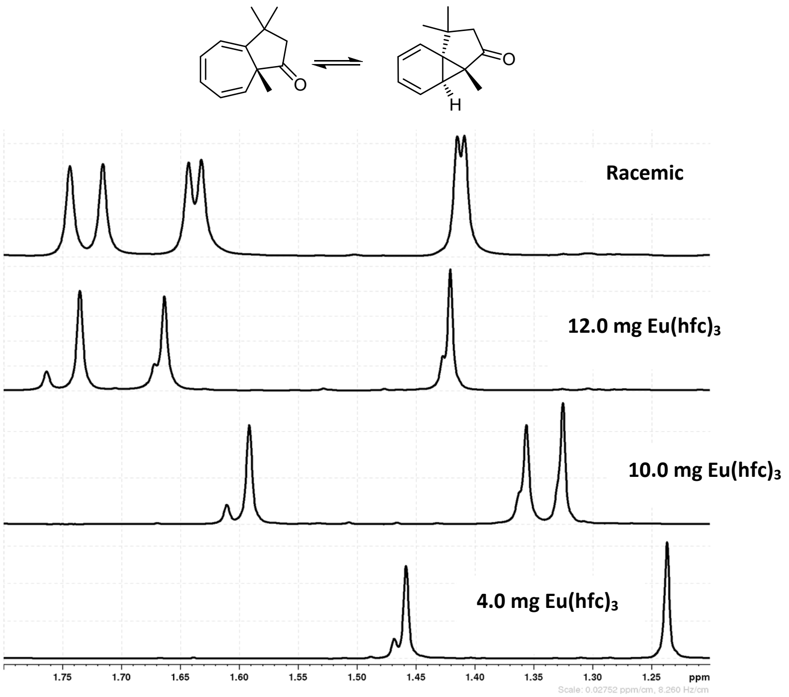

Table 2. Position of signals with varying amounts of (+)-Eu(hfc)$_3$ added to 20 mg of azulenone 31 in 0.5 mL of $CDCl_3$

Eu(hfc)$_3$	C(8a)CH_3	C(3)CH_3	C(8a)CH_3
4.0 mg	1.07, s	1.24, s	1.46, s
			1.47, s
10.0 mg	1.39, s	1.57, s	1.69, s
			1.72, s
12.0 mg	1.42, s	1.66, s	**1.74,s** [a]
		1.67, s	**1.76,s** [b]
10.5 mg	1.41, s	1.63, s	1.72, s
(Racemic)		1.64, s	1.74, s

[a] Signal due to dextrorotatory (+) enantiomer

[b] Signal due to levorotatory (–) enantiomer

(+) Enantiomer of azulenone: $[\alpha]_D^{20}$ +15.67 [*c* 0.450, $CHCl_3$, 62% ee]

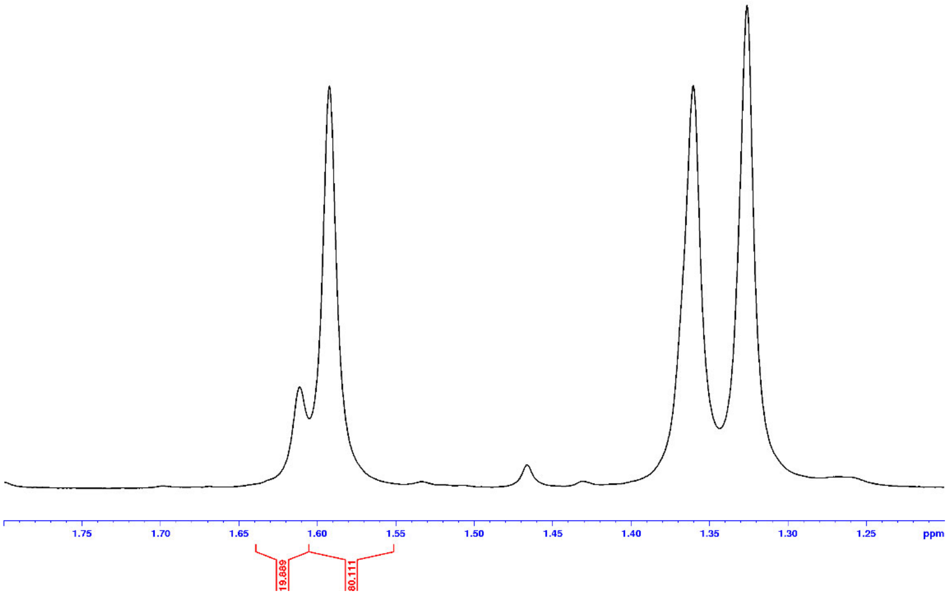

Figure S7. Telescoped generation of triflyl azide (**22**), debenzoylative diazo transfer and aromatic addition of α-diazoketone **11** with IPB **9** in flow; 13.3 mg of (+)-Eu(hfc)3 added to 20 mg of azulenone **31** in 0.5 mL of CDCl3. $[\alpha]_D^{20}$ +13.86 (c. 1.05, $CHCl_3$, 60% ee).

Copies of ^{1}H, $^{13}C\{^{1}H\}$ and $^{19}F\{^{1}H\}$ NMR Spectra

NMR spectra of the following compounds were in agreement with those previously reported:

2-diazo-5-methyl-5-(3',4',5'-methylphenyl)hexan-3-one (**8**),[8]

2-diazo-5-methyl-5-phenylhexan-3-one (**11**),[8]

2-diazo-5-methyl-5-(4-chlorophenyl)hexan-3-one (**12**),[8]

2-diazo-5-methyl-5-(4-fluorophenyl)hexan-3-one (**13**),[8]

3,8a-dihydro-3,3,8a-trimethylazulen-1(2*H*)-one (**31**),[8]

1,2,3b,4-tetrahydro-1,1,3a,4,11,12-hexamethyl-7-phenyl-4,10-etheno-6H,10H-cyclopenta[1,3]cyclopropa[1,2-d][1,2,4]triazolo[1,2-a]pyridazine-3,6,8(3a*H*,7*H*)-trione (**10**),[8]

methyl 2-((2-cyclohexylethyl)sulfonyl)-2-diazoacetate (**63**),[9] and

methyl (1*R**,4a*R**,8a*S**)-octahydro-1H-isothiochromene-1-carboxylate 2,2-dioxide (**65**)[9]

1'-(1H-Benzo[d][1,2,3]triazol-1-yl)-3'-methyl-3'-phenylbutan-1'-one[10]

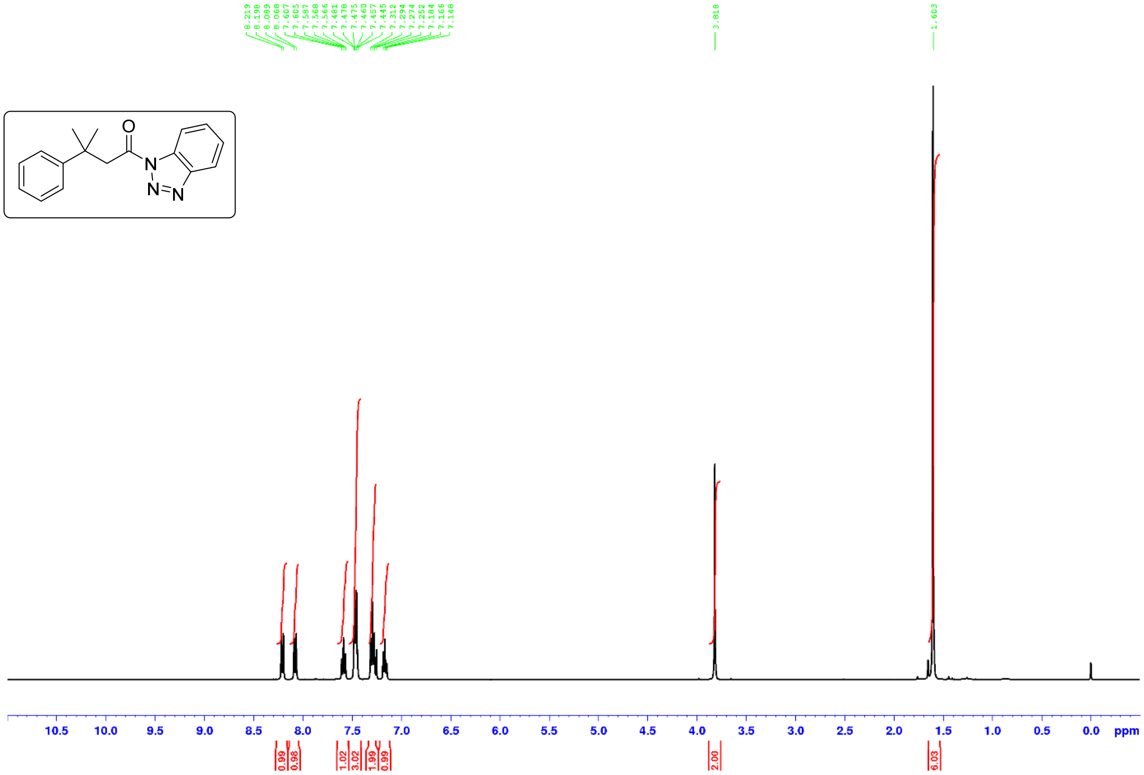

Figure S8. ^{1}H NMR ($CDCl_3$, 400 MHz) spectrum.

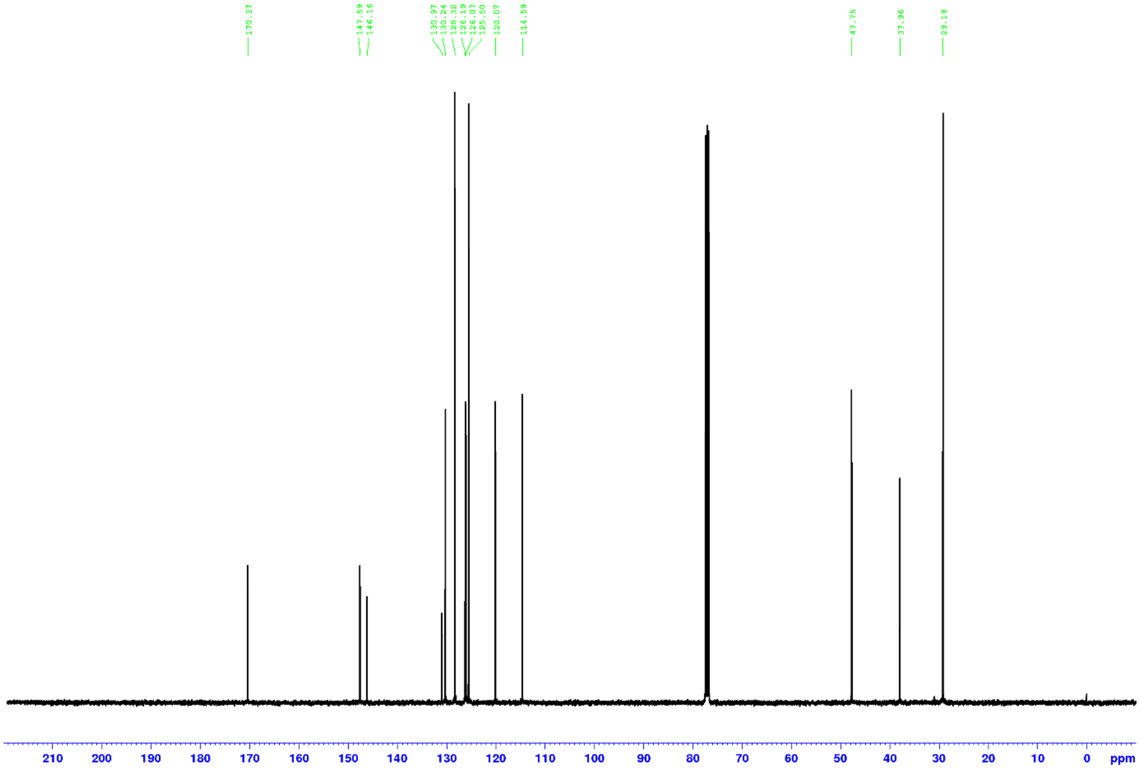

Figure S9. $^{13}C\{^{1}H\}$ NMR ($CDCl_3$, 100.6 MHz) spectrum.

1'-(1H-Benzo[d][1,2,3]triazol-1-yl)-3'-methyl-3'-(3'',4'',5''-trimethylphenyl)-butan-1'-one

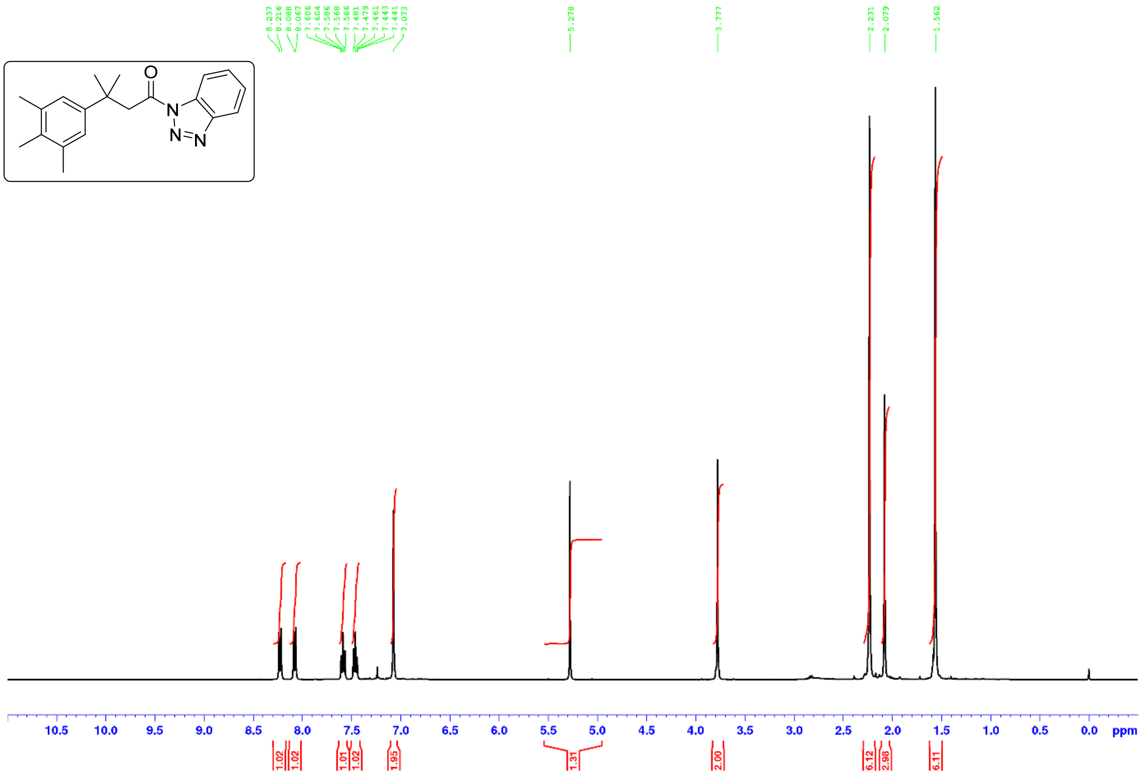

Figure S10. ^{1}H NMR ($CDCl_3$, 400 MHz) spectrum (contains a signal at δ 5.30 for dichloromethane).

1'-(1H-Benzo[d][1,2,3]triazol-1-yl)-3'-methyl-3'-(4''-chlorophenyl)butan-1'-one

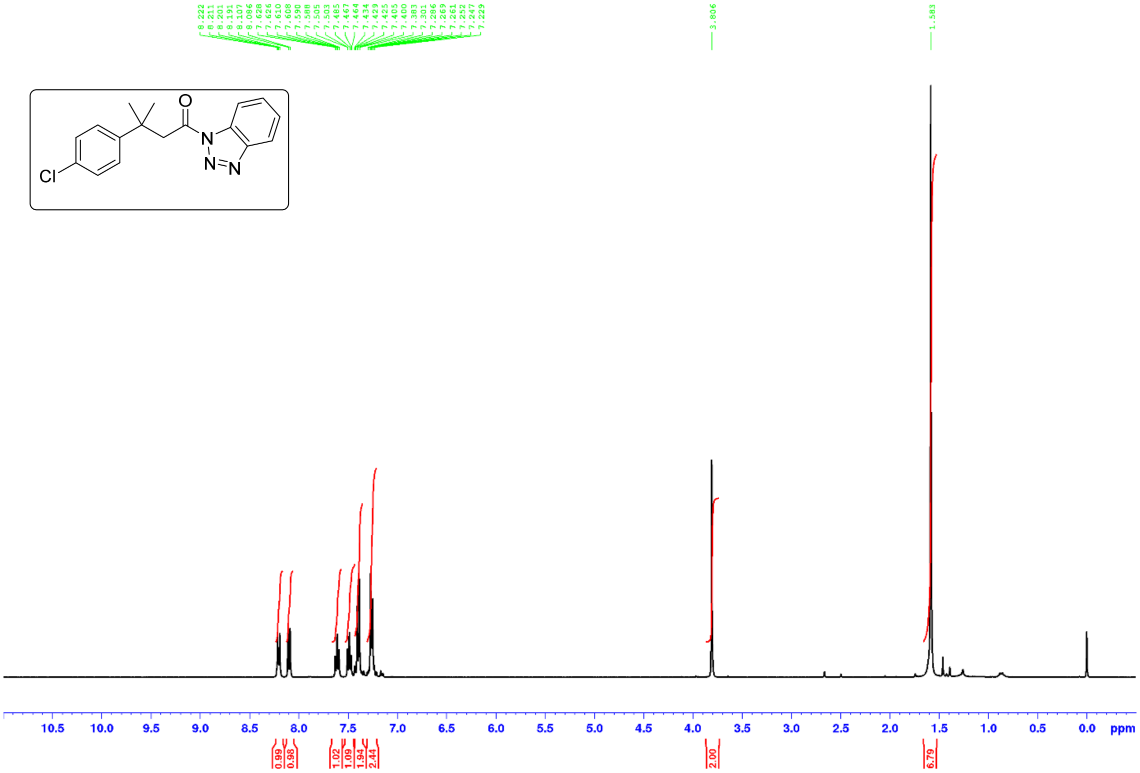

Figure S11. ^{1}H NMR ($CDCl_3$, 400 MHz) spectrum.

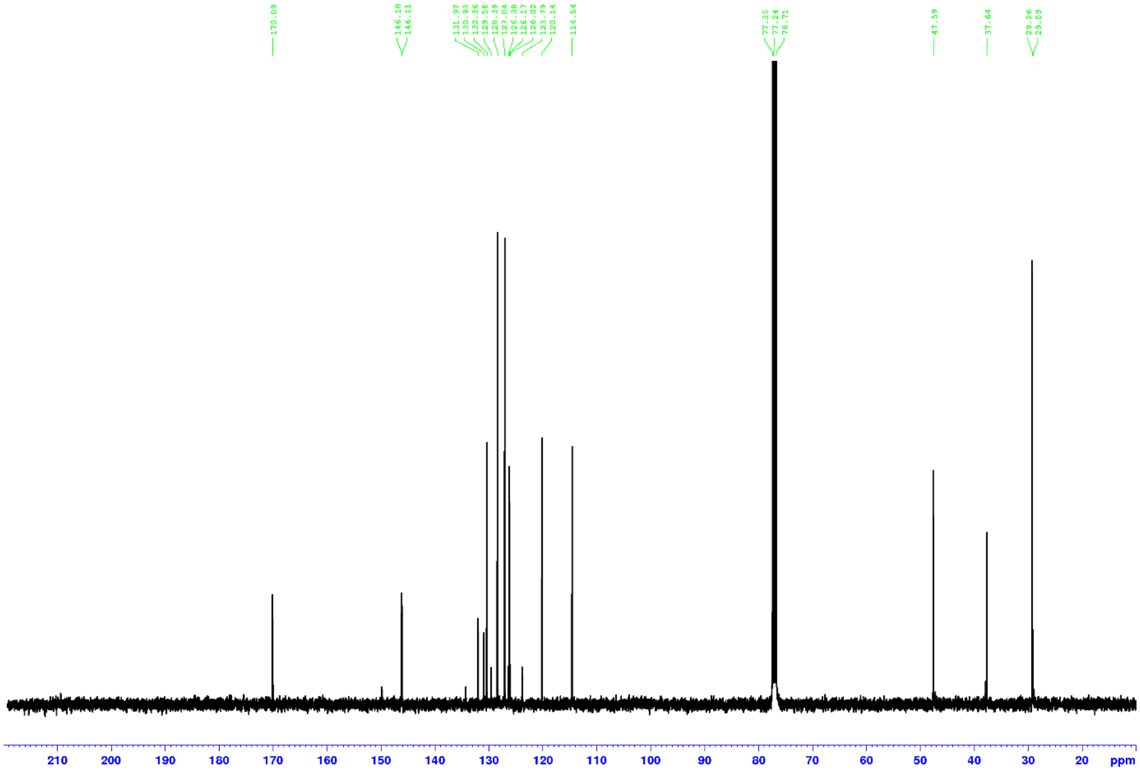

Figure S12. $^{13}C\{^{1}H\}$ NMR ($CDCl_3$, 100.6 MHz) spectrum.

1'-(1H-Benzo[d][1,2,3]triazol-1-yl)-3'-methyl-3'-(4''-fluorophenyl)butan-1'-one

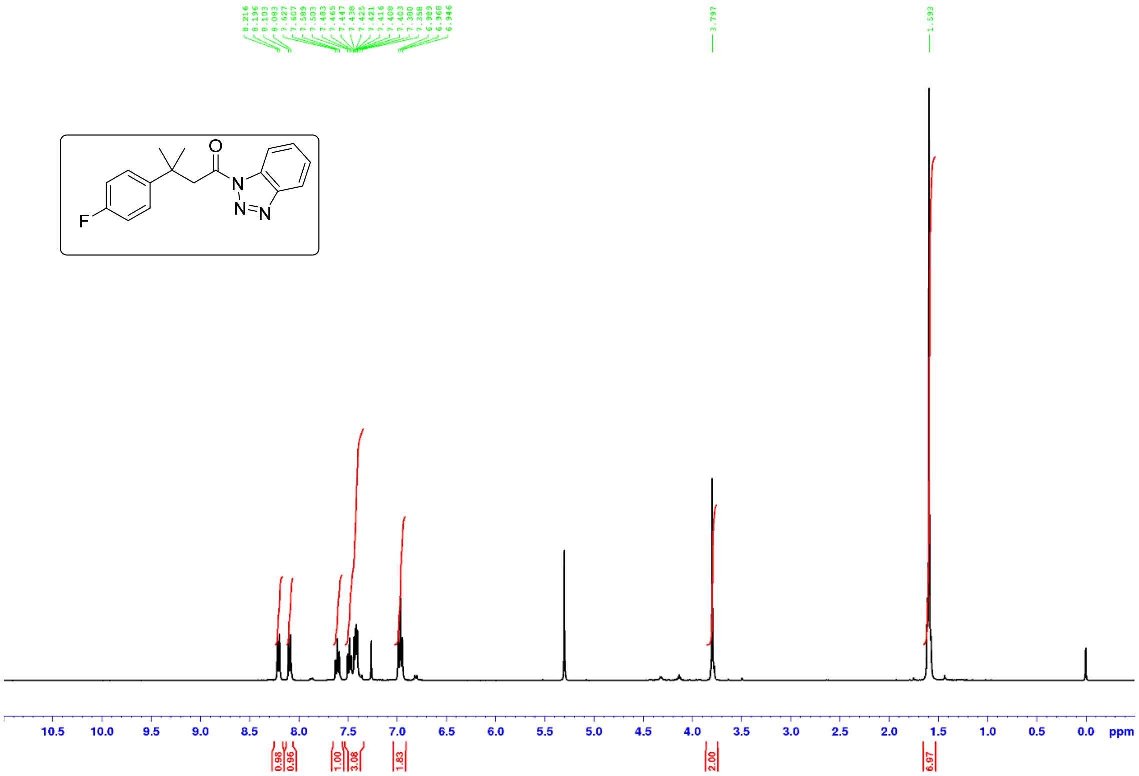

Figure S13. ^{1}H NMR ($CDCl_3$, 400 MHz) spectrum (contains a signal at δ 5.30 for dichloromethane).

2,5-Dimethyl-1-phenyl-5-(3',4',5'-trimethylphenyl)hexane-1,3-dione (18)

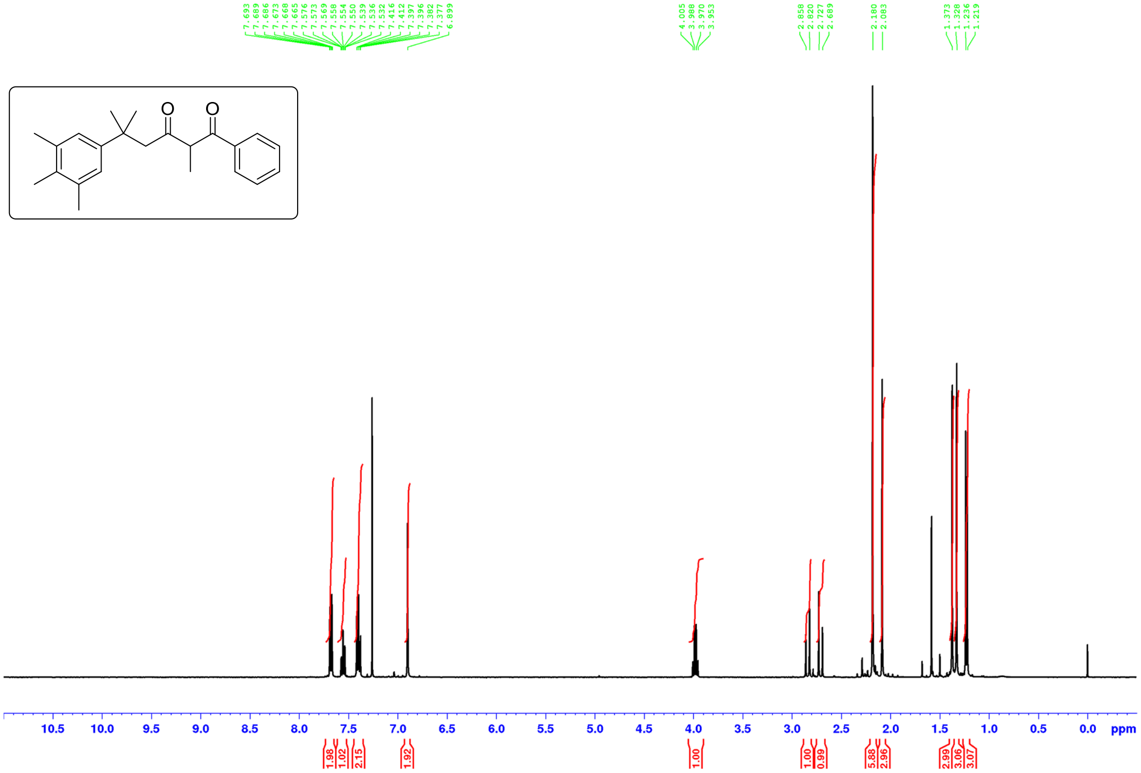

Figure S14. ^{1}H NMR ($CDCl_3$, 400 MHz) spectrum of 1,3-diketone **18**.

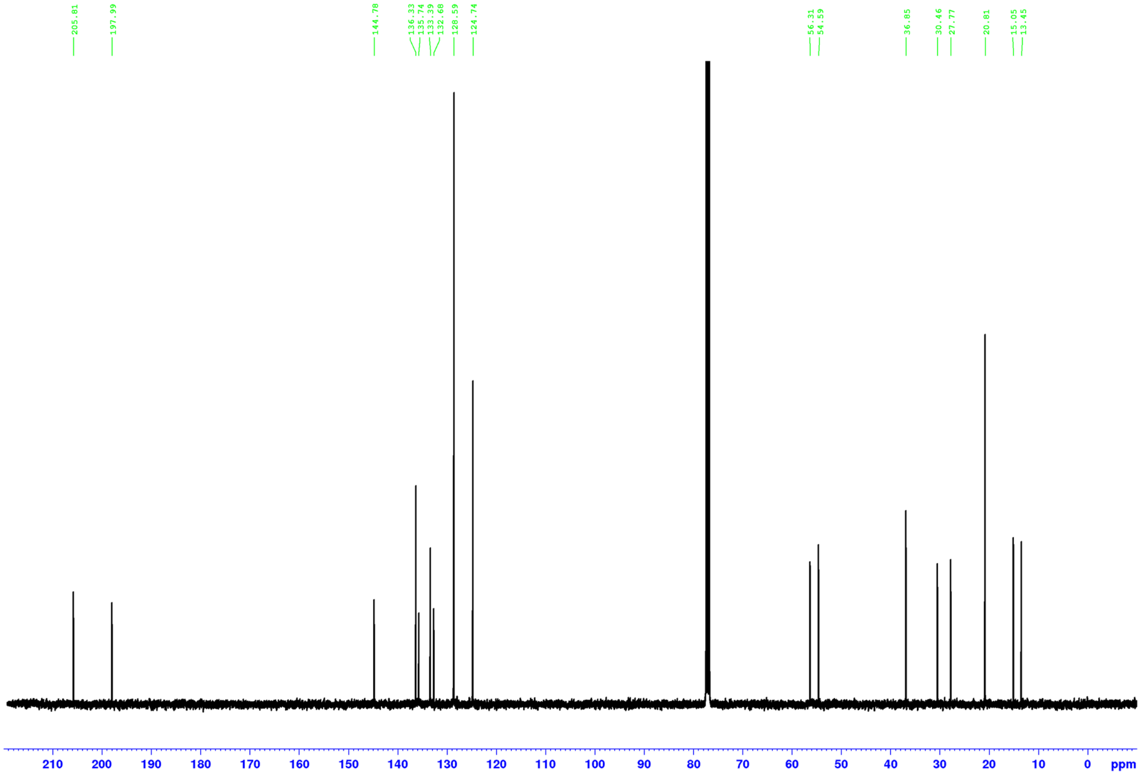

Figure S15. $^{13}C\{^{1}H\}$ NMR ($CDCl_3$, 100.6 MHz) spectrum of 1,3-diketone **18**.

2,5-Dimethyl-1,5-diphenylhexane-1,3-dione (19)[11]

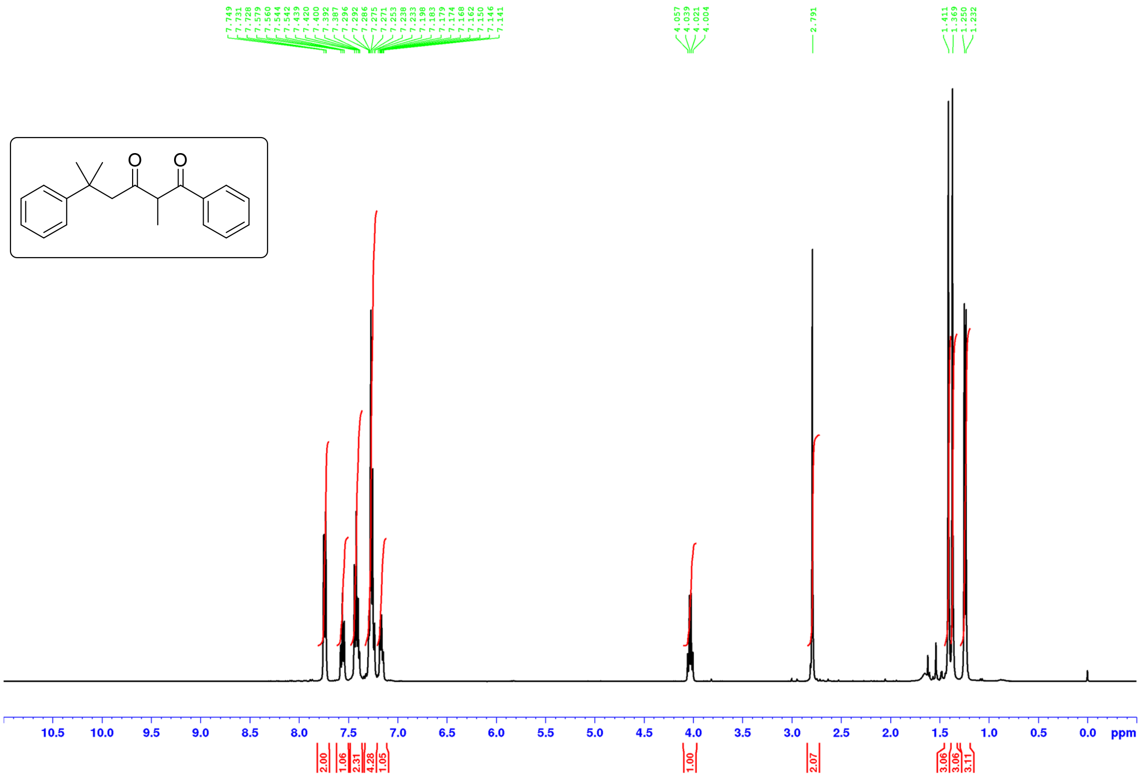

Figure S16. ^{1}H NMR ($CDCl_3$, 400 MHz) spectrum of 1,3-diketone **19**.

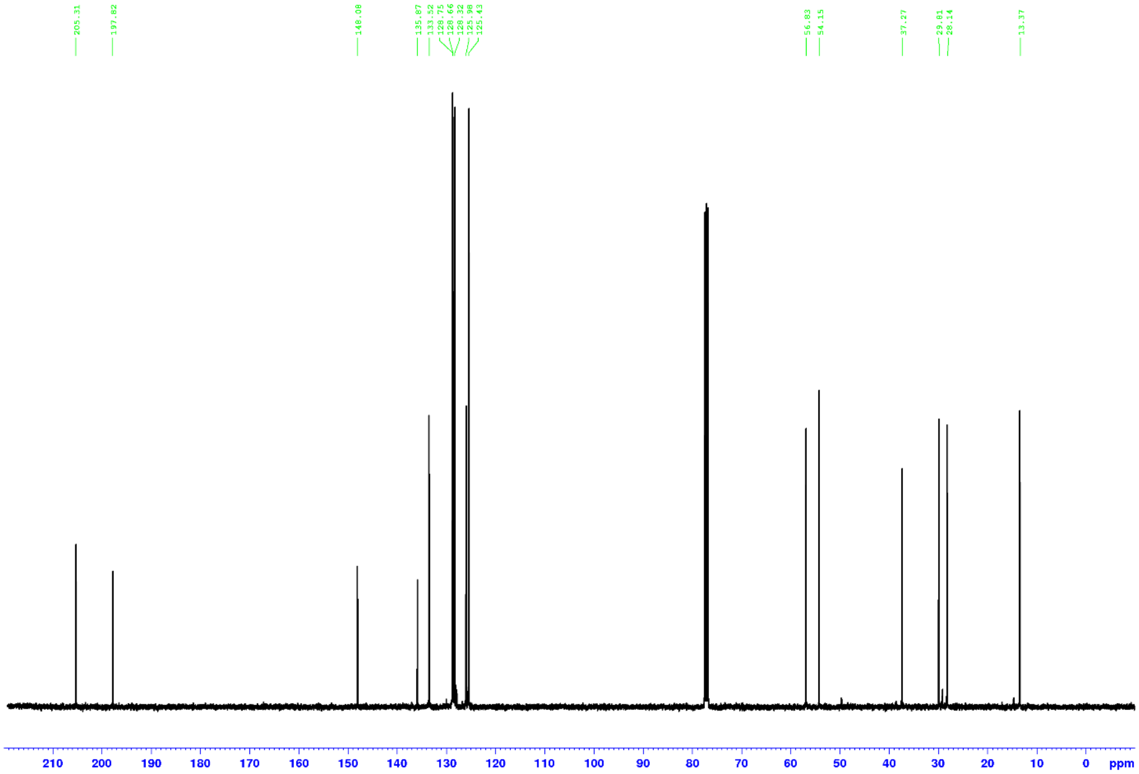

Figure S17. $^{13}C\{^{1}H\}$ NMR ($CDCl_3$,100.6 MHz) spectrum of 1,3-diketone **19**.

2,5-Dimethyl-1-phenyl-5-(4'-chlorophenyl)hexane-1,3-dione (20)

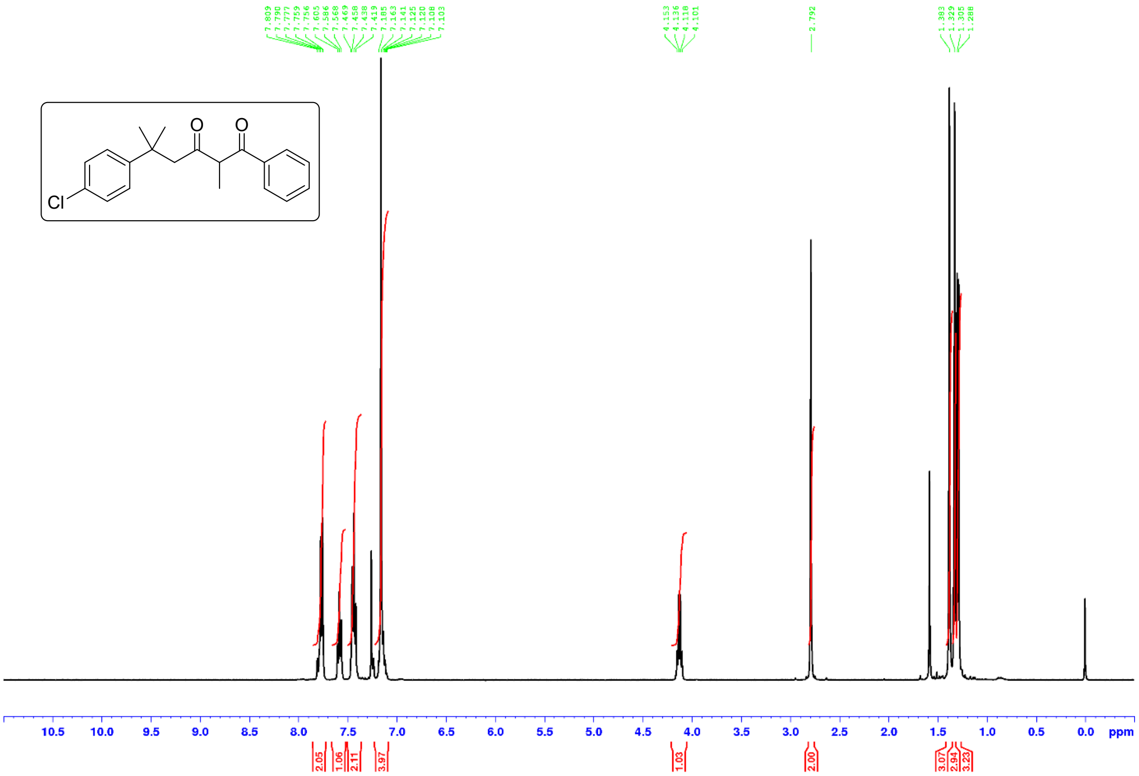

Figure S18. ^{1}H NMR ($CDCl_3$, 400 MHz) spectrum of 1,3-diketone **20**.

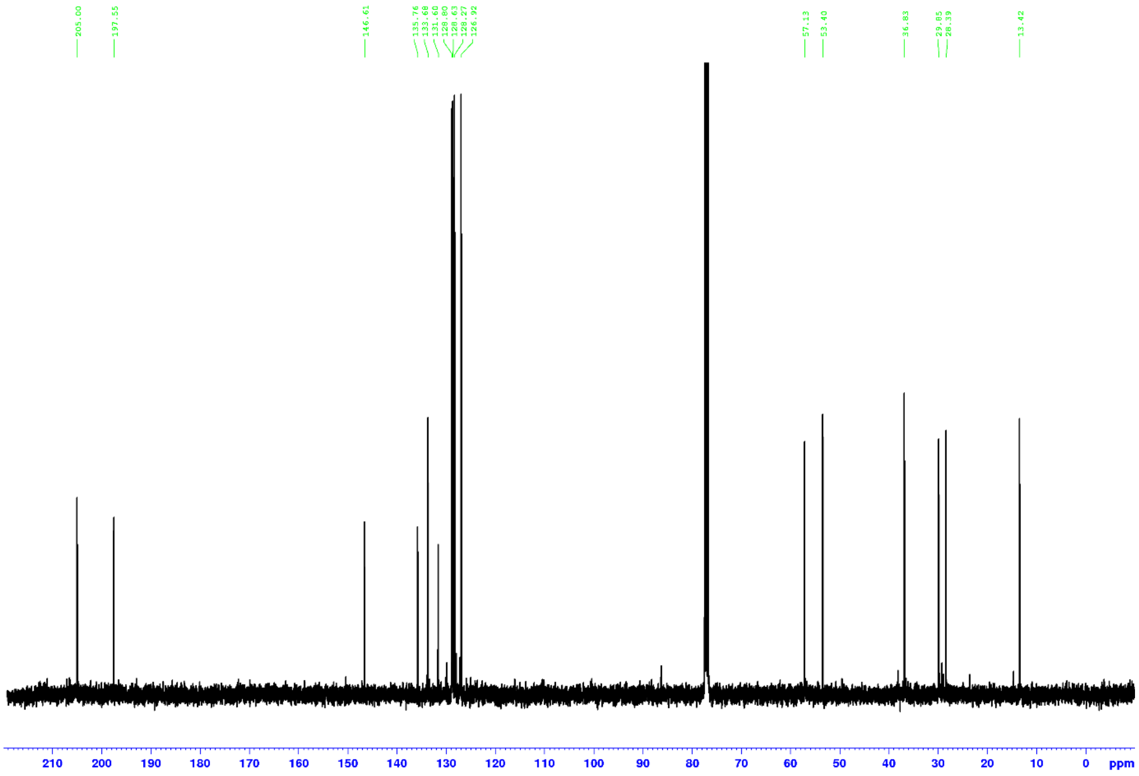

Figure S19. $^{13}C\{^{1}H\}$ NMR ($CDCl_3$, 100.6 MHz) spectrum of 1,3-diketone **20**.

2,5-Dimethyl-1-phenyl-5-(4'-fluorophenyl)hexane-1,3-dione (21)

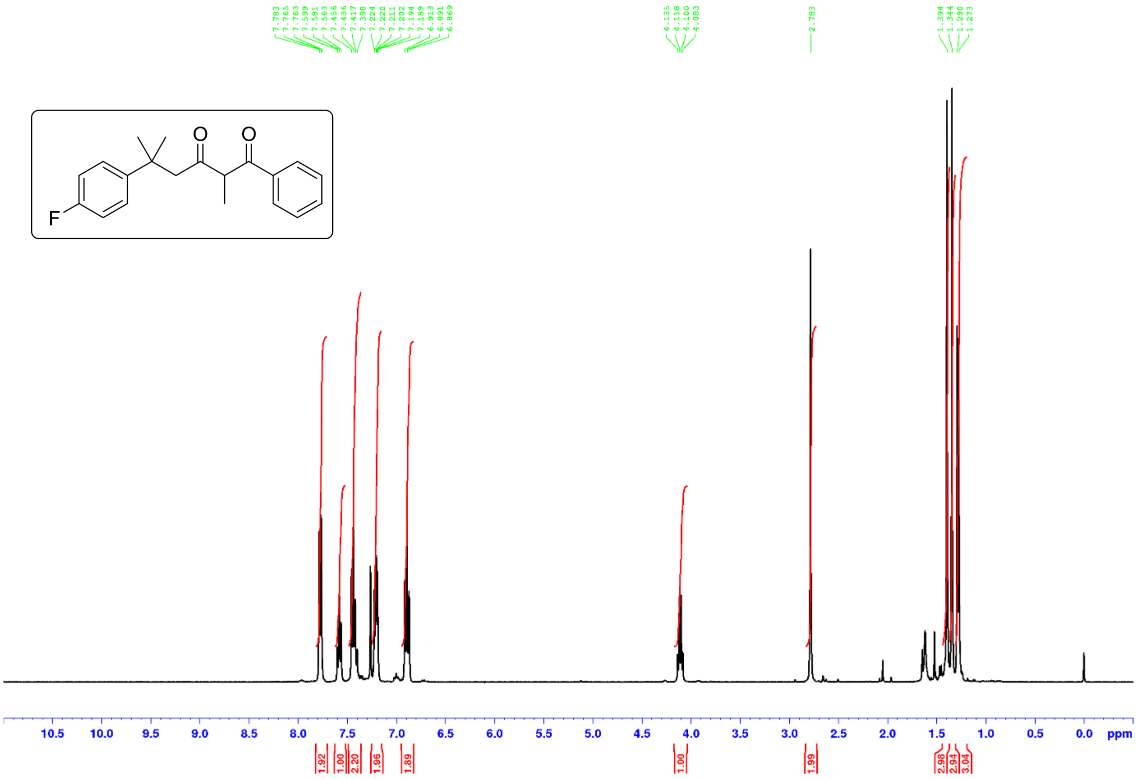

Figure S20. ^{1}H NMR ($CDCl_3$, 400 MHz) spectrum of 1,3-diketone **21**.

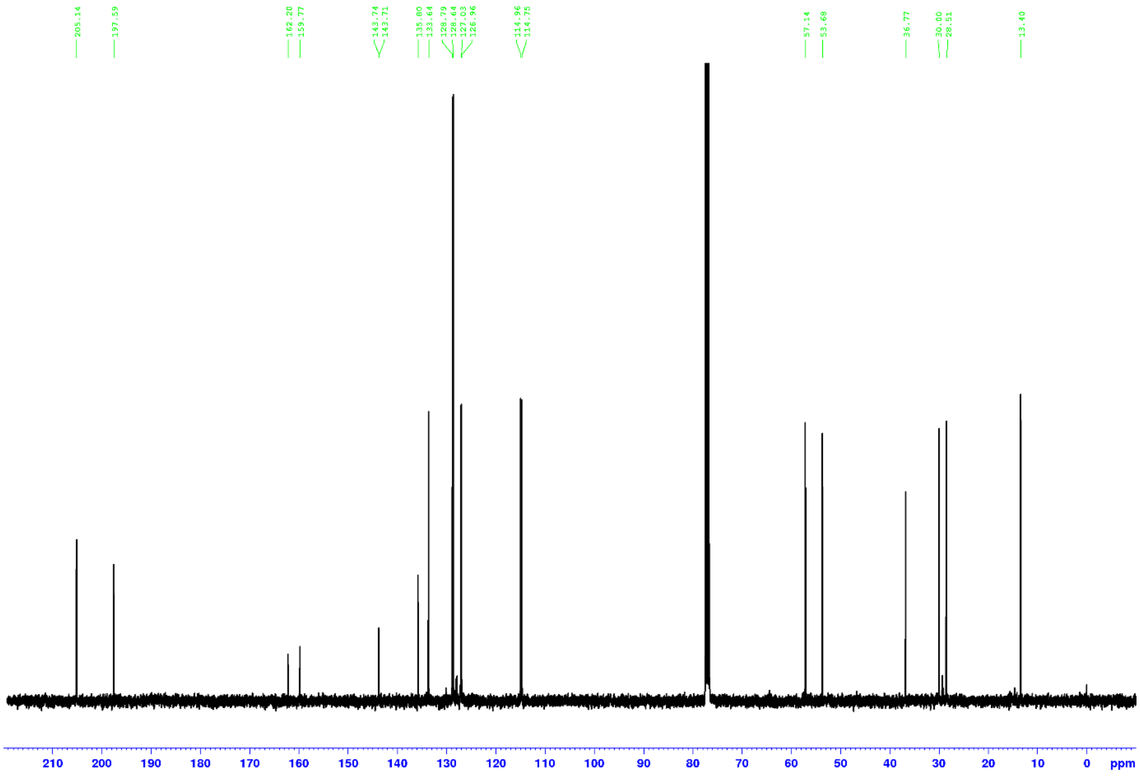

Figure S21. $^{13}C\{^{1}H\}$ NMR ($CDCl_3$,100.6 MHz) spectrum of 1,3-diketone **21**.

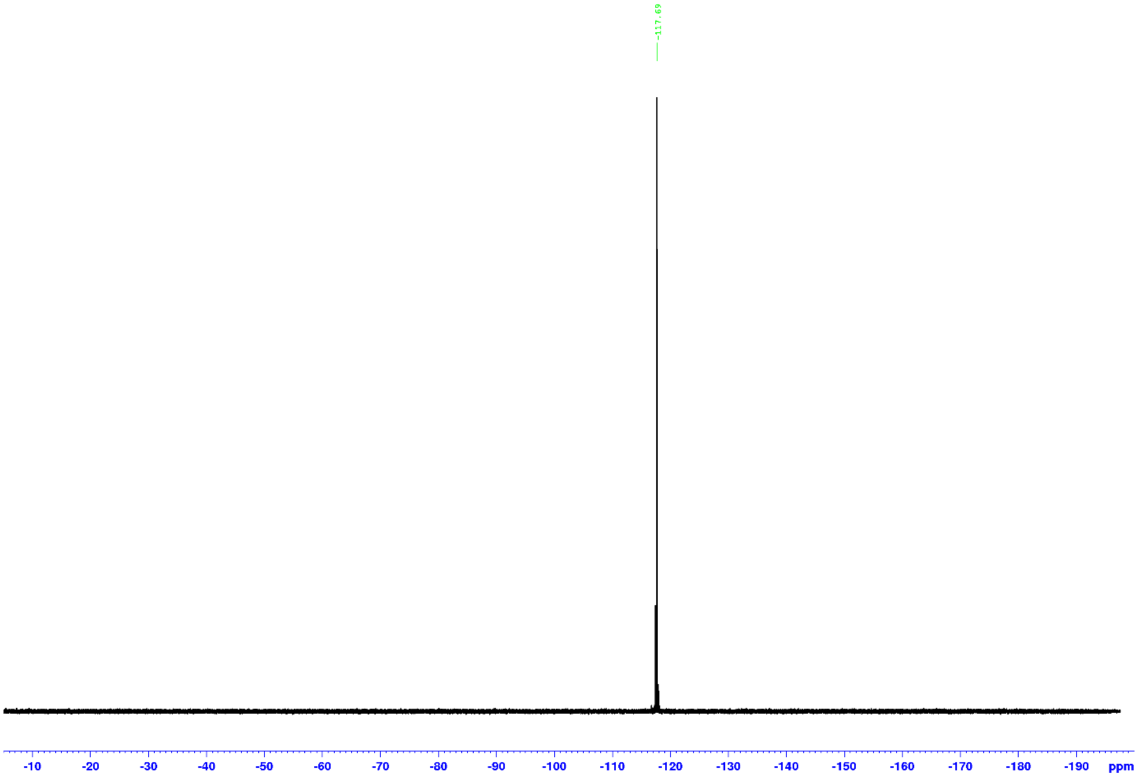

Figure S22. $^{19}F\{^1H\}$ NMR ($CDCl_3$, 376.5 MHz) spectrum of 1,3-diketone **21**.

2-Ethyl-5-methyl-1,5-diphenylhexa-1,3-dione (24)

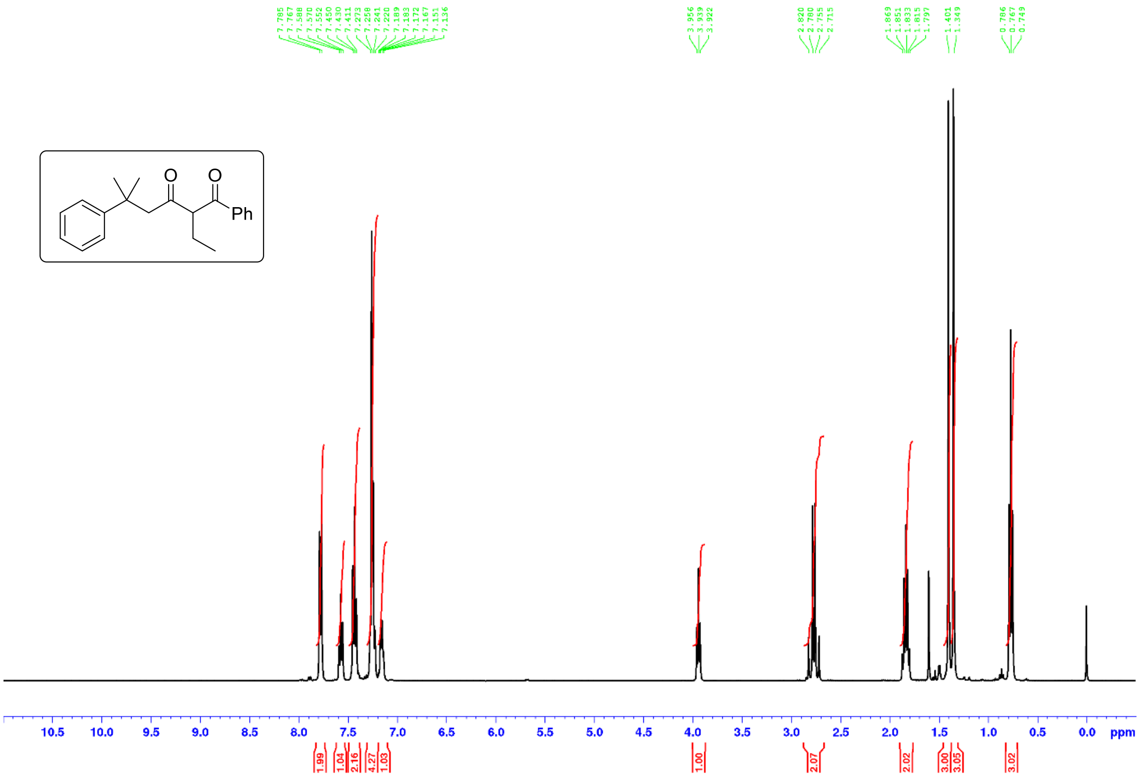

Figure S23. ^{1}H NMR ($CDCl_3$, 400 MHz) spectrum of 1,3-diketone **24**.

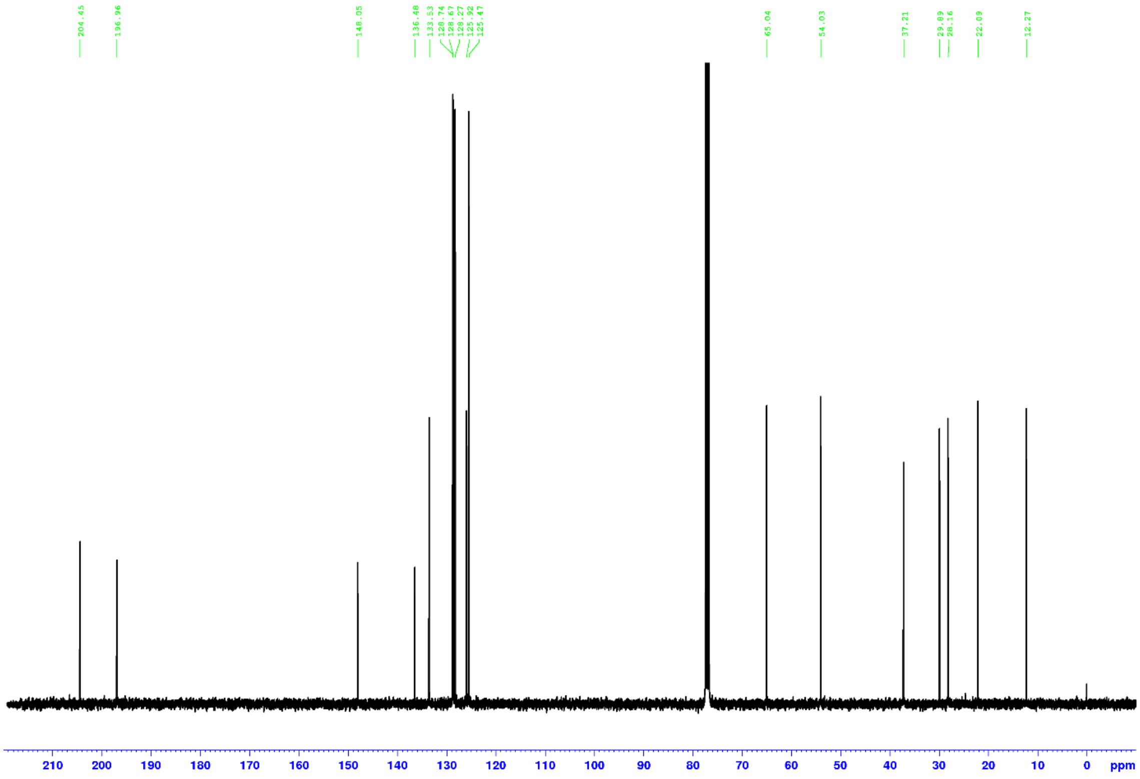

Figure S24. $^{13}C\{^{1}H\}$ NMR ($CDCl_3$, 100.6 MHz) spectrum of 1,3-diketone **24**.

2-(Cyclohexylmethyl)-5-methyl-1,5-diphenylhexane-1,3-dione (25)

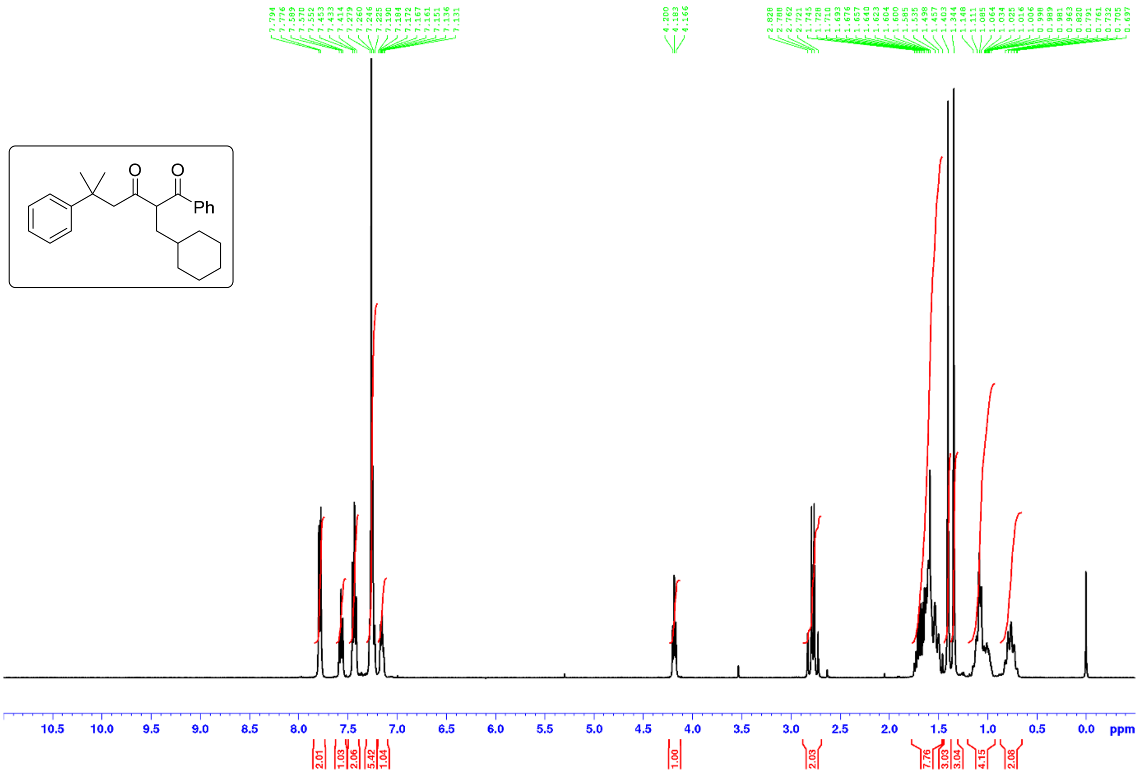

Figure S25. ^{1}H NMR ($CDCl_3$, 400 MHz) spectrum of 1,3-diketone **25**.

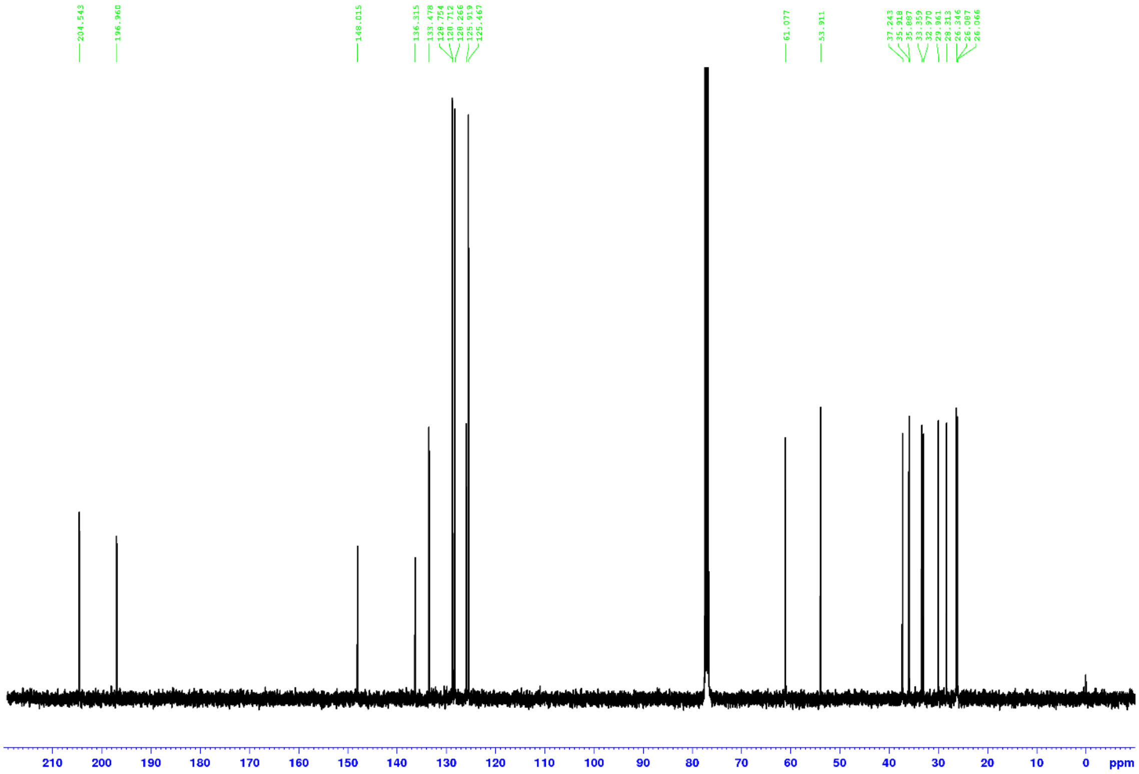

Figure S26. $^{13}C\{^{1}H\}$ NMR ($CDCl_3$, 100.6 MHz,) spectrum of 1,3-diketone **25**.

5-Diazo-2-methyl-2-phenylheptan-4-one (26)

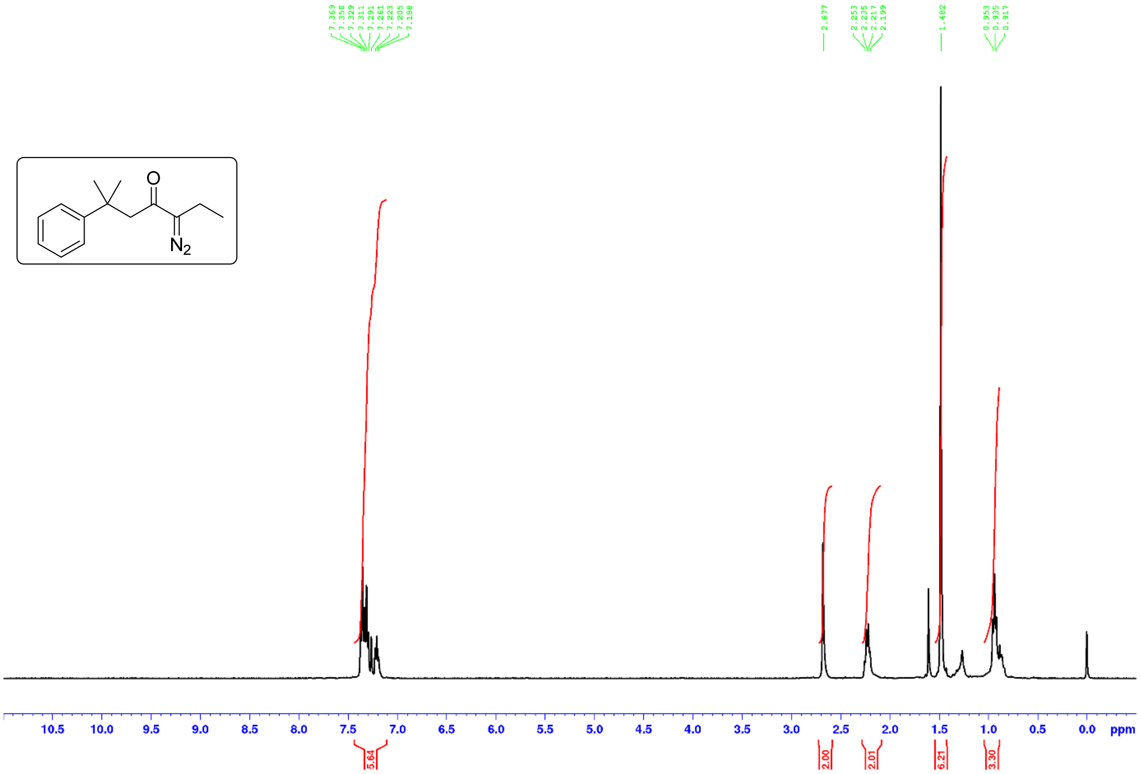

Figure S27. ^{1}H NMR ($CDCl_3$, 300 MHz) spectrum of α-diazoketone **26**.

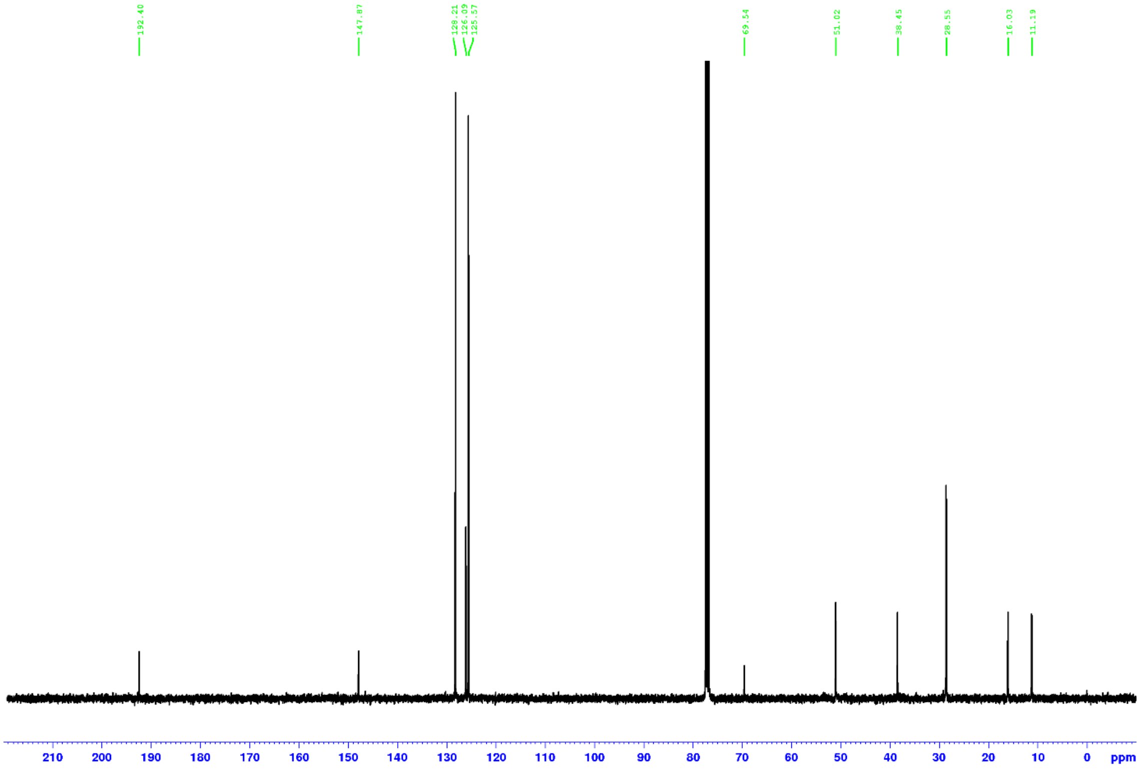

Figure S28. $^{13}C\{^{1}H\}$ NMR ($CDCl_3$, 100.6 MHz) spectrum of α-diazoketone **26**.

1-(Cyclohexyl)-2-diazo-5-methyl-5-phenylhexan-3-one (27)

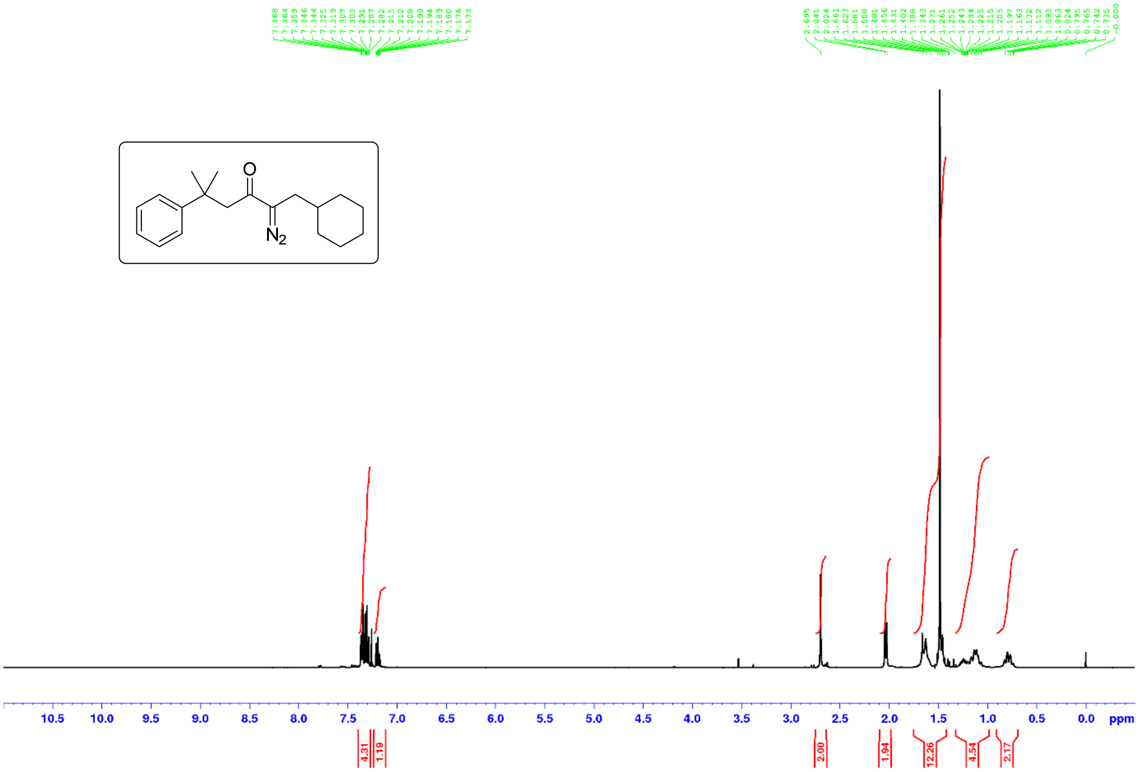

Figure S29. ^{1}H NMR ($CDCl_3$, 400 MHz) spectrum of α-diazoketone **27**.

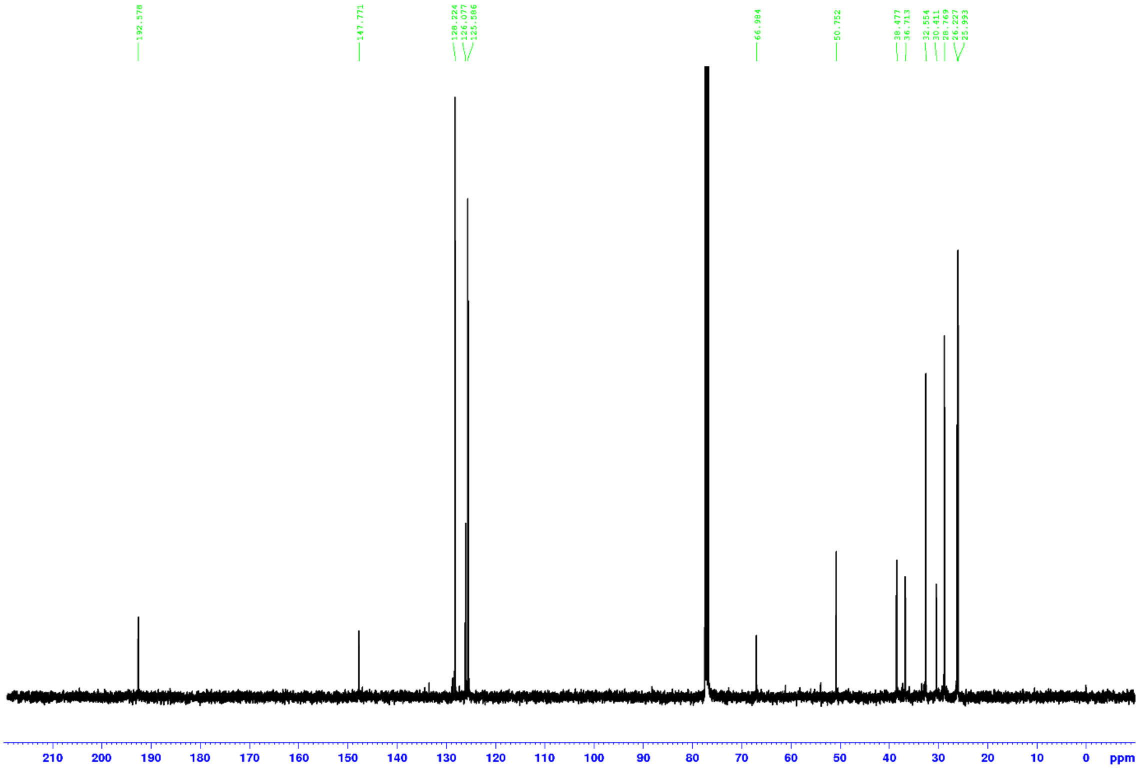

Figure S30. $^{13}C\{^{1}H\}$ NMR ($CDCl_3$, 100.6 MHz) spectrum of α-diazoketone **27**.

1-(4-Chlorophenyl)-3-diazopyrrolidin-2-one (29)[12]

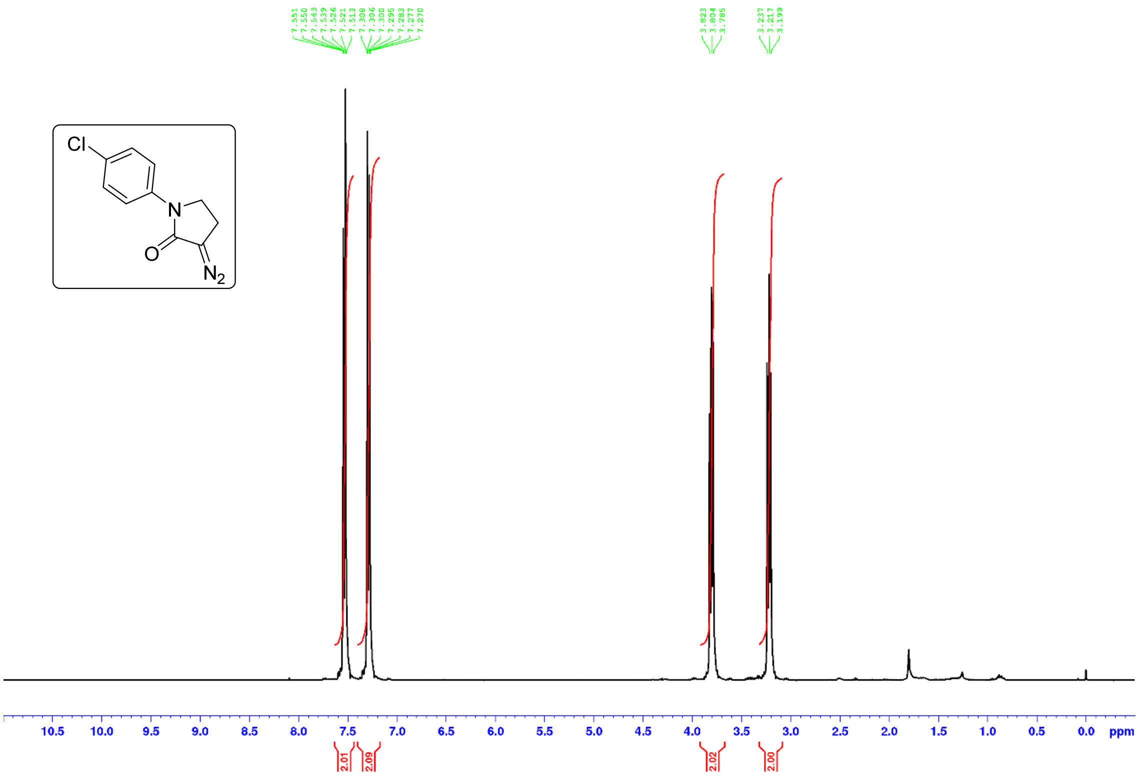

Figure S31. ^{1}H NMR ($CDCl_3$, 400 MHz) spectrum of α-diazoketone **29**.

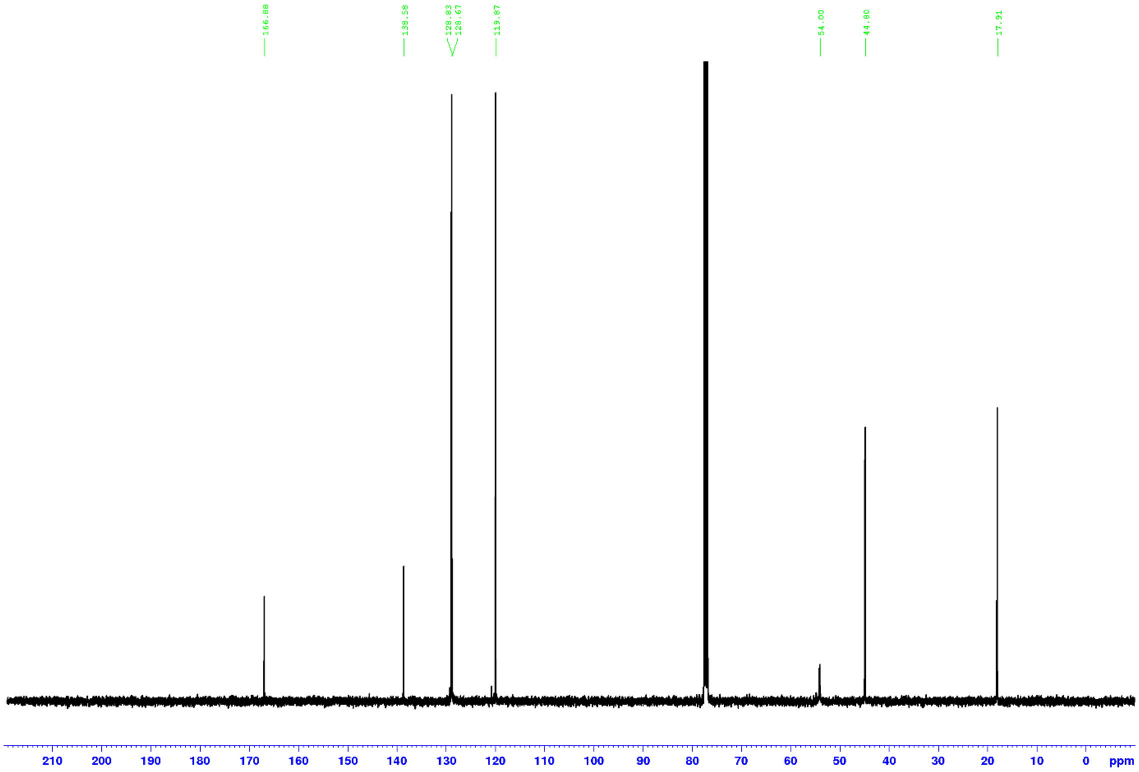

Figure S32. $^{13}C\{^{1}H\}$ NMR ($CDCl_3$,100.6 MHz) spectrum of α-diazoketone **29**.

3,8a-Dihydro-3,3,8a-trimethylazulen-1(2*H*)-one (31)[8]

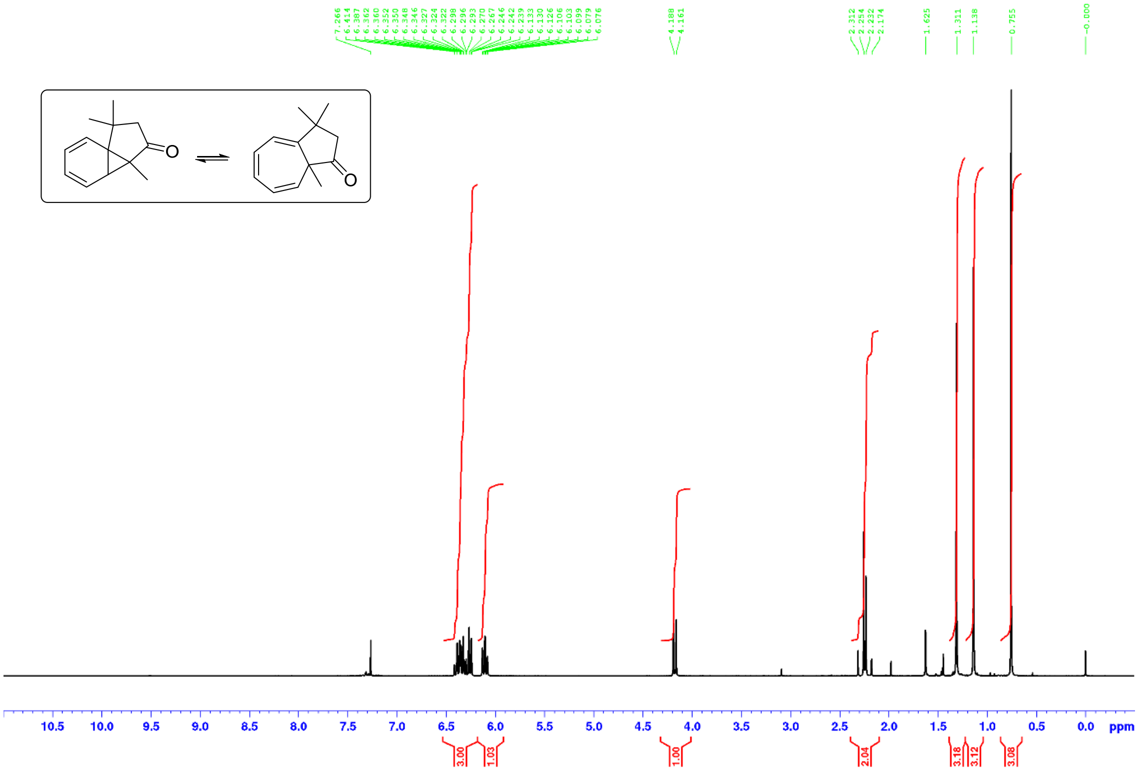

Figure S33. ^{1}H NMR ($CDCl_3$, 300 MHz) spectrum of azulenone **31** isolated from the telescoped process for the synthesis and aromatic addition of α-diazoketone **11** (Scheme 11); spectroscopic data were in agreement with those previously reported.[8]

5-Methyl-5-phenylhexane-2,3-dione (34)[13]

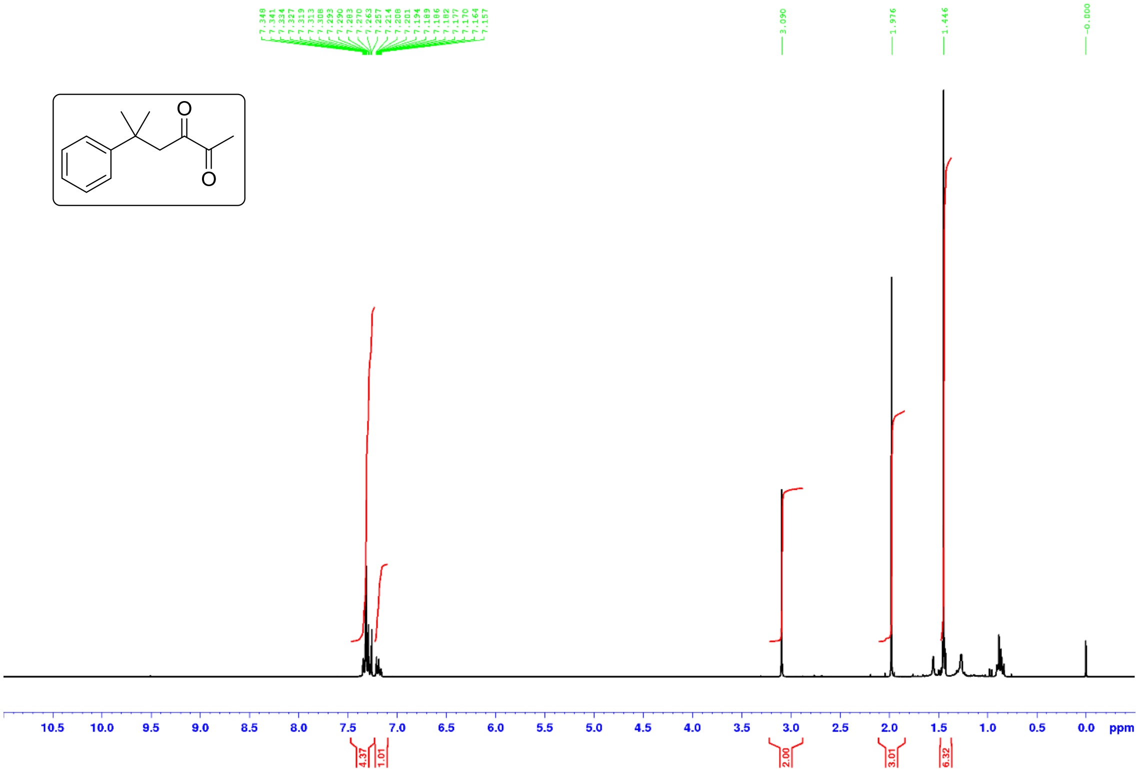

Figure S34. ^{1}H NMR ($CDCl_3$, 300 MHz) spectrum of diketone **34**.

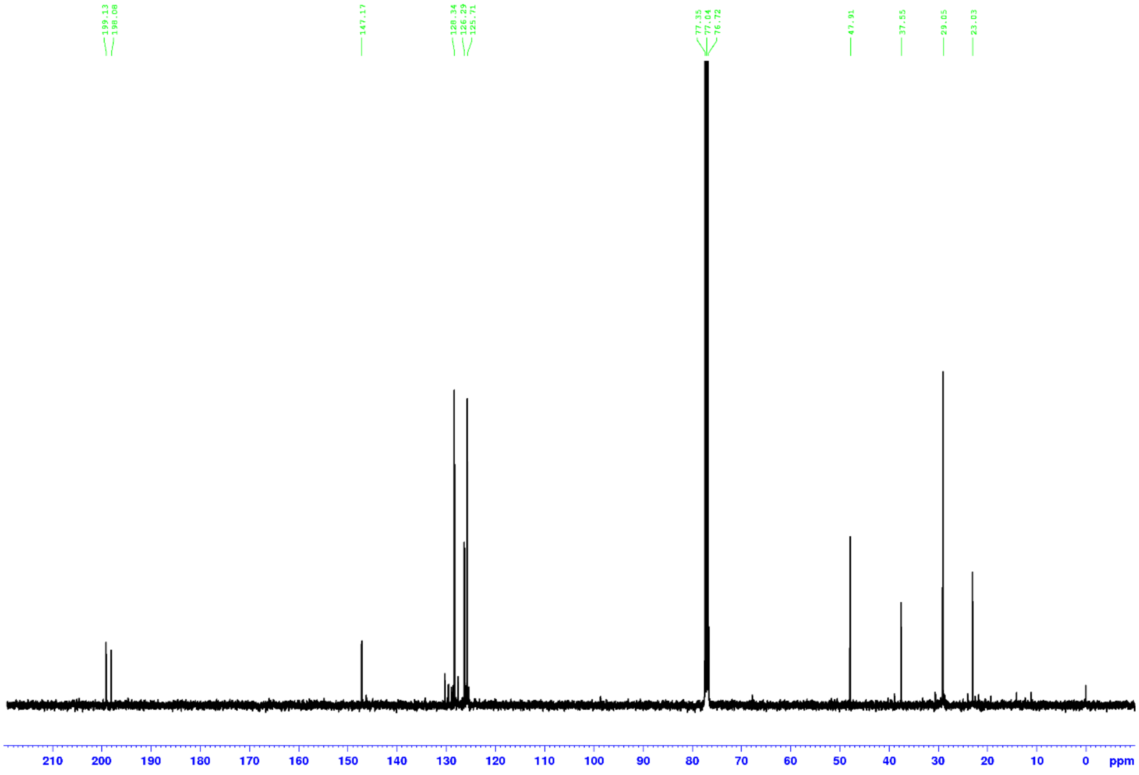

Figure S35. $^{13}C\{^{1}H\}$ NMR ($CDCl_3$,100.6 MHz) spectrum of diketone **34**.

1,2,3b,4-Tetrahydro-1,1,3a,4,11,12-hexamethyl-7-phenyl-4,10-etheno-6H,10H-cyclopenta[1,3]cyclopropa[1,2-d][1,2,4]triazolo[1,2-a]pyridazine-3,6,8(3a*H*,7*H*)-trione (10)[8]

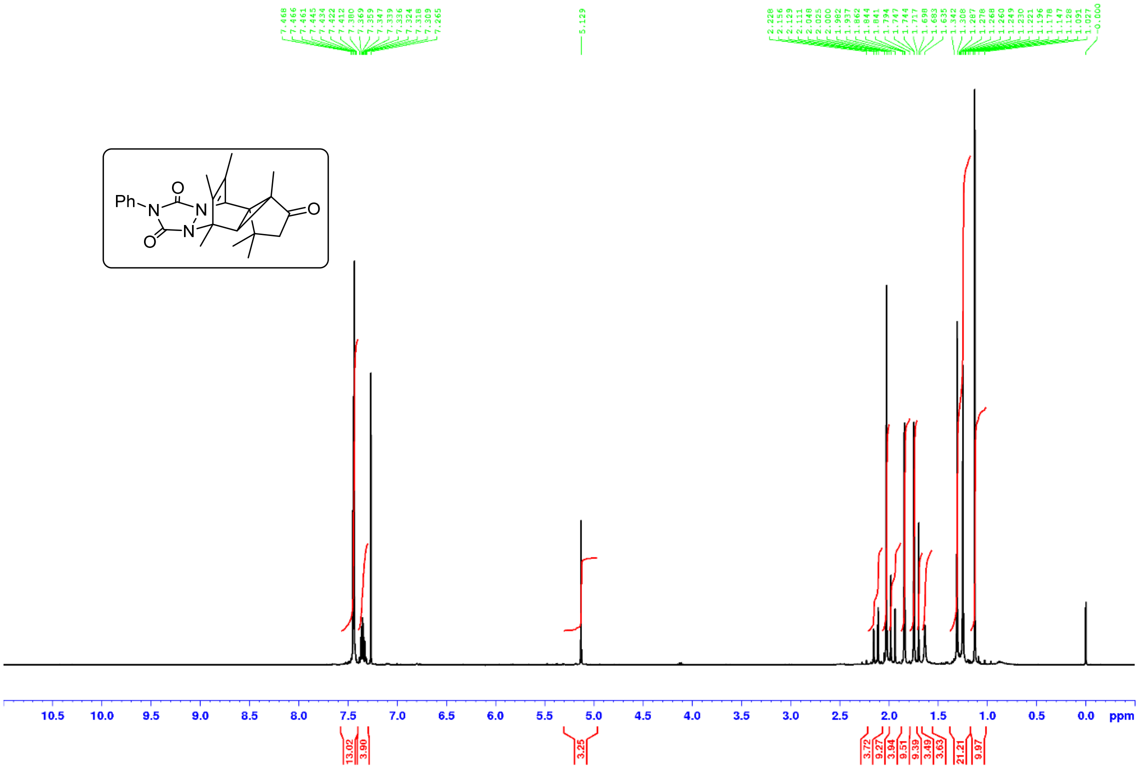

Figure S36. ^{1}H NMR ($CDCl_3$, 300 MHz) spectrum of cycloadduct **10** isolated from the telescoped process for the synthesis and aromatic addition of α-diazoketone **8** (Scheme 11); spectroscopic data were in agreement with those previously reported.[8]

3-Hydroxy-5-methyl-1,5-diphenylhexan-1-one (35)

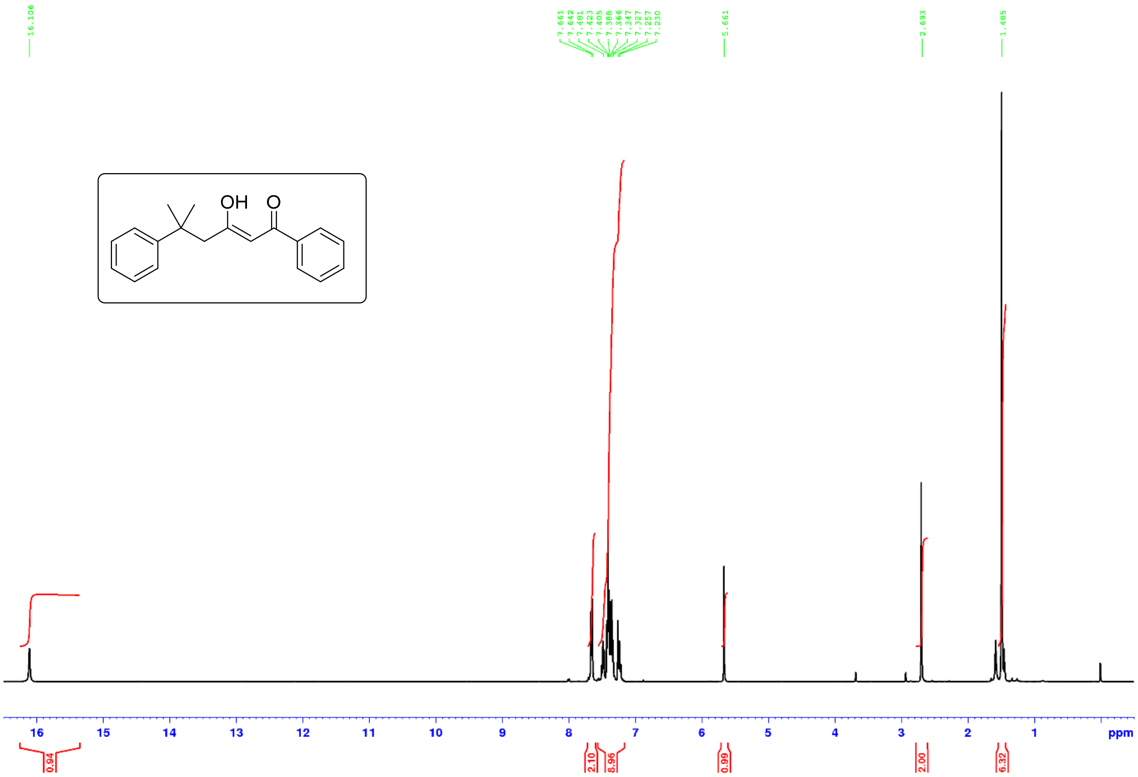

Figure S37. ^{1}H NMR ($CDCl_3$, 400 MHz) spectrum of hydroxyenone **35**.

2-Diazo-5-methyl-1,5-diphenylhexan-1,3-dione (36)

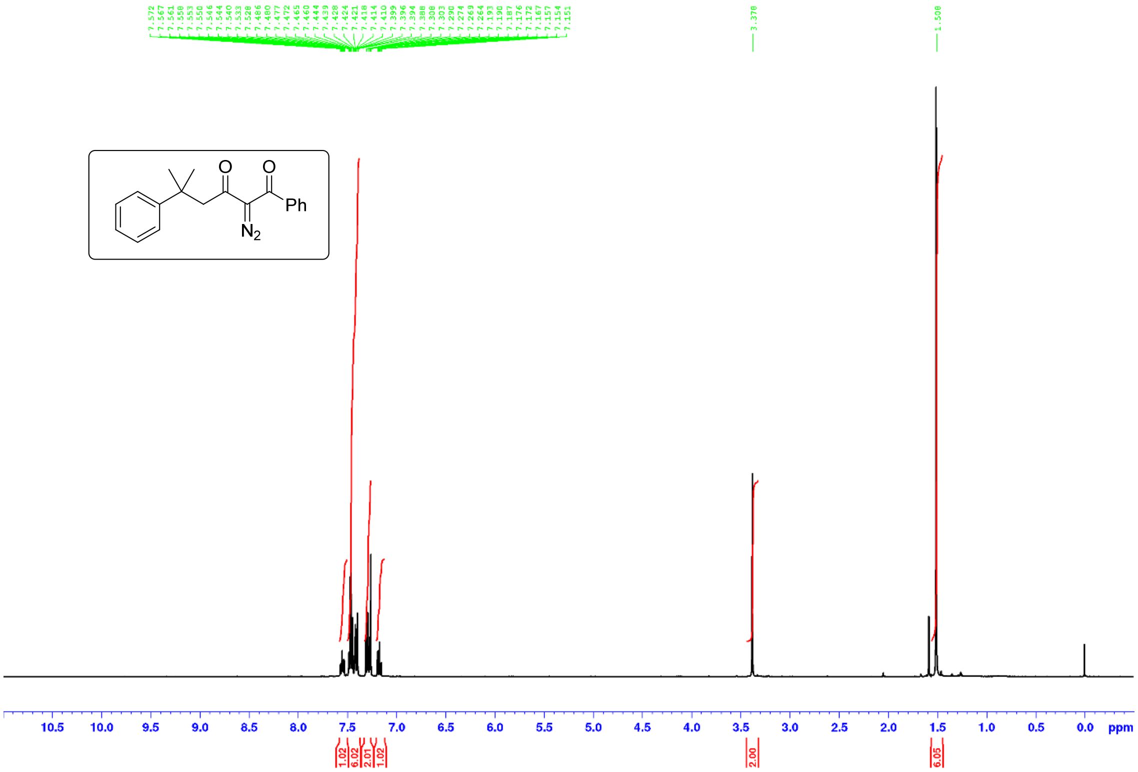

Figure S38. ^{1}H NMR ($CDCl_3$, 400 MHz) spectrum of α-diazoketone **36**.

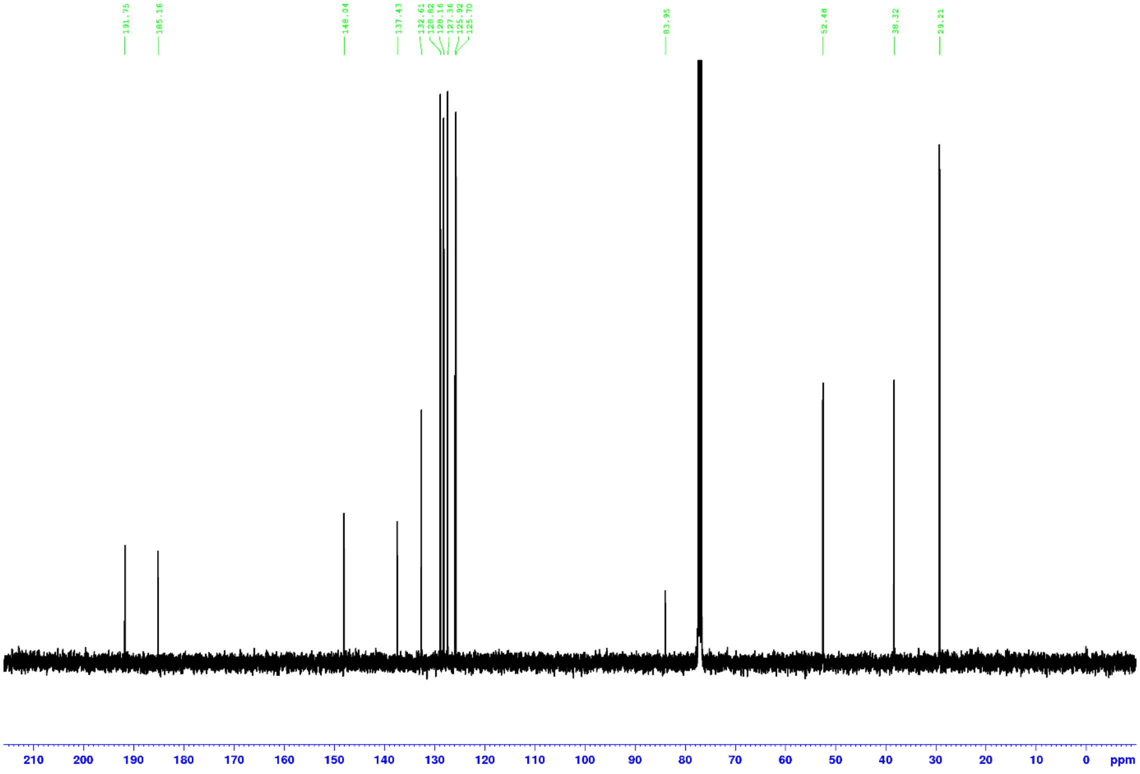

Figure S39. $^{13}C\{^{1}H\}$ NMR ($CDCl_3$, 100.6 MHz) spectrum of α-diazoketone **36**.

5-(4-Chlorophenyl)-5-methyl-3-oxohexanenitrile (37)

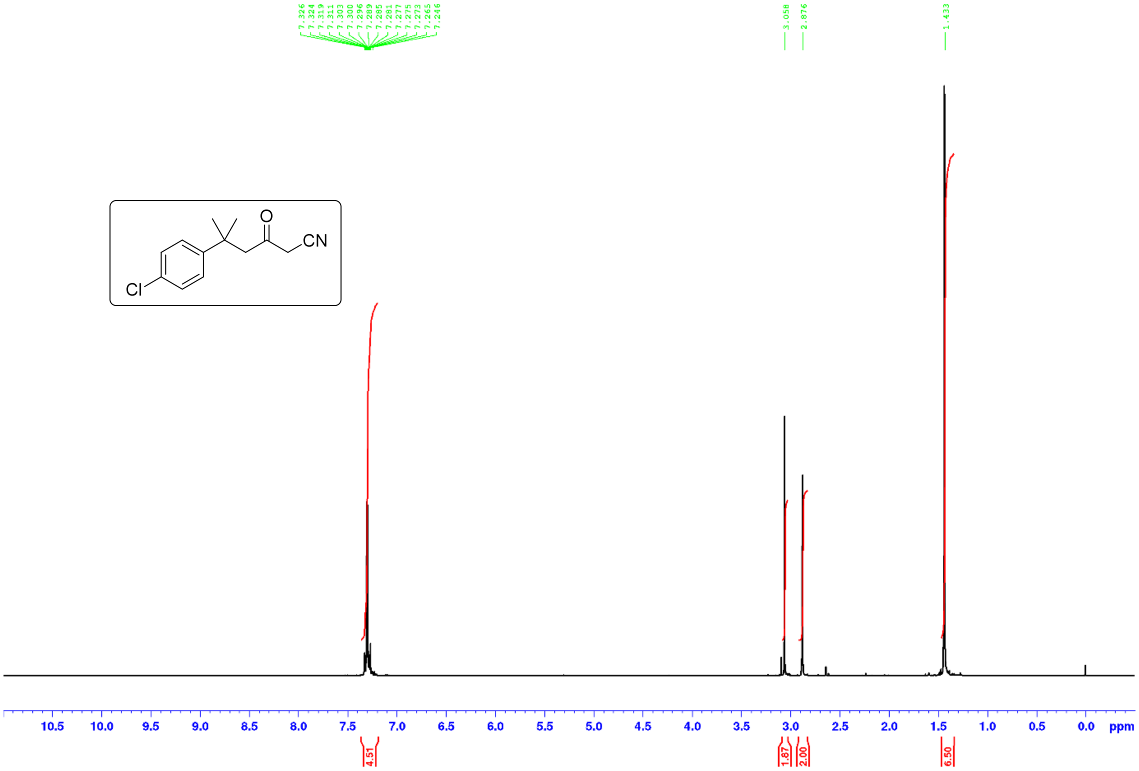

Figure S40. ^{1}H NMR ($CDCl_3$, 400 MHz) spectrum of β-ketonitrile **37**.

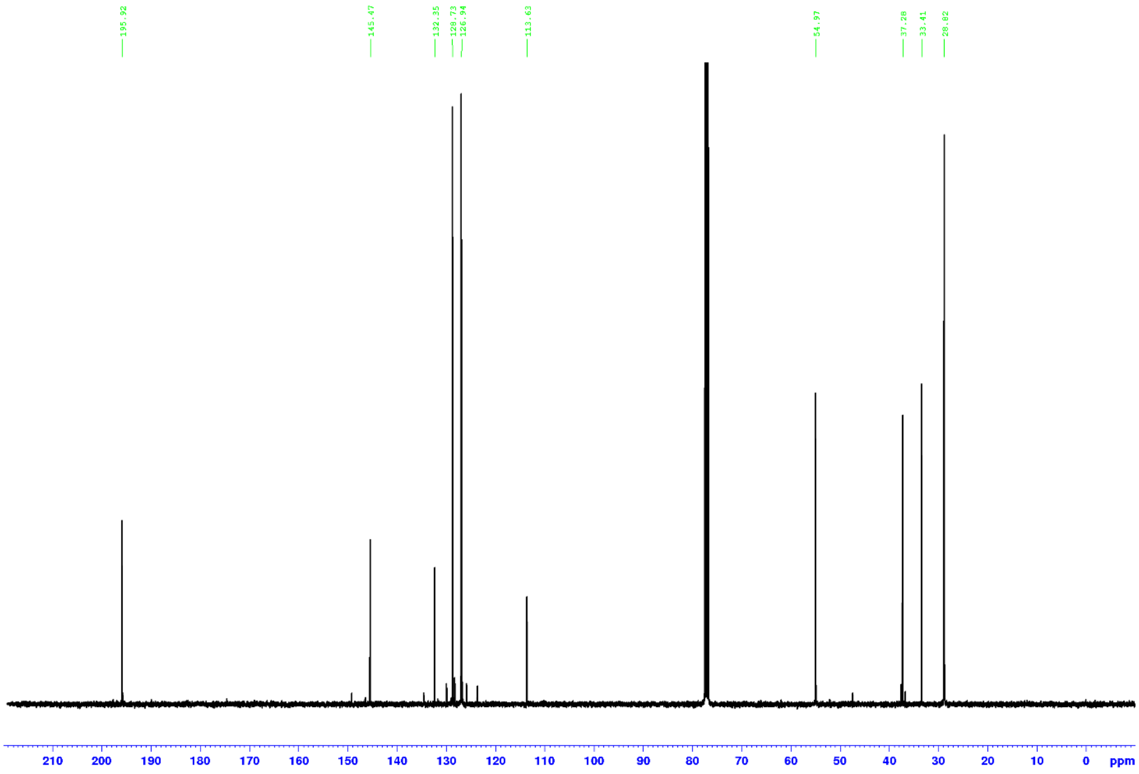

Figure S41. $^{13}C\{^{1}H\}$ NMR ($CDCl_3$, 100.6 MHz) spectrum of β-ketonitrile **37**.

5-(4-Chlorophenyl)-2-diazo-5-methyl-3-oxohexanenitrile (38)

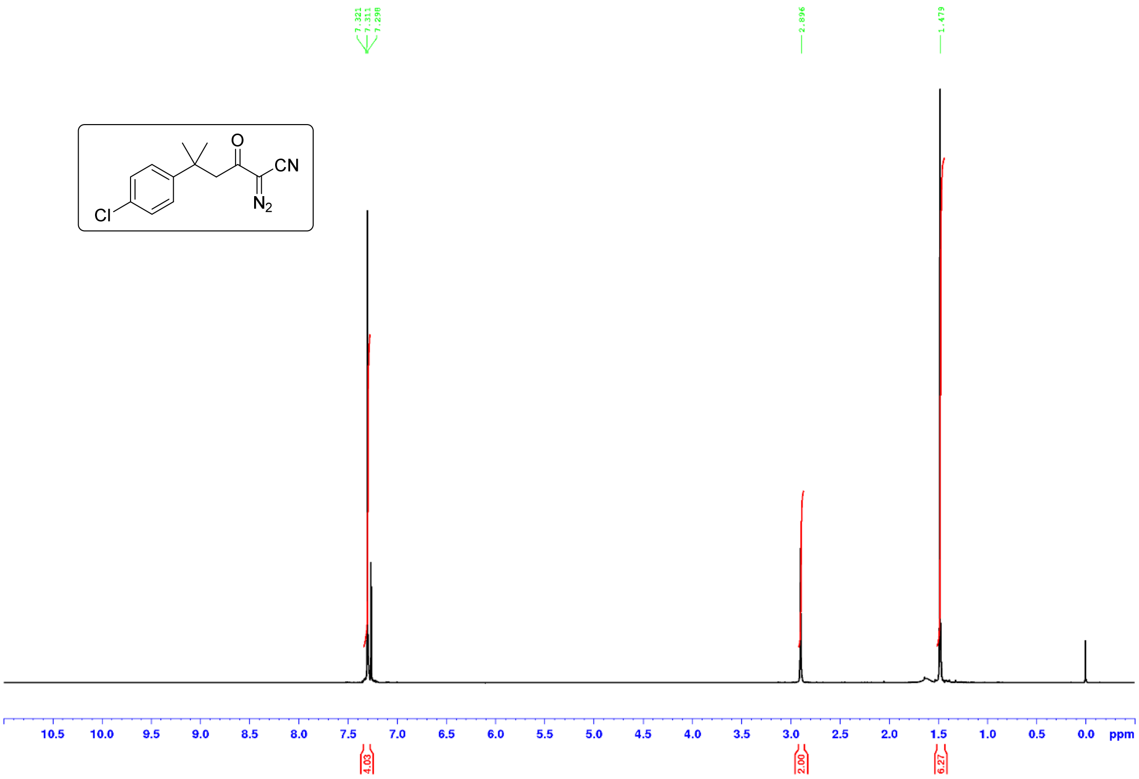

Figure S42. ^{1}H NMR ($CDCl_3$, 400 MHz) spectrum of α-diazoketone **38**.

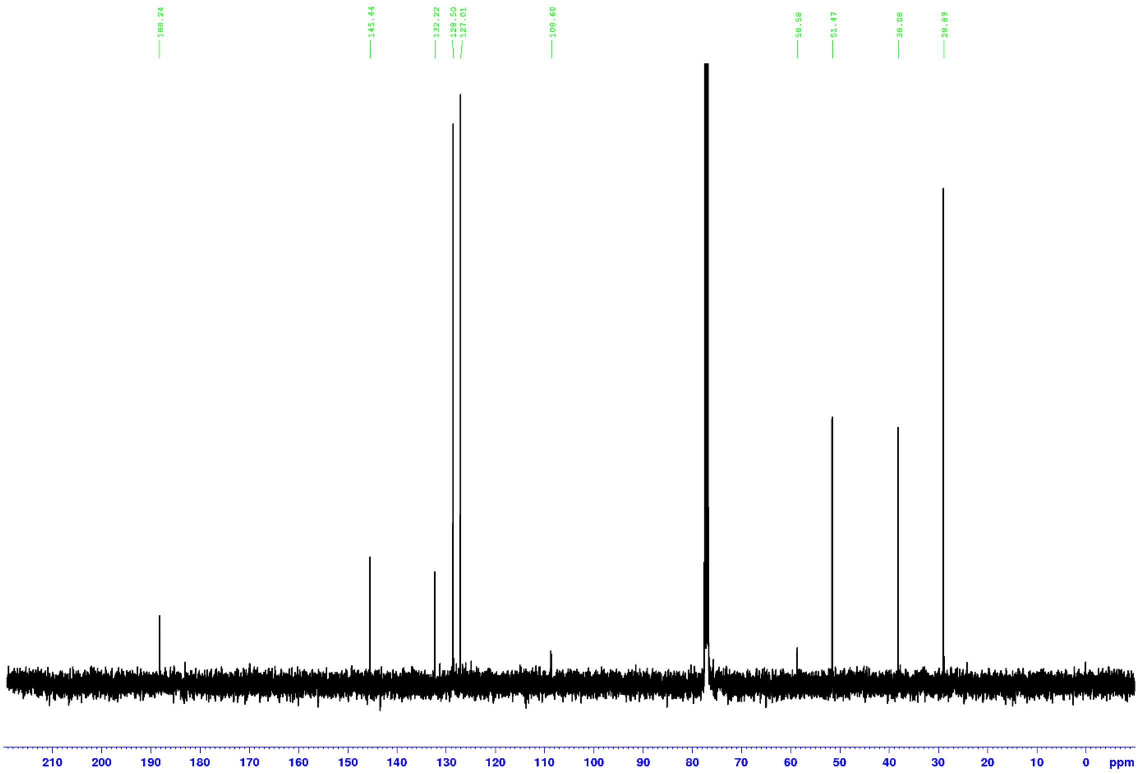

Figure S43. $^{13}C\{^{1}H\}$ NMR ($CDCl_3$, 100.6 MHz) spectrum of α-diazoketone **38**.

N,*N*-Dibenzyl-2cyanoacetamide (47)[14]

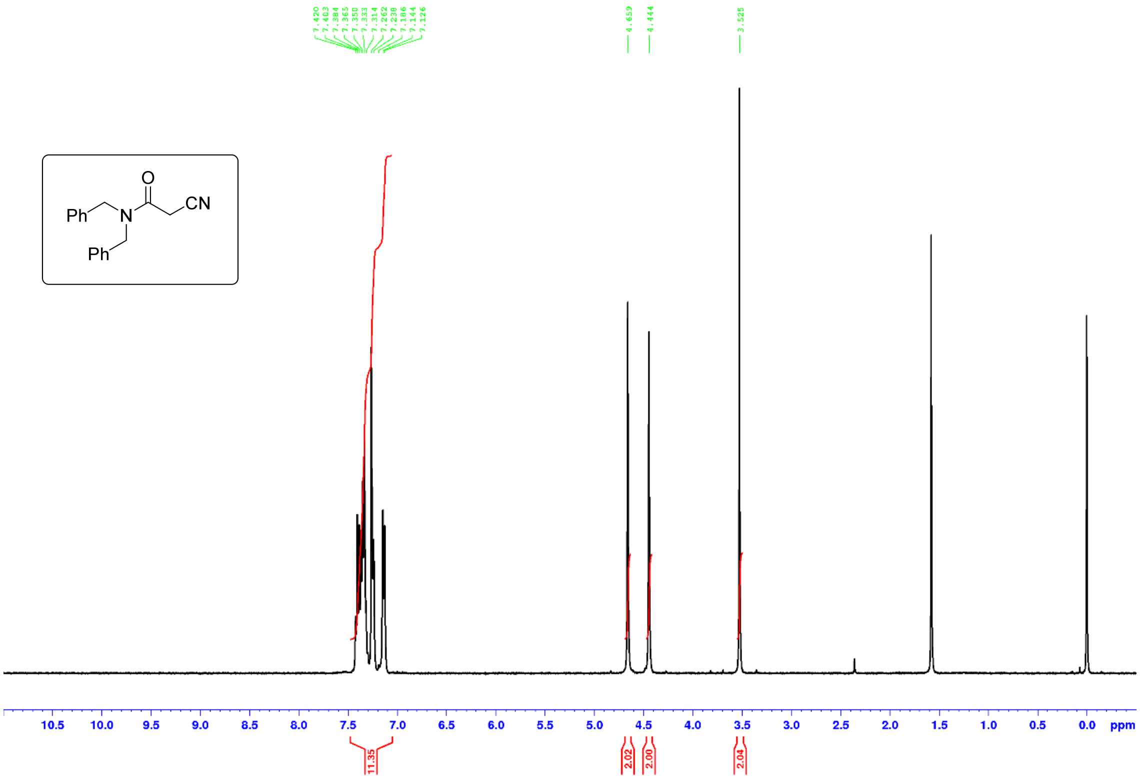

Figure S44 ^{1}H NMR ($CDCl_3$, 400 MHz) spectrum of α-cyanoacetamide **47**.

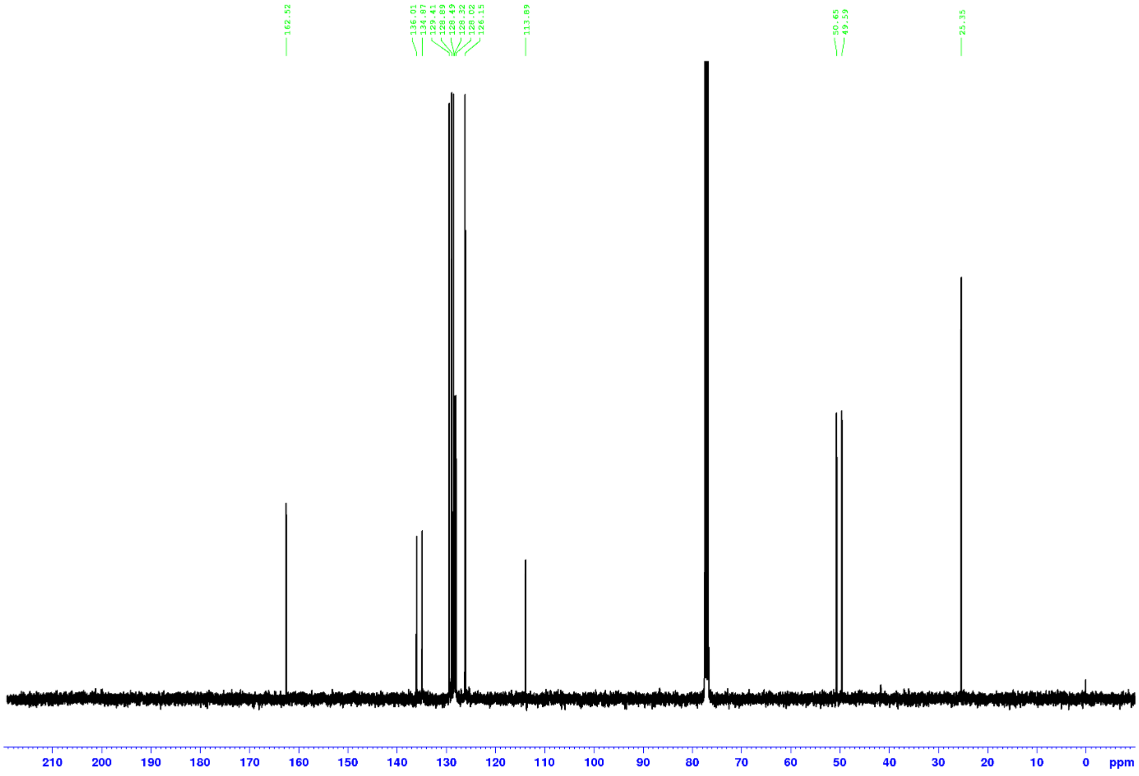

Figure S45. $^{13}C\{^{1}H\}$ NMR ($CDCl_3$, 100.6 MHz) spectrum of α-cyanoacetamide **47**.

N-(tert-Butyl)-2-cyano-N-(4-chlorobenzyl)acetamide (48)[14]

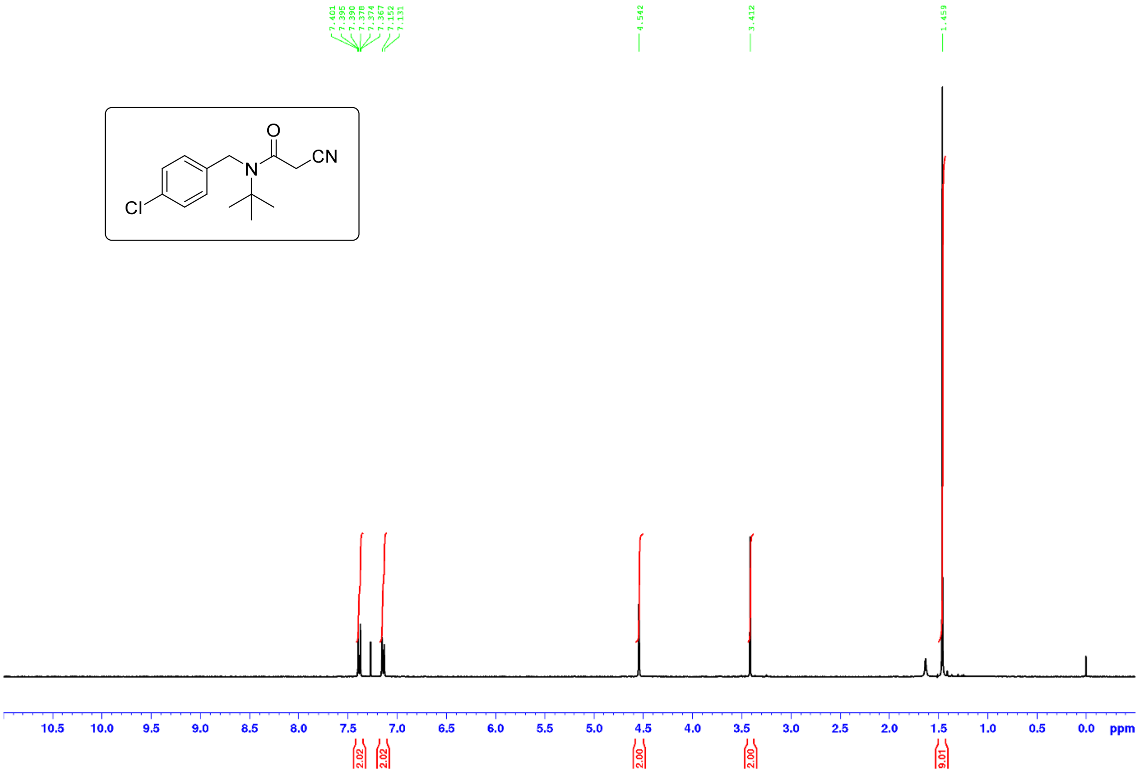

Figure S46. ^{1}H NMR ($CDCl_3$, 400 MHz) spectrum of α-cyanoacetamide **48**.

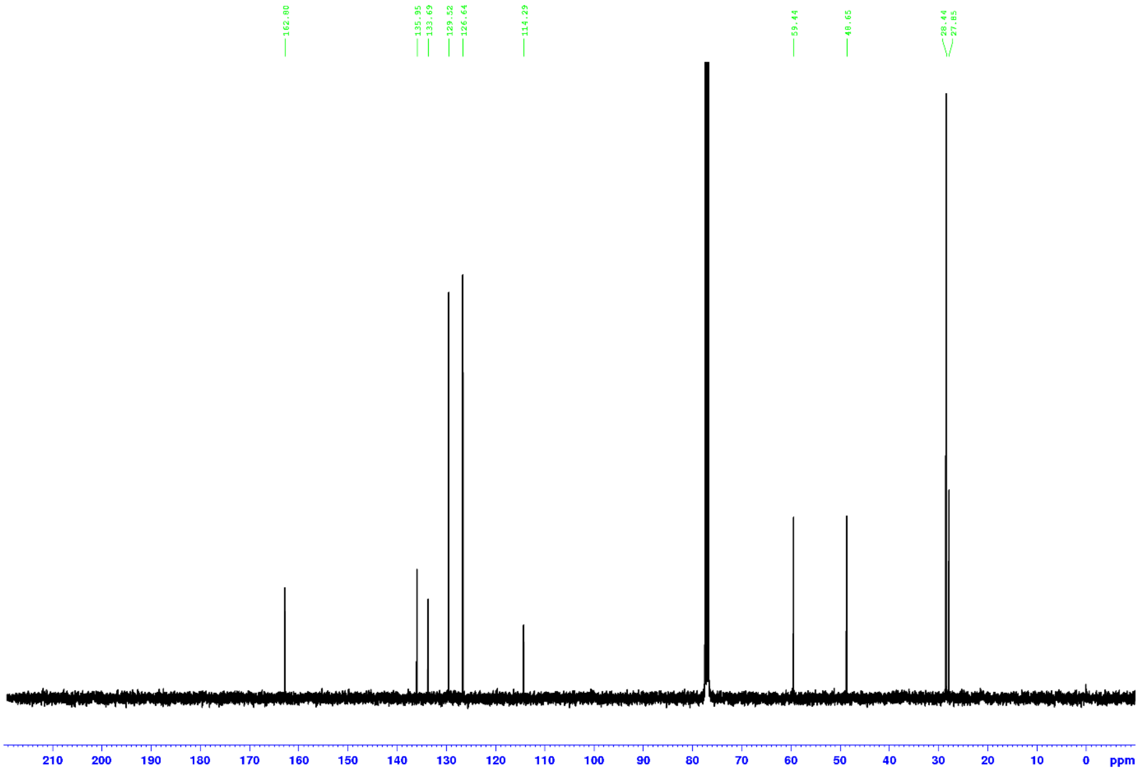

Figure S47. $^{13}C\{^{1}H\}$ NMR ($CDCl_3$, 100.6 MHz) spectrum of α-cyanoacetamide **48**.

N-Benzyl-*N*-*tert*-butyl-2-cyanoacetamide (49)[14]

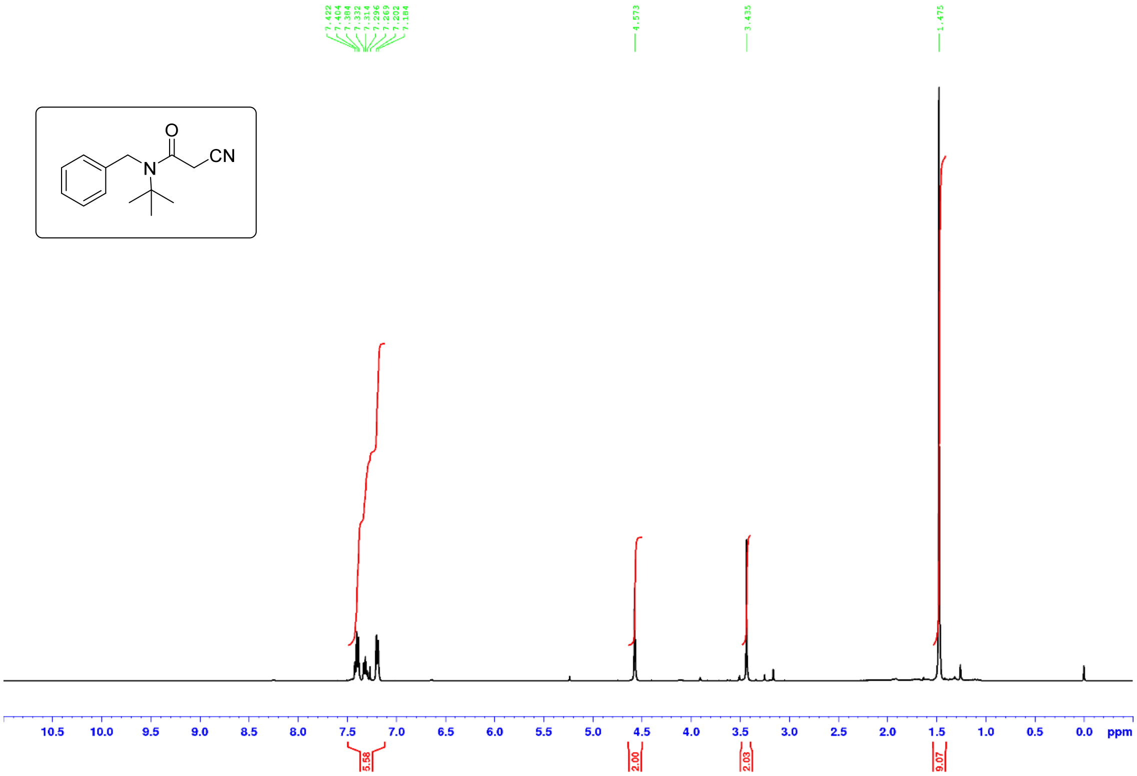

Figure S48. ^{1}H NMR ($CDCl_3$, 400 MHz) spectrum of α-cyanoacetamide **49**.

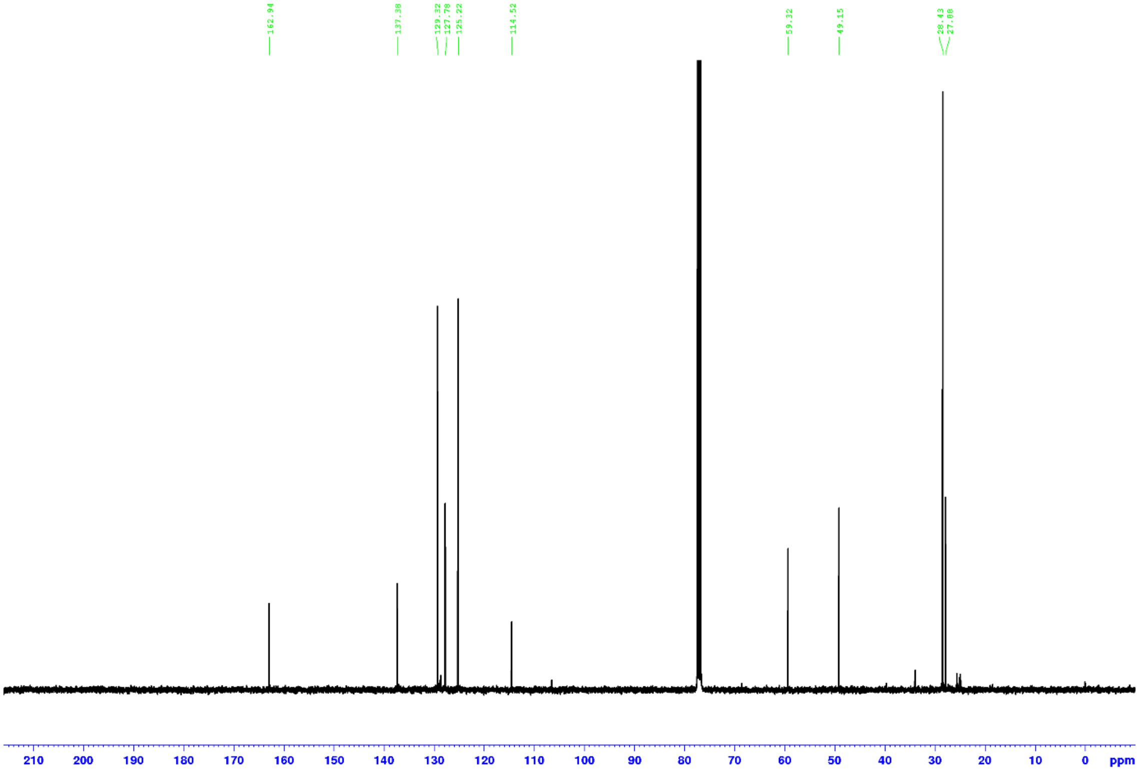

Figure S49. ^{13}C{^{1}H} NMR ($CDCl_3$, 100.6 MHz) spectrum of α-cyanoacetamide **49**.

_N_-(_tert_-Butyl)-2-cyano-_N_-(4-fluorobenzyl)acetamide (50)[14]

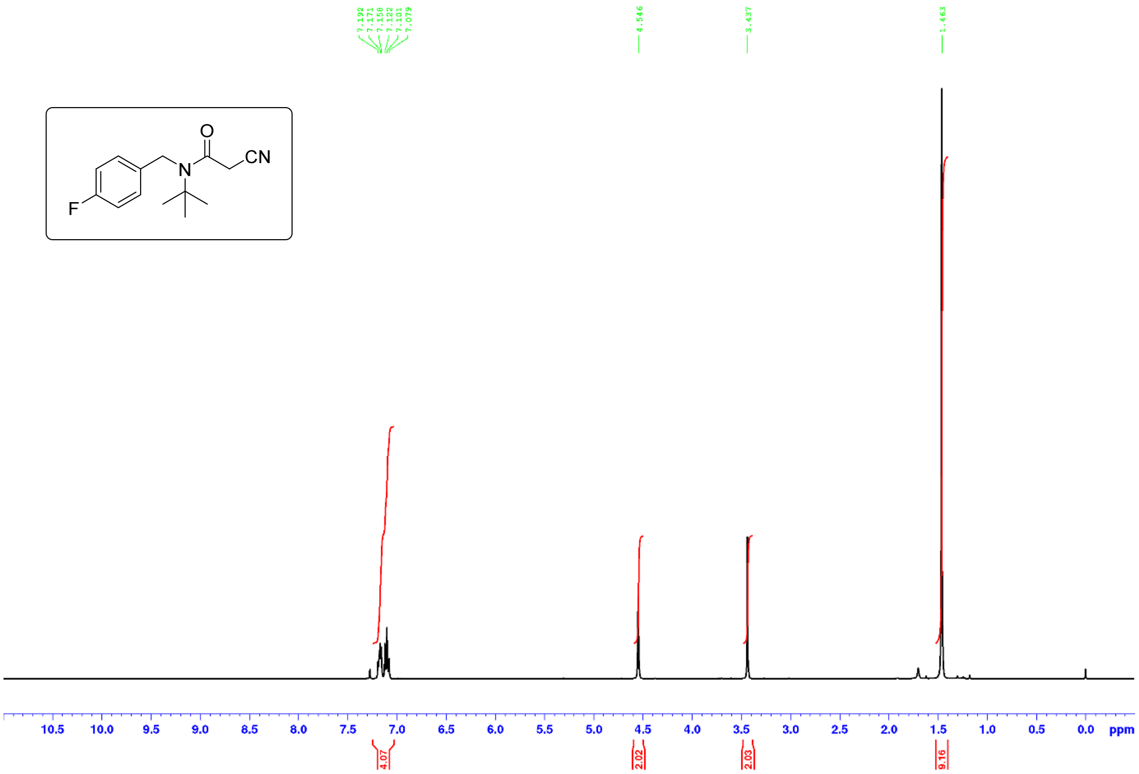

Figure S50. ^{1}H NMR ($CDCl_3$, 400 MHz) spectrum of α-cyanoacetamide **50**.

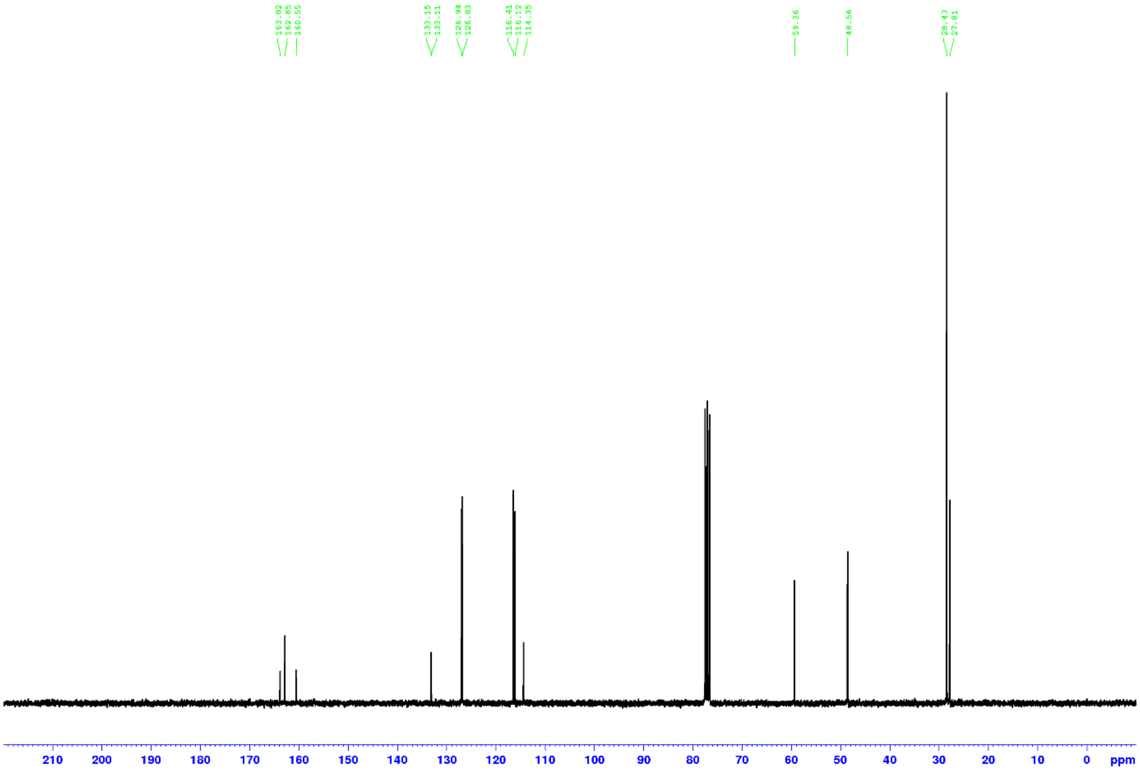

Figure S51. $^{13}C\{^{1}H\}$ NMR ($CDCl_3$, 75.5 MHz) spectrum of α-cyanoacetamide **50**.

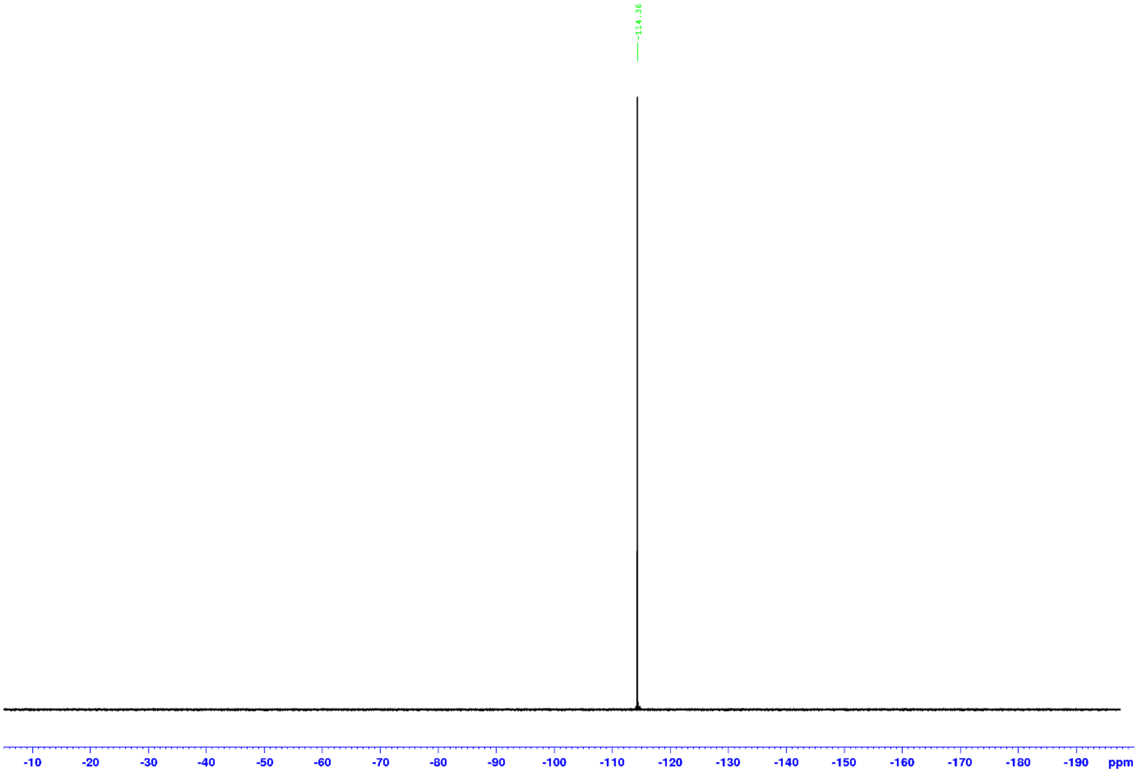

Figure S52. $^{19}F\{^{1}H\}$ NMR ($CDCl_3$, 376.5 MHz) spectrum of α-cyanoacetamide **50**.

N-(tert-Butyl)-2-cyano-N-(4-methylbenzyl)acetamide (51)[14]

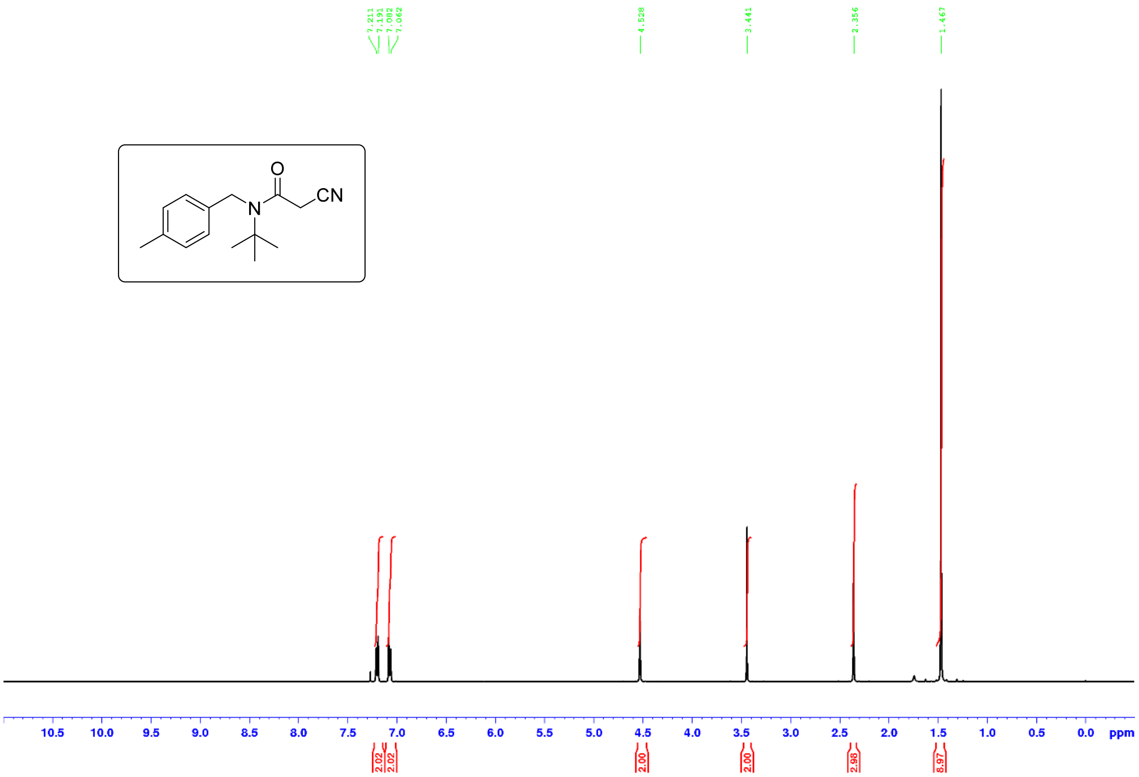

Figure S53. ^{1}H NMR ($CDCl_3$, 400 MHz) spectrum of α-cyanoacetamide **51**.

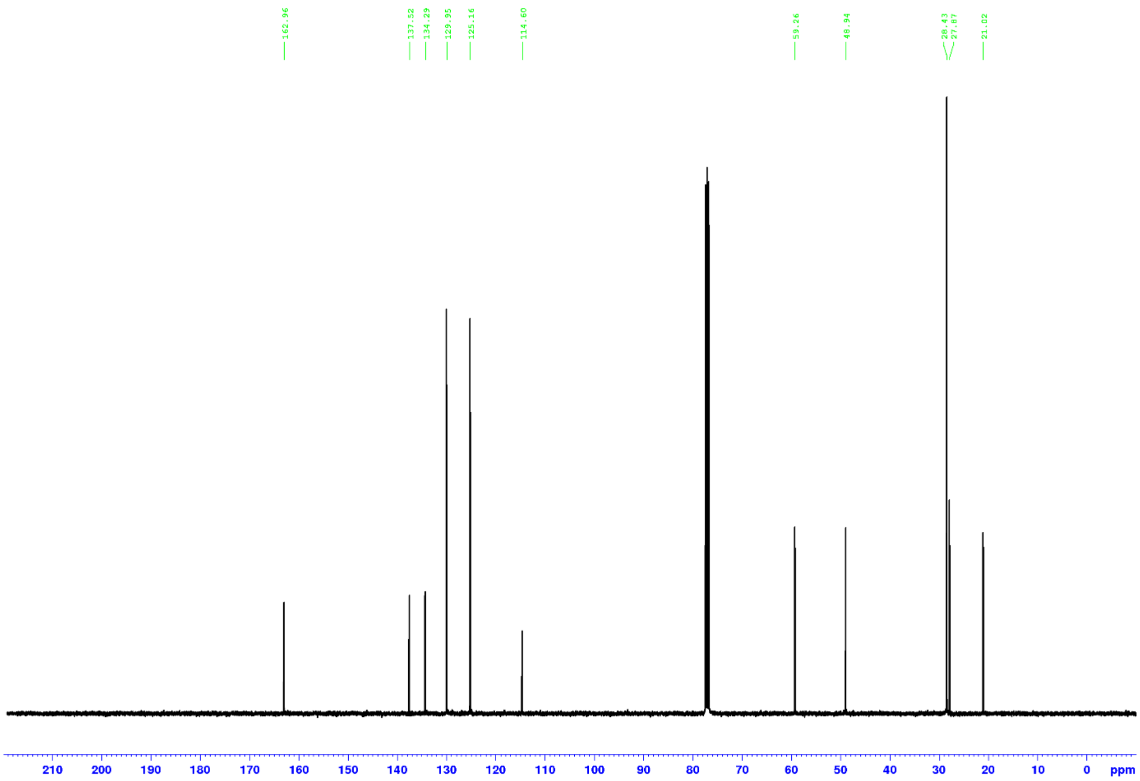

Figure S54. $^{13}C\{^1H\}$ NMR ($CDCl_3$, 100.6 MHz) spectrum of α-cyanoacetamide **51**.

N-(*tert*-Butyl)-2-cyano-N-(4-bromobenzyl)acetamide (52)[14]

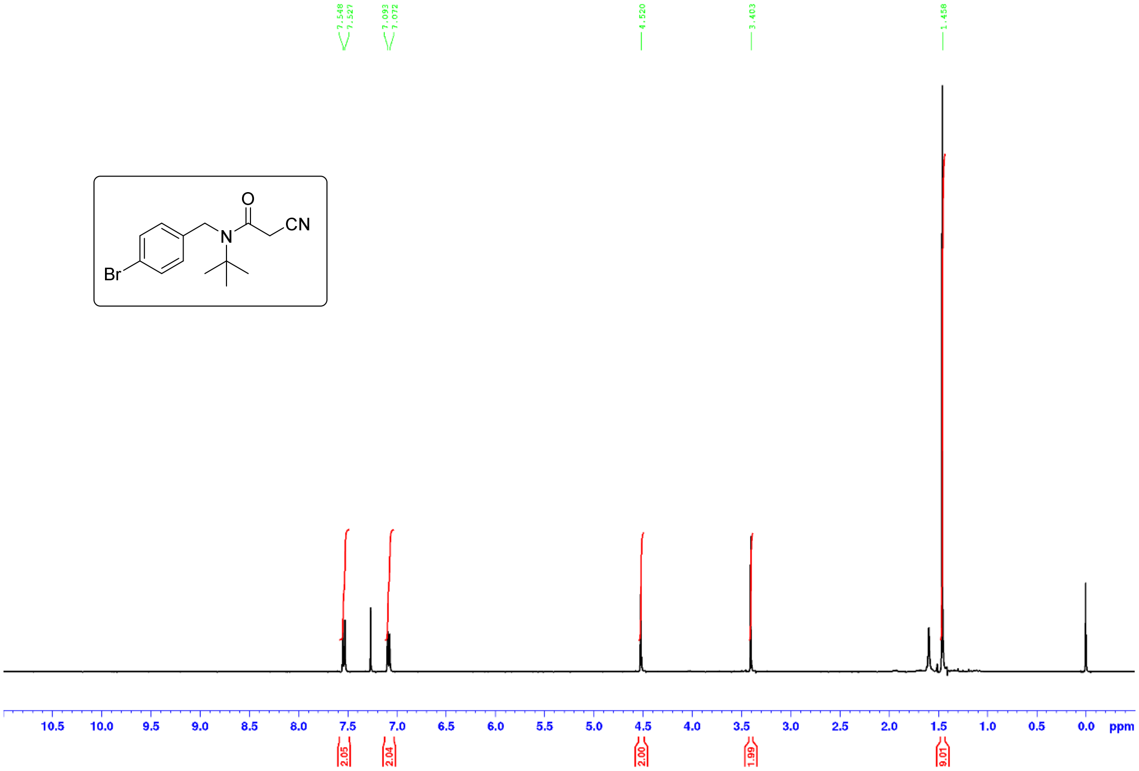

Figure S55. ^{1}H NMR ($CDCl_3$, 400 MHz) spectrum of α-cyanoacetamide **52**.

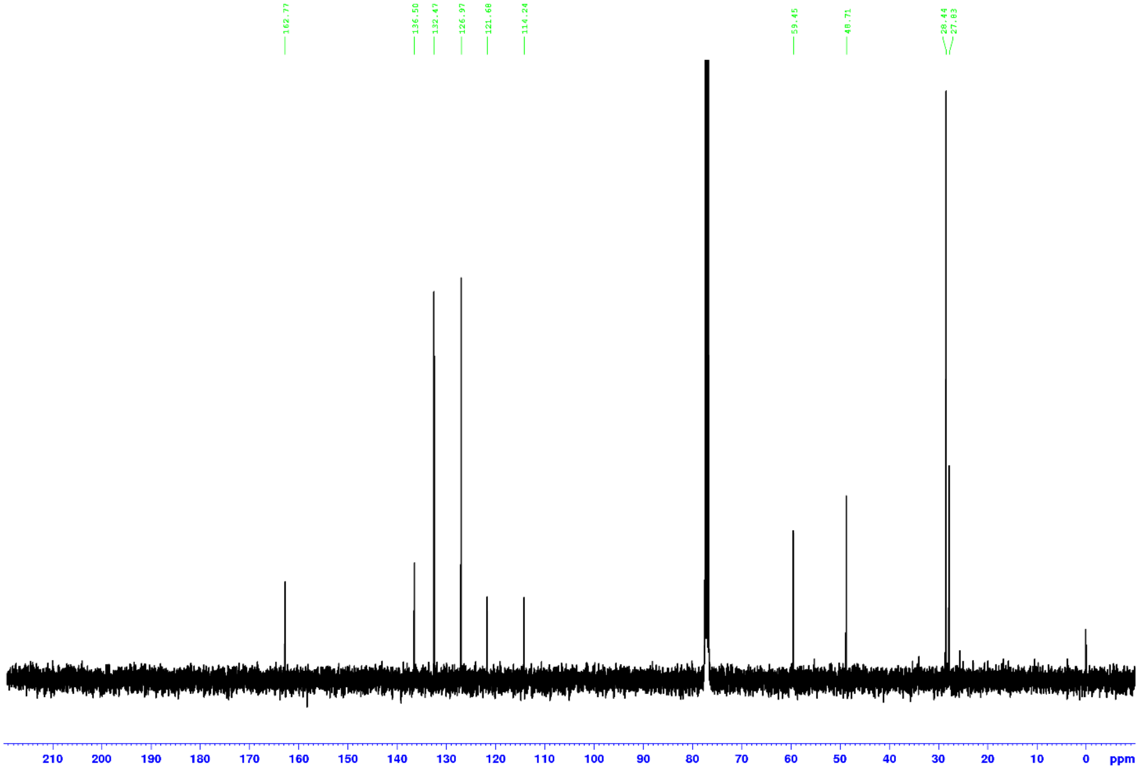

Figure S56. $^{13}C\{^{1}H\}$ NMR ($CDCl_3$, 100.6 MHz) spectrum of α-cyanoacetamide **52**.

N-(tert-Butyl)-2-cyano-N-(3,5-dimethylbenzyl)acetamide (53)

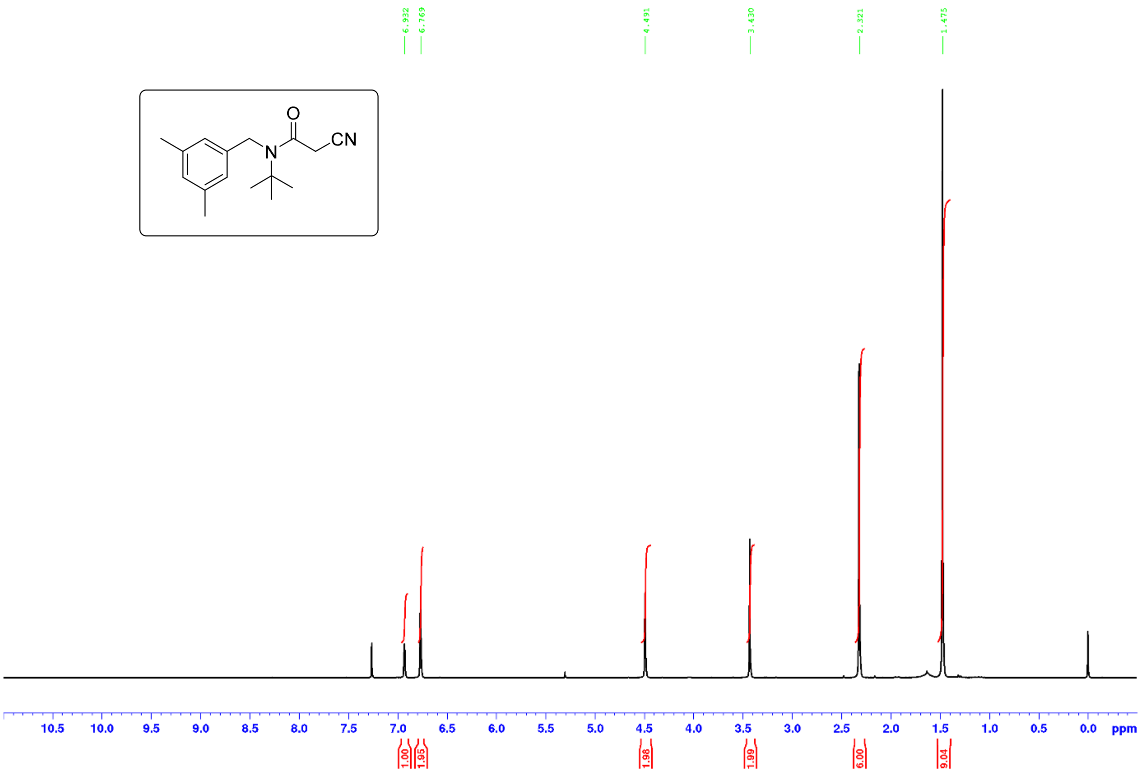

Figure S57. ^{1}H NMR (CDCl$_3$, 400 MHz, spectrum of α-cyanoacetamide **53**.

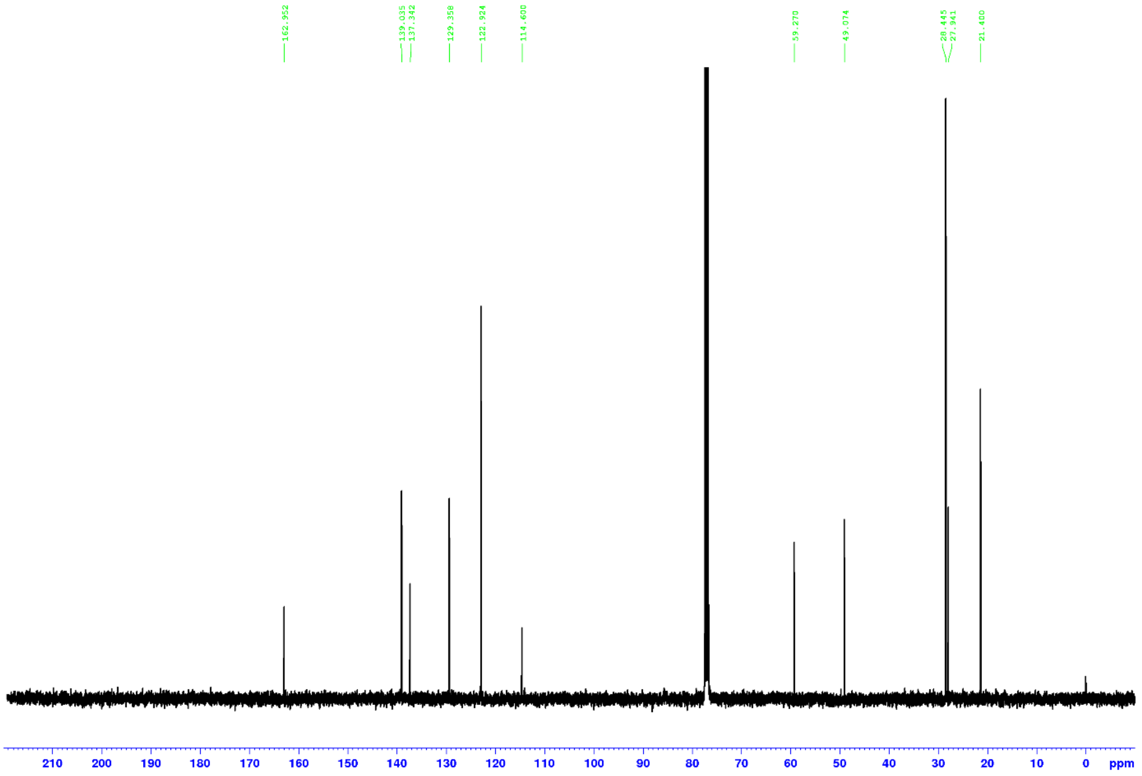

Figure S58. ^{13}C{^{1}H} NMR (CDCl$_3$, 100.6 MHz) spectrum of α-cyanoacetamide **53**.

***N-tert*-Butyl-2-cyano-*N*-(pyridin-4-ylmethyl)acetamide (54)**

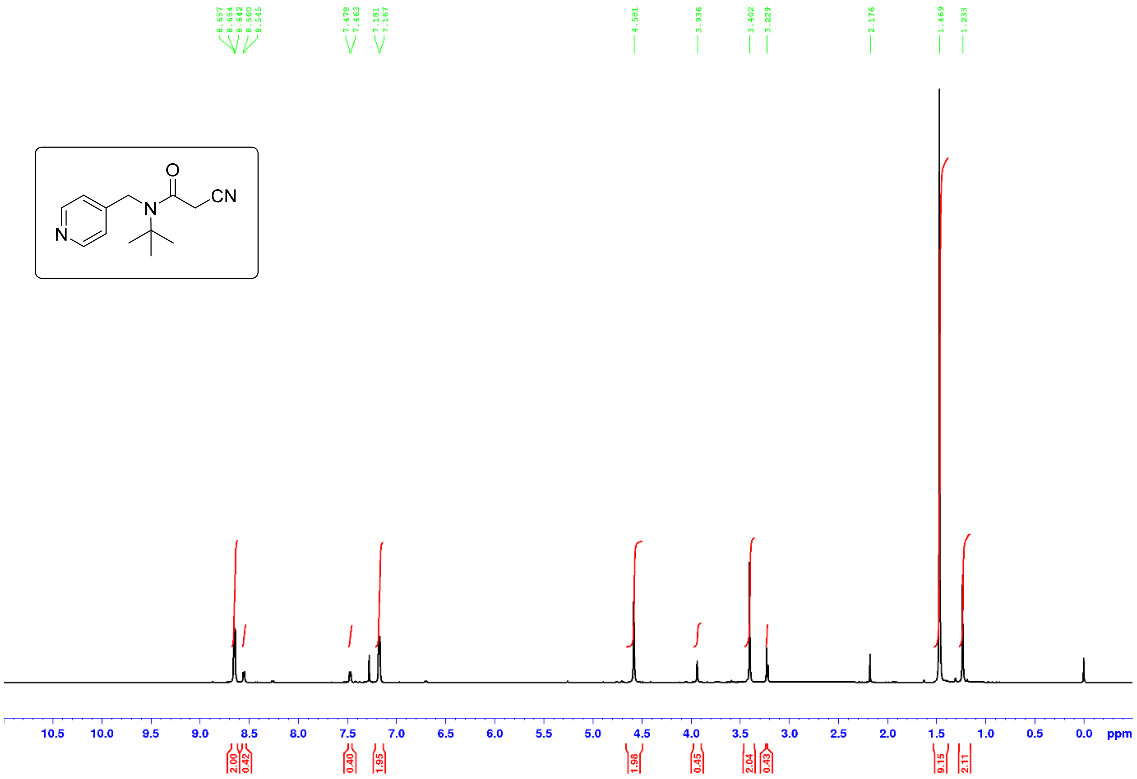

Figure S59. ^{1}H NMR ($CDCl_3$, 400 MHz) spectrum of α-cyanoacetamide **54**.

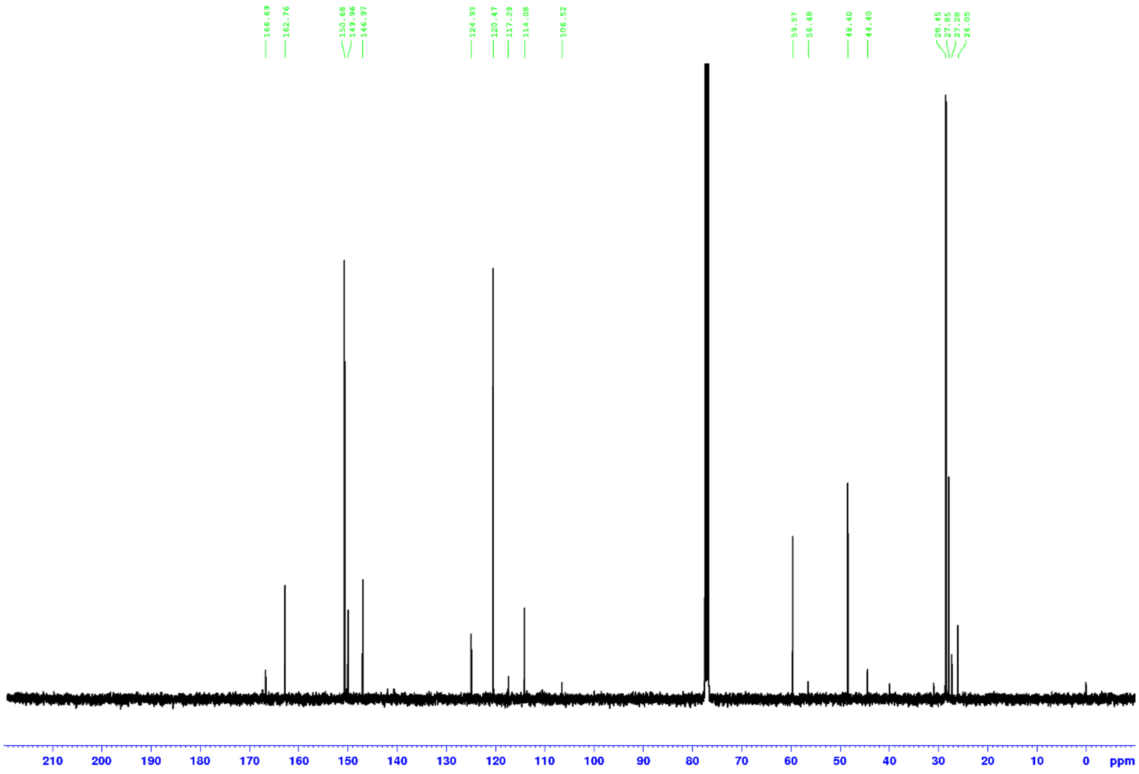

Figure S60. $^{13}C\{^{1}H\}$ NMR ($CDCl_3$, 100.6 MHz) spectrum of α-cyanoacetamide **54**.

***N*,*N*-Dibenzyl-2-cyano-2-diazoacetamide (55)**[14]

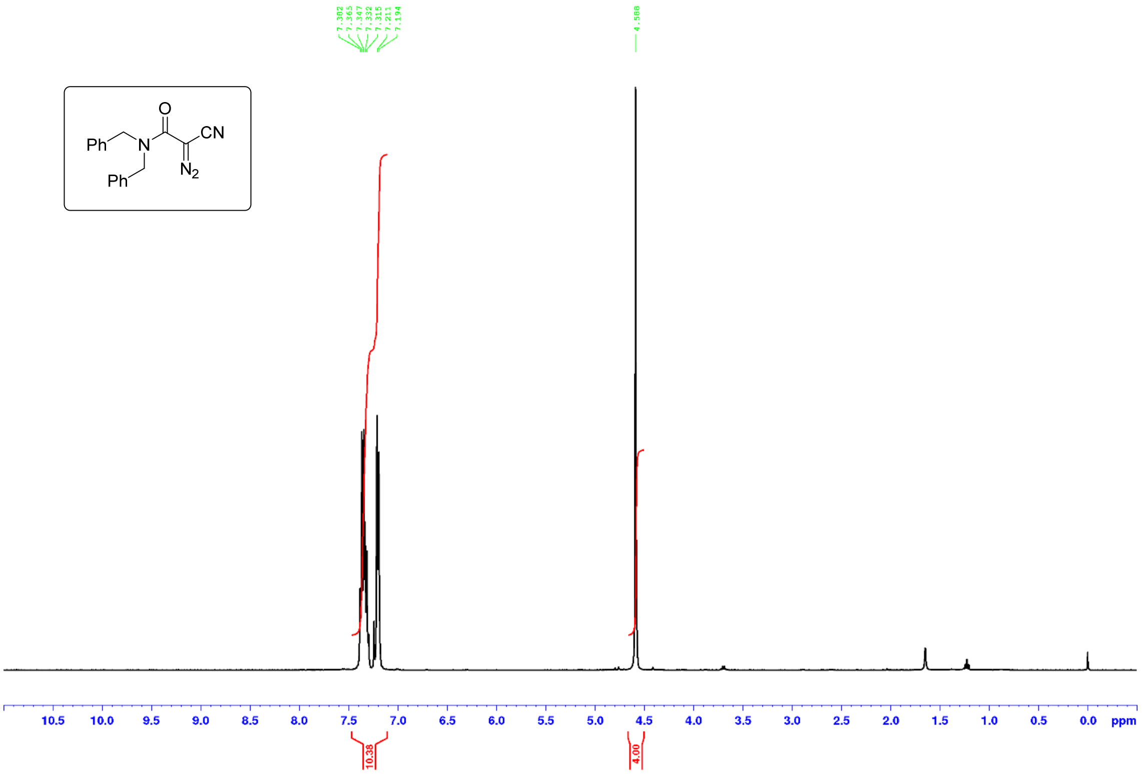

Figure S61. ^{1}H NMR ($CDCl_3$, 400 MHz) spectrum of α-diazoacetamide **55**.

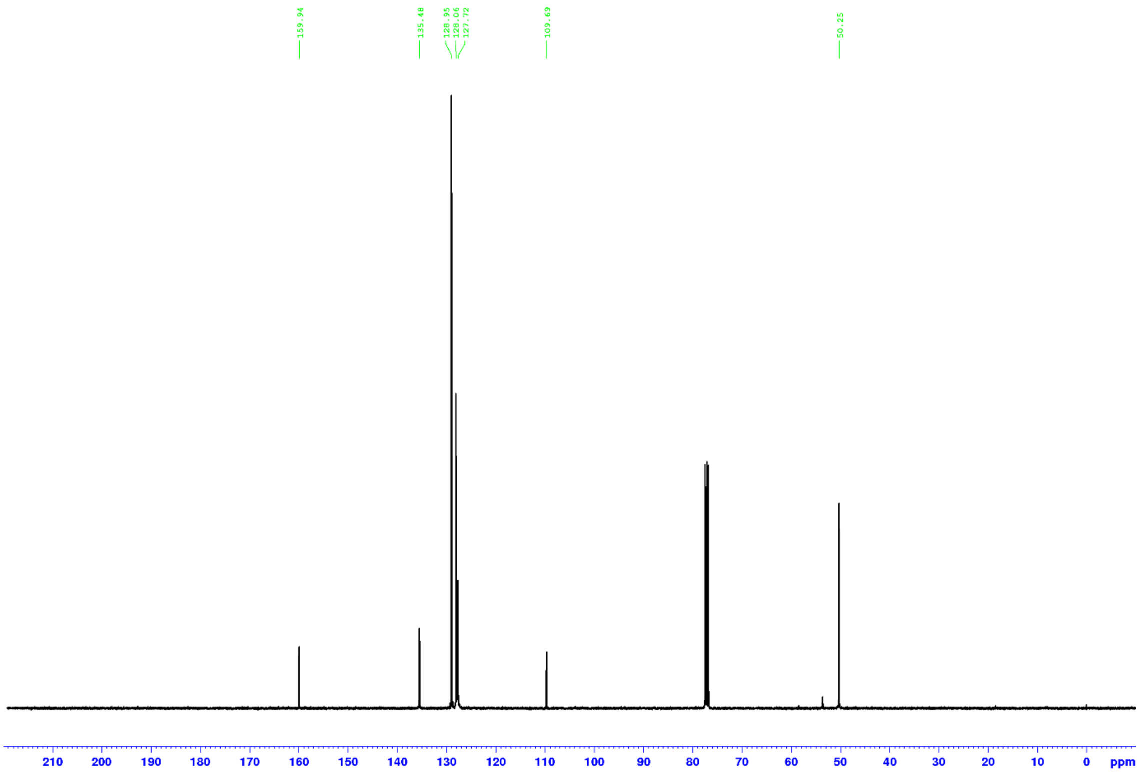

Figure S62. $^{13}C\{^{1}H\}$ NMR ($CDCl_3$, 100.6 MHz) spectrum of α-diazoacetamide **55**.

N-(*tert*-Butyl)-2-cyano-2-diazo-N-(4-chlorobenzyl)acetamide (56)[14]

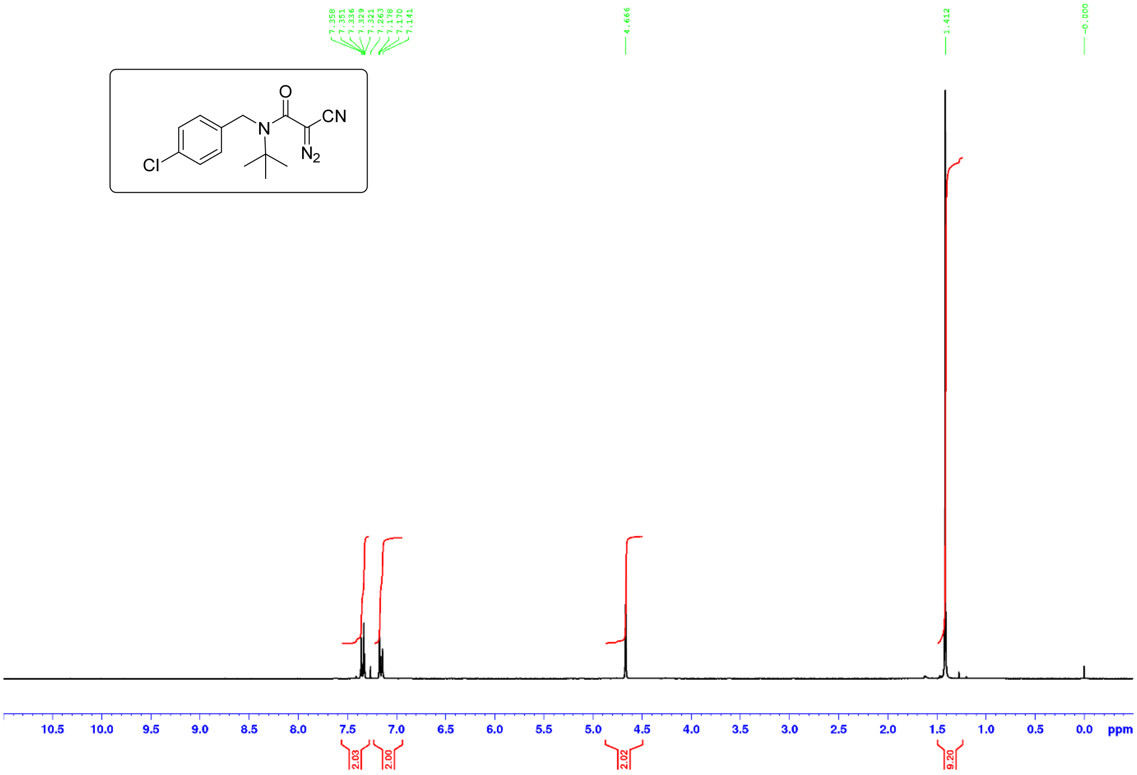

Figure S63. ^{1}H NMR ($CDCl_3$, 300 MHz) spectrum of α-diazoacetamide **56**.

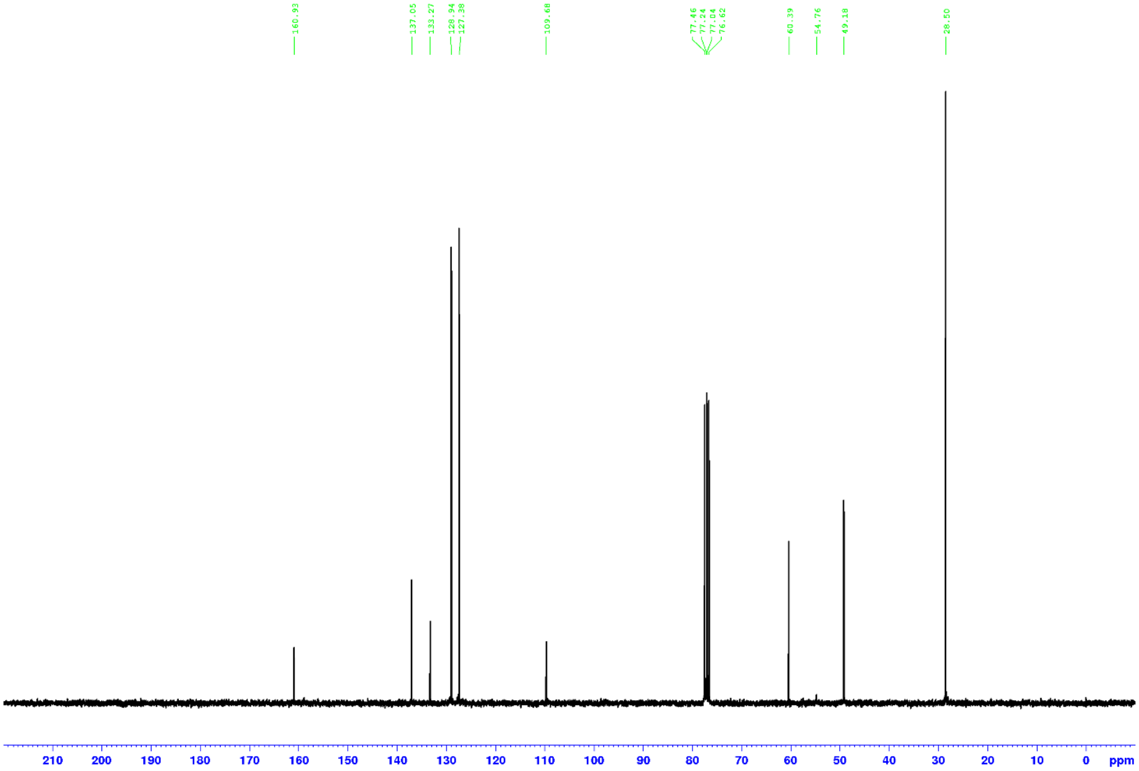

Figure S64. $^{13}C\{^1H\}$ NMR ($CDCl_3$, 75.5 MHz) spectrum of α-diazoacetamide **56**.

***N*-Benzyl-*N*-(*tert*-butyl)-2-cyano-2-diazoacetamide (57)**[14]

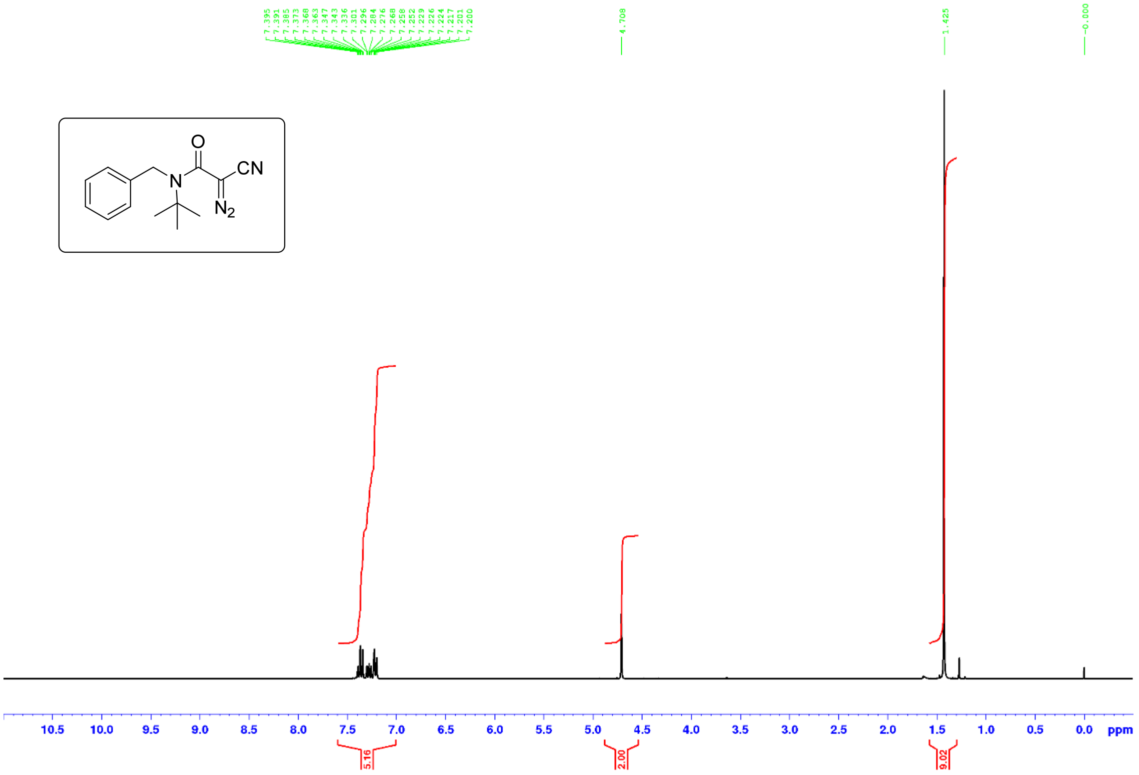

Figure S65. ^{1}H NMR ($CDCl_3$, 300 MHz) spectrum of α-diazoacetamide **57**.

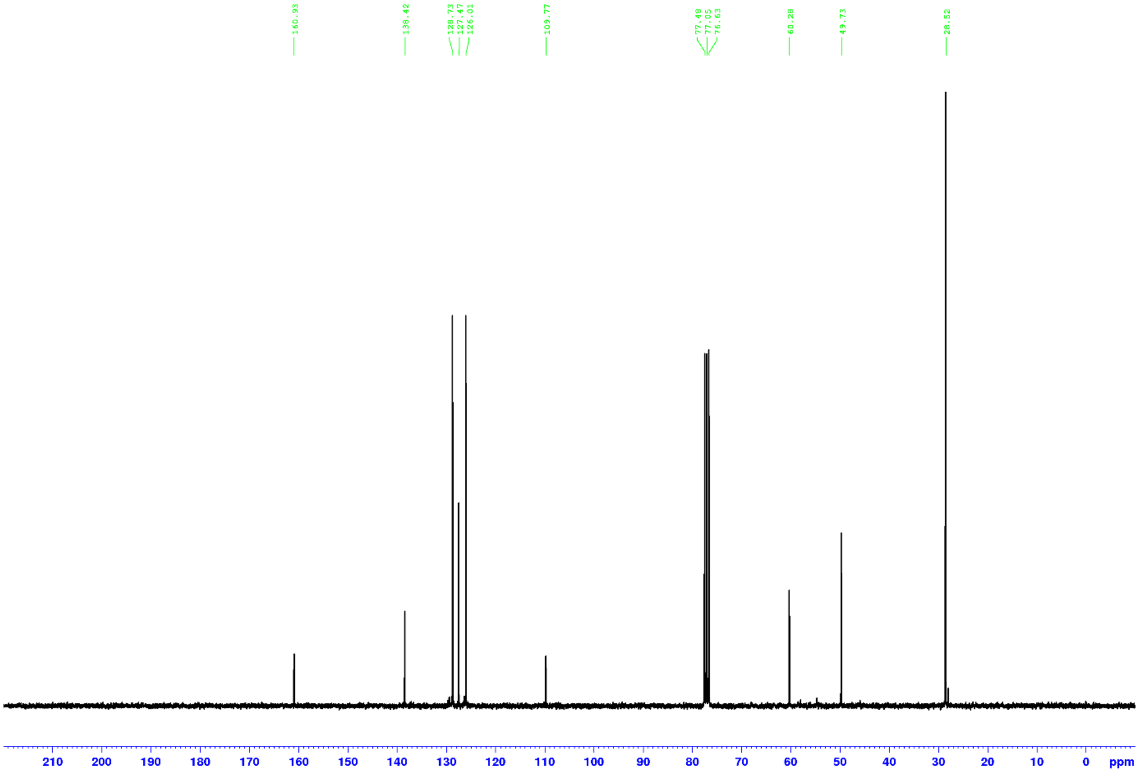

Figure S66. $^{13}C\{^{1}H\}$ NMR ($CDCl_3$, 75.5 MHz) spectrum of α-diazoacetamide **57**.

***N*-(*tert*-Butyl)-2-cyano-2-diazo-*N*-(4-fluorobenzyl)acetamide (58)[14]**

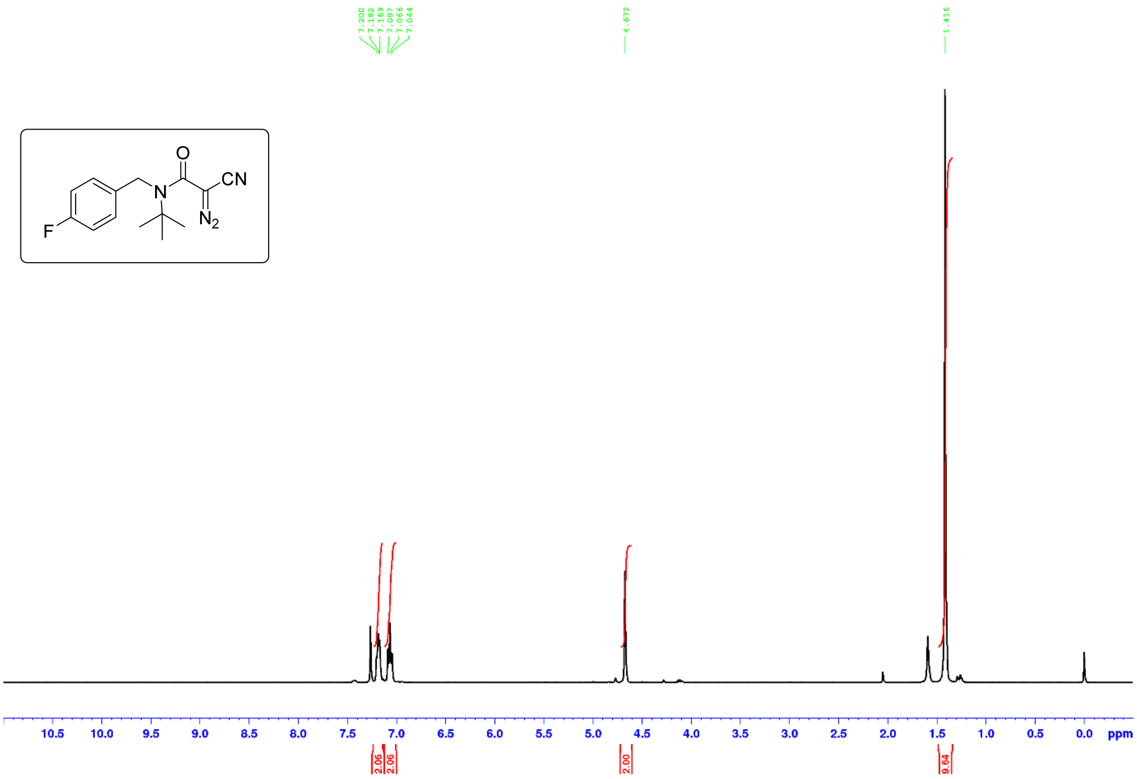

Figure S67. ^{1}H NMR ($CDCl_3$, 400 MHz) spectrum of α-diazoacetamide **58**.

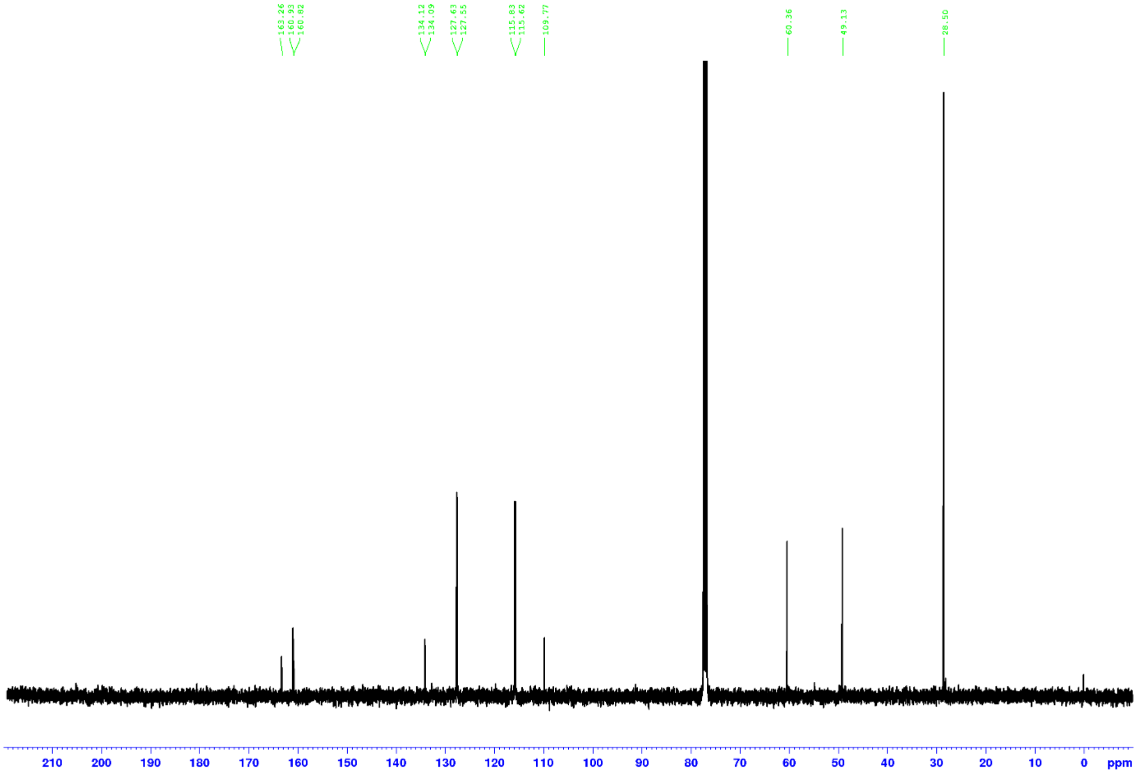

Figure S68. $^{13}C\{^{1}H\}$ NMR ($CDCl_3$, 100.6 MHz) spectrum of α-diazoacetamide **58**.

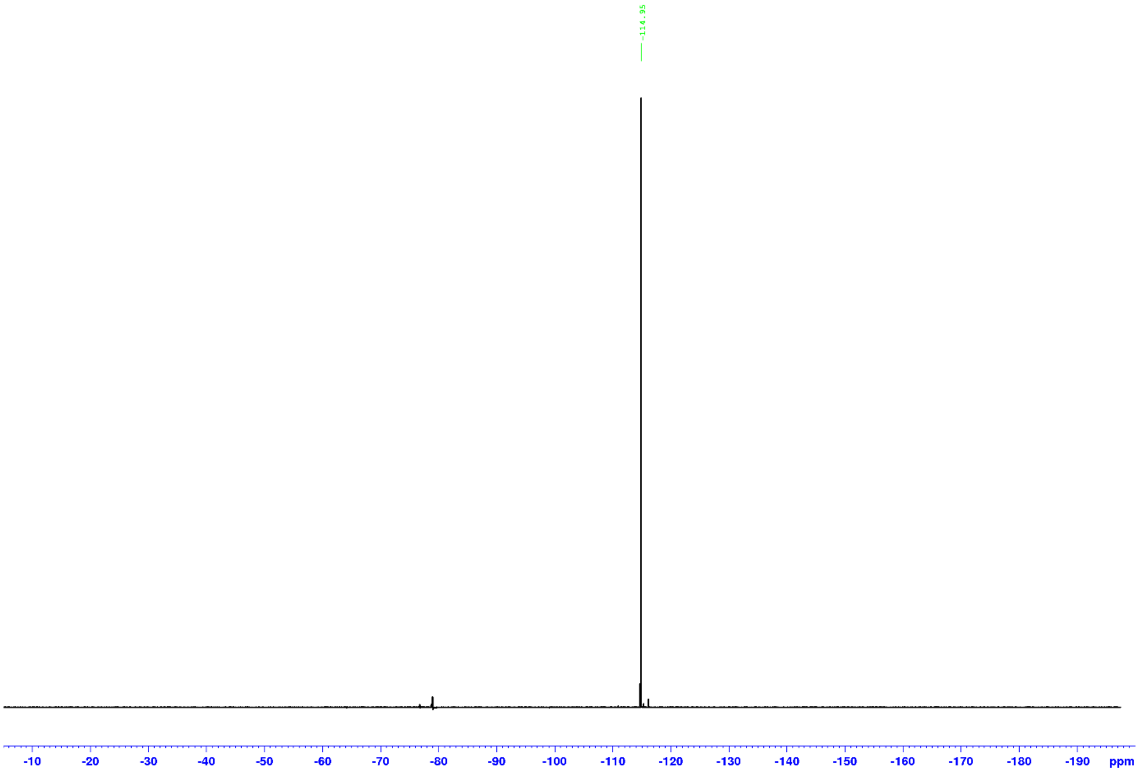

Figure S69. $^{19}F\{^1H\}$ NMR ($CDCl_3$, 376.5 MHz) spectrum of α-diazoacetamide **58**.

N-(*tert*-Butyl)-2-cyano-2-diazo-*N*-(4-methylbenzyl)acetamide (59)[14]

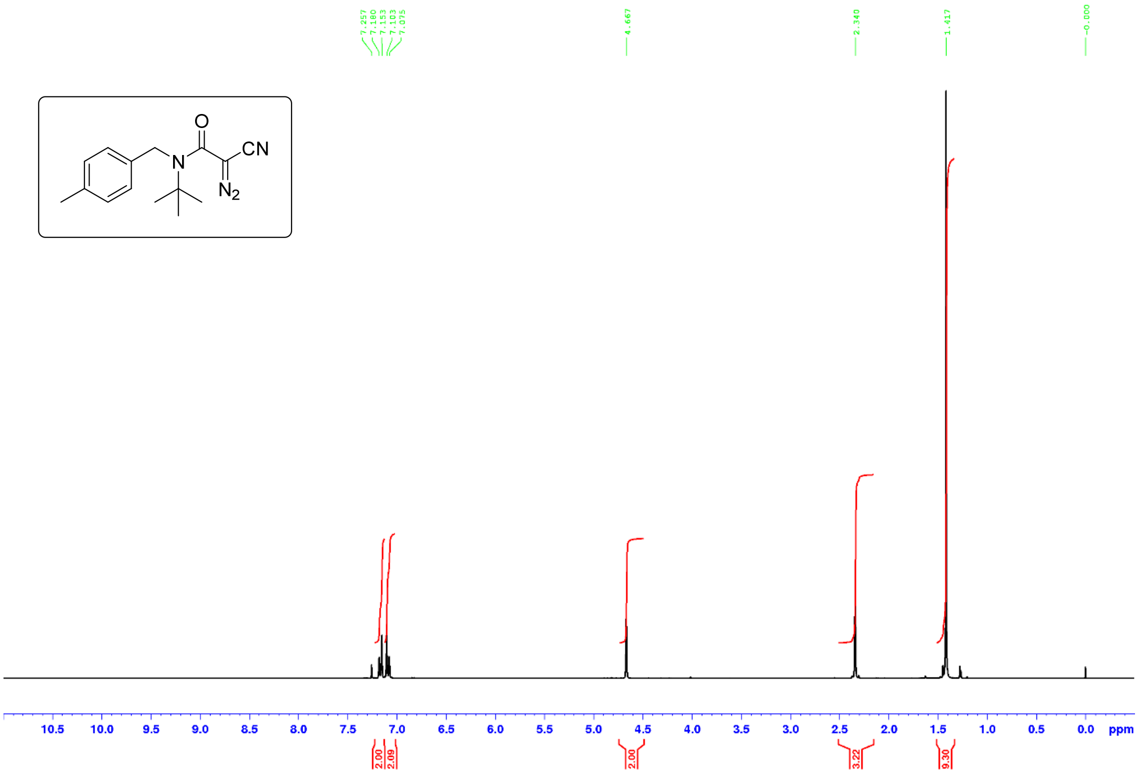

Figure S70. ^{1}H NMR ($CDCl_3$, 300 MHz) spectrum of α-diazoacetamide **59**.

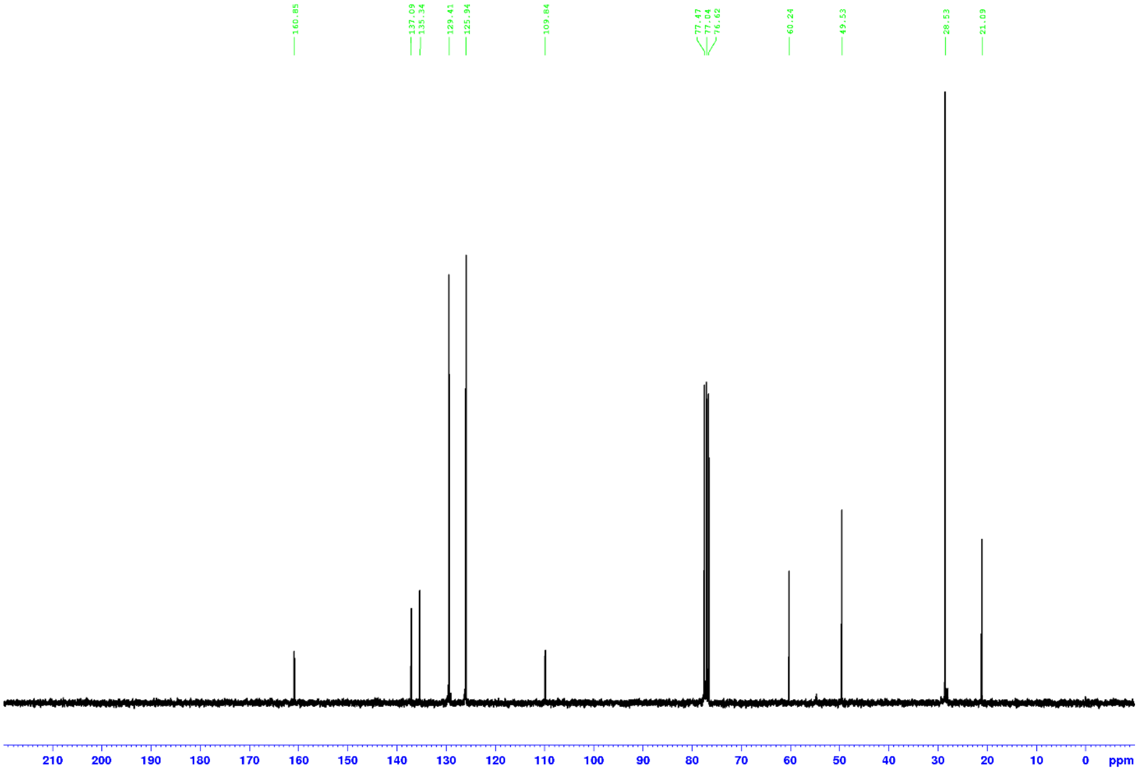

Figure S71. $^{13}C\{^{1}H\}$ NMR ($CDCl_3$, 100.6 MHz) spectrum of α-diazoacetamide **59**.

N-(*tert*-Butyl)-2-cyano-2-diazo-*N*-(4-bromobenzyl)acetamide (60)[14]

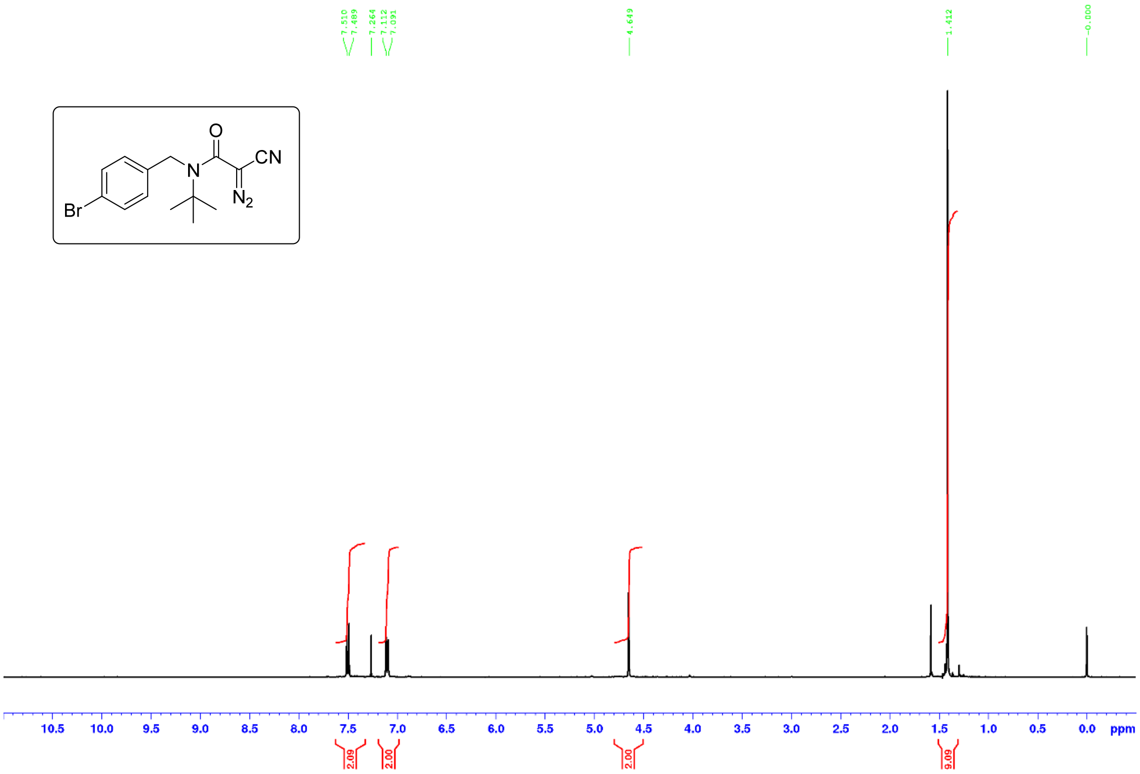

Figure S72. ^{1}H NMR ($CDCl_3$, 400 MHz) spectrum of α-diazoacetamide **60**.

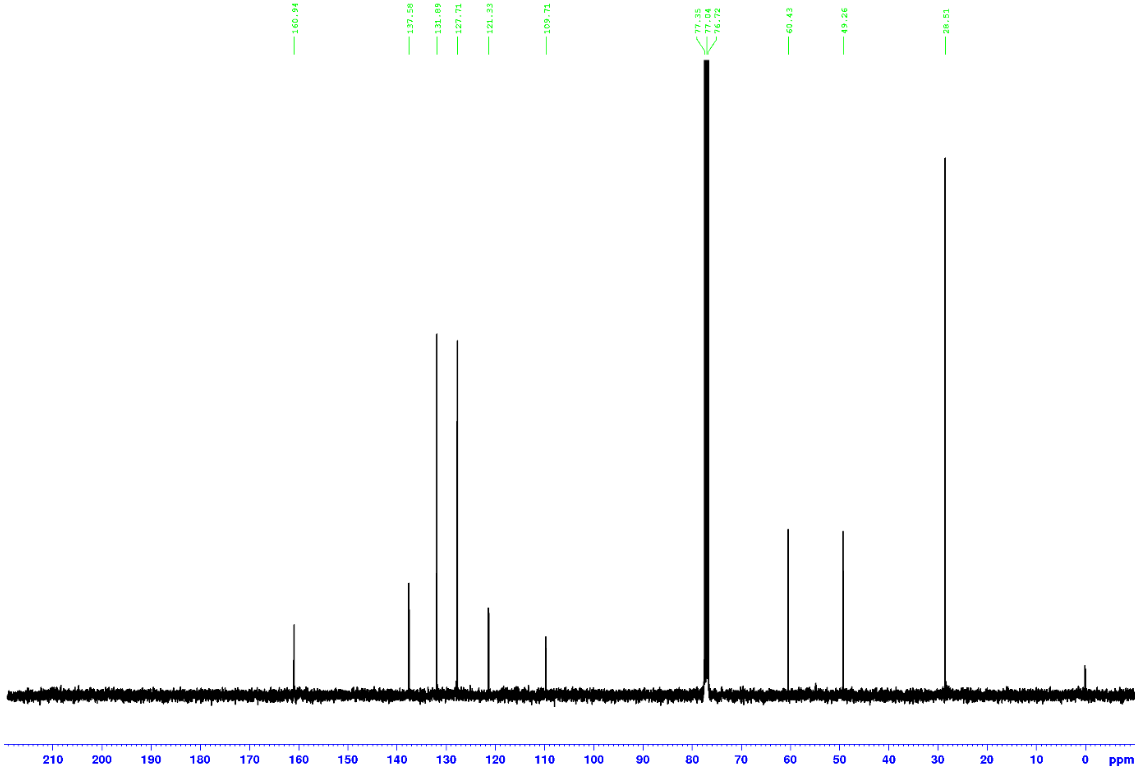

Figure S73. $^{13}C\{^{1}H\}$ NMR ($CDCl_3$, 100.6 MHz) spectrum of α-diazoacetamide **60**.

***N*-(*tert*-Butyl)-2-cyano-2-diazo-*N*-(3,5-dimethylbenzyl)acetamide (61)**

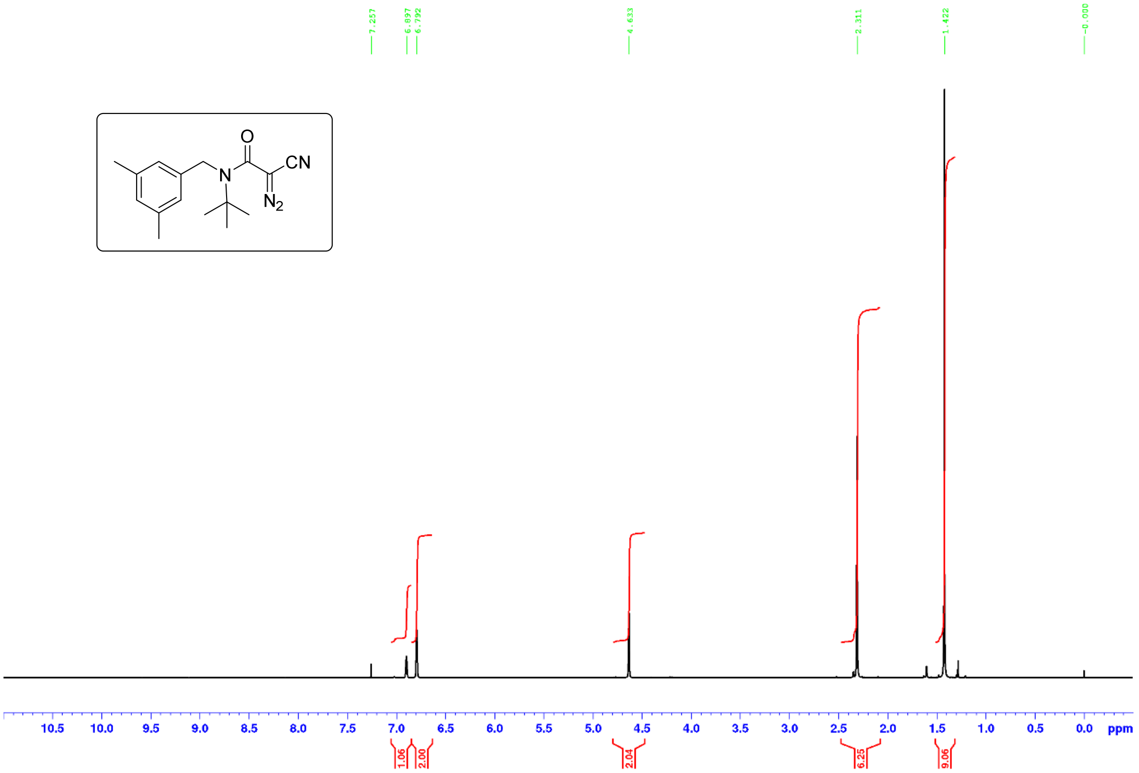

Figure S74. ^{1}H NMR (CDCl$_3$, 300 MHz) spectrum of α-diazoacetamide **61**.

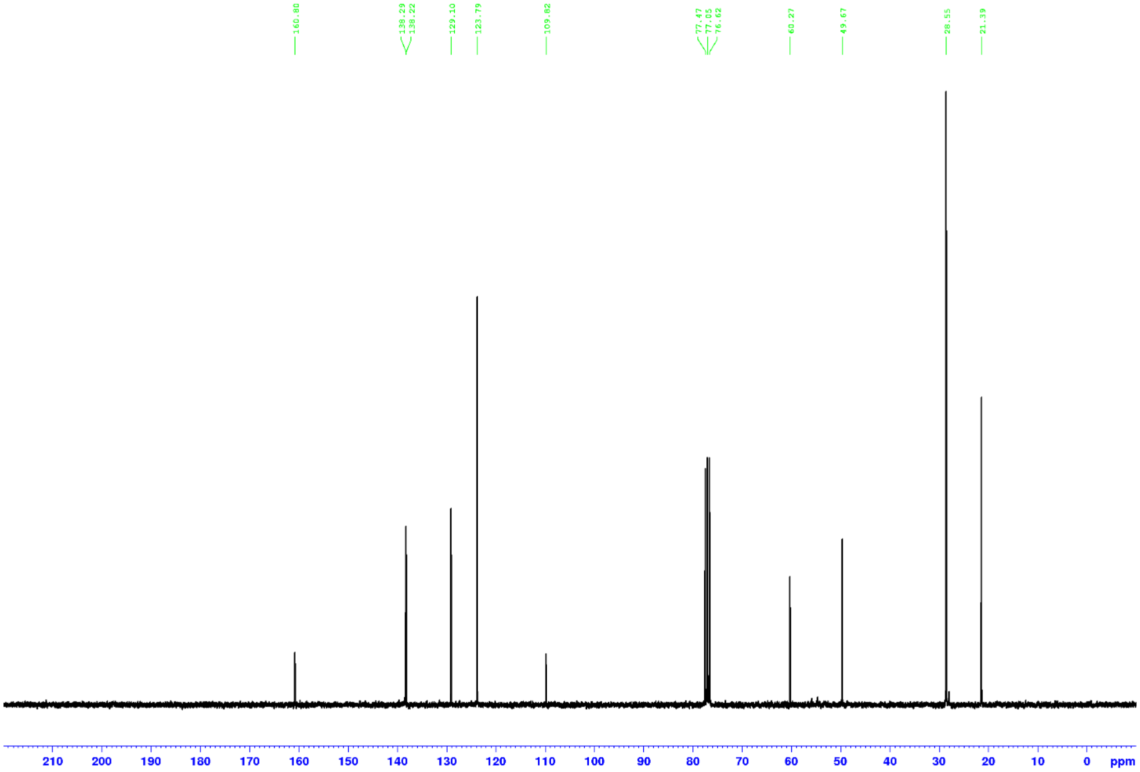

Figure S75. ^{13}C{^{1}H} NMR (CDCl$_3$, 100.6 MHz) spectrum of α-diazoacetamide **61**.

N-tert-Butyl-2-cyano-2-diazo-_N_-(pyridin-4-ylmethyl)acetamide (62)

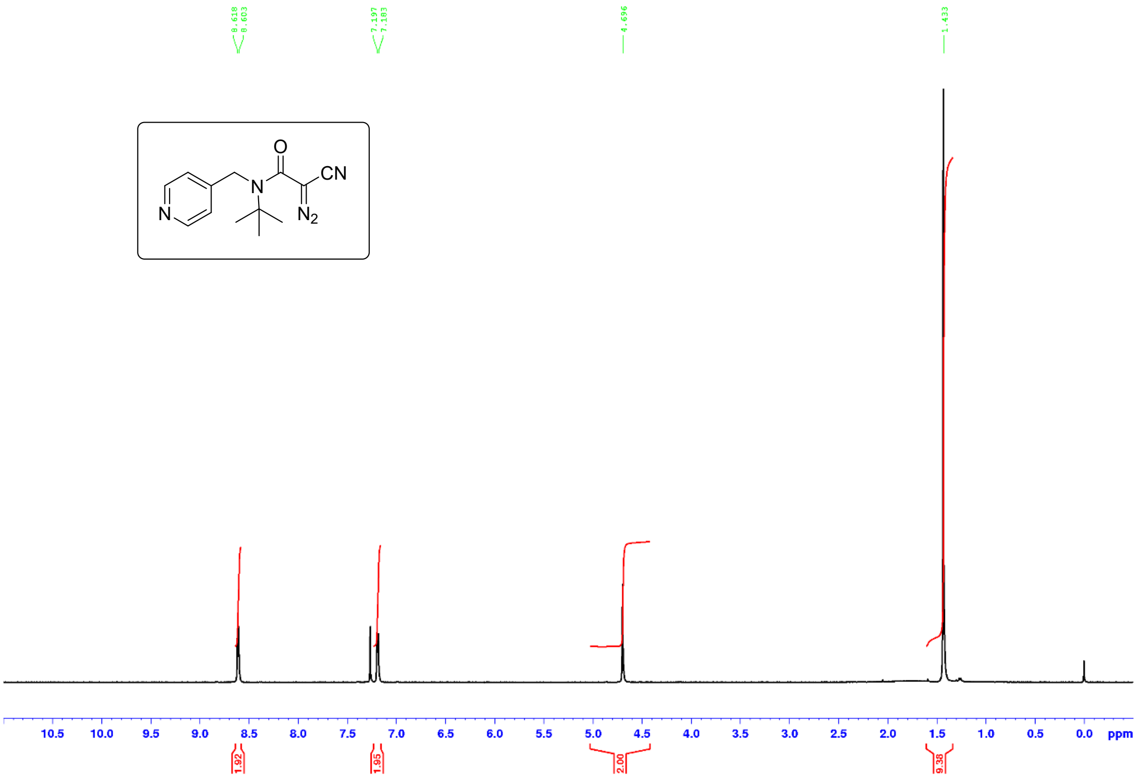

Figure S76. ^{1}H NMR ($CDCl_3$, 400 MHz) spectrum of α-diazoacetamide **62**.

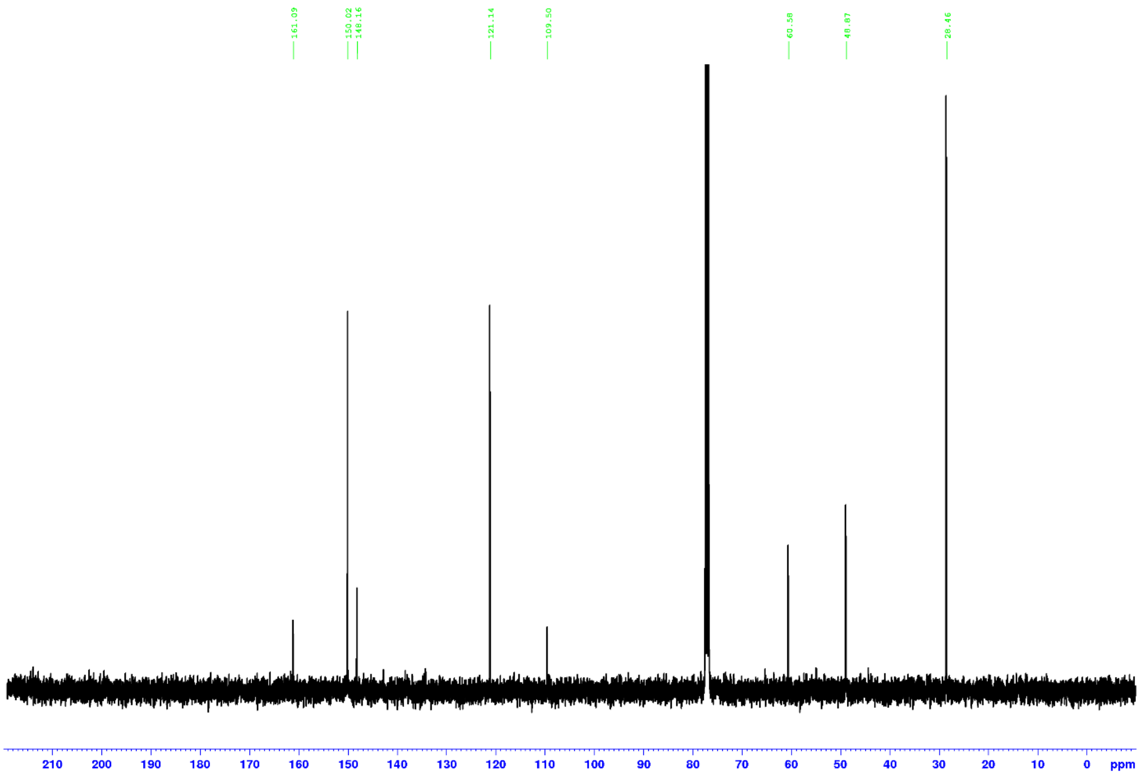

Figure S77. $^{13}C\{^{1}H\}$ NMR ($CDCl_3$, 100.6 MHz) spectrum of α-diazoacetamide **62**.

Methyl 2-((2-cyclohexylethyl)sulfonyl)-2-diazoacetate (63)[9]

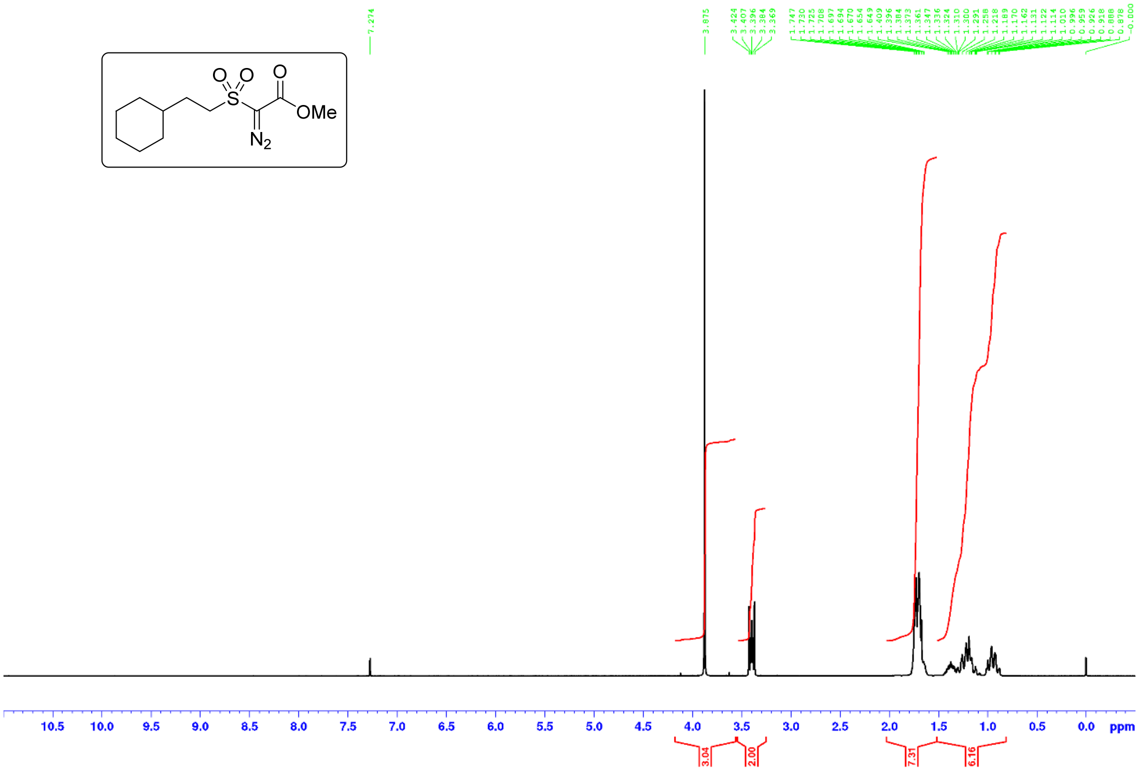

Figure S78. ^{1}H NMR ($CDCl_3$, 300 MHz) spectrum of α-diazosulfone **63**.

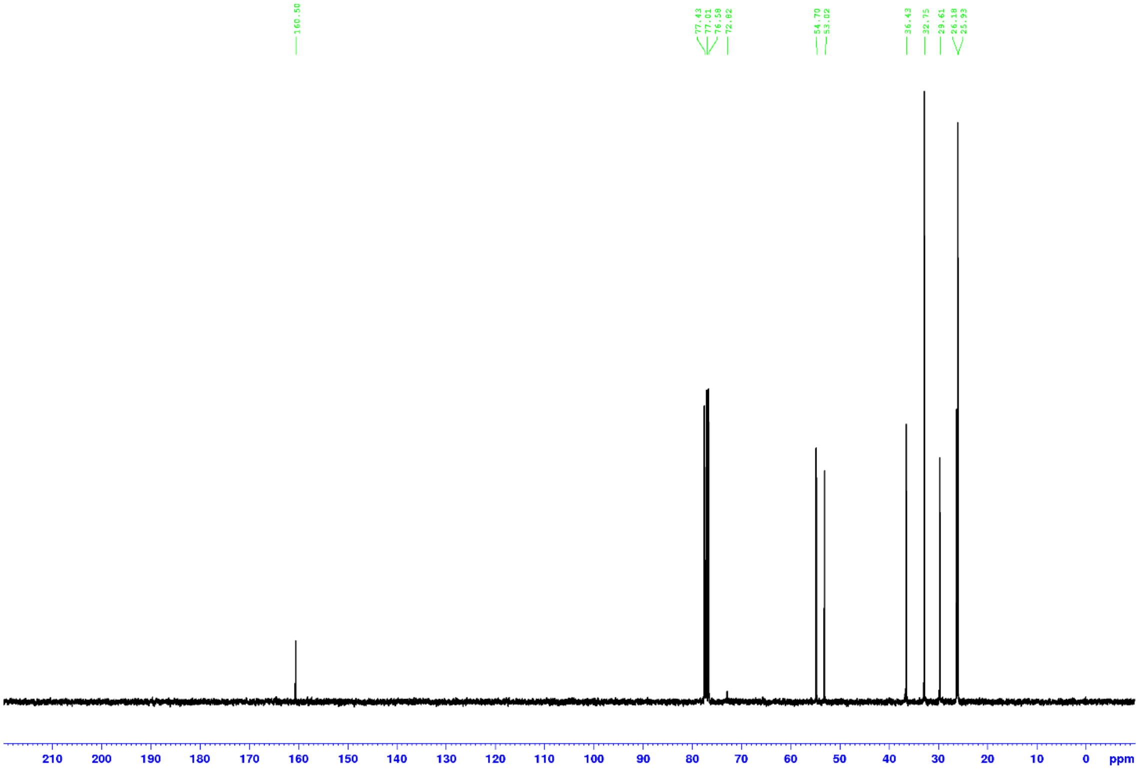

Figure S79. $^{13}C\{^{1}H\}$ NMR ($CDCl_3$, 75.5 MHz) spectrum of α-diazosulfone **63**.

Methyl (1*R,4a*R**,8a*S**)-octahydro-1H-isothiochromene-1-carboxylate 2,2-dioxide (65)**[9]

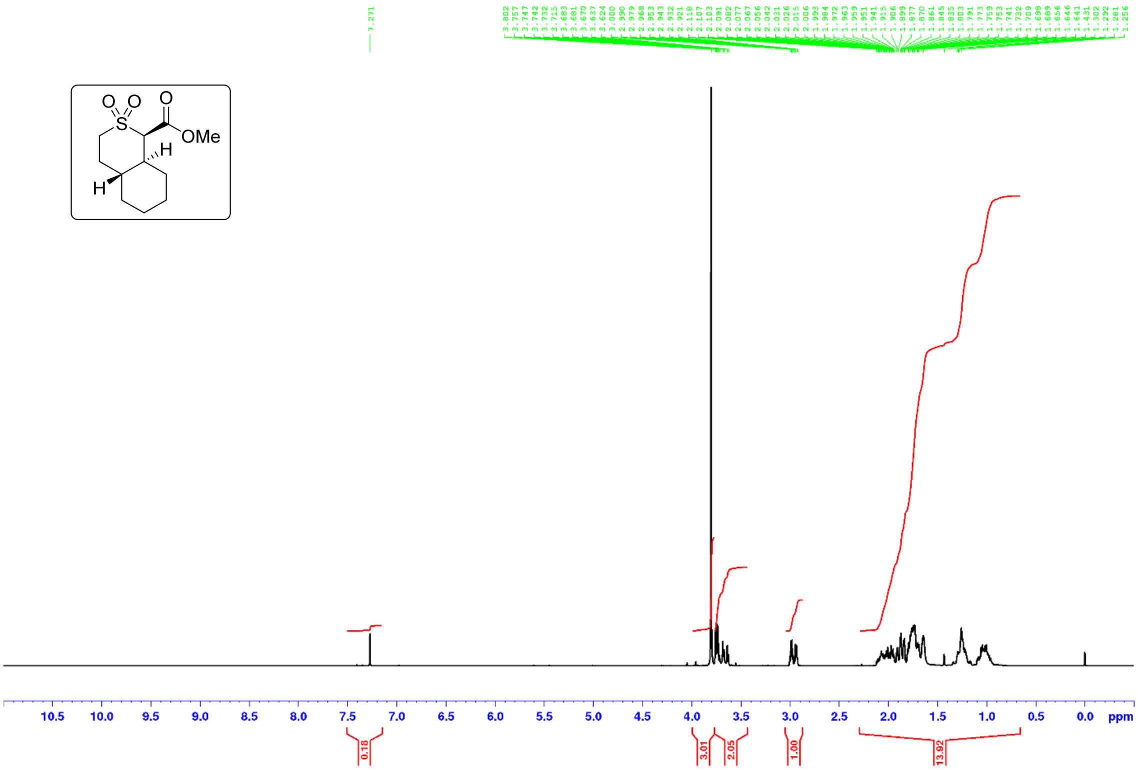

Figure S80. ^{1}H NMR ($CDCl_3$, 300 MHz) spectrum of thiopyran *S*,*S*-dioxide **65** isolated from the telescoped process for the synthesis and C–H insertion of α-diazo-β-oxosulfone **63** (Scheme 18); spectroscopic data were in agreement with those previously reported.[9]

References

(1) Conrow, R. E.; Dean, W. D. Diazidomethane Explosion. *Org. Process Res. Dev.* **2008**, *12*, 1285–1286.

(2) Hassner, A.; Stern, M.; Gottlieb, H. E.; Frolow, F. Synthetic methods. Utility of a polymeric azide reagent in the formation of di- and triazidomethane. Their NMR spectra and the X-ray structure of derived triazoles. *J. Org. Chem.* **1990**, *55*, 2304–2306.

(3) Mo, S.; Xu, J. Chemospecific Intramolecular Buchner Reaction Catalyzed by Copper(II) Acetylacetonate. *ChemCatChem* **2014**, *6*, 1679–1683.

(4) Liu, Q.; Tor, Y. Simple conversion of aromatic amines into azides. *Org. Lett.* **2003**, *5*, 2571–2572.

(5) Alper, P. B.; Hung, S.-C.; Wong, C.-H. Metal Catalyzed Diazo Transfer for the Synthesis of Azides from Amines. *Tetrahedron Lett.* **1996**, *37*, 6029–6032.

(6) NIST Chemistry WebBook, Standard Reference Database No. 69. U.S. Department of Commerce: 2018. DOI: https://doi.org/10.18434/T4D303

(7) *Prudent practices in the laboratory: handling and disposal of chemicals*. (U.S.), National Research Council, National Academy Press: Washington DC, 1995.

(8) Crowley, D. C.; Lynch, D.; Maguire, A. R. Copper-Mediated, Heterogeneous, Enantioselective Intramolecular Buchner Reactions of α-Diazoketones Using Continuous Flow Processing. *J. Org. Chem.* **2018**, *83*, 3794–3805.

(9) Brouder, T. A., Slattery, C. N.; Ford, A.; Khandavilli, U. B. R.; Skořepová, E.; Eccles, K. S.; Lusi, M, Lawrence, S. E.; Maguire, A. R. Desymmetrization by Asymmetric Copper-Catalyzed Intramolecular C−H Insertion Reactions of α-Diazo-β-oxosulfones. *J. Org. Chem.* **2019**, *84*, 7543–7563.

(10) Katritzky, A. R.; Zhang, Y.; Singh, S. K. Efficient Conversion of Carboxylic Acids into *N*-Acylbenzotriazoles. *Synthesis* **2003**, 2795−2344.

(11) Katritzky, A. R.; Pastor, A. Synthesis of β-Dicarbonyl Compounds Using 1-Acylbenzotriazoles as Regioselective C-Acylating Reagents. *J. Org. Chem.* **2000**, *65*, 3679−3682.

(12) Zhukovsky, D.; Dar'in, D.; Kantin, G.; Krasavin, M. Synthetic exploration of α-diazo γ-butyrolactams*.* *Eur. J. Org. Chem.* **2019**, *2019*, 2397−2400.

(13) McNamara, O. A.; Buckley, N. R.; O'Leary, P.; Harrington, F.; Kelly, N.; O'Keeffe, S.; Stack, A.; O'Neill, S.; Lawrence, S. E.; Slattery, C. N.; Maguire, A. R. Catalyst and substituent effects on the rhodium(II)-catalysed intramolecular Buchner reaction. Tetrahedron **2014**, *70*, 6870−6878.

(14) Mo, S.; Xu, J. Chemospecific Intramolecular Buchner Reaction Catalyzed by Copper(II) Acetylacetonate. *ChemCatChem* **2014**, *6*, 1679−1683.
